# Identifying and presenting key country-specific indicators related to medication adherence: a comprehensive study across European countries

**DOI:** 10.3389/fphar.2024.1390629

**Published:** 2024-10-04

**Authors:** Tamás Ágh, Kristina Garuolienė, Anne Gerd Granas, João Gregório, Nilay Aksoy, Nataliia Khanyk, Maja Ortner Hadžiabdić, Przemyslaw Kardas, European Network to Advance Best Practices and Technology on Medication Adherence (ENABLE) Collaborators, Emma Aarnio

**Affiliations:** ^1^ Syreon Research Institute, Budapest, Hungary; ^2^ Pharmacy Center, Institute of Biomedical Science, Faculty of Medicine, Vilnius University, Vilnius, Lithuania; ^3^ Section for Pharmaceutics and Social Pharmacy, Department of Pharmacy, University of Oslo, Oslo, Norway; ^4^ Norwegian Centre for E-health Research, University Hospital of North Norway, Tromsø, Norway; ^5^ CBIOS–Universidade Lusófona’s Research Center for Biosciences and Health Technologies, Lisboa, Portugal; ^6^ Department of Clinical Pharmacy, Faculty of Pharmacy, Altinbas University, Istanbul, Türkiye; ^7^ Department of Pharmacy, Uppsala University, Uppsala, Sweden; ^8^ Department of Pharmacy, Danylo Halytsky Lviv National Medical University, Lviv, Ukraine; ^9^ Department of Applied Pharmacy, Faculty of Pharmacy and Biochemistry, University of Zagreb, Zagreb, Croatia; ^10^ Department of Family Medicine, Medication Adherence Research Centre, Medical University of Lodz, Lodz, Poland

**Keywords:** medication adherence, persistence, health policy, indicator, Europe

## Abstract

This study tackles the critical challenge of medication non-adherence in healthcare by pinpointing indicators related to medication adherence (IRMAs) across 39 European countries and Israel. Utilizing a structured expert survey methodology within the European Network to Advance Best Practices and Technology on Medication Adherence (ENABLE; COST Action CA19132), our research identified key country-specific IRMAs and collected data on these indicators to understand the multifaceted nature of medication adherence. The research was conducted in two phases: firstly, defining key IRMAs through a two-round expert survey, and secondly, gathering country-specific data on these IRMAs through literature reviews and additional expert surveys. The study revealed a diverse range of 26 top-ranked IRMAs, including six related to country characteristics, four to social/economic factors, three each to therapy-related and patient-related factors, one to condition-related factors, and nine to healthcare system-related factors. The availability of country-specific data on these IRMAs varied among the countries, highlighting the need for more comprehensive data collection and research. The findings from this study not only underscore the complexity of predicting medication adherence but also lay the groundwork for developing targeted, country-specific interventions to improve adherence. Moreover, this research offers valuable insights for policymakers, highlighting the importance of understanding the multifaceted nature of medication adherence and offering a valuable resource in formulating targeted health policies to enhance health outcomes and reduce the economic burden associated with medication non-adherence.

## Introduction

Adherence, as defined by the ABC taxonomy of the International Society for Medication Adherence (ESPACOMP), pertains to patients adhering to their prescribed medication regimen (Vrijens et al., 2012). This encompasses three phases: initiation, implementation, and discontinuation. Initiation marks the patient consuming the initial dose of the prescribed medication. Discontinuation marks the deliberate cessation of the medication by the patient. Implementation measures the degree to which a patient’s actual intake of medication aligns with the prescribed dosing schedule, spanning from the initiation to the last administered dose. Research highlights that medication adherence rates for chronic disorders, such as hypertension, diabetes mellitus, or cardiovascular diseases, often fall below optimal levels. Approximately 50% of patients fail to adhere to their prescribed medication regimens (WHO, 2003; Foley et al., 2021).

Medication non-adherence is associated with higher morbidity and mortality rates, leading to deteriorating health outcomes, progression of diseases, worsening symptoms, and reduced therapy effectiveness (Mongkhon et al., 2018; Inotai et al., 2021). Consequently, healthcare costs escalate as non-adherent patients may require more expensive treatments, longer hospital stays, increased emergency room visits, or face more severe complications from untreated diseases (Mikyas et al., 2014; Cutler et al., 2018). Moreover, patients may experience a decline in their overall quality of life (Ágh et al., 2011; Márquez-Contreras et al., 2017). Medication non-adherence poses significant implications for individual patients, healthcare professionals and healthcare system. The financial impact of non-adherence is substantial, with 80–125 billion EUR lost annually in Europe due to increased use of healthcare resources and the emergence of preventable health problems (European Commission, 2011).

Medication non-adherence stands out as a significant issue in modern medicine, representing a critical challenge to the sustainability of existing healthcare systems (Stewart et al., 2023). The persistence of this problem undermines the potential advantages of medical interventions and places strain on the overall viability and efficiency of contemporary healthcare systems. Addressing this concern becomes paramount for the effective functioning of healthcare structures. Achieving progress and resilience in clinical practices necessitates a comprehensive understanding and strategic response to medication non-adherence, establishing these elements as imperative pillars in fortifying the foundations of healthcare systems.

Medication adherence is a complex issue (Kardas et al., 2013; Gast and Mathes, 2019), which–according to the World Health Organization (WHO) model (WHO, 2003)–is influenced by multiple factors including socio-economic, healthcare team and system-related, condition-related, therapy-related, and patient-related considerations. Given the intricate factors influencing this problem, it is essential to prioritize comprehensive strategies that effectively address these challenges to improve medication adherence and, thereby, health outcomes. Understanding non-adherence statistics and identifying potential indicators at the country level are critical for tailoring health policies that are sensitive to socio-economic differences, cater to the unique needs of populations, and to gain a better understanding of the causes of non-adherence. Through such targeted approaches, significant enhancement of health outcomes and reduction of the economic burden associated with non-adherence could be achieved.

In the context of addressing these challenges, the European Network to Advance Best Practices and Technology on Medication Adherence (ENABLE), a COST Action supported by the European Commission, is as a pivotal initiative. ENABLE aims to foster best practices and technological advancements in medication adherence, emphasizing the importance of interdisciplinary understanding, the application of innovative technologies in clinical settings, and the development of economically viable policies for the adoption of adherence-enhancing technologies in healthcare systems (van Boven et al., 2021). Aligned with these efforts, our study focuses on identifying key country-specific indicators associated with medication adherence [referred to as country-specific indicators related to medication adherence (IRMAs)] and presenting country-specific data on these key IRMAs for European countries and Israel. By doing so, we aim to contribute to a more nuanced understanding of medication adherence, facilitating targeted interventions that address the specific needs and challenges within these countries.

## Material and methods

### Study design

This mixed-methods study was designed around two main phases: (i) identification of IRMAs through a two-round online expert survey, and (ii) collection of country-specific data on IRMAs through a targeted literature review and data validation by an online expert survey.

**FIGURE 1:**
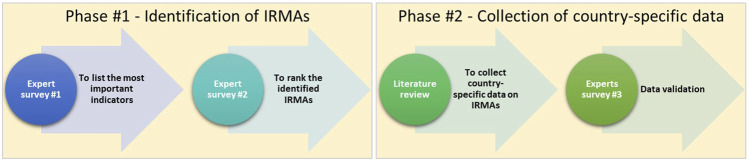
Study flow diagram IRMA, indicator related to medication adherence.

The initial phase of the study involved a two-round expert survey approach to define the key IRMAs specific to each country (i.e., all 39 European countries and Israel; Figure 1). In the Expert Survey #1, participants were requested to itemize significant indicators spanning multiple domains, such as country characteristics, as well as the WHO model-related dimensions, i.e., socio-economic, therapy-related, patient-related, condition-related, and healthcare system-related factors (WHO, 2003). Subsequently, during the Expert Survey #2, participants ranked the relevance of identified indicators on a 5-point Likert scale, with 1 representing “not relevant at all” and 5 indicating “extremely relevant.” Indicators with a mean score of ≥3.5 were classified as key IRMAs. The outcomes of this round facilitated the formulation of a definitive list of country-specific IRMAs, assuming that the intervals between response categories are equidistant. Concurrently, both terminology refinement and the development of comprehensive definitions for each indicator were undertaken through an iterative process by the research team. This process began with preliminary definitions crafted based on existing literature and the collective expertise within our team. These definitions served as the initial framework and were continually refined and modified throughout the study, especially after the data collection phase. The refinement was significantly influenced by the definitions present in the data sources we utilized, ensuring our terminology aligned with prevailing standards. Importantly, this process of refining and validating the definitions was carried out internally, without the involvement of external experts.

The second phase of the study focused on collecting country-specific data related to the finalized list of IRMAs (Figure 1). This phase was initiated with a comprehensive targeted literature review. The review employed a search strategy using Medline (via PubMed) for peer-reviewed articles, supplemented by searches in grey literature sources such as Eurostat and Google. The search strategy was designed to capture relevant studies and reports using a combination of specific keywords associated with the list of included IRMAs and the focus countries, which encompass all European countries and Israel. To enhance the integrity of the acquired data, expert survey #3 was implemented, reaching out to ENABLE country representatives (i.e., all European countries and Israel). The goal was to validate and, if necessary, update the data based on the insights received.

### Data collection

Surveys utilized in this research were administered via SurveyMonkey.com (www.surveymonkey.com). Participation was voluntary. Invitations for the first two surveys were sent to ENABLE members (i.e., medication adherence experts from various clinical fields: physicians, pharmacists, psychologists, and nurses), while the third targeted only country representatives of the ENABLE team from all European countries and Israel. Online surveying system settings were set to block multiple entries from the same IP address. No incentives were provided for participation. At the beginning of each survey before providing informed consent, participants were informed about the objectives, data usage and storage, and expected duration. The average completion times for the first, second and third surveys were estimated to be 20, 15, and 45 min, respectively. Each survey was administered in a single session. Expert Survey #1 was conducted from 14th to 31st October 2022, Expert Survey #2 from 14th to 30th November 2022, and Expert Survey #3 from 5th January to 31st May 2023.

This study was conducted under the ENABLE COST Action research program, received favorable approval from the Research Ethics Committee of the Province of Malaga on 29 April 2021. The study collected individual opinions and publicly available information from ENABLE members. Names were collected solely from participants who consented to be acknowledged in the manuscript. All study data were collected and analysed anonymously, and ethical standards were strictly followed to ensure participant privacy and data protection.

### Data analysis

The collected country-specific data on IRMAs were summarized in a descriptive manner, providing detailed insights into each country’s unique context. This approach involved profiling each indicator separately, highlighting the availability of data and specific national circumstances and trends. Although this information effectively illustrates variations across countries within each indicator, direct comparisons between countries were not conducted. This maintains the study’s focus on descriptive analysis rather than on comparative metrics.

## Results

Out of 34 ENABLE members invited for the Expert Survey #1, 17 participants actively contributed, collectively providing 205 indicators, which are detailed in Supplementary Table S1. In the Expert Survey #2, 21 participants (representing a response rate of 62%) ranked these 205 indicators. From these, 25 indicators reached a relevance score above 3.5 (Supplementary Table S2). Additionally, the ‘country population’ indicator was included by the research team, recognizing its essential role in facilitating future comparative analyses across countries. The mean scores for the ranked indicators varied between 2.76 and 4.52, with a median of 3.89. The final set of included indicators encompassed various domains: six indicators pertained to country characteristics, four to social/economic factors, three to therapy-related aspects, three to patient-related factors, one to condition-related aspects, and nine to healthcare system-related factors (Table 1).

**TABLE 1:** Final list and definition of top-ranked country-specific indicators related to medication adherence.

Domain	Indicator	Definition
Country characteristics	IRMA #1–Method of payment	Refers to the process by which patient is reimbursed for healthcare expenses
	IRMA #2–Medication adherence assessed and reported on the national level	Is medication adherence assessed and reported on the national level? (yes/no)
	IRMA #3–Healthcare provider	Refers to the system by which patient can access healthcare services (e.g., public, private)
	IRMA #4–Model of healthcare system financing	Refers to the system of healthcare financing (e.g., taxes, voluntary health insurance, co-payment)
	IRMA #5–Proportion of population aged 65 and over	Proportion of population aged 65 and over
	IRMA #6–Country population	Population size (based on projection for a given year)
Social/economic factors	IRMA #7–Patient co-payment	The amount of money that a patient is required to pay out-of-pocket for medications
	IRMA #8–Percentage of prescriptions dispensed at no cost to patients	Percentage of prescriptions dispensed at no cost to patients at population level
	IRMA #9–Population coverage	Proportion of population that has access to healthcare services (public, private)
	IRMA #10–Availability of doctors’ services for citizens at no payment	Availability of healthcare services without requiring any out-of-pocket payment
Therapy related factors	IRMA #11–Average number of medicines per patient	Average number of medicines per patient
	IRMA #12–Proportion of adults aged 75 years and older who are taking >5 medications concurrently	Proportion of adults aged 75 years and older who are taking >5 medications concurrently
	IRMA #13–Self-reported use of prescribed medicines	Proportion of population using prescribed medication
Patient related factors	IRMA #14–Persons reporting a chronic disease	Proportion of population reporting asthma, COPD, hypertension, diabetes mellitus, chronic depression
	IRMA #15–Self-perceived health	Proportion of population with very good level of self-perceived health
	IRMA #16–Current depressive symptoms	Proportion of population reporting current depressive symptoms
Condition related factors	IRMA #17–General health literacy	Proportion of population with inadequate/problematic/sufficient/excellent general health literacy
Healthcare system related factors	IRMA #18–Percentage of patients receiving adherence interventions	Proportion of patients received interventions designed to improve medication adherence
	IRMA #19–Nationwide availability of e-prescription (Yes/No)	Is e-prescription system nationwide available for patients? (yes/no)
	IRMA #20–Waiting time for prescriptions/medical appointments	Average waiting time for prescriptions and medical appointments
	IRMA #21–Number of practicing physicians	Number of practicing physicians per 100,000 inhabitants
	IRMA #22–Proportion of healthcare expenditure on pharmaceuticals	Proportion of healthcare expenditure on pharmaceuticals
	IRMA #23–Number of practicing pharmacists	Number of practicing pharmacists per 100,000 inhabitants
	IRMA #24–Total healthcare expenditure	Total healthcare expenditure expressed in percentage of GDP
	IRMA #25–Public pharmaceutical expenditure as % of total pharmaceutical expenditure	Public pharmaceutical expenditure expressed in percentage of total pharmaceutical expenditure
	IRMA #26–Self-reported consultations of a medical professional	Distribution of the population according to the number of consultations of a medical doctor in the past 4 weeks

COPD, chronic obstructive pulmonary disease; GDP, gross domestic product; IRMA, indicators related to medication adherence.

**FIGURE 2:**
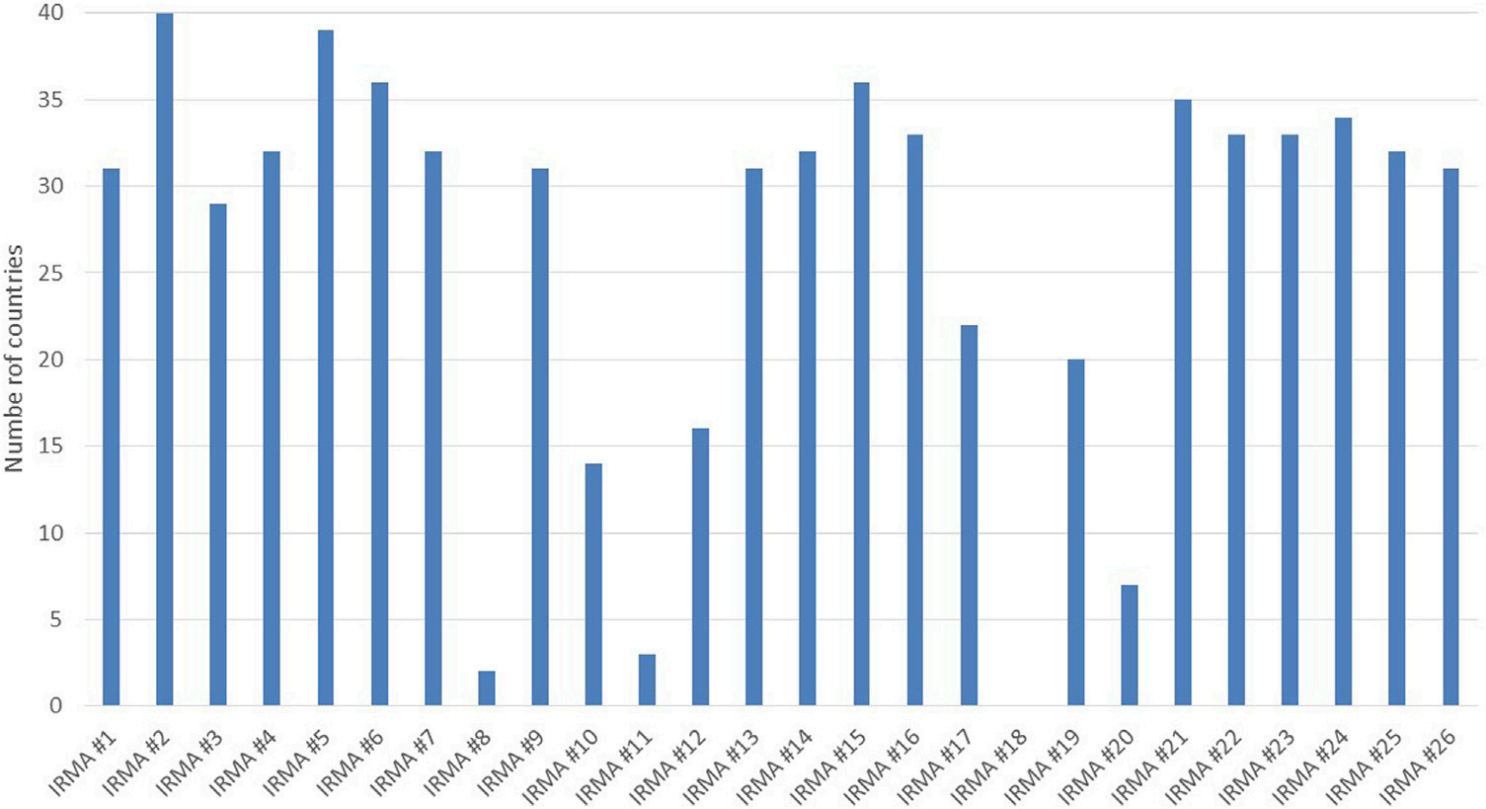
Data availability on indicators related to medication adherence for the studied countries.

Data on the identified key IRMAs were collected for a total of 39 European countries and Israel. The gathered country-specific data were subjected to a validation process by the ENABLE country representatives to ensure its accuracy and reliability. Although efforts were made to validate the data from all participating countries, only 75% successfully completed this process. Unfortunately, we did not receive responses from ENABLE’s representatives in Austria, Belgium, Denmark, Ireland, Israel, Luxembourg, Moldova, the Netherlands, Sweden, and the United Kingdom by the study’s deadline. Detailed country-specific data on key IRMAs can be found in Supplementary Table S3, where countries are listed alphabetically. To maintain transparency and credibility, all data sources are cited alongside the information presented in this table. The availability of data on IRMAs varied among the countries studied, as illustrated in Figure 2.

### Country characteristics

The availability of data for the six indicators under this subgroup was relatively high compared to the other categories (Figure 2). For IRMA #2, which assesses and reports on medication adherence at the national level, information was available for all countries. However, only two countries reported having an established system in place: Croatia, which uses a self-report questionnaire in pharmacies, and Italy, where the method of adherence measurement was not specified during the data validation process. Additionally, the proportion of the population aged 65 and over (IRMA #5) was reported for all countries except one (Israel), ranging from 9.5% in Türkiye to 23.5% in Italy.

### Social/economic factors

Regarding patient co-payment (IRMA #7), data was available for 32 countries. For IRMA #8, which tracks the percentage of prescriptions dispensed at no cost to patients, valid data came exclusively from Serbia (14.8%) and Slovenia (51.9%). For population coverage (IRMA #9), information was accessible from 31 countries, while data on the availability of doctors’ services for citizens without payment (IRMA #10) was reported by 14 countries. The coverage of the population with access to healthcare services was above 90% in all countries with valid data, except for Bulgaria at 85% and Ukraine at 83%.

### Therapy-related aspects

**FIGURE 3:**
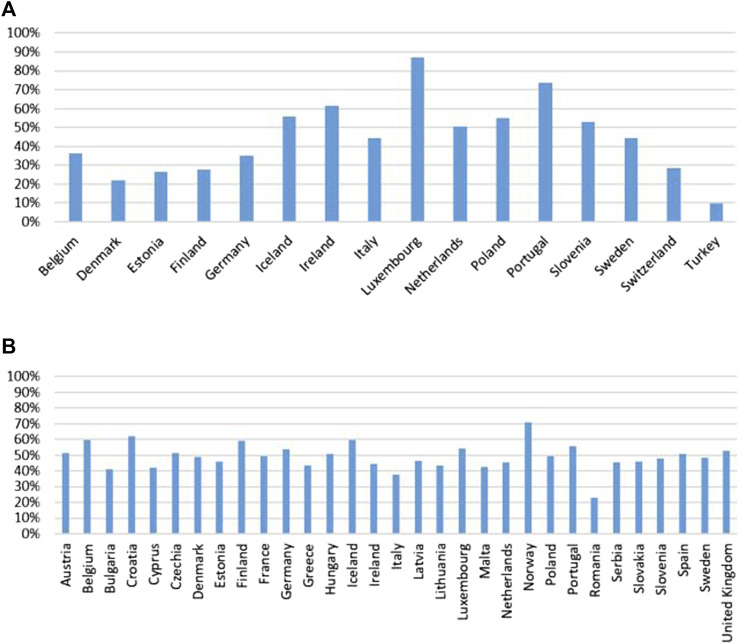
Proportion of adults aged 75 years and older who are taking more than five medications **(A)** and self-reported use of prescribed medicines in the countries with available data **(B)**.

The average number of medications per patient (IRMA #11), data was only accessible from a few countries: Germany reported an average of 4.1 medications per patient, Poland 3.7, and Slovenia 8.9. Considering the proportion of adults aged 75 years or older are taking more than five medications concurrently (IRMA #12), there was a notable variation among the 16 countries with available data. This proportion ranged from a low of 10% in Türkiye to a high of 87% in Luxembourg. Additionally, the self-reported use of prescribed medicines (IRMA #13) had data available for 78% of the countries (n = 31). The reported use of medicines varied widely, from 23% in Romania to 71% in Norway, illustrating the diversity in medication usage patterns among countries. Country-specific data for IMRA #12 (Proportion of adults aged 75 years and older who are taking more than five medications) and IRMA #13 (Self-reported use of prescribed medicines) are presented in Figure 3.

### Patient-related factors

**FIGURE 4:**
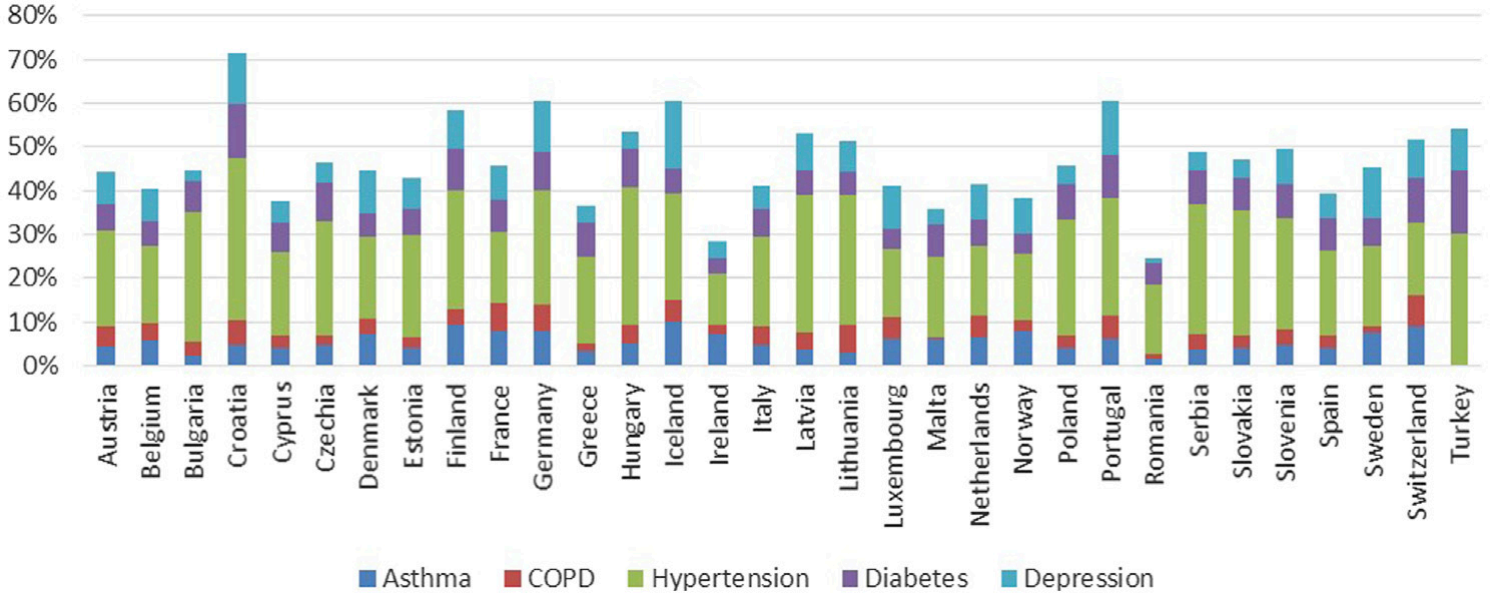
Proportion of population reporting asthma, COPD, hypertension, diabetes mellitus, chronic depression in the countries with available data.

The availability of country-specific data for patient-related IRMAs was at or above 80%. Data on persons reporting a chronic disease (IRMA #14), such as asthma, chronic obstructive pulmonary disease (COPD), hypertension, diabetes, and depression are illustrated in Figure 4. The proportion of the population with a very good level of self-perceived health (IRMA #15 Self-perceived health) varied significantly, ranging from 6.5% in Türkiye to 46.9% in Greece. The proportion of the population reporting current depressive symptoms (IRMA #16) showed less variability, ranging from 2% (Albania) to 10.8% (France).

### Condition-related aspects

**FIGURE 5:**
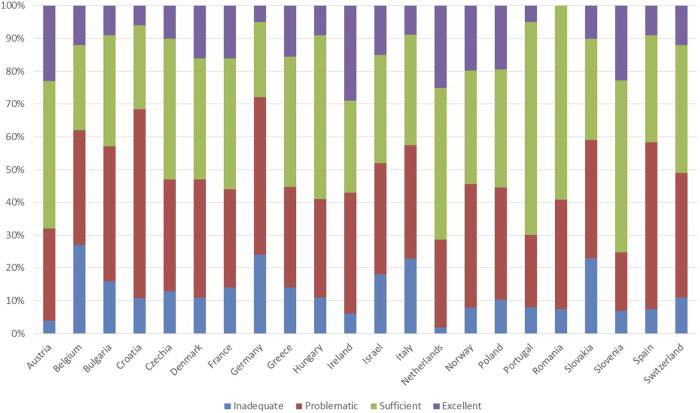
Proportion of the population with varying levels of general health literacy in the countries with available data.

Data on the proportion of the population with varying levels of general health literacy (IRMA # 17)–categorized as inadequate, problematic, sufficient, and excellent—was reported by 55% of the countries studied (n = 22), is presented in Figure 5.

### Healthcare system-related factors

No country provided data for IRMA #18, which concerns the percentage of patients receiving adherence interventions. Regarding IRMA #19, which focuses on the nationwide availability of e-prescription systems, only 50% of the countries provided data, and all reported having some form of e-prescription system in place. Additionally, information on the average waiting time for prescriptions and medical appointments (IRMA #20) was available from just seven countries: Bulgaria, Estonia, Finland, Germany, Italy, Norway, and Poland (data varied across countries, for details see Supplementary Table S3).

**FIGURE 6:**
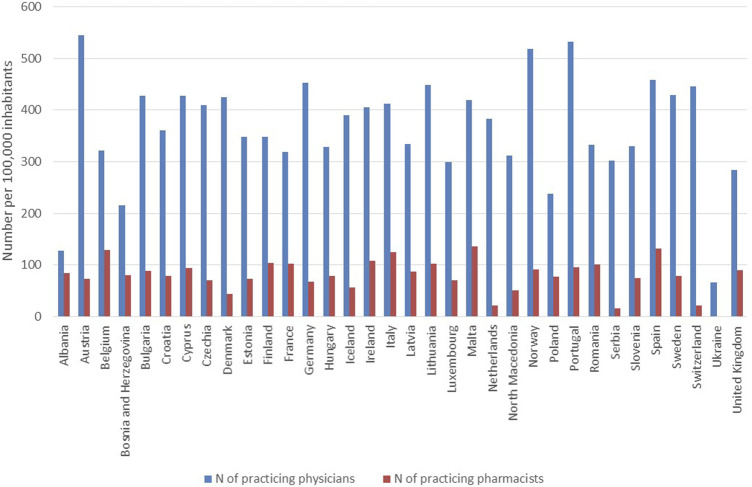
Number of practicing physicians and pharmacists per 100,000 inhabitants in the countries with available data.

**FIGURE 7:**
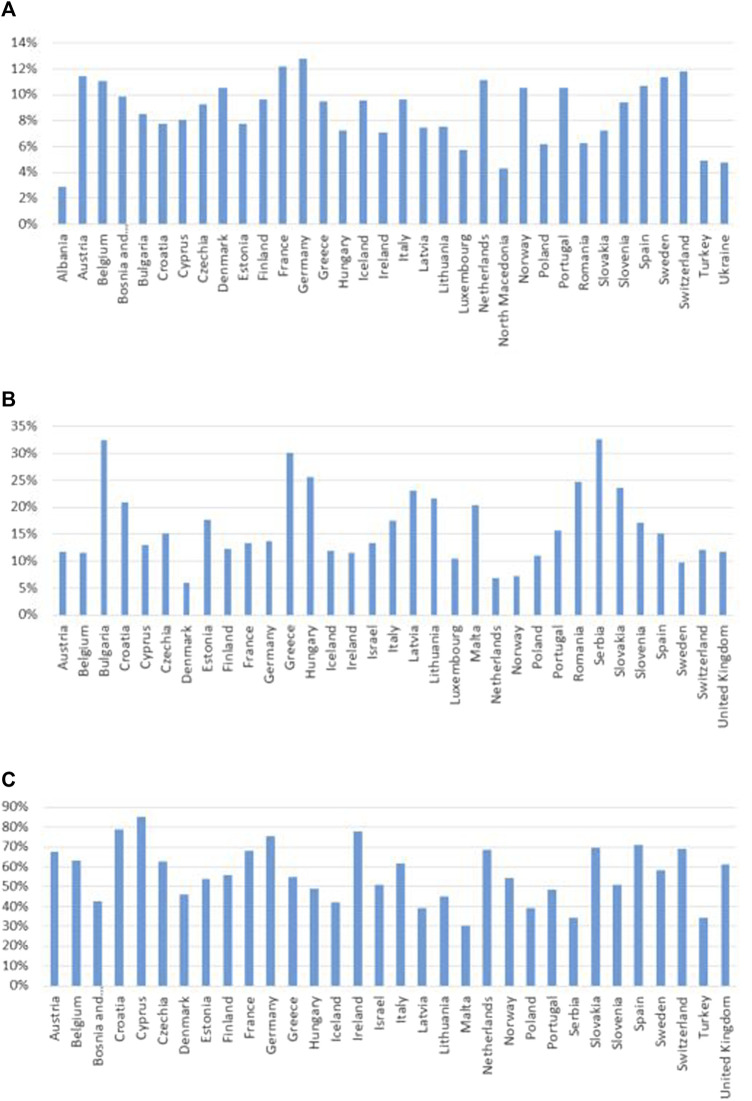
Total healthcare expenditure as a percentage of GDP *per capita* **(A)**, the proportion of healthcare expenditure on pharmaceuticals **(B)**, and public pharmaceutical expenditure as a percentage of total pharmaceutical expenditure in the countries with available data **(C)** GDP, gross domestic product.

**FIGURE 8:**
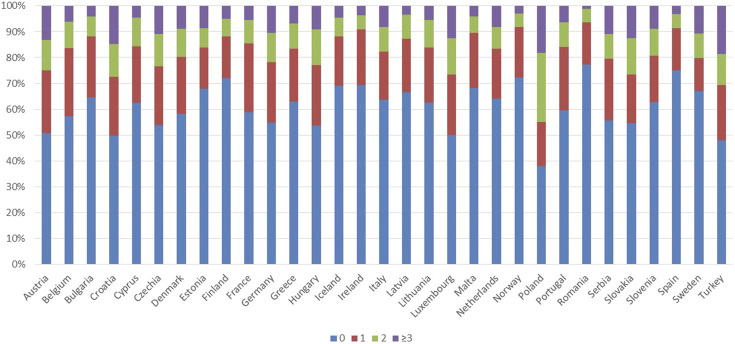
Distribution of the population according to the number of consultations with a medical doctor in the past 4 weeks in the countries with available data.

For the other IRMAs in this subcategory the country-specific data availability was around or above 80%. Country-specific data on the number of practicing physicians (IRMA #21) and pharmacists (IRMA #23) per 100,000 inhabitants are presented in Figure 6. Total healthcare expenditure as a percentage of GDP *per capita* (IRMA #24), the proportion of healthcare expenditure on pharmaceuticals (IRMA #23), and public pharmaceutical expenditure as a percentage of total pharmaceutical expenditure (IRMA #25) are illustrated in Figure 7. Lastly, results on the distribution of the population according to the number of consultations with a medical doctor in the past 4 weeks are depicted in Figure 8.

## Discussion

The identification and collection of data on key IRMAs through a structured expert survey methodology have provided valuable insights into the multifaceted nature of medication adherence. Findings of our study underscore the complexity of indicators that might predict medication adherence across European countries and Israel, highlighting the pivotal role of country-specific IRMAs.

The methodology employed in identifying and ranking key IRMAs through expert surveys within the ENABLE network has successfully highlighted the priority areas as perceived by professionals engaged in this field. The emphasis on healthcare system-related factors, with nine indicators, underscores the systemic challenges inherent in medication adherence. Additionally, the inclusion of indicators across social/economic, therapy-related, patient-related, and condition-related domains recognizes the multifaceted nature of medication adherence. The consensus on certain indicators (e.g., IRMA #1 Method of payment, IRMA #5 Proportion of population aged 65, and over, and IRMA #7 Patient co-payment), reflected by the standard deviation scores (Supplementary Table S2), points to a shared understanding of core indicators of medication adherence within a country. Nevertheless, the variation in relevance scores and the comprehensive range of domains represented by the final indicators highlight the complexity of medication adherence challenges (Mathes et al., 2014; Yeam et al., 2018). These results suggest that effective adherence-enhancing interventions must extend beyond addressing patient and therapy-related factors to include broader socio-economic and healthcare system-related determinants.

The vast variability in the availability of data on key IRMAs across the studied countries highlights the need for further research and more comprehensive primary data collection. Data for certain IRMAs, such as IRMA #8 (Percentage of prescriptions dispensed at no cost to patients), IRMA #11 (Average number of medicines per patient), IRMA #18 (Percentage of patients receiving adherence interventions), and IRMA #20 (Waiting time for prescriptions/medical appointments), were either not available or very limited in a number of countries. Nonetheless, according to expert opinions, these factors may significantly influence the level of medication adherence at the country level and thus warrant closer attention.

Our study revealed that the majority of European countries do not assess medication adherence on a national scale (Indicator: IRMA #2–Medication adherence assessed and reported at the national level). However, the prevalent use of electronic medical record datasets and e-prescription systems across Europe could facilitate the production of these data (Brennan et al., 2015; Ágh et al., 2021). Moreover, the initiative to integrate big data within European nations (European Health Data Space—EHDS) would further enable cross-country comparisons (European Commission, 2024). The EHDS initiative could be instrumental in understanding and improving medication adherence trends both within individual countries and across Europe as a whole. Such data integration could lead to more informed healthcare policies and better patient outcomes.

The absence of country-level medication adherence rates for chronic therapies did not allow for an investigation into the correlation between the identified key IRAMs and medication adherence rates. This is not surprising, as a recent OECD report demonstrates a majority of the European countries are neither monitoring adherence nor taking regular actions to improve it (Khan R and Socha-Dietrich, 2018). Despite this limitation, the country-level data collected in this study emphasize the variability of these indicators across countries, pointing out the importance of contextual factors such as healthcare infrastructure, patient education, and access to medications. Strategies to improve medication adherence should be tailored to address the specific barriers and opportunities within each country’s unique healthcare ecosystem (Riley et al., 2021). In this context, it is crucial to recognize that adherence-enhancing interventions effective in one country are not guaranteed to work in another. Transferability analysis can help to identify key factors of variability and formulate implementation strategies for the application of interventions across different jurisdictions, ensuring that strategies are both effective and adaptable to local contexts.

The potential effect on country-level adherence could be multifaceted. Countries with a higher incidence of polypharmacy among their elderly populations might face challenges with adherence due to the complexities of managing multiple medications simultaneously, as depicted in Figure 3 (Sinha et al., 2021; Franchi et al., 2022). These complexities can lead to increased risks of adverse drug reactions, poor adherence and decreased effectiveness of treatment regimens (de Vries et al., 2014; Zazzara et al., 2021). Conversely, high rates of prescription usage across a population could indicate robust healthcare systems, suggesting potentially higher adherence rates due to better access to medications and more streamlined healthcare processes. However, a strong prescription culture does not inherently assure superior health outcomes without effective medication management and patient education. The varied percentages of populations using prescribed medications, ranging from 23% in Romania to 71% in Norway, suggest differing healthcare service utilizations that could also impact adherence levels. Furthermore, the proportion of the population reporting chronic conditions such as asthma, COPD, hypertension, diabetes mellitus, and chronic depression varies significantly across countries (Figure 4), which could influence both the extent of polypharmacy and the complexity of medication regimes. Additionally, the distribution of health literacy levels, from inadequate to excellent, varies widely between countries (Figure 5) (Sørensen et al., 2015). Higher levels of health literacy are typically associated with a better understanding and management of one’s health conditions, which facilitates medication adherence (Arad et al., 2021; Hyvert et al., 2023). In contrast, lower levels of health literacy can lead to misunderstandings about medication usage, resulting in lower adherence and poorer health outcomes (Miller, 2016).

Healthcare system-related factors might also be critical in influencing medication adherence. A higher *per capita* number of practicing physicians and pharmacists can facilitate more consistent and personalized patient care, especially among populations with a high prevalence of chronic conditions (Schneider et al., 2020). Better access to healthcare providers enhances the monitoring and adjustment of medications, potentially improving adherence rates (Figure 6) (Kini and Ho, 2018). Healthcare spending (Figure 7) may also effect in medication adherence. Investments in healthcare, particularly those earmarked for pharmaceuticals, can improve access to healthcare services and medications. Adequate funding may allow for the implementation of comprehensive medication management programs and patient education, which can improve adherence by ensuring that patients understand their treatment plans and the importance of following them (Viswanathan et al., 2012). The frequency of medical consultations (Figure 8) further underscores the importance of healthcare access. Regular contact with healthcare providers is a key factor in adherence, as it enables ongoing health education, timely identification of side effects, and medication non-adherence, as well as implementation of adequate interventions. Countries where populations have fewer medical consultations may need to enhance healthcare accessibility and encourage regular provider-patient interactions to support adherence.

Our study’s findings have significant implications for policymakers, healthcare providers, and researchers. By identifying key country-specific IRMAs, our study provides a foundation for the formulation of targeted health policies and medication adherence enhancing interventions. These adherence interventions should aim to address the unique challenges and leverage the specific strengths of each country’s healthcare system and patient population. Medication adherence is a measure of quality and effectiveness of the entire healthcare system (Khan R and Socha-Dietrich, 2018). Therefore, despite its crucial role in strengthening the sustainability of the national healthcare systems, it may also serve as a valid indicator of their effectiveness, allowing for fast and objective benchmarking. This is of utmost importance for unfavorable conditions as those set by recent COVID-19 pandemic, and current economic crisis (Ágh et al., 2021; Ágh et al., 2023). Moreover, our study highlights the need to further refine and validate the identified IRMAs, thereby enhancing the effectiveness of medication adherence interventions.

Our results must be considered in light of certain limitations. The potential for bias in participants’ responses is notable, as the majority of respondents had an academic background and may not have had comprehensive access to or familiarity with various data sources, affecting the adequacy of data validation. Moreover, the data validation process was carried out by only one representative per country, potentially diminishing its robustness. These factors suggest that the study’s conclusions must be interpreted with caution, as the integrity of data and subsequent analyses may not fully capture the complex and varied landscape of medication adherence across different countries. Additionally, while the expertise of the selected professional panel provided valuable insights into medication adherence, the exclusion of patient representatives from the panel may limit the diversity of perspectives considered. Future studies could benefit from incorporating patient viewpoints to enhance the comprehensiveness and applicability of the findings.

In conclusion, the iterative approach employed in this study successfully facilitated the identification of key country-specific IRMAs, providing a valuable resource for policymakers and stakeholders to deepen their understanding of medication adherence across European countries and Israel. The cohesive list of indicators not only promotes fair benchmarking among countries but also serves as a foundation for future studies aiming to assess the predictive value of these indicators in determining medication adherence rates within a given country. Our findings highlight the importance of targeted, country-specific interventions and the potential of technological advancements in improving medication adherence. Further research is needed to rank these indicators accordingly and better comprehend their impact.

## Data Availability

The original contributions presented in the study are included in the article/Supplementary Material, further inquiries can be directed to the corresponding author.

## European Network to Advance Best Practices and Technology on Medication Adherence (ENABLE) Collaborators Participating in the study

Emma Aarnio, Darinka Gorgieva Ackova, Vesna Vujic Aleksic, Martina Bago, Juris Barzdins, Manon Belhassen, Katharina Blankart, Maria A. Cordina, Josip Culig, Erdősi Dalma, Cristina Ghiciuc, Francisca Leiva Fernández, Pilar Barnestein Fonseca, Freyja Jonsdottir, Fatjona Kamberi, Barbora Kostalova, Urška Nabergoj Makovec, Valentina Marinković, Enrica Menditto, Vildan Mevsim, Zornista Mitkova, Herbolka Natalka, Christos Petrou, Panagiotis Petrou, Guenka Petrova, Mitar Popovic, Katarina Smilkov, Ioanna Tsiligianni, Marie Paule Schneider Voirol, Daisy Volmer, Martin Wawruch.
