# Supplementary material for: Identifying and presenting key country-specific indicators related to medication adherence: a comprehensive study across European countries
Source: Front Pharmacol. 2024 Oct 4;15:1390629. doi: 10.3389/fphar.2024.1390629 (PMC11487323; doi:10.3389/fphar.2024.1390629)
Supplement: Supplementary file 1 [file DataSheet1.PDF]

# Identifying and presenting key country-specific indicators related to medication adherence: A comprehensive study across European countries

## Supplementary Materials

Table of Contents

Supplementary Table S1. List of identified potential country-specific IRMAs ..... 4
Supplementary Table S2. Results of the ranking of the identified country-specific IRMAs ..... 9
Supplementary Figure S1. Country-specific data on key IRMAs ..... 11
ALBANIA ..... 11
AUSTRIA ..... 17
BELGIUM ..... 23
BOSNIA AND HERZEGOVINA ..... 29
BULGARIA ..... 35
CROATIA ..... 41
CYPRUS ..... 47
CZECHIA ..... 53
DENMARK ..... 59
ESTONIA ..... 65
FINLAND ..... 71
FRANCE ..... 77
GERMANY ..... 83
GREECE ..... 89
HUNGARY ..... 95
ICELAND ..... 101
IRELAND ..... 107
ISRAEL ..... 113
ITALY ..... 119
LATVIA ..... 125
LITHUANIA ..... 131
LUXEMBOURG ..... 137
MALTA ..... 143
MOLDOVA ..... 149
MONTENEGRO ..... 155
THE NETHERLANDS ..... 161
NORTH MACEDONIA ..... 167
NORWAY ..... 173
POLAND ..... 179
PORTUGAL ..... 185

ROMANIA .......... 191
SERBIA .......... 198
SLOVAKIA .......... 204
SLOVENIA .......... 210
SPAIN .......... 217
SWEDEN .......... 223
SWITZERLAND .......... 229
TURKEY .......... 235
UKRAINE .......... 241
UNITED KINGDOM .......... 248

Supplementary Table S1. List of identified potential country-specific IRMAs

| Domain | Potential indicators |
|---|---|
| Social/economic factors | Access to affordable medications<br>Access to care<br>Aging population<br>Availability of doctors' services for citizens at no payment<br>Average cost of medication to patient<br>Average level of patient co-payment for drugs in outpatient settings<br>Beliefs about medications<br>Cost of therapy<br>Dependency rate of the elderly<br>Education<br>Employment<br>High cost of healthcare services (including medicines)<br>Income<br>Income of patient<br>Knowledge of medication/disease<br>Number of medications in different reimbursement categories<br>People entitled to special reimbursement<br>Percentage of generics among drugs available for cost to patients<br>Percentage of patients on a medication subsidy programmes<br>Percentage of patients who have access to no cost drugs<br>Percentage of patients with low socio-economical status that are non-adherent<br>Percentage of prescriptions dispensed at no cost to patients<br>Percentage of products per ATC category which are reimbursed<br>Percentage of products with low contribution<br>Percentage of products with no personal contribution<br>Percentage of Rx drugs available at no cost to patients<br>Poverty rate<br>Proportion of people without completed high school (literacy level)<br>Race, gender, age, educational level etc<br>Reimbursement coverage policy<br>Reimbursement of Rx drugs in place<br>Social support<br>Social welfare services being accessible to persons in need<br>The percentage of out-of-pocket payments from total health expenditure<br>The share of inhabitants speaking the national language |
| Therapy related factors | Ability to contact physicians in cases of queries/questions<br>Access to personalised care, eg comorbidities influence adherence<br>Adherence to treatment influenced by comorbidities, number of medications, side effects etc<br>Availability of additional services (e.g. lab tests) for citizens at no payment<br>complexity of the regimen/therapy (pill burden)<br>Conscious simplification of medication regimens by prescribers<br>Cost<br>Evaluation (%) of medicine administration (e.g. Asthma inhalers)<br>Extent of proper briefing of patients regarding the proper use<br>Extent of shared decision making, prior to the prescription of each product<br>Form of administration (e.g. pain associated with administration, taste, frequency) |

| | |
|---|---|
| | Health literacy<br>Health workers inform patients about possible drug interactions and ADRs<br>Implementation of services and activities to support polypharmacy patients<br>Interference with a person's lifestyle<br>Lifelong therapy<br>Long and complicated therapy<br>Long term treatment<br>Medication possession ratio (MPR)<br>(Number of) adverse events<br>Number of medicines per patient<br>Number of patients on 5 or more regular medication<br>Percentage of patient satisfied with treatment<br>Percentage of patients on once daily regimes<br>Percentage of patients taking alternative dosage forms (eg. combination therapy/ slow release)<br>Percentage of patients with complex therapy regimee using medication adherence interventions<br>Polipharmacotherapy<br>Proportion of days covered (PDC)<br>Quantity of medicines prescribed for one patient<br>Requiring frequent specialists visits<br>Services reminding the patient a need to renew the prescription on time are available<br>Time which the patient have to wait to receive an appointment (consultation) to the doctor<br>Too many medicines and procedures |
| Patient related factors | (Level of) health literacy<br>Age<br>Beliefs, misconceptions, perceptions<br>Distance to pharmacy (km)<br>Education<br>Income level<br>Knowledge<br>Lack of confidence in healthcare professionals<br>Lack of knowledge and education considering the results of low level of adherence<br>Low motivation as a results of weak consultation<br>Memorizing capabilities<br>Mental health<br>Multiple sources of reliable information available for the patient<br>Neurodivergence (e.g. ADHD, low IQ)<br>Overall days used for treatment of condition (medications used consecutively)<br>Patient satisfaction with therapy<br>Patient's beliefs, preferences and needs are taken into consideration when prescribing<br>Percentage of patient with cognitive deficiency that are non-adherent<br>Percentage of patients using adherence aids<br>Percentage of patients with multimorbidity that are non-adherent<br>Percentage of patients with regular medication routine<br>Percentage of prescriptions not filled/poorly filled per time frame |

| | |
|---|---|
| | Quantity of doses used incorrect (before/after meal, etc.)<br>Quantity of drug side affects<br>Quantity of missed appointments or doses<br>Socio-economic factors<br>Visual capabilities |
| Condition related factors | (Overuse) estimated use vs population (ex. OTC-painkillers) per time frame<br>Acute<br>Breakdown of conditions by severity of illness<br>Care according to guidelines that improve adherence<br>Chronic<br>Clinical outcomes<br>Complexity<br>Complicated therapy<br>Dissemination to health professionals of the proper therapeutic doses<br>Drugs for certain specific conditions (e.g. psychiatric disorders) available at no cost<br>Frequent hospitalisations<br>Health specific literacy<br>Health workers and psychologists support adherence in patients with mental and behavioral disorders<br>Health workers educate patients on their diseases<br>Health workers focus on adherence counselling in case of asymptomatic and chronic diseases<br>Health-related quality of life<br>High cost of adverse event treatment<br>Low level of education considering manifestation of adverse reactions<br>Mortality<br>Percentage of patients taking long-term therapies that are non-adherent<br>Percentage of patients taking prophylactic drugs that are non-adherent<br>Percentage of short versus long term conditions<br>Polymorbidity<br>Proportion of people with their conditions controlled according to professional societies guidelines<br>Psychiatric diseases<br>Quality of care<br>Severe diseases<br>Treatment guidelines (medication adherence in these) |
| Healthcare system related factors | Percentage of drug-related hospital admissions per time frame<br>Percentage of population with universal coverage<br>Access<br>Access to care<br>Access to reimbursement<br>Access to relevant information regarding medicines<br>Access to specialised care<br>Access to treatment<br>Availability of e-prescription<br>Availability of follow-up system (indicators of non-adherence)<br>Availability of healthcare services<br>Availability of medicines<br>Capacity of HCPs (time per patient)<br>Continuity of care |

| | |
|---|---|
| | Cost<br>Digital dispensing records are available and assessed for non-adherence by health workers<br>Frequency of visits<br>Health care digitalization status<br>Health care financing status<br>Health insurance policy<br>Health workers are trained to provide comprehensive counselling on medications<br>Health workers are trained to provide person-centered care<br>Healthcare costs per capita<br>Implementation of clinical audits<br>Implementation of clinical tools for adherence, behavioral communication skills<br>Implementation of interprofessional education and collaboration<br>Insurance<br>Knowledge of HCPs on medication adherence, patient experience and alternative treatments/solutions<br>Lack of capacity for patients monitoring and consultation<br>Lack of effective feedback<br>Level of implementation outcomes-based approach<br>Medication availability (number of medication shortages)<br>National implementation and remuneration of activities to improve adherence<br>Number of adverse events / hospitalisation due to non-adherence<br>Number of GP/outpatient/inpatient/ER visits per year per capita<br>Number of pharmacies<br>Number of physicians/pharmacists<br>Nursers leadership<br>Patient's out-of-pocket costs/reimbursement<br>Percentage of healthcare workers receiving training on adherence management<br>Percentage of patients on subsidised or free medication<br>Percentage of patients receiving adherence interventions<br>Percentage of patients with follow-up on adherence at every contact with health care provider<br>Percentage of Rx drugs available at no cost to patients<br>Pharmacist leadership<br>Pharmacists' knowledge<br>Physicians' knowledge<br>Proportion of health care budget allocated to reimbursement<br>Proportion of health care services reimbursed<br>Proportion of medicines covered by reimbursement<br>Psychological support for patients available<br>Quality of care<br>Quality systems development<br>Quantity of drugs not existing in pharmacy due to legislation restrictions<br>Ratio of pharmacist/patients<br>Ratio of pharmacists/habitant<br>Relationship between HCPs and the patient<br>Satisfaction with care |

| | |
|---|---|
| | Share of people covered by the national health insurance<br>Short time for consultation (including doctors and pharmacist)<br>Support<br>Timely response by healthcare professionals<br>Unlimited access to primary care<br>Waiting time for prescriptions / medical appointments<br>Waiting time for therapy<br>Wide availability of e-prescription system (e.g. at primary care level) |
| Other | Average patient health literacy<br>Clinical audits that improve adherence in place<br>Discussing health issues and treatment options by healthcare system<br>Health politicians understand the importance of medicines adherence<br>Healthcare systems policies<br>Lack of digital platform or devices (reimbursed) for support and monitoring of adherence<br>Lack of time for consulting<br>Medicines adherence in pharmaceutical policy<br>Missing social support to patients with chronic diseases (especially those needed social worker)<br>Necessity of frequent days out of work and fear of work lost<br>Number of adherence interventions delivered per patient per annum<br>Patients’ values and beliefs<br>Patients are still working and busy<br>Percentage of health professionals trained in adherence education or behaviour change<br>The number of peer-support groups for chronic conditions<br>Tools to measure adherence |

Supplementary Table S2. Results of the ranking of the identified country-specific IRMAs

| Domain | Indicator | Number of valid answers | Mean score | SD score |
|---|---|---|---|---|
| Country characteristics | Drug reimbursement model | 21 | 4.38 | 0.67 |
| | Medication adherence assessed and reported on the national level: y/n | 21 | 4.38 | 1.02 |
| | Healthcare provider & coverage | 21 | 4.24 | 0.83 |
| | Model of healthcare system financing | 21 | 4.24 | 0.89 |
| | Proportion of population aged 65 and over | 21 | 3.71 | 0.78 |
| | Population by educational attainment level | 21 | 3.43 | 1.08 |
| | Rates of preventable and treatable causes of death | 21 | 3.29 | 1.01 |
| | GDP per capita in EUR | 21 | 3.24 | 1.14 |
| | Country population | 20 | 2.90 | 1.55 |
| | Population by family status | 21 | 2.76 | 1.00 |
| Social/economic factors | Average level of patient co-payment for drugs in outpatient settings | 21 | 4.48 | 0.68 |
| | Percentage of prescriptions dispensed at no cost to patients | 21 | 4.33 | 0.97 |
| | Percentage of population with universal coverage | 21 | 4.24 | 1.00 |
| | Availability of doctors' services for citizens at no payment | 21 | 4.14 | 1.01 |
| Therapy-related factors | Average number of medicines per patient | 21 | 4.38 | 0.92 |
| | Percentage of patients on 5 or more regular medication | 21 | 4.33 | 1.11 |
| | Self-reported use of prescribed medicines | 21 | 3.86 | 1.15 |
| Patient-related factors | % of citizens with chronic conditions | 21 | 4.05 | 1.28 |
| | Self-perceived health | 21 | 3.86 | 0.91 |
| | Current depressive symptoms | 21 | 3.76 | 1.09 |
| | Life-expectancy at birth | 21 | 2.90 | 1.18 |
| Condition-related factors | Health specific literacy | 21 | 4.43 | 1.08 |
| Healthcare system-related factors | Percentage of patients receiving adherence interventions | 21 | 4.52 | 0.98 |
| | Nationwide availability of e-prescription: Yes/No | 21 | 4.14 | 1.06 |
| | Waiting time for prescriptions / medical appointments | 21 | 4.05 | 1.02 |
| | Number of practicing physicians per 1000 population | 21 | 3.95 | 1.07 |

| | | | | |
|---|---|---|---|---|
| | Proportion of health care expenditure on pharmaceuticals and medical devices | 21 | 3.90 | 1.18 |
| | Number of practicing pharmacists per 1000 population | 21 | 3.86 | 1.06 |
| | Health care expenditure and share of GDP | 21 | 3.76 | 1.26 |
| | Public spending as a proportion of pharmaceutical spending | 21 | 3.62 | 1.32 |
| | Self-reported yearly number of consultations of a medical professional | 21 | 3.57 | 1.12 |

Supplementary Table S3. Country-specific data on key IRMAs

ALBANIA

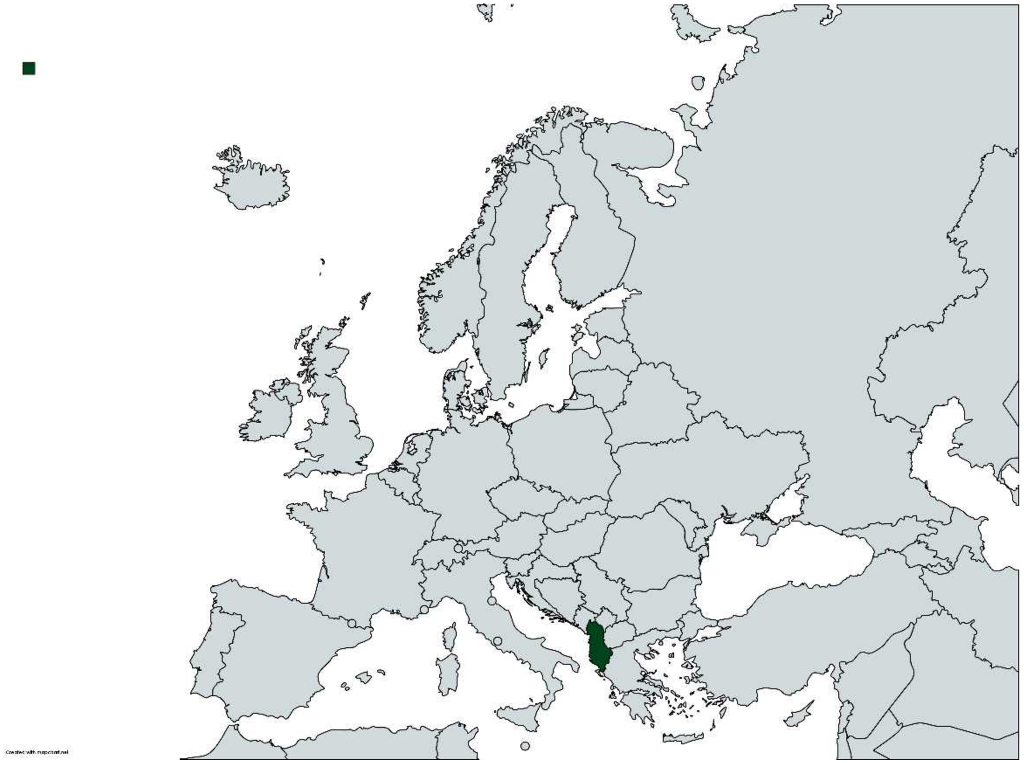

| Country | Albania |
|---|---|

Country-specific data

| Country characteristics | Method of payment | |
|---|---|---|
| | Data | NA |
| | Time | NA |
| | Reference | NA |
| | | |
| | Medication adherence assessed and reported on the national level | |
| | Data | No |
| | Time | 2022 |
| | Reference | Expert opinion |
| | | |
| | Health care provider | |
| | Data | NA |
| | Time | NA |
| | Reference | NA |
| | | |
| | Model of healthcare system financing | |
| | Data | Public |
| | Time | 2023 |
| | Reference | https://shendetesia.gov.al/organizimi-i-sistemit-shendetesor/ |
| | | |
| | Proportion of population aged 65 years and over | |
| | Data - % of persons | 15.2 |
| | Time | 2021 |
| | Reference | https://ec.europa.eu/eurostat/databrowser/view/TPS00028/default/table?lang=en&category=demo.demo_ind / |
| | | |
| | Country population (projection) | |
| | Data - N of persons | NA |
| | Time | NA |
| | Reference | NA |

| | | |
|---|---|---|
| Social/economic factors | **Patient co-payment** | |
| | Data | NA |
| | Time | NA |
| | Reference | NA |
| | | |
| | **Percentage of prescriptions dispensed at no cost to patients** | |
| | Data - % of prescriptions | NA |
| | Time | NA |
| | Reference | NA |
| | | |
| | **Population coverage** | |
| | Data | NA |
| | Time | NA |
| | Reference | NA |
| | | |
| | **Availability of doctors' services for citizens at no payment** | |
| | Data | NA |
| | Time | NA |
| | Reference | NA |
| | | |
| Therapy-related factors | **Average number of medicines per patient** | |
| | Data - N of medicines per patient | NA |
| | Time | NA |
| | Reference | NA |
| | | |
| | **Proportion of 75 years and over who are taking more than 5 medications concurrently** | |
| | Data - % of persons | NA |
| | Time | NA |
| | Reference | NA |
| | | |
| | **Percentage of self-reported use of prescribed medicines** | |
| | Data - % of persons | NA |

| | | |
|---|---|---|
| | Time | NA |
| | Reference | NA |

| | | |
|---|---|---|
| Patient-related factors | **Percentage of persons reporting a chronic disease** | |
| | Data - Asthma, % of persons | NA |
| | Data - Chronic lower respiratory diseases, % of persons | NA |
| | Data - High blood pressure, % of persons | NA |
| | Data - Diabetes, % of persons | NA |
| | Data - Chronic depression, % of persons | NA |
| | Time | NA |
| | Reference | NA |
| | | |
| | **Percentage of self-perceived health - very good (16 years and over)** | |
| | Data - % of persons | 54 |
| | Time | 2020 |
| | Reference | https://ec.europa.eu/eurostat/databrowser/view/HLTH_SILC_02/default/table?lang=en&category=hlth.hlth_state.hlth_sph/ |
| | | |
| | **Percenatge of persons with current depressive symptoms** | |
| | Data - % of persons | 2 |
| | Time | 2016 |
| | Reference | https://www.ishp.gov.al/fakte-mbi-perhapjen-e-depresionit-ne-popullate-dhe-stigmen-rreth-tij/ |

| | | |
|---|---|---|
| Condition-related factor | **General health literacy** | |
| | Data - Inadequate health literacy, % of persons | NA |
| | Data - Problematic health literacy, % of persons | NA |
| | Data - Sufficient health literacy, % of persons | NA |
| | Data - Excellent health literacy, % of persons | NA |
| | Time | NA |
| | Reference | NA |

| | | |
|---|---|---|
| Healthcare system-relat | **Percentage of patients receiving adherence interventions** | |
| | Data - % of persons | NA |
| | Time | NA |

| | |
|---|---|
| Reference | NA |
| | |
| **Nationwide availability of e-prescription** | |
| Data | Yes (primary care) |
| Time | 2023 |
| Reference | https://akshi.gov.al/wp-content/uploads/2022/06/vendim-2022-06-01-370.pdf |
| | |
| **Waiting time for prescriptions / medical appointments** | |
| Data | NA |
| Time | NA |
| Reference | NA |
| | |
| **Number of practising physicians per 100,000 inhabitants** | |
| Data - N of practisisng physicians per 100,000 inhabitants | 128.11 |
| Time | 2013 |
| Reference | https://ec.europa.eu/eurostat/databrowser/view/TPS00044/default/table?lang=en&category=hlth.hlth_care.hlth_res.hlth_staff%20%2F |
| | |
| **Proportion of health care expenditure on pharmaceuticals** | |
| Data - % of health care expenditure | NA |
| Time | NA |
| Reference | NA |
| | |
| **Number of practising pharmacists per 100,000 inhabitants** | |
| Data - N of practisisng pharmacists per 100,000 inhabitants | 84.32 |
| Time | 2013 |
| Reference | https://ec.europa.eu/eurostat/databrowser/view/HLTH_RS_PRS1__custom_4104351/default/table?lang=en |
| | |
| **Total health care expenditure as percentage of GDP** | |
| Data - % of GDP | 2.9 |
| Time | 2021 |
| Reference | https://financa.gov.al/wp-content/uploads/2018/08/Dokumenti-i-PBA-Pjesa-1.docx |
| | |
| **Public pharmaceutical expenditure as percentage of total pharmaceutical expenditure** | |

| | | |
|---|---|---|
| | Data - % of total pharmaceutical expenditure | NA |
| | Time | NA |
| | Reference | NA |
| | | |
| | **Self-reported consultations of a medical doctor*** | |
| | Data - No contact, % of population according to the number of consultations of a medical doctor in the past 4 weeks | NA |
| | Data - 1 contact, % of population according to the number of consultations of a medical doctor in the past 4 weeks | NA |
| | Data - 2 contacts, % of population according to the number of consultations of a medical doctor in the past 4 weeks | NA |
| | Data - 3 or more contacts, % of population according to the number of consultations of a medical doctor in the past 4 weeks | NA |
| | Time | NA |
| | Reference | NA |

*Medical doctors include generalist medical practitioners and specialist medical practitioners

AUSTRIA

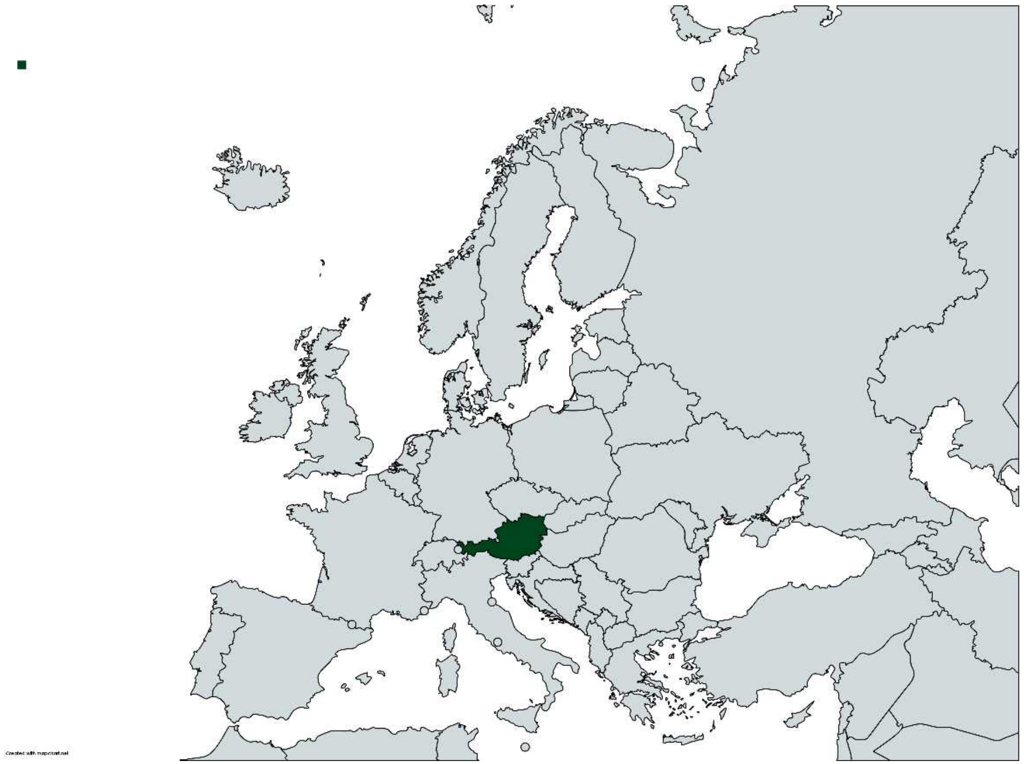

| Country | Austria |
|---|---|

| | | Country-specific data |
|---|---|---|
| Country characteristics | **Method of payment** | |
| | Data | NA |
| | Time | NA |
| | Reference | NA |
| | | |
| | **Medication adherence assessed and reported on the national level** | |
| | Data | No |
| | Time | 2022 |
| | Reference | NA |
| | | |
| | **Health care provider** | |
| | Data | NA |
| | Time | NA |
| | Reference | NA |
| | | |
| | **Model of healthcare system financing** | |
| | Data | NA |
| | Time | NA |
| | Reference | NA |
| | | |
| | **Proportion of population aged 65 years and over** | |
| | Data - % of persons | 19.2 |
| | Time | 2021 |
| | Reference | https://ec.europa.eu/eurostat/databrowser/view/TPS00028/default/table?lang=en&category=demo.demo_ind / |
| | | |
| | **Country population (projection)** | |
| | Data - N of persons | 8904262 |
| | Time | 2020 |
| | Reference | https://ec.europa.eu/eurostat/databrowser/view/CENS_HNMGA/default/table?lang=en&category=cens.cens_hn.cens_hnstr |

| | | |
|---|---|---|
| Social/economic factors | **Patient co-payment** | |
| | Data | NA |
| | Time | NA |
| | Reference | NA |
| | | |
| | **Percentage of prescriptions dispensed at no cost to patients** | |
| | Data - % of prescriptions | NA |
| | Time | NA |
| | Reference | NA |
| | | |
| | **Population coverage** | |
| | Data | NA |
| | Time | NA |
| | Reference | NA |
| | | |
| | **Availability of doctors' services for citizens at no payment** | |
| | Data | NA |
| | Time | NA |
| | Reference | NA |
| | | |
| Therapy-related factors | **Average number of medicines per patient** | |
| | Data - N of medicines per patient | NA |
| | Time | NA |
| | Reference | NA |
| | | |
| | **Proportion of 75 years and over who are taking more than 5 medications concurrently** | |
| | Data - % of persons | NA |
| | Time | NA |
| | Reference | NA |
| | | |
| | **Percentage of self-reported use of prescribed medicines** | |
| | Data - % of persons | 51.1 |

| | | |
|---|---|---|
| | Time | 2019 |
| | Reference | https://ec.europa.eu/eurostat/databrowser/view/HLTH_EHIS_MD1E__custom_3764895/default/table?lang=en / |

| | | |
|---|---|---|
| **Patient-related factors** | **Percentage of persons reporting a chronic disease** | |
| | Data - Asthma, % of persons | 4.3 |
| | Data - Chronic lower respiratory diseases, % of persons | 4.6 |
| | Data - High blood pressure, % of persons | 21.8 |
| | Data - Diabetes, % of persons | 6 |
| | Data - Chronic depression, % of persons | 7.5 |
| | Time | 2019 |
| | Reference | https://ec.europa.eu/eurostat/databrowser/view/HLTH_EHIS_CD1E/default/table?lang=en&category=hlth.hlth_state.hlth_srcm / |
| | | |
| | **Percentage of self-perceived health - very good (16 years and over)** | |
| | Data - % of persons | 33.4 |
| | Time | 2021 |
| | Reference | https://ec.europa.eu/eurostat/databrowser/view/HLTH_SILC_02/default/table?lang=en&category=hlth.hlth_state.hlth_sph/ |
| | | |
| | **Percenatge of persons with current depressive symptoms** | |
| | Data - % of persons | 5.6 |
| | Time | 2019 |
| | Reference | https://ec.europa.eu/eurostat/databrowser/view/HLTH_EHIS_MH1E/default/table?lang=en&category=hlth.hlth_state.hlth_sph / |

| | | |
|---|---|---|
| **Condition-related factor** | **General health literacy** | |
| | Data - Inadequate health literacy, % of persons | 4 |
| | Data - Problematic health literacy, % of persons | 28 |
| | Data - Sufficient health literacy, % of persons | 45 |
| | Data - Excellent health literacy, % of persons | 23 |
| | Time | 2021 |
| | Reference | https://m-pohl.net/Int_Report_methdology_results_recommendations |

| | | |
|---|---|---|
| **Healthcare system-relat** | **Percentage of patients receiving adherence interventions** | |
| | Data - % of persons | NA |
| | Time | NA |

| | |
|---|---|
| Reference | NA |
| | |
| **Nationwide availability of e-prescription** | |
| Data | NA |
| Time | NA |
| Reference | NA |
| | |
| **Waiting time for prescriptions / medical appointments** | |
| Data | NA |
| Time | NA |
| Reference | NA |
| | |
| **Number of practising physicians per 100,000 inhabitants** | |
| Data - N of practisisng physicians per 100,000 inhabitants | 545.25 |
| Time | 2021 |
| Reference | https://ec.europa.eu/eurostat/databrowser/view/TPS00044/default/table?lang=en&category=hlth.hlth_care.hlth_res.hlth_staff%20%2F |
| | |
| **Proportion of health care expenditure on pharmaceuticals** | |
| Data - % of health care expenditure | 11.693 |
| Time | 2020 |
| Reference | https://data.oecd.org/healthres/pharmaceutical-spending.htm |
| | |
| **Number of practising pharmacists per 100,000 inhabitants** | |
| Data - N of practisisng pharmacists per 100,000 inhabitants | 73.08 |
| Time | 2020 |
| Reference | https://ec.europa.eu/eurostat/databrowser/view/HLTH_RS_PRS1__custom_4104351/default/table?lang=en |
| | |
| **Total health care expenditure as percentage of GDP** | |
| Data - % of GDP | 11.47 |
| Time | 2020 |
| Reference | https://ec.europa.eu/eurostat/databrowser/view/TPS00207/default/table?lang=en&category=hlth.hlth_care.hlth_sha11.hlth_sha11_sum |
| | |

| | | |
|---|---|---|
| | **Public pharmaceutical expenditure as percentage of total pharmaceutical expenditure** | |
| | Data - % of total pharmaceutical expenditure | 67.6 |
| | Time | 2011 |
| | Reference | https://gateway.euro.who.int/en/indicators/hfa_580-6790-public-pharmaceutical-expenditure-as-of-total-pharmaceutical-expenditure/visualizations/#id=19675&tab=table |
| | | |
| | **Self-reported consultations of a medical doctor*** | |
| | Data - No contact, % of population according to the number of consultations of a medical doctor in the past 4 weeks | 50.7 |
| | Data - 1 contact, % of population according to the number of consultations of a medical doctor in the past 4 weeks | 24.2 |
| | Data - 2 contacts, % of population according to the number of consultations of a medical doctor in the past 4 weeks | 11.9 |
| | Data - 3 or more contacts, % of population according to the number of consultations of a medical doctor in the past 4 weeks | 13.2 |
| | Time | 2019 |
| | Reference | https://ec.europa.eu/eurostat/databrowser/view/HLTH_EHIS_AM2U/default/table?lang=en&category=hlth.hlth_care.hlth_consult / |

*Medical doctors include generalist medical practitioners and specialist medical practitioners

BELGIUM

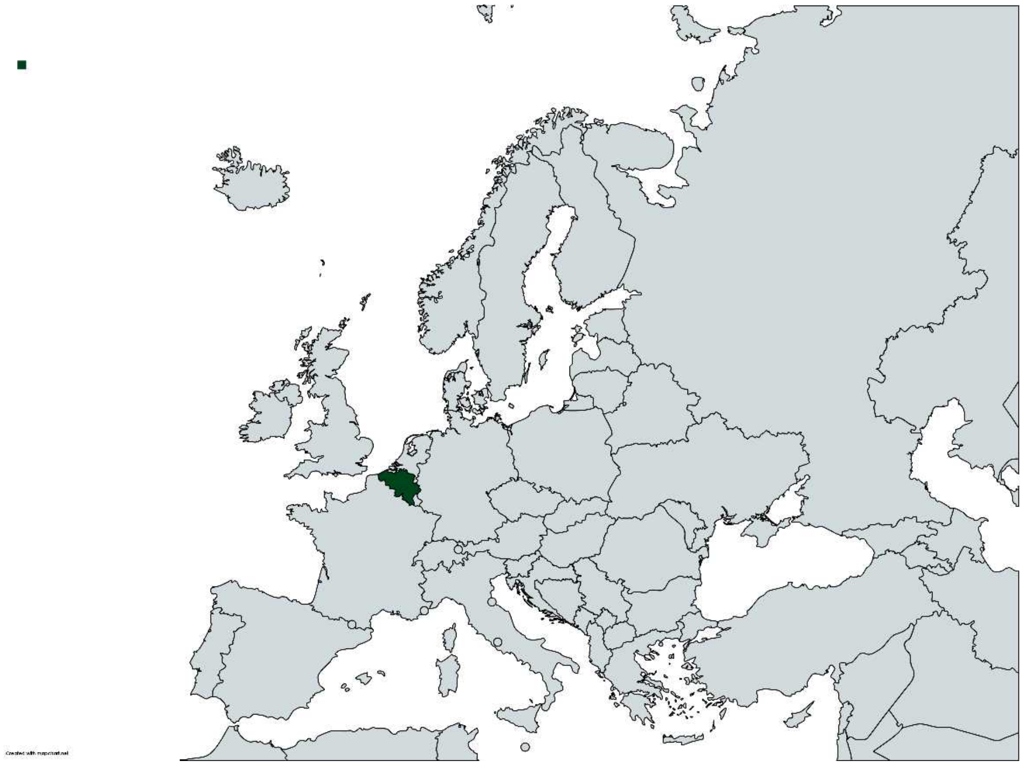

| Country | Belgium |
|---|---|

Country-specific data

| Country characteristics | Method of payment | |
|---|---|---|
| | Data | The patient only pays part of the total cost of a medicine and the sickness funds pay the remaining part to the pharmacist. |
| | Time | 2013 |
| | Reference | http://imi-protect.eu/documents/DUinventory_2013_COUNTRIESyear4_Dec2013.pdf |
| | | |
| | Medication adherence assessed and reported on the national level | |
| | Data | No |
| | Time | 2022 |
| | Reference | NA |
| | | |
| | Health care provider | |
| | Data | Health insurance system |
| | Time | 2013 |
| | Reference | http://imi-protect.eu/documents/DUinventory_2013_COUNTRIESyear4_Dec2013.pdf |
| | | |
| | Model of healthcare system financing | |
| | Data | Compulsory system of health insurance which main sources of funding are the social security contributions depending upon a persons's income (66%), and the subsidies from the federal government (10%). Other: indirect tax revenues (14%), allocated and diverse receipts (10%). |
| | Time | 2013 |
| | Reference | http://imi-protect.eu/documents/DUinventory_2013_COUNTRIESyear4_Dec2013.pdf |
| | | |
| | Proportion of population aged 65 years and over | |
| | Data - % of persons | 19.3 |
| | Time | 2021 |
| | Reference | https://ec.europa.eu/eurostat/databrowser/view/TPS00028/default/table?lang=en&category=demo.demo_ind / |
| | | |
| | Country population (projection) | |
| | Data - N of persons | 11507338 |
| | Time | 2020 |

| | | |
|---|---|---|
| | Reference | https://ec.europa.eu/eurostat/databrowser/view/CENS_HNMGA/default/table?lang=en&category=cens.cens_hn.cens_hnstr |

| | | |
|---|---|---|
| **Social/economic factors** | **Patient co-payment** | |
| | Data | Compayment consits a flat fee for medicine supplied and to a percentage of the real costs and limited to a ciling-fee. The flat fee varies also by reimbursement categories (i.e. A: 100%, B: 75/85%, C: 50%, Cs: 40%, Cx: 20%). |
| | Time | 2013 |
| | Reference | http://imi-protect.eu/documents/DUinventory_2013_COUNTRIESyear4_Dec2013.pdf |
| | | |
| | **Percentage of prescriptions dispensed at no cost to patients** | |
| | Data - % of prescriptions | NA |
| | Time | NA |
| | Reference | NA |
| | | |
| | **Population coverage** | |
| | Data | Universal access |
| | Time | 2013 |
| | Reference | http://imi-protect.eu/documents/DUinventory_2013_COUNTRIESyear4_Dec2013.pdf |
| | | |
| | **Availability of doctors' services for citizens at no payment** | |
| | Data | NA |
| | Time | NA |
| | Reference | NA |

| | | |
|---|---|---|
| **Therapy-related factors** | **Average number of medicines per patient** | |
| | Data - N of medicines per patient | NA |
| | Time | NA |
| | Reference | NA |
| | | |
| | **Proportion of 75 years and over who are taking more than 5 medications concurrently** | |
| | Data - % of persons | 36.1 |
| | Time | 2017 |
| | Reference | https://stats.oecd.org/Index.aspx?ThemeTreeId=11 |
| | | |
| | **Percentage of self-reported use of prescribed medicines** | |

| | | |
|---|---|---|
| | Data - % of persons | 59.7 |
| | Time | 2019 |
| | Reference | https://ec.europa.eu/eurostat/databrowser/view/HLTH_EHIS_MD1E__custom_3764895/default/table?lang=en / |

| | | |
|---|---|---|
| Patient-related factors | Percentage of persons reporting a chronic disease | |
| | Data - Asthma, % of persons | 5.8 |
| | Data - Chronic lower respiratory diseases, % of persons | 4 |
| | Data - High blood pressure, % of persons | 17.4 |
| | Data - Diabetes, % of persons | 5.8 |
| | Data - Chronic depression, % of persons | 7.3 |
| | Time | 2019 |
| | Reference | https://ec.europa.eu/eurostat/databrowser/view/HLTH_EHIS_CD1E/default/table?lang=en&category=hlth.hlth_state.hlth_srcm / |
| | | |
| | Percentage of self-perceived health - very good (16 years and over) | |
| | Data - % of persons | 30.3 |
| | Time | 2021 |
| | Reference | https://ec.europa.eu/eurostat/databrowser/view/HLTH_SILC_02/default/table?lang=en&category=hlth.hlth_state.hlth_sph/ |
| | | |
| | Percenatge of persons with current depressive symptoms | |
| | Data - % of persons | 8.4 |
| | Time | 2019 |
| | Reference | https://ec.europa.eu/eurostat/databrowser/view/HLTH_EHIS_MH1E/default/table?lang=en&category=hlth.hlth_state.hlth_sph / |

| | | |
|---|---|---|
| Condition-related factor | General health literacy | |
| | Data - Inadequate health literacy, % of persons | 27 |
| | Data - Problematic health literacy, % of persons | 35 |
| | Data - Sufficient health literacy, % of persons | 26 |
| | Data - Excellent health literacy, % of persons | 12 |
| | Time | 2021 |
| | Reference | https://m-pohl.net/Int_Report_methdology_results_recommendations |

| | | |
|---|---|---|
| Healthcare system-relat | Percentage of patients receiving adherence interventions | |
| | Data - % of persons | NA |

| | |
|---|---|
| Time | NA |
| Reference | NA |
| | |
| **Nationwide availability of e-prescription** | |
| Data | NA |
| Time | NA |
| Reference | NA |
| | |
| **Waiting time for prescriptions / medical appointments** | |
| Data | NA |
| Time | NA |
| Reference | NA |
| | |
| **Number of practising physicians per 100,000 inhabitants** | |
| Data - N of practisisng physicians per 100,000 inhabitants | 321.28 |
| Time | 2020 |
| Reference | https://ec.europa.eu/eurostat/databrowser/view/TPS00044/default/table?lang=en&category=hlth.hlth_care.hlth_res.hlth_staff%20%2F |
| | |
| **Proportion of health care expenditure on pharmaceuticals** | |
| Data - % of health care expenditure | 11.553 |
| Time | 2020 |
| Reference | https://data.oecd.org/healthres/pharmaceutical-spending.htm |
| | |
| **Number of practising pharmacists per 100,000 inhabitants** | |
| Data - N of practisisng pharmacists per 100,000 inhabitants | 128.57 |
| Time | 2020 |
| Reference | https://ec.europa.eu/eurostat/databrowser/view/HLTH_RS_PRS1__custom_4104351/default/table?lang=en |
| | |
| **Total health care expenditure as percentage of GDP** | |
| Data - % of GDP | 11.06 |
| Time | 2020 |
| Reference | https://ec.europa.eu/eurostat/databrowser/view/TPS00207/default/table?lang=en&category=hlth.hlth_care.hlth_sha11.hlth_sha11_sum |

| | | |
|---|---|---|
| | | |
| | **Public pharmaceutical expenditure as percentage of total pharmaceutical expenditure** | |
| | Data - % of total pharmaceutical expenditure | 63.3 |
| | Time | 2020 |
| | Reference | https://gateway.euro.who.int/en/indicators/hfa_580-6790-public-pharmaceutical-expenditure-as-of-total-pharmaceutical-expenditure/visualizations/#id=19675&tab=table |
| | | |
| | **Self-reported consultations of a medical doctor*** | |
| | Data - No contact, % of population according to the number of consultations of a medical doctor in the past 4 weeks | 57.2 |
| | Data - 1 contact, % of population according to the number of consultations of a medical doctor in the past 4 weeks | 26.4 |
| | Data - 2 contacts, % of population according to the number of consultations of a medical doctor in the past 4 weeks | 10.3 |
| | Data - 3 or more contacts, % of population according to the number of consultations of a medical doctor in the past 4 weeks | 6.1 |
| | Time | 2019 |
| | Reference | https://ec.europa.eu/eurostat/databrowser/view/HLTH_EHIS_AM2U/default/table?lang=en&category=hlth.hlth_care.hlth_consult / |

*Medical doctors include generalist medical practitioners and specialist medical practitioners

## BOSNIA AND HERZEGOVINA

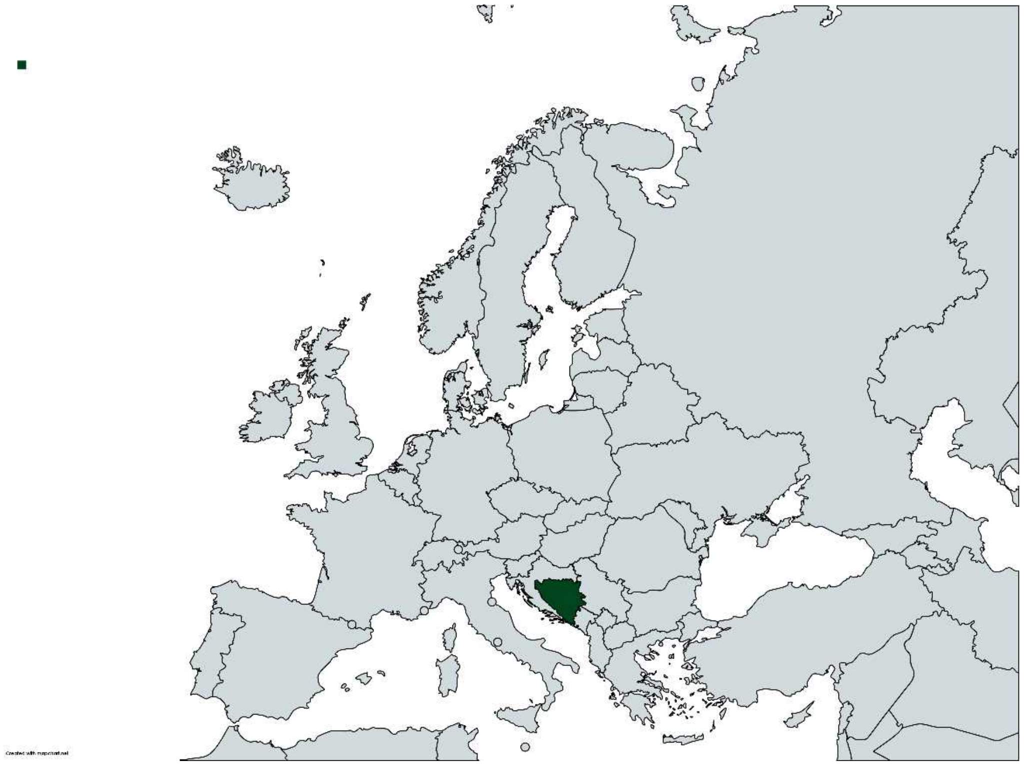

| Country | Bosnia and Herzegovina |
|---|---|

**Country-specific data**

| Country characteristics | Method of payment | |
|---|---|---|
| | Data | Cost of medicines is payed totally (100%, essential medicines list) or partially (50%) by health insurance fund. |
| | Time | 2022 |
| | Reference | https://eurohealthobservatory.who.int/publications/i/health-systems-in-action-bosnia-and-herzegovina-2022 |
| | | |
| | Medication adherence assessed and reported on the national level | |
| | Data | No |
| | Time | 2022 |
| | Reference | NA |
| | | |
| | Health care provider | |
| | Data | 13 health insurance funds in total: Health Insurance and reinsurance Institute of Federation of Bosnia and Herzegovina (1), Cantonal institutions for Health Insurance (10), Health Insurance Fund of the Republic of Srpska (1), Health Insurance Institute of Brcko District (1). |
| | Time | 2022 |
| | Reference | https://eurohealthobservatory.who.int/publications/i/health-systems-in-action-bosnia-and-herzegovina-2022 |
| | | |
| | Model of healthcare system financing | |
| | Data | The basic division of healthcare system financing includes public, private sector and foreign sources of financing health care. In the context of the public funding there is division between the central, entity, cantonal and local level, as well as mandatory health insurance, while the private sector is divided into private health insurance, direct payments for health care of the household, i.e. household expenditure out of pocket, nonprofit organizations, and institutions and companies (except those from the health insurance). |
| | Time | 2020 |
| | Reference | https://bhas.gov.ba/data/Publikacije/Saopstenja/2022/NHA_01_2020_Y1_1_BS.pdf |
| | | |
| | Proportion of population aged 65 years and over | |
| | Data - % of persons | 14.22 |
| | Time | 2013 |
| | Reference | https://www.popis.gov.ba/popis2013/doc/Knjiga1/K1_S_E.pdf |
| | | |

| | | |
|---|---|---|
| | **Country population (projection)** | |
| | Data - N of persons | 3453000 |
| | Time | 2021 |
| | Reference | https://bhas.gov.ba/data/Publikacije/Bilteni/2022/DEM_00_2021_TB_1_BS.pdf |

| | | |
|---|---|---|
| Social/economic factors | **Patient co-payment** | |
| | Data | Outpatient medicines are subject to fixed, percentage and refference pricing co-payments. |
| | Time | 2022 |
| | Reference | https://eurohealthobservatory.who.int/publications/i/health-systems-in-action-bosnia-and-herzegovina-2022 |
| | | |
| | **Percentage of prescriptions dispensed at no cost to patients** | |
| | Data - % of prescriptions | NA |
| | Time | NA |
| | Reference | NA |
| | | |
| | **Population coverage** | |
| | Data | Universal access |
| | Time | 2022 |
| | Reference | https://eurohealthobservatory.who.int/publications/i/health-systems-in-action-bosnia-and-herzegovina-2022 |
| | | |
| | **Availability of doctors' services for citizens at no payment** | |
| | Data | NA |
| | Time | NA |
| | Reference | NA |
| | | |
| Therapy-related factors | **Average number of medicines per patient** | |
| | Data - N of medicines per patient | NA |
| | Time | NA |
| | Reference | NA |
| | | |
| | **Proportion of 75 years and over who are taking more than 5 medications concurrently** | |
| | Data - % of persons | NA |
| | Time | NA |

| | | |
|---|---|---|
| | Reference | NA |
| | | |
| | **Percentage of self-reported use of prescribed medicines** | |
| | Data - % of persons | NA |
| | Time | NA |
| | Reference | NA |

| | | |
|---|---|---|
| Patient-related factors | **Percentage of persons reporting a chronic disease** | |
| | Data - Asthma, % of persons | NA |
| | Data - Chronic lower respiratory diseases, % of persons | NA |
| | Data - High blood pressure, % of persons | NA |
| | Data - Diabetes, % of persons | NA |
| | Data - Chronic depression, % of persons | NA |
| | Time | NA |
| | Reference | NA |
| | | |
| | **Percentage of self-perceived health - very good (16 years and over)** | |
| | Data - % of persons | NA |
| | Time | NA |
| | Reference | NA |
| | | |
| | **Percenatge of persons with current depressive symptoms** | |
| | Data - % of persons | NA |
| | Time | NA |
| | Reference | NA |

| | | |
|---|---|---|
| Condition-related factor | **General health literacy** | |
| | Data - Inadequate health literacy, % of persons | NA |
| | Data - Problematic health literacy, % of persons | NA |
| | Data - Sufficient health literacy, % of persons | NA |
| | Data - Excellent health literacy, % of persons | NA |
| | Time | NA |
| | Reference | NA |

| | | |
|---|---|---|
| Healthcare system-relat | **Percentage of patients receiving adherence interventions** | |
| | Data - % of persons | NA |
| | Time | NA |
| | Reference | NA |
| | | |
| | **Nationwide availability of e-prescription** | |
| | Data | NA |
| | Time | NA |
| | Reference | NA |
| | | |
| | **Waiting time for prescriptions / medical appointments** | |
| | Data | NA |
| | Time | NA |
| | Reference | NA |
| | | |
| | **Number of practising physicians per 100,000 inhabitants** | |
| | Data - N of practisisng physicians per 100,000 inhabitants | 216 |
| | Time | 2015 |
| | Reference | https://eurohealthobservatory.who.int/publications/i/health-systems-in-action-bosnia-and-herzegovina-2022 |
| | | |
| | **Proportion of health care expenditure on pharmaceuticals** | |
| | Data - % of health care expenditure | NA |
| | Time | NA |
| | Reference | NA |
| | | |
| | **Number of practising pharmacists per 100,000 inhabitants** | |
| | Data - N of practisisng pharmacists per 100,000 inhabitants | 80 |
| | Time | 2022 |
| | Reference | Expert opinion |
| | | |
| | **Total health care expenditure as percentage of GDP** | |
| | Data - % of GDP | 9.84 |

| | | |
|---|---|---|
| | Time | 2020 |
| | Reference | https://ec.europa.eu/eurostat/databrowser/view/TPS00207/default/table?lang=en&category=hlth.hlth_care.hlth_sha11.hlth_sha11_sum |
| | | |
| | **Public pharmaceutical expenditure as percentage of total pharmaceutical expenditure** | |
| | Data - % of total pharmaceutical expenditure | 42.5 |
| | Time | 2017 |
| | Reference | https://gateway.euro.who.int/en/indicators/hfa_580-6790-public-pharmaceutical-expenditure-as-of-total-pharmaceutical-expenditure/visualizations/#id=19675&tab=table |
| | | |
| | **Self-reported consultations of a medical doctor*** | |
| | Data - No contact, % of population according to the number of consultations of a medical doctor in the past 4 weeks | NA |
| | Data - 1 contact, % of population according to the number of consultations of a medical doctor in the past 4 weeks | NA |
| | Data - 2 contacts, % of population according to the number of consultations of a medical doctor in the past 4 weeks | NA |
| | Data - 3 or more contacts, % of population according to the number of consultations of a medical doctor in the past 4 weeks | NA |
| | Time | NA |
| | Reference | NA |

*Medical doctors include generalist medical practitioners and specialist medical practitioners

BULGARIA

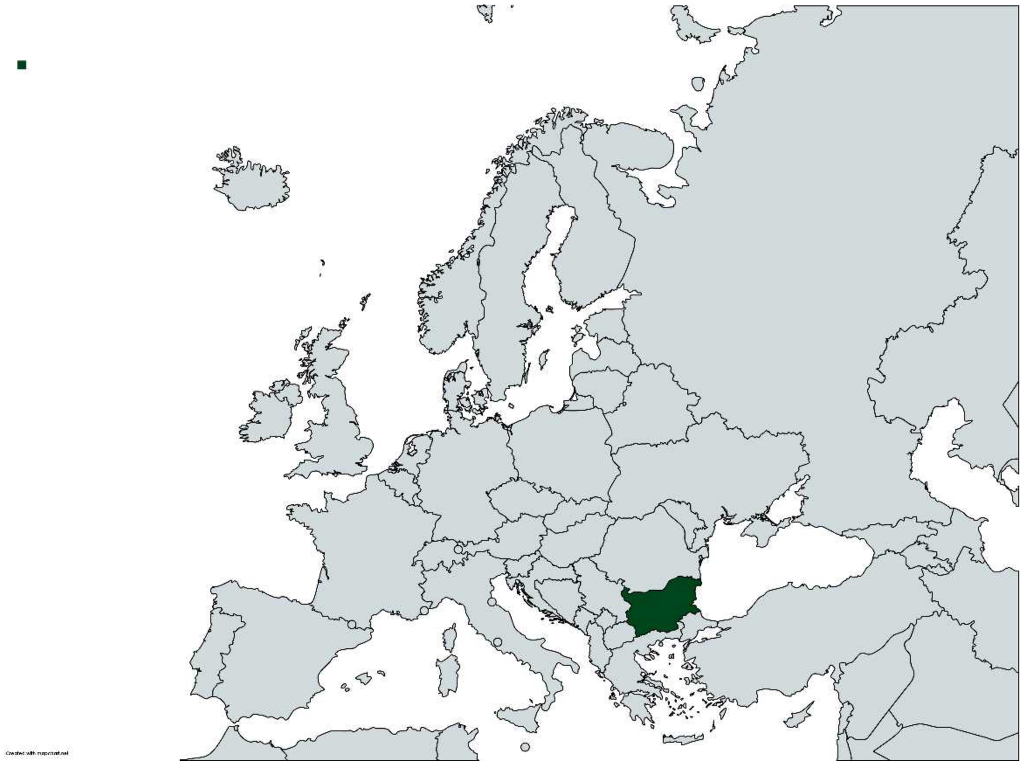

| Country | Bulgaria |
|---|---|

**Country-specific data**

| Country characteristics | Method of payment | |
|---|---|---|
| | Data | The PDL is having 3 main annexes according to the financing institution<br>- Annex 1 medicnes are paid by the NHIF for ambulatory care<br>(100%, 75%,50%, and 25% reimbursement). Annex 2 are the medicnes<br>paid by hospital budgets, Annex 3 are paid by the MoH budget<br>(vaccines, HIV, antituberculosis medicines). |
| | Time | 2023 |
| | Reference | ncpr.bg |
| | | |
| | **Medication adherence assessed and reported on the national level** | |
| | Data | No |
| | Time | 2022 |
| | Reference | NA |
| | | |
| | **Health care provider** | |
| | Data | Health insurance system with compulsory and voluntary health insurance managed by the National Health Insurance Fund (NHIF). The Ministry of Health is responsible of vaccines, tuberculosis, anti-viral therapy, therapeutic radio pharmaceuticals. In addition it is also responsible for maternal care. |
| | Time | 2022 |
| | Reference | https://portal.ncpr.bg/registers/pages/register/list-medicament.xhtml |
| | | |
| | **Model of healthcare system financing** | |
| | Data | Mixed pubilc-private: compulsory statutory health insurance (SHI), contributions, taxes, out-of-pocket payments, voluntary health insurance premiums, corporate payments, donations and external funding. |
| | Time | 2018 |
| | Reference | Dimova A, Rohova M, Koeva S, Atanasova E, Koeva-Dimitrova L, Kostadinova T, Spranger A. Bulgaria: Health System Review. Health Syst Transit. 2018 Sep;20(4):1-230. |
| | | |
| | **Proportion of population aged 65 years and over** | |
| | Data - % of persons | 21.7 |
| | Time | 2021 |
| | Reference | https://ec.europa.eu/eurostat/databrowser/view/TPS00028/default/table?lang=en&category=demo.demo_ind / |

| | | |
|---|---|---|
| | | |
| | **Country population (projection)** | |
| | Data - N of persons | 6838937 |
| | Time | 2121 |
| | Reference | https://nsi.bg/en/content/2974/population |
| Social/economic factors | **Patient co-payment** | |
| | Data | The NCPRMP defines the reimbursement level of each "International non proprietary name" group. Co-payment for pharmaceuticals is 0%( there are fully reimbursed medicines), 25 %, 50 %,75 %. Direct payments occur in three cases: for services not included in the basic package at prices set by individual providers, for services that are included but patients go outside the standard public patient pathway, and for uninsured individuals. |
| | Time | 2022 |
| | Reference | Dimova A, Rohova M, Koeva S, Atanasova E, Koeva-Dimitrova L, Kostadinova T, Spranger A. Bulgaria: Health System Review. Health Syst Transit. 2018 Sep;20(4):1-230. Dimova A, Rohova M, Koeva S, Atanasova E, Koeva-Dimitrova L, Kostadinova T, Spranger A, Polin K (2022), Bulgaria: Health System Summary, 2022. WHO Regional Office for Europe on behalf of the European Observatory on Health Systems and Policies, Copenhagen. ISBN 9789289059299 (PDF); Available at: https://eurohealthobservatory.who.int/publications/i/bulgaria-health-system-summary |
| | | |
| | **Percentage of prescriptions dispensed at no cost to patients** | |
| | Data - % of prescriptions | NA |
| | Time | NA |
| | Reference | NA |
| | | |
| | **Population coverage** | |
| | Data | 85.2 %, compulsory health insurance for all Bulgarian citizens. |
| | Time | 2022 |
| | Reference | Dimova A, Rohova M, Koeva S, Atanasova E, Koeva-Dimitrova L, Kostadinova T, Spranger A, Polin K (2022), Bulgaria: Health System Summary, 2022. WHO Regional Office for Europe on behalf of the European Observatory on Health Systems and Policies, Copenhagen. ISBN 9789289059299 (PDF); Available at: https://eurohealthobservatory.who.int/publications/i/bulgaria-health-system-summary |
| | | |
| | **Availability of doctors' services for citizens at no payment** | |
| | Data | All services that are included in the basic health insurance pacakge are available at no payment for the citisens. |
| | Time | 2023 |
| | Reference | www.nhif.bg |
| | | |

| | | |
|---|---|---|
| Therapy-related factors | **Average number of medicines per patient** | |
| | Data - N of medicines per patient | NA |
| | Time | NA |
| | Reference | NA |
| | | |
| | **Proportion of 75 years and over who are taking more than 5 medications concurrently** | |
| | Data - % of persons | NA |
| | Time | NA |
| | Reference | NA |
| | | |
| | **Percentage of self-reported use of prescribed medicines** | |
| | Data - % of persons | 41.2 |
| | Time | 2019 |
| | Reference | https://ec.europa.eu/eurostat/databrowser/view/HLTH_EHIS_MD1E__custom_3764895/default/table?lang=en / |

| | | |
|---|---|---|
| Patient-related factors | **Percentage of persons reporting a chronic disease** | |
| | Data - Asthma, % of persons | 2.2 |
| | Data - Chronic lower respiratory diseases, % of persons | 3.2 |
| | Data - High blood pressure, % of persons | 29.7 |
| | Data - Diabetes, % of persons | 6.9 |
| | Data - Chronic depression, % of persons | 2.7 |
| | Time | 2019 |
| | Reference | https://ec.europa.eu/eurostat/databrowser/view/HLTH_EHIS_CD1E/default/table?lang=en&category=hlth.hlth_state.hlth_srcm / |
| | | |
| | **Percentage of self-perceived health - very good (16 years and over)** | |
| | Data - % of persons | 18.2 |
| | Time | 2021 |
| | Reference | https://ec.europa.eu/eurostat/databrowser/view/HLTH_SILC_02/default/table?lang=en&category=hlth.hlth_state.hlth_sph/ |
| | | |
| | **Percenatge of persons with current depressive symptoms** | |
| | Data - % of persons | 5 |
| | Time | 2019 |
| | Reference | https://ec.europa.eu/eurostat/databrowser/view/HLTH_EHIS_MH1E/default/table?lang=en&category=hlth.hlth_state.hlth_sph / |

| | | |
|---|---|---|
| Condition-related factor: | **General health literacy** | |
| | Data - Inadequate health literacy, % of persons | 16 |
| | Data - Problematic health literacy, % of persons | 41 |
| | Data - Sufficient health literacy, % of persons | 34 |
| | Data - Excellent health literacy, % of persons | 9 |
| | Time | 2021 |
| | Reference | https://m-pohl.net/Int_Report_methdology_results_recommendations |

| | | |
|---|---|---|
| Healthcare system-relat | **Percentage of patients receiving adherence interventions** | |
| | Data - % of persons | NA |
| | Time | NA |
| | Reference | NA |
| | | |
| | **Nationwide availability of e-prescription** | |
| | Data | Yes |
| | Time | 2022 |
| | Reference | https://www.bda.bg |
| | | |
| | **Waiting time for prescriptions / medical appointments** | |
| | Data | 20 minutes |
| | Time | 2022 |
| | Reference | www.nhif.bg |
| | | |
| | **Number of practising physicians per 100,000 inhabitants** | |
| | Data - N of practisisng physicians per 100,000 inhabitants | 427.69 |
| | Time | 2020 |
| | Reference | https://ec.europa.eu/eurostat/databrowser/view/TPS00044/default/table?lang=en&category=hlth.hlth_care.hlth_res.hlth_staff%20% 2F |
| | | |
| | **Proportion of health care expenditure on pharmaceuticals** | |
| | Data - % of health care expenditure | 32.482 |
| | Time | 2020 |
| | Reference | https://data.oecd.org/healthres/pharmaceutical-spending.htm |

| | | |
|---|---|---|
| | | |
| | **Number of practising pharmacists per 100,000 inhabitants** | |
| | Data - N of practisisng pharmacists per 100,000 inhabitants | 88.21 |
| | Time | 2021 |
| | Reference | https://ec.europa.eu/eurostat/databrowser/view/HLTH_RS_PRS1__custom_4104351/default/table?lang=en |
| | | |
| | **Total health care expenditure as percentage of GDP** | |
| | Data - % of GDP | 8.52 |
| | Time | 2020 |
| | Reference | https://ec.europa.eu/eurostat/databrowser/view/TPS00207/default/table?lang=en&category=hlth.hlth_care.hlth_sha11.hlth_sha11_sum |
| | | |
| | **Public pharmaceutical expenditure as percentage of total pharmaceutical expenditure** | |
| | Data - % of total pharmaceutical expenditure | NA |
| | Time | NA |
| | Reference | NA |
| | | |
| | **Self-reported consultations of a medical doctor*** | |
| | Data - No contact, % of population according to the number of consultations of a medical doctor in the past 4 weeks | 64.6 |
| | Data - 1 contact, % of population according to the number of consultations of a medical doctor in the past 4 weeks | 23.6 |
| | Data - 2 contacts, % of population according to the number of consultations of a medical doctor in the past 4 weeks | 7.8 |
| | Data - 3 or more contacts, % of population according to the number of consultations of a medical doctor in the past 4 weeks | 4 |
| | Time | 2019 |
| | Reference | https://ec.europa.eu/eurostat/databrowser/view/HLTH_EHIS_AM2U/default/table?lang=en&category=hlth.hlth_care.hlth_consult / |

*Medical doctors include generalist medical practitioners and specialist medical practitioners

CROATIA

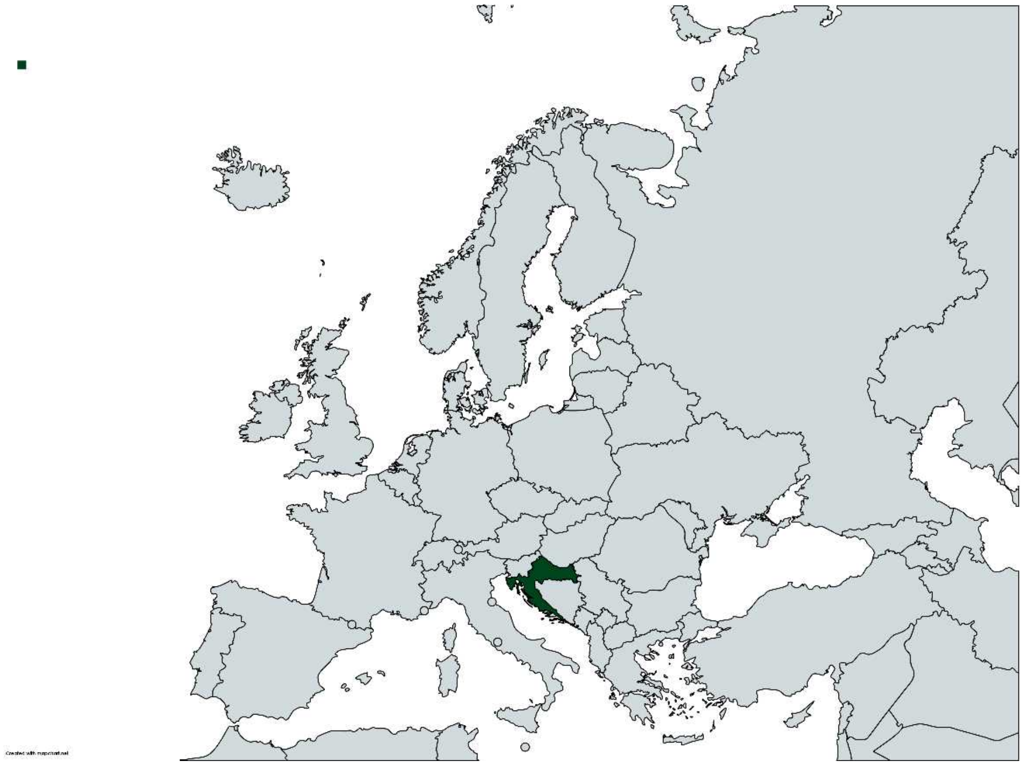

| Country | Croatia |
|---|---|

Country-specific data

| Country characteristics | Method of payment | |
|---|---|---|
| | Data | The patient only pays part of the total cost of a medicine and the sickness funds pay the remaining part. Expect for the basic list. Article 18. enable this remaining part to be paid by additional voluntary insurance of 10 EUR. |
| | Time | 2013 |
| | Reference | http://imi-protect.eu/documents/DUinventory_2013_COUNTRIESyear4_Dec2013.pdf |
| | | |
| | Medication adherence assessed and reported on the national level | |
| | Data | Self-report quetionnaire in pharmacy |
| | Time | 2014 |
| | Reference | Psychiatria Danubina 2014;vol.26,suppl.3, pp 498-508 |
| | | |
| | Health care provider | |
| | Data | Compulsory public Health Insurance Fund (Croatian Institute for Health Insurance) |
| | Time | 2013 |
| | Reference | http://imi-protect.eu/documents/DUinventory_2013_COUNTRIESyear4_Dec2013.pdf |
| | | |
| | Model of healthcare system financing | |
| | Data | Mixed system of financing: health insurance contributions, co-payments, volunatry complementary haelth insurance, privately provided supplementary health insurance, the state budget and local self-administration county units' budgets. |
| | Time | 2013 |
| | Reference | http://imi-protect.eu/documents/DUinventory_2013_COUNTRIESyear4_Dec2013.pdf |
| | | |
| | Proportion of population aged 65 years and over | |
| | Data - % of persons | 21.4 |
| | Time | 2021 |
| | Reference | https://ec.europa.eu/eurostat/databrowser/view/TPS00028/default/table?lang=en&category=demo.demo_ind / |
| | | |
| | Country population (projection) | |
| | Data - N of persons | 4056285 |
| | Time | 2020 |

| | | |
|---|---|---|
| | Reference | https://ec.europa.eu/eurostat/databrowser/view/CENS_HNMGA/default/table?lang=en&category=cens.cens_hn.cens_hnstr |

| | | |
|---|---|---|
| Social/economic factors | **Patient co-payment** | |
| | Data | Prescription fee of HRK15 (~ EUR2) per year on mediicnes. The basic list: 0% |
| | Time | 2013 |
| | Reference | http://imi-protect.eu/documents/DUinventory_2013_COUNTRIESyear4_Dec2013.pdf |
| | | |
| | **Percentage of prescriptions dispensed at no cost to patients** | |
| | Data - % of prescriptions | NA |
| | Time | NA |
| | Reference | NA |
| | | |
| | **Population coverage** | |
| | Data | Universal access |
| | Time | 2013 |
| | Reference | http://imi-protect.eu/documents/DUinventory_2013_COUNTRIESyear4_Dec2013.pdf |
| | | |
| | **Availability of doctors' services for citizens at no payment** | |
| | Data | NA |
| | Time | NA |
| | Reference | NA |
| | | |
| Therapy-related factors | **Average number of medicines per patient** | |
| | Data - N of medicines per patient | NA |
| | Time | NA |
| | Reference | NA |
| | | |
| | **Proportion of 75 years and over who are taking more than 5 medications concurrently** | |
| | Data - % of persons | NA |
| | Time | NA |
| | Reference | NA |
| | | |
| | **Percentage of self-reported use of prescribed medicines** | |

| | | |
|---|---|---|
| | Data - % of persons | 62.3 |
| | Time | 2019 |
| | Reference | https://ec.europa.eu/eurostat/databrowser/view/HLTH_EHIS_MD1E__custom_3764895/default/table?lang=en / |

| | | |
|---|---|---|
| **Patient-related factors** | **Percentage of persons reporting a chronic disease** | |
| | Data - Asthma, % of persons | 4.8 |
| | Data - Chronic lower respiratory diseases, % of persons | 5.5 |
| | Data - High blood pressure, % of persons | 37.3 |
| | Data - Diabetes, % of persons | 12.1 |
| | Data - Chronic depression, % of persons | 11.6 |
| | Time | 2019 |
| | Reference | https://ec.europa.eu/eurostat/databrowser/view/HLTH_EHIS_CD1E/default/table?lang=en&category=hlth.hlth_state.hlth_srcm / |
| | | |
| | **Percentage of self-perceived health - very good (16 years and over)** | |
| | Data - % of persons | 31.4 |
| | Time | 2021 |
| | Reference | https://ec.europa.eu/eurostat/databrowser/view/HLTH_SILC_02/default/table?lang=en&category=hlth.hlth_state.hlth_sph/ |
| | | |
| | **Percenatge of persons with current depressive symptoms** | |
| | Data - % of persons | 8.9 |
| | Time | 2019 |
| | Reference | https://ec.europa.eu/eurostat/databrowser/view/HLTH_EHIS_MH1E/default/table?lang=en&category=hlth.hlth_state.hlth_sph / |

| | | |
|---|---|---|
| **Condition-related factor** | **General health literacy** | |
| | Data - Inadequate health literacy, % of persons | 11 |
| | Data - Problematic health literacy, % of persons | 58 |
| | Data - Sufficient health literacy, % of persons | 26 |
| | Data - Excellent health literacy, % of persons | 6 |
| | Time | 2021 |
| | Reference | https://www.telegram.hr/pitanje-zdravlja/proveli-smo-prvo-istrazivanje-o-zdravstvenoj-pismenosti-u-hrvatskoj-stanje-jednostavno-nije-dobro/ |

| | | |
|---|---|---|
| **Healthcare system-relat** | **Percentage of patients receiving adherence interventions** | |
| | Data - % of persons | NA |

| | |
|---|---|
| Time | NA |
| Reference | NA |
| | |
| **Nationwide availability of e-prescription** | |
| Data | Yes |
| Time | 2017 |
| Reference | https://hzzo.hr/wp-content/uploads/2017/02/PROC_TEKST_PRAV_o_nac_prop_i_izdav_lijekova_na_recept_17_15.pdf |
| | |
| **Waiting time for prescriptions / medical appointments** | |
| Data | NA |
| Time | NA |
| Reference | NA |
| | |
| **Number of practising physicians per 100,000 inhabitants** | |
| Data - N of practisisng physicians per 100,000 inhabitants | 359.95 |
| Time | 2021 |
| Reference | https://ec.europa.eu/eurostat/databrowser/view/TPS00044/default/table?lang=en&category=hlth.hlth_care.hlth_res.hlth_staff%20%2F |
| | |
| **Proportion of health care expenditure on pharmaceuticals** | |
| Data - % of health care expenditure | 20.866 |
| Time | 2020 |
| Reference | https://data.oecd.org/healthres/pharmaceutical-spending.htm |
| | |
| **Number of practising pharmacists per 100,000 inhabitants** | |
| Data - N of practisisng pharmacists per 100,000 inhabitants | 78.6 |
| Time | 2020 |
| Reference | https://ec.europa.eu/eurostat/databrowser/view/HLTH_RS_PRS1__custom_4104351/default/table?lang=en |
| | |
| **Total health care expenditure as percentage of GDP** | |
| Data - % of GDP | 7.77 |
| Time | 2020 |
| Reference | https://ec.europa.eu/eurostat/databrowser/view/TPS00207/default/table?lang=en&category=hlth.hlth_care.hlth_sha11.hlth_sha11_sum |

| | | |
|---|---|---|
| | | |
| | **Public pharmaceutical expenditure as percentage of total pharmaceutical expenditure** | |
| | Data - % of total pharmaceutical expenditure | 79 |
| | Time | 2020 |
| | Reference | https://gateway.euro.who.int/en/indicators/hfa_580-6790-public-pharmaceutical-expenditure-as-of-total-pharmaceutical-expenditure/visualizations/#id=19675&tab=table |
| | | |
| | **Self-reported consultations of a medical doctor*** | |
| | Data - No contact, % of population according to the number of consultations of a medical doctor in the past 4 weeks | 49.8 |
| | Data - 1 contact, % of population according to the number of consultations of a medical doctor in the past 4 weeks | 22.7 |
| | Data - 2 contacts, % of population according to the number of consultations of a medical doctor in the past 4 weeks | 12.7 |
| | Data - 3 or more contacts, % of population according to the number of consultations of a medical doctor in the past 4 weeks | 14.8 |
| | Time | 2019 |
| | Reference | https://ec.europa.eu/eurostat/databrowser/view/HLTH_EHIS_AM2U/default/table?lang=en&category=hlth.hlth_care.hlth_consult / |

*Medical doctors include generalist medical practitioners and specialist medical practitioners

CYPRUS

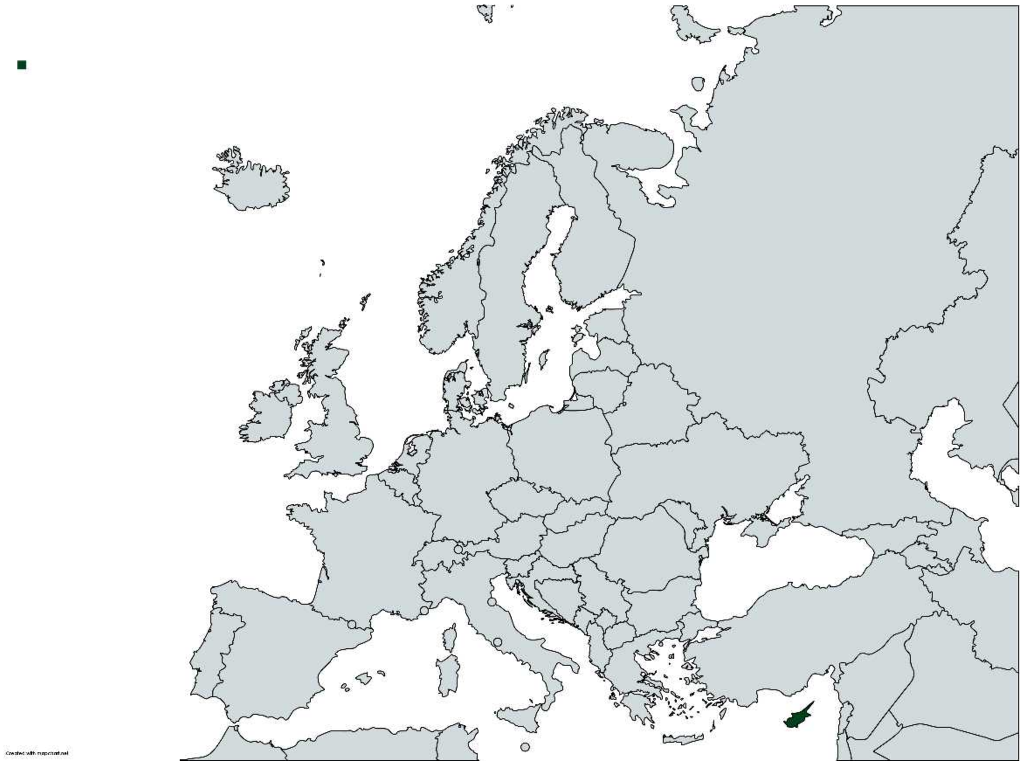

| Country | Cyprus |
|---|---|

Country-specific data

| Country characteristics | Method of payment | |
|---|---|---|
| | Data | Medicines are reimbursed by Health Insurance Organisation |
| | Time | 2022 |
| | Reference | https://www.sciencedirect.com/science/article/pii/S0168851021000051?via%3Dihub |
| | | |
| | Medication adherence assessed and reported on the national level | |
| | Data | No |
| | Time | 2022 |
| | Reference | Expert opinion |
| | | |
| | Health care provider | |
| | Data | Health Insurance Organisation |
| | Time | 2022 |
| | Reference | Expert opinion |
| | | |
| | Model of healthcare system financing | |
| | Data | For the financial operation of the NHS, a Health Insurance Fund was established. The Fund's main source of financing is contributions levied on wages, incomes and pensions and state revenues. Some of the applicable contributions are the following: Employeess contribute 2.65% on their income, employers 2.9% on employee income, self-employed 4% on their income and government contributes 4.7% on employee and self-employee income |
| | Time | 2022 |
| | Reference | https://www.sciencedirect.com/science/article/pii/S0168851021000051?via%3Dihub |
| | | |
| | Proportion of population aged 65 years and over | |
| | Data - % of persons | 20.2 |
| | Time | 2021 |
| | Reference | https://data.worldbank.org/indicator/SP.POP.65UP.TO?locations=CY |
| | | |
| | Country population (projection) | |
| | Data - N of persons | 887331 |

| | | |
|---|---|---|
| | Time | 2020 |
| | Reference | https://ec.europa.eu/eurostat/databrowser/view/CENS_HNMGA/default/table?lang=en&category=cens.cens_hn.cens_hnstr |

| | | |
|---|---|---|
| Social/economic factors | **Patient co-payment** | |
| | Data | In Cyprus,co -payment applies as following: one euro per pharmaceutical product, 6 for visit to specialist physician, 10 for imaging services, one euro per lab test, and 10 per allied health professionals. Co-payments accumulate to annual maximum cap of 150 or 75 EUR. |
| | Time | 2022 |
| | Reference | Expert opinion |
| | | |
| | **Percentage of prescriptions dispensed at no cost to patients** | |
| | Data - % of prescriptions | 1400 out of 2071 medicines of NHS formulary have only co-payment and no personal contribution |
| | Time | 2023 |
| | Reference | www.gesy.org.cy |
| | | |
| | **Population coverage** | |
| | Data | Universal health coverage |
| | Time | 2023 |
| | Reference | www.gesy.org.cy |
| | | |
| | **Availability of doctors' services for citizens at no payment** | |
| | Data | NA |
| | Time | NA |
| | Reference | NA |

| | | |
|---|---|---|
| Therapy-related factors | **Average number of medicines per patient** | |
| | Data - N of medicines per patient | NA |
| | Time | NA |
| | Reference | NA |
| | | |
| | **Proportion of 75 years and over who are taking more than 5 medications concurrently** | |
| | Data - % of persons | NA |
| | Time | NA |
| | Reference | NA |

| | | |
|---|---|---|
| | | |
| | **Percentage of self-reported use of prescribed medicines** | |
| | Data - % of persons | 42.1 |
| | Time | 2019 |
| | Reference | https://ec.europa.eu/eurostat/databrowser/view/HLTH_EHIS_MD1E__custom_3764895/default/table?lang=en / |

| | | |
|---|---|---|
| Patient-related factors | **Percentage of persons reporting a chronic disease** | |
| | Data - Asthma, % of persons | 4 |
| | Data - Chronic lower respiratory diseases, % of persons | 2.9 |
| | Data - High blood pressure, % of persons | 18.9 |
| | Data - Diabetes, % of persons | 7 |
| | Data - Chronic depression, % of persons | 4.7 |
| | Time | 2019 |
| | Reference | https://ec.europa.eu/eurostat/databrowser/view/HLTH_EHIS_CD1E/default/table?lang=en&category=hlth.hlth_state.hlth_srcm / |
| | | |
| | **Percentage of self-perceived health - very good (16 years and over)** | |
| | Data - % of persons | 46.1 |
| | Time | 2021 |
| | Reference | https://ec.europa.eu/eurostat/databrowser/view/HLTH_SILC_02/default/table?lang=en&category=hlth.hlth_state.hlth_sph/ |
| | | |
| | **Percenatge of persons with current depressive symptoms** | |
| | Data - % of persons | 2.5 |
| | Time | 2019 |
| | Reference | https://ec.europa.eu/eurostat/databrowser/view/HLTH_EHIS_MH1E/default/table?lang=en&category=hlth.hlth_state.hlth_sph / |

| | | |
|---|---|---|
| Condition-related factor | **General health literacy** | |
| | Data - Inadequate health literacy, % of persons | NA |
| | Data - Problematic health literacy, % of persons | NA |
| | Data - Sufficient health literacy, % of persons | NA |
| | Data - Excellent health literacy, % of persons | NA |
| | Time | NA |
| | Reference | NA |

| Healthcare system-relat | Percentage of patients receiving adherence interventions | |
|---|---|---|
| | Data - % of persons | NA |
| | Time | NA |
| | Reference | NA |
| | | |
| | **Nationwide availability of e-prescription** | |
| | Data | NA |
| | Time | NA |
| | Reference | NA |
| | | |
| | **Waiting time for prescriptions / medical appointments** | |
| | Data | NA |
| | Time | NA |
| | Reference | NA |
| | | |
| | **Number of practising physicians per 100,000 inhabitants** | |
| | Data - N of practisisng physicians per 100,000 inhabitants | 427.23 |
| | Time | 2019 |
| | Reference | https://ec.europa.eu/eurostat/databrowser/view/TPS00044/default/table?lang=en&category=hlth.hlth_care.hlth_res.hlth_staff%20%2F |
| | | |
| | **Proportion of health care expenditure on pharmaceuticals** | |
| | Data - % of health care expenditure | 13.033 |
| | Time | 2020 |
| | Reference | https://data.oecd.org/healthres/pharmaceutical-spending.htm |
| | | |
| | **Number of practising pharmacists per 100,000 inhabitants** | |
| | Data - N of practisisng pharmacists per 100,000 inhabitants | 94.56 |
| | Time | 2019 |
| | Reference | https://ec.europa.eu/eurostat/databrowser/view/HLTH_RS_PRS1__custom_4104351/default/table?lang=en |
| | | |
| | **Total health care expenditure as percentage of GDP** | |
| | Data - % of GDP | 8.09 |
| | Time | 2020 |

| | | |
|---|---|---|
| | Reference | https://ec.europa.eu/eurostat/databrowser/view/TPS00207/default/table?lang=en&category=hlth.hlth_care.hlth_sha11.hlth_sha11_sum |
| | | |
| | **Public pharmaceutical expenditure as percentage of total pharmaceutical expenditure** | |
| | Data - % of total pharmaceutical expenditure | 85.3 |
| | Time | 2020 |
| | Reference | https://gateway.euro.who.int/en/indicators/hfa_580-6790-public-pharmaceutical-expenditure-as-of-total-pharmaceutical-expenditure/visualizations/#id=19675&tab=table |
| | | |
| | **Self-reported consultations of a medical doctor*** | |
| | Data - No contact, % of population according to the number of consultations of a medical doctor in the past 4 weeks | 62.4 |
| | Data - 1 contact, % of population according to the number of consultations of a medical doctor in the past 4 weeks | 21.8 |
| | Data - 2 contacts, % of population according to the number of consultations of a medical doctor in the past 4 weeks | 11.3 |
| | Data - 3 or more contacts, % of population according to the number of consultations of a medical doctor in the past 4 weeks | 4.5 |
| | Time | 2019 |
| | Reference | https://ec.europa.eu/eurostat/databrowser/view/HLTH_EHIS_AM2U/default/table?lang=en&category=hlth.hlth_care.hlth_consult / |

*Medical doctors include generalist medical practitioners and specialist medical practitioners

# CZECHIA

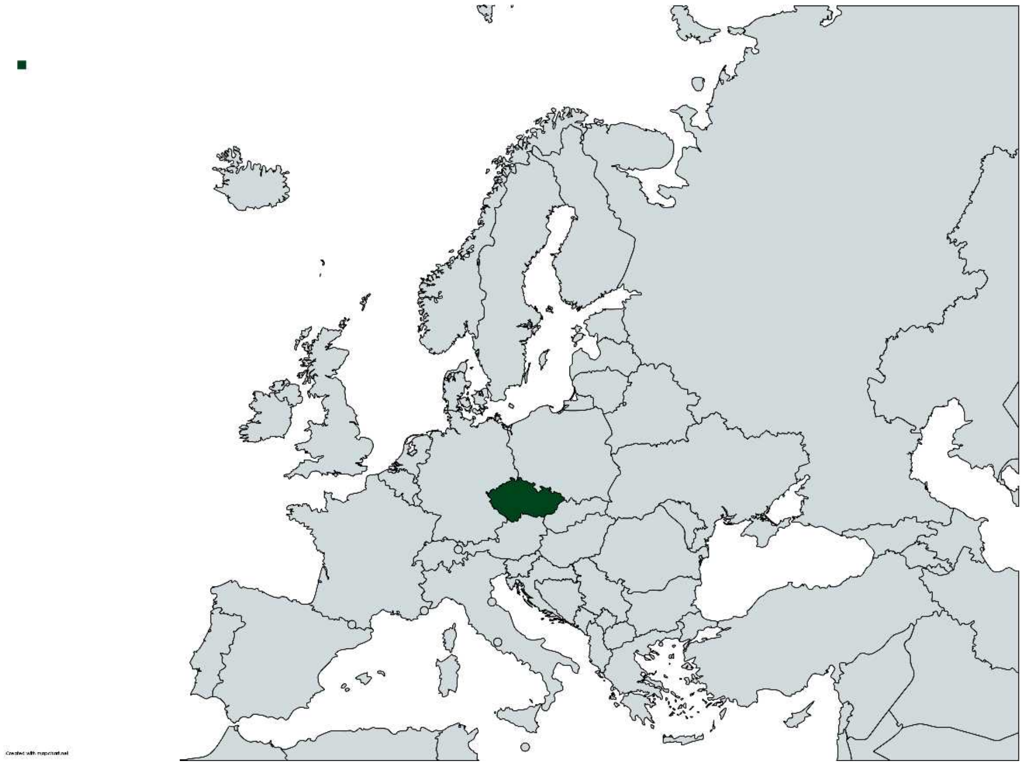

| Country | Czechia |
|---|---|

Country-specific data

| Country characteristics | Method of payment | |
|---|---|---|
| | Data | The patient only pays part of the total cost of a medicine and the sickness funds pay the rest directly to the pharmacy. |
| | Time | 2013 |
| | Reference | http://imi-protect.eu/documents/DUinventory_2013_COUNTRIESyear4_Dec2013.pdf |
| | | |
| | Medication adherence assessed and reported on the national level | |
| | Data | No |
| | Time | 2022 |
| | Reference | NA |
| | | |
| | Health care provider | |
| | Data | Health insurance is based on obligatory participation of insured person. It is provided by 7 health insurance funds. |
| | Time | 2023 |
| | Reference | https://kancelarzp.cz/usefull-links-info/health-insurance-system-in-cz/ |
| | | |
| | Model of healthcare system financing | |
| | Data | Mixed public-private budget: The financial participation of patients in overall costs of healthcare is about 17 % in average. It concerns medicaments and dental care above all. |
| | Time | 2023 |
| | Reference | https://kancelarzp.cz/usefull-links-info/health-insurance-system-in-cz |
| | | |
| | Proportion of population aged 65 years and over | |
| | Data - % of persons | 20.2 |
| | Time | 2021 |
| | Reference | https://ec.europa.eu/eurostat/databrowser/view/TPS00028/default/table?lang=en&category=demo.demo_ind / |
| | | |
| | Country population (projection) | |
| | Data - N of persons | 10693861 |
| | Time | 2020 |
| | Reference | https://ec.europa.eu/eurostat/databrowser/view/CENS_HNMGA/default/table?lang=en&category=cens.cens_hn.cens_hnstr |

| | | |
|---|---|---|
| Social/economic factors | **Patient co-payment** | |
| | Data | Copayment of a maximum ceiling of CZK5000 (~EUR200) per year on medicines. Above which, the sickness funds per a calendar year, the health insurance company is obliged to pay the insured person back the exceeding amount. The overall limit for children under the age of 18 and for pensioners 65-70 years old is 1.000 CZK per a calendar year. The limit for pensioners above 70 years of age is 500 CZK. |
| | Time | 2023 |
| | Reference | https://www.vzp.cz/o-nas/nejcasteji-resite/otazka/jake-jsou-rocni-ochranne-limity-u-doplatku-na-leky |
| | | |
| | **Percentage of prescriptions dispensed at no cost to patients** | |
| | Data - % of prescriptions | NA |
| | Time | NA |
| | Reference | NA |
| | | |
| | **Population coverage** | |
| | Data | Universal access |
| | Time | 2013 |
| | Reference | http://imi-protect.eu/documents/DUinventory_2013_COUNTRIESyear4_Dec2013.pdf |
| | | |
| | **Availability of doctors' services for citizens at no payment** | |
| | Data | Healthcare is provided to anyone insured in the Czech Republic who falls ill or requires medical care.<br>This includes EU nationals and their family members who are insured in Member States outside the Czech Republic. Persons working or residing in the Czech Republic are eligible for full healthcare. Those in the country for a short period of time (tourism or business) are provided necessary care in case of an illness or accident. |
| | Time | 2023 |
| | Reference | https://ec.europa.eu/social/main.jsp?catId=1106&intPageId=4473&langId=en |
| | | |
| Therapy-related factors | **Average number of medicines per patient** | |
| | Data - N of medicines per patient | NA |
| | Time | NA |
| | Reference | NA |
| | | |
| | **Proportion of 75 years and over who are taking more than 5 medications concurrently** | |
| | Data - % of persons | NA |
| | Time | NA |

| | | |
|---|---|---|
| | Reference | NA |
| | | |
| | **Percentage of self-reported use of prescribed medicines** | |
| | Data - % of persons | 51.1 |
| | Time | 2019 |
| | Reference | https://ec.europa.eu/eurostat/databrowser/view/HLTH_EHIS_MD1E__custom_3764895/default/table?lang=en / |

| | | |
|---|---|---|
| Patient-related factors | **Percentage of persons reporting a chronic disease** | |
| | Data - Asthma, % of persons | 4.6 |
| | Data - Chronic lower respiratory diseases, % of persons | 2.2 |
| | Data - High blood pressure, % of persons | 26.3 |
| | Data - Diabetes, % of persons | 8.8 |
| | Data - Chronic depression, % of persons | 4.4 |
| | Time | 2019 |
| | Reference | https://ec.europa.eu/eurostat/databrowser/view/HLTH_EHIS_CD1E/default/table?lang=en&category=hlth.hlth_state.hlth_srcm / |
| | | |
| | **Percentage of self-perceived health - very good (16 years and over)** | |
| | Data - % of persons | 26.8 |
| | Time | 2021 |
| | Reference | https://ec.europa.eu/eurostat/databrowser/view/HLTH_SILC_02/default/table?lang=en&category=hlth.hlth_state.hlth_sph/ |
| | | |
| | **Percenatge of persons with current depressive symptoms** | |
| | Data - % of persons | 4.2 |
| | Time | 2019 |
| | Reference | https://ec.europa.eu/eurostat/databrowser/view/HLTH_EHIS_MH1E/default/table?lang=en&category=hlth.hlth_state.hlth_sph / |

| | | |
|---|---|---|
| Condition-related factor[illegible] | **General health literacy** | |
| | Data - Inadequate health literacy, % of persons | 13 |
| | Data - Problematic health literacy, % of persons | 34 |
| | Data - Sufficient health literacy, % of persons | 43 |
| | Data - Excellent health literacy, % of persons | 10 |
| | Time | 2021 |
| | Reference | https://m-pohl.net/Int_Report_methdology_results_recommendations |

| | | |
|---|---|---|
| Healthcare system-relat | **Percentage of patients receiving adherence interventions** | |
| | Data - % of persons | NA |
| | Time | NA |
| | Reference | NA |
| | | |
| | **Nationwide availability of e-prescription** | |
| | Data | Yes |
| | Time | 2018 |
| | Reference | https://www.epreskripce.cz/about-prescription-0 |
| | | |
| | **Waiting time for prescriptions / medical appointments** | |
| | Data | NA |
| | Time | NA |
| | Reference | NA |
| | | |
| | **Number of practising physicians per 100,000 inhabitants** | |
| | Data - N of practisisng physicians per 100,000 inhabitants | 409.52 |
| | Time | 2020 |
| | Reference | https://ec.europa.eu/eurostat/databrowser/view/TPS00044/default/table?lang=en&category=hlth.hlth_care.hlth_res.hlth_staff%20%2F |
| | | |
| | **Proportion of health care expenditure on pharmaceuticals** | |
| | Data - % of health care expenditure | 15.234 |
| | Time | 2020 |
| | Reference | https://data.oecd.org/healthres/pharmaceutical-spending.htm |
| | | |
| | **Number of practising pharmacists per 100,000 inhabitants** | |
| | Data - N of practisisng pharmacists per 100,000 inhabitants | 70.99 |
| | Time | 2020 |
| | Reference | https://ec.europa.eu/eurostat/databrowser/view/HLTH_RS_PRS1__custom_4104351/default/table?lang=en |
| | | |
| | **Total health care expenditure as percentage of GDP** | |
| | Data - % of GDP | 9.24 |

| | | |
|---|---|---|
| | Time | 2020 |
| | Reference | https://ec.europa.eu/eurostat/databrowser/view/TPS00207/default/table?lang=en&category=hlth.hlth_care.hlth_sha11.hlth_sha11_sum |
| | | |
| | **Public pharmaceutical expenditure as percentage of total pharmaceutical expenditure** | |
| | Data - % of total pharmaceutical expenditure | 62.5 |
| | Time | 2011 |
| | Reference | https://gateway.euro.who.int/en/indicators/hfa_580-6790-public-pharmaceutical-expenditure-as-of-total-pharmaceutical-expenditure/visualizations/#id=19675&tab=table |
| | | |
| | **Self-reported consultations of a medical doctor*** | |
| | Data - No contact, % of population according to the number of consultations of a medical doctor in the past 4 weeks | 53.9 |
| | Data - 1 contact, % of population according to the number of consultations of a medical doctor in the past 4 weeks | 22.8 |
| | Data - 2 contacts, % of population according to the number of consultations of a medical doctor in the past 4 weeks | 12.3 |
| | Data - 3 or more contacts, % of population according to the number of consultations of a medical doctor in the past 4 weeks | 11 |
| | Time | 2019 |
| | Reference | https://ec.europa.eu/eurostat/databrowser/view/HLTH_EHIS_AM2U/default/table?lang=en&category=hlth.hlth_care.hlth_consult / |

*Medical doctors include generalist medical practitioners and specialist medical practitioners

DENMARK

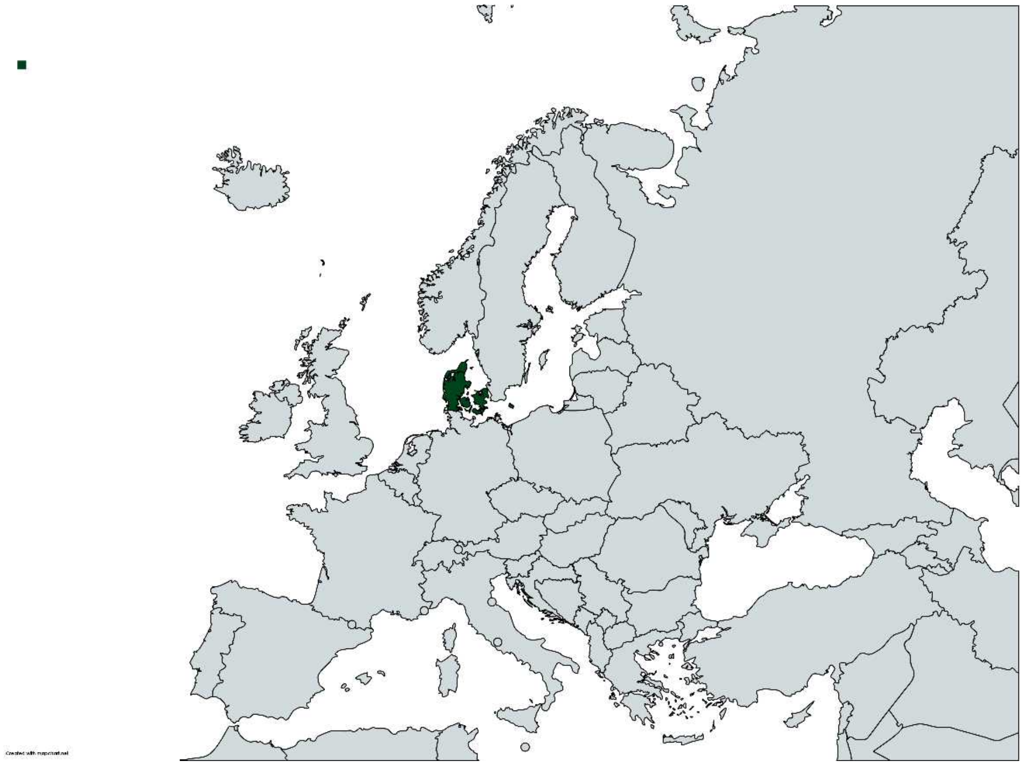

| Country | Denmark |
|---|---|

Country-specific data

| Country characteristics | | |
|---|---|---|
| | **Method of payment** | |
| | Data | The patient pays fully/partially the cost of the medicine and the municipality pays the remaining part to the pharmacist. |
| | Time | 2013 |
| | Reference | http://imi-protect.eu/documents/DUinventory_2013_COUNTRIESyear4_Dec2013.pdf |
| | | |
| | **Medication adherence assessed and reported on the national level** | |
| | Data | No |
| | Time | 2022 |
| | Reference | NA |
| | | |
| | **Health care provider** | |
| | Data | Universal compulsory system. No possibility of opting out of the system. Around 20% of Danish population have purchased additional voluntary health insurance from non-for-profit organizations. |
| | Time | 2013 |
| | Reference | http://imi-protect.eu/documents/DUinventory_2013_COUNTRIESyear4_Dec2013.pdf |
| | | |
| | **Model of healthcare system financing** | |
| | Data | Income taxes and block grants from the state finance municipalitites. Regions are financed by the state through income taxes, VAT, other taxes and by the municipalities. |
| | Time | 2013 |
| | Reference | http://imi-protect.eu/documents/DUinventory_2013_COUNTRIESyear4_Dec2013.pdf |
| | | |
| | **Proportion of population aged 65 years and over** | |
| | Data - % of persons | 20.1 |
| | Time | 2021 |
| | Reference | https://ec.europa.eu/eurostat/databrowser/view/TPS00028/default/table?lang=en&category=demo.demo_ind / |
| | | |
| | **Country population (projection)** | |
| | Data - N of persons | 5811651 |
| | Time | 2020 |

| | | |
|---|---|---|
| | Reference | https://ec.europa.eu/eurostat/databrowser/view/CENS_HNMGA/default/table?lang=en&category=cens.cens_hn.cens_hnstr |

| | | |
|---|---|---|
| Social/economic factors | **Patient co-payment** | |
| | Data | Positive list of medicines eligible for reimbursement. Copayment consits to full payment of medicines up to a maximum cost for a period of 12 months. Above this maximum, different percentages are paid by the patient depending on patient's health expenditure with compayment of 100%, 50%, 25%, 15% and 0%. |
| | Time | 2013 |
| | Reference | http://imi-protect.eu/documents/DUinventory_2013_COUNTRIESyear4_Dec2013.pdf |
| | | |
| | **Percentage of prescriptions dispensed at no cost to patients** | |
| | Data - % of prescriptions | NA |
| | Time | NA |
| | Reference | NA |
| | | |
| | **Population coverage** | |
| | Data | Universal access, non-residents are entitled acute treatment |
| | Time | 2013 |
| | Reference | http://imi-protect.eu/documents/DUinventory_2013_COUNTRIESyear4_Dec2013.pdf |
| | | |
| | **Availability of doctors' services for citizens at no payment** | |
| | Data | NA |
| | Time | NA |
| | Reference | NA |

| | | |
|---|---|---|
| Therapy-related factors | **Average number of medicines per patient** | |
| | Data - N of medicines per patient | NA |
| | Time | NA |
| | Reference | NA |
| | | |
| | **Proportion of 75 years and over who are taking more than 5 medications concurrently** | |
| | Data - % of persons | 22.1 |
| | Time | 2020 |
| | Reference | https://stats.oecd.org/Index.aspx?ThemeTreeId=17 |
| | | |

| | | |
|---|---|---|
| | **Percentage of self-reported use of prescribed medicines** | |
| | Data - % of persons | 48.9 |
| | Time | 2019 |
| | Reference | https://ec.europa.eu/eurostat/databrowser/view/HLTH_EHIS_MD1E__custom_3764895/default/table?lang=en / |

| | | |
|---|---|---|
| **Patient-related factors** | **Percentage of persons reporting a chronic disease** | |
| | Data - Asthma, % of persons | 7.2 |
| | Data - Chronic lower respiratory diseases, % of persons | 3.4 |
| | Data - High blood pressure, % of persons | 18.9 |
| | Data - Diabetes, % of persons | 5.3 |
| | Data - Chronic depression, % of persons | 10 |
| | Time | 2019 |
| | Reference | https://ec.europa.eu/eurostat/databrowser/view/HLTH_EHIS_CD1E/default/table?lang=en&category=hlth.hlth_state.hlth_srcm / |
| | | |
| | **Percentage of self-perceived health - very good (16 years and over)** | |
| | Data - % of persons | 19.5 |
| | Time | 2021 |
| | Reference | https://ec.europa.eu/eurostat/databrowser/view/HLTH_SILC_02/default/table?lang=en&category=hlth.hlth_state.hlth_sph/ |
| | | |
| | **Percenatge of persons with current depressive symptoms** | |
| | Data - % of persons | 8.3 |
| | Time | 2019 |
| | Reference | https://ec.europa.eu/eurostat/databrowser/view/HLTH_EHIS_MH1E/default/table?lang=en&category=hlth.hlth_state.hlth_sph / |

| | | |
|---|---|---|
| **Condition-related factor** | **General health literacy** | |
| | Data - Inadequate health literacy, % of persons | 11 |
| | Data - Problematic health literacy, % of persons | 36 |
| | Data - Sufficient health literacy, % of persons | 37 |
| | Data - Excellent health literacy, % of persons | 16 |
| | Time | 2021 |
| | Reference | https://m-pohl.net/Int_Report_methdology_results_recommendations |

| | | |
|---|---|---|
| **Healthcare system-relat** | **Percentage of patients receiving adherence interventions** | |

| | |
|---|---|
| Data - % of persons | NA |
| Time | NA |
| Reference | NA |
| | |
| **Nationwide availability of e-prescription** | |
| Data | NA |
| Time | NA |
| Reference | NA |
| | |
| **Waiting time for prescriptions / medical appointments** | |
| Data | NA |
| Time | NA |
| Reference | NA |
| | |
| **Number of practising physicians per 100,000 inhabitants** | |
| Data - N of practisisng physicians per 100,000 inhabitants | 425.06 |
| Time | 2019 |
| Reference | https://ec.europa.eu/eurostat/databrowser/view/TPS00044/default/table?lang=en&category=hlth.hlth_care.hlth_res.hlth_staff%20%2F |
| | |
| **Proportion of health care expenditure on pharmaceuticals** | |
| Data - % of health care expenditure | 5.971 |
| Time | 2021 |
| Reference | https://data.oecd.org/healthres/pharmaceutical-spending.htm |
| | |
| **Number of practising pharmacists per 100,000 inhabitants** | |
| Data - N of practisisng pharmacists per 100,000 inhabitants | 44.32 |
| Time | 2019 |
| Reference | https://ec.europa.eu/eurostat/databrowser/view/HLTH_RS_PRS1__custom_4104351/default/table?lang=en |
| | |
| **Total health care expenditure as percentage of GDP** | |
| Data - % of GDP | 10.53 |
| Time | 2020 |

| | | |
|---|---|---|
| | Reference | https://ec.europa.eu/eurostat/databrowser/view/TPS00207/default/table?lang=en&category=hlth.hlth_care.hlth_sha11.hlth_sha11_sum |
| | | |
| | **Public pharmaceutical expenditure as percentage of total pharmaceutical expenditure** | |
| | Data - % of total pharmaceutical expenditure | 46 |
| | Time | 2012 |
| | Reference | https://gateway.euro.who.int/en/indicators/hfa_580-6790-public-pharmaceutical-expenditure-as-of-total-pharmaceutical-expenditure/visualizations/#id=19675&tab=table |
| | | |
| | **Self-reported consultations of a medical doctor*** | |
| | Data - No contact, % of population according to the number of consultations of a medical doctor in the past 4 weeks | 58.1 |
| | Data - 1 contact, % of population according to the number of consultations of a medical doctor in the past 4 weeks | 22.2 |
| | Data - 2 contacts, % of population according to the number of consultations of a medical doctor in the past 4 weeks | 10.9 |
| | Data - 3 or more contacts, % of population according to the number of consultations of a medical doctor in the past 4 weeks | 8.8 |
| | Time | 2019 |
| | Reference | https://ec.europa.eu/eurostat/databrowser/view/HLTH_EHIS_AM2U/default/table?lang=en&category=hlth.hlth_care.hlth_consult / |

*Medical doctors include generalist medical practitioners and specialist medical practitioners

ESTONIA

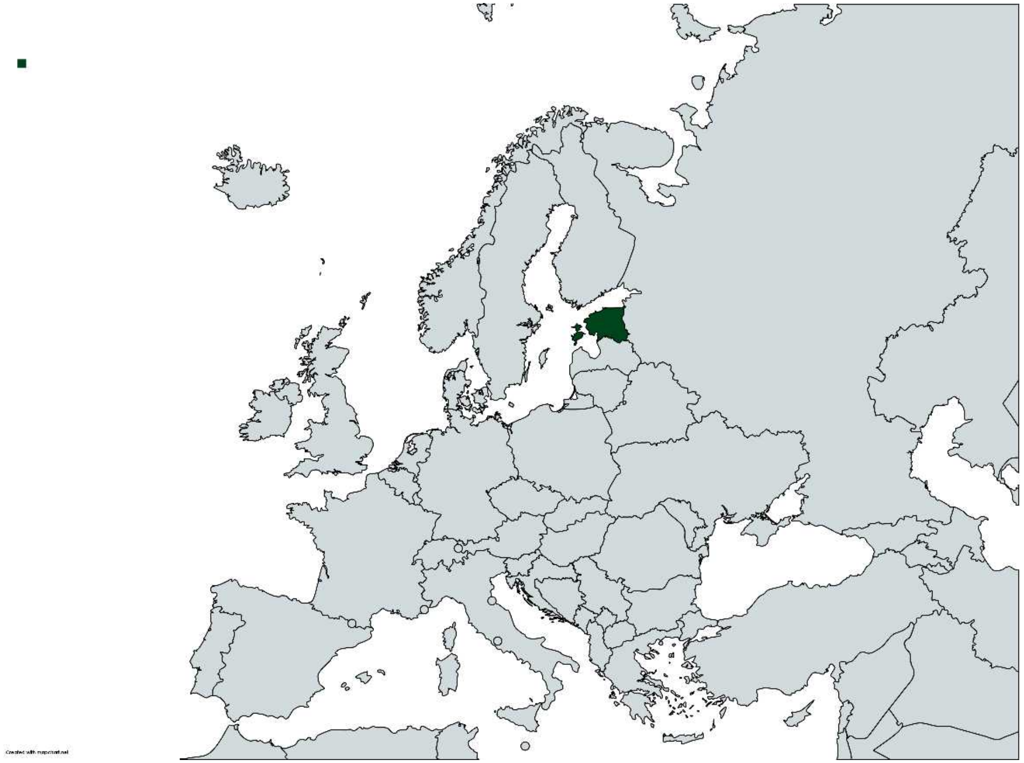

| Country | Estonia |
|---|---|

Country-specific data

| Country characteristics | Method of payment | |
|---|---|---|
| | Data | The patient partially pays the cost of the medicine and the Estonian Health Insurance Fund (EHIF) pays the rest. |
| | Time | 2013 |
| | Reference | http://imi-protect.eu/documents/DUinventory_2013_COUNTRIESyear4_Dec2013.pdf |
| | | |
| | Medication adherence assessed and reported on the national level | |
| | Data | No |
| | Time | 2022 |
| | Reference | NA |
| | | |
| | Health care provider | |
| | Data | The National Health Service supervised by the Ministry of Social Affairs. |
| | Time | 2013 |
| | Reference | http://imi-protect.eu/documents/DUinventory_2013_COUNTRIESyear4_Dec2013.pdf |
| | | |
| | Model of healthcare system financing | |
| | Data | Estonia has a centralised health system with a single health insurance fund. The Estonian health system is largely funded through payroll tax. The Estonian Health Insurance Fund (EHIF) operates as a semi-autonomous public organisation, pooling most of the public funding for health and organising the purchasing of health care. The Ministry of Social Affairs oversees the Estonian health system |
| | Time | 2021 |
| | Reference | Estonia health system information (who.int) |
| | | |
| | Proportion of population aged 65 years and over | |
| | Data - % of persons | 20.3 |
| | Time | 2021 |
| | Reference | https://ec.europa.eu/eurostat/databrowser/view/TPS00028/default/table?lang=en&category=demo.demo_ind / |
| | | |
| | Country population (projection) | |
| | Data - N of persons | 1357739 |

| | | |
|---|---|---|
| | Time | 2023 |
| | Reference | Avaleht \| Statistikaamet |

| | | |
|---|---|---|
| **Social/economic factors** | **Patient co-payment** | |
| | Data | EHIF reimburses to a certain extent for prescription medicines, the effectiveness of which has been previously thoroughly assessed and therefore they have been included in the list of reimbursed pharmaceuticals. These pharmaceuticals are available at a 50, 75, 90 or 100% discount. The highest discount rates are available for principal pharmaceuticals needed to treat serious and chronic diseases, or for certain groups of the population (old-age and incapacity pensioners).<br>The buyer has to pay a prescription fee of 2.5 euros for each prescription medicine.<br>The EHIF discount is calculated on the remaining part of the price according to the prescribed percentage. This means that the patient has to pay for the part exceeding the discount amount.<br>If there are many pharmaceuticals with the same active ingredient by different manufacturers on the market, a reference price is set for the whole group of pharmaceuticals with the same active ingredient, and the EHIF discount is calculated on this reference price. For example, if a pharmacy has a selection of medicines with the same active ingredient at the price of 5.60, 8.30, 10.50 and 12.80, the price of the second cheapest medicine (8.30 in this example) is used as the reference price and the EHIF discount is calculated on this amount. If the buyer prefers a more expensive medicine, then in addition to the basic rate (2.5 euros) and mandatory contribution, he/she must also pay for the part exceeding the reference price. Therefore, it is worth asking your pharmacist which medicine with the same active ingredient is sold at better price. |
| | Time | 2023 |
| | Reference | Pharmaceuticals \| Estonian Health Insurance Fund (haigekassa.ee) |
| | | |
| | **Percentage of prescriptions dispensed at no cost to patients** | |
| | Data - % of prescriptions | Despite on discount % there is still a prescription fee EUR 2.50 per prescription |
| | Time | 2023 |
| | Reference | Pharmaceuticals \| Estonian Health Insurance Fund (haigekassa.ee) |
| | | |
| | **Population coverage** | |
| | Data | Universal access |
| | Time | 2013 |
| | Reference | http://imi-protect.eu/documents/DUinventory_2013_COUNTRIESyear4_Dec2013.pdf |
| | | |
| | **Availability of doctors' services for citizens at no payment** | |
| | Data | The EHIF defines the benefits package and price list for health services, pharmaceuticals and medical products. The price list contains over 2 000 items with a range of payment mechanisms, and is updated at least once per year. The EHIF covers most hospital and outpatient medical care, with pharmaceuticals, dental care and therapeutic appliances often using elements of cost-sharing |
| | Time | 2021 |

| | | |
|---|---|---|
| | Reference | Estonia health system information (who.int) |
| | | |
| Therapy-related factors | **Average number of medicines per patient** | |
| | Data - N of medicines per patient | NA |
| | Time | NA |
| | Reference | NA |
| | | |
| | **Proportion of 75 years and over who are taking more than 5 medications concurrently** | |
| | Data - % of persons | 26.3 |
| | Time | 2020 |
| | Reference | https://stats.oecd.org/Index.aspx?ThemeTreeId=18 |
| | | |
| | **Percentage of self-reported use of prescribed medicines** | |
| | Data - % of persons | 45.8 |
| | Time | 2019 |
| | Reference | https://ec.europa.eu/eurostat/databrowser/view/HLTH_EHIS_MD1E__custom_3764895/default/table?lang=en / |

| | | |
|---|---|---|
| Patient-related factors | **Percentage of persons reporting a chronic disease** | |
| | Data - Asthma, % of persons | 4.1 |
| | Data - Chronic lower respiratory diseases, % of persons | 2.5 |
| | Data - High blood pressure, % of persons | 23.3 |
| | Data - Diabetes, % of persons | 6 |
| | Data - Chronic depression, % of persons | 7 |
| | Time | 2019 |
| | Reference | https://ec.europa.eu/eurostat/databrowser/view/HLTH_EHIS_CD1E/default/table?lang=en&category=hlth.hlth_state.hlth_srcm / |
| | | |
| | **Percentage of self-perceived health - very good (16 years and over)** | |
| | Data - % of persons | 13.3 |
| | Time | 2021 |
| | Reference | https://ec.europa.eu/eurostat/databrowser/view/HLTH_SILC_02/default/table?lang=en&category=hlth.hlth_state.hlth_sph/ |
| | | |
| | **Percenatge of persons with current depressive symptoms** | |
| | Data - % of persons | 8.2 |

| | | |
|---|---|---|
| | Time | 2019 |
| | Reference | https://ec.europa.eu/eurostat/databrowser/view/HLTH_EHIS_MH1E/default/table?lang=en&category=hlth.hlth_state.hlth_sph / |

| | | |
|---|---|---|
| Condition-related factor: | **General health literacy** | |
| | Data - Inadequate health literacy, % of persons | NA |
| | Data - Problematic health literacy, % of persons | NA |
| | Data - Sufficient health literacy, % of persons | NA |
| | Data - Excellent health literacy, % of persons | NA |
| | Time | NA |
| | Reference | NA |

| | | |
|---|---|---|
| Healthcare system-relat | **Percentage of patients receiving adherence interventions** | |
| | Data - % of persons | NA |
| | Time | NA |
| | Reference | NA |
| | | |
| | **Nationwide availability of e-prescription** | |
| | Data | Yes |
| | Time | 2019 |
| | Reference | Estonian e-Prescription – e-estoniax.com, https://e-estonia.com/first-eu-citizens-using-eprescriptions-in-other-eu-country/ |
| | | |
| | **Waiting time for prescriptions / medical appointments** | |
| | Data | Estonians report a high level of unmet needs for medical care, mainly due to waiting times More people in Estonia reported that their health needs were not met than in any other EU country in 2019, with 15.5 % experiencing unmet needs for medical care compared to an EU average of 1.7 % |
| | Time | 2021 |
| | Reference | Estonia health system information (who.int) |
| | | |
| | **Number of practising physicians per 100,000 inhabitants** | |
| | Data - N of practisisng physicians per 100,000 inhabitants | 347.94 |
| | Time | 2020 |
| | Reference | https://ec.europa.eu/eurostat/databrowser/view/TPS00044/default/table?lang=en&category=hlth.hlth_care.hlth_res.hlth_staff%20%2F |
| | | |

| | |
|---|---|
| **Proportion of health care expenditure on pharmaceuticals** | |
| Data - % of health care expenditure | 17.625 |
| Time | 2020 |
| Reference | https://data.oecd.org/healthres/pharmaceutical-spending.htm |
| | |
| **Number of practising pharmacists per 100,000 inhabitants** | |
| Data - N of practisisng pharmacists per 100,000 inhabitants | 72.81 |
| Time | 2020 |
| Reference | https://ec.europa.eu/eurostat/databrowser/view/HLTH_RS_PRS1__custom_4104351/default/table?lang=en |
| | |
| **Total health care expenditure as percentage of GDP** | |
| Data - % of GDP | 7.75 |
| Time | 2020 |
| Reference | https://ec.europa.eu/eurostat/databrowser/view/TPS00207/default/table?lang=en&category=hlth.hlth_care.hlth_sha11.hlth_sha11_sum |
| | |
| **Public pharmaceutical expenditure as percentage of total pharmaceutical expenditure** | |
| Data - % of total pharmaceutical expenditure | 53.8 |
| Time | 2020 |
| Reference | https://gateway.euro.who.int/en/indicators/hfa_580-6790-public-pharmaceutical-expenditure-as-of-total-pharmaceutical-expenditure/visualizations/#id=19675&tab=table |
| | |
| **Self-reported consultations of a medical doctor*** | |
| Data - No contact, % of population according to the number of consultations of a medical doctor in the past 4 weeks | 67.8 |
| Data - 1 contact, % of population according to the number of consultations of a medical doctor in the past 4 weeks | 16 |
| Data - 2 contacts, % of population according to the number of consultations of a medical doctor in the past 4 weeks | 7.5 |
| Data - 3 or more contacts, % of population according to the number of consultations of a medical doctor in the past 4 weeks | 8.6 |
| Time | 2019 |
| Reference | https://ec.europa.eu/eurostat/databrowser/view/HLTH_EHIS_AM2U/default/table?lang=en&category=hlth.hlth_care.hlth_consult / |

FINLAND

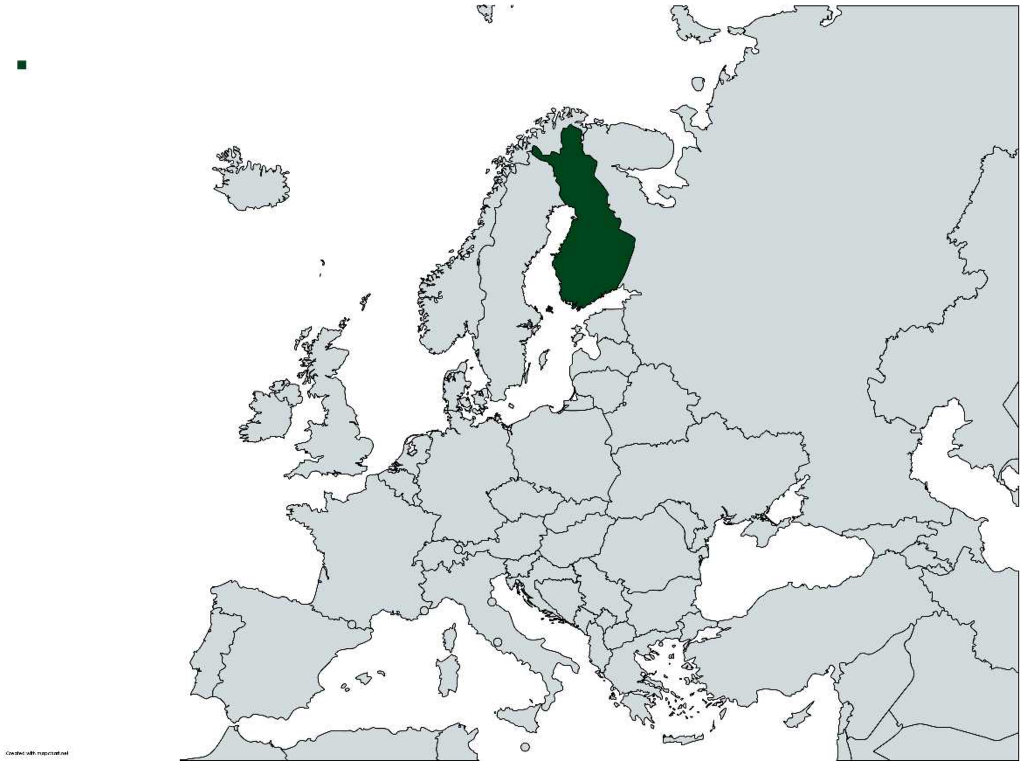

| Country | Finland |
|---|---|

Country-specific data

| Country characteristics | Method of payment | |
|---|---|---|
| | Data | The amount the patient pays for the medicine depends on the reimbursement status of the medicine, what the patient has paid for reimbursable medicines in the calendar year, and if the patient is eligible for special rate of reimbursement. The Social Insurance Institution (KELA) pays the remaining part to the pharmacy. |
| | Time | 2022 |
| | Reference | Finnish statistics on medicines 2021 https://urn.fi/URN:NBN:fi-fe2022121672024 |
| | | |
| | **Medication adherence assessed and reported on the national level** | |
| | Data | No |
| | Time | 2022 |
| | Reference | NA |
| | | |
| | **Health care provider** | |
| | Data | Universal access to health care. Three different sectors: public, private and occupational health care. |
| | Time | 2022 |
| | Reference | https://stm.fi/en/health-services |
| | | |
| | **Model of healthcare system financing** | |
| | Data | Municipal financing based on taxes and National Health Insurance based on compulsory insurance fees (sickness and income insurrance). Private health care system: there are voluntary health insurances. Statutory motor accident and occupational accident, both compulsory. |
| | Time | 2013 |
| | Reference | http://imi-protect.eu/documents/DUinventory_2013_COUNTRIESyear4_Dec2013.pdf |
| | | |
| | **Proportion of population aged 65 years and over** | |
| | Data - % of persons | 22.7 |
| | Time | 2021 |
| | Reference | https://ec.europa.eu/eurostat/databrowser/view/TPS00028/default/table?lang=en&category=demo.demo_ind / |
| | | |
| | **Country population (projection)** | |
| | Data - N of persons | 5563695 |

| | | |
|---|---|---|
| | Time | 2022 |
| | Reference | https://www.stat.fi/index_en.html |
| | | |
| Social/economic factors | **Patient co-payment** | |
| | Data | For reimbursable medicines, there is an initial deductible of 50 euros (persons at least 19 years old). After reaching the initial deductible limit, the patient will receive reimbursement depending on the reimbursement status of the medicine. The patient will pay 35 % (basic rate) or 65 % (lower special rate) of the medicine's price or 4.50 euros (higher special rate). If the patient reaches during a calendar year the annual maximum limit for reimbursable medicines (updated yearly, 592.16 euros in 2023), the patient will pay 2.50 euros for each reimbursable medicine after exceeding the limit. Non-reimbursable medicines are paid by patients and are not counted for the limits. |
| | Time | 2021 |
| | Reference | https://urn.fi/URN:NBN:fi-fe2022121672024 |
| | | |
| | **Percentage of prescriptions dispensed at no cost to patients** | |
| | Data - % of prescriptions | NA |
| | Time | NA |
| | Reference | NA |
| | | |
| | **Population coverage** | |
| | Data | Public health care covers all permanent residents. |
| | Time | 2022 |
| | Reference | https://stm.fi/en/health-services |
| | | |
| | **Availability of doctors' services for citizens at no payment** | |
| | Data | Services free for children (under 18 years). |
| | Time | 2023 |
| | Reference | https://www.eu-healthcare.fi/what-you-pay/costs-of-treatment-in-finland/treatment-costs-in-public-health-care/ |
| | | |
| Therapy-related factors | **Average number of medicines per patient** | |
| | Data - N of medicines per patient | NA |
| | Time | NA |
| | Reference | NA |
| | | |
| | **Proportion of 75 years and over who are taking more than 5 medications concurrently** | |
| | Data - % of persons | 27.9 |

| | | |
|---|---|---|
| | Time | 2020 |
| | Reference | https://stats.oecd.org/Index.aspx?ThemeTreeId=19 |
| | | |
| | **Percentage of self-reported use of prescribed medicines** | |
| | Data - % of persons | 59 |
| | Time | 2019 |
| | Reference | https://ec.europa.eu/eurostat/databrowser/view/HLTH_EHIS_MD1E__custom_3764895/default/table?lang=en / |

| | | |
|---|---|---|
| **Patient-related factors** | **Percentage of persons reporting a chronic disease** | |
| | Data - Asthma, % of persons | 9.4 |
| | Data - Chronic lower respiratory diseases, % of persons | 3.3 |
| | Data - High blood pressure, % of persons | 27.3 |
| | Data - Diabetes, % of persons | 9.5 |
| | Data - Chronic depression, % of persons | 8.8 |
| | Time | 2019 |
| | Reference | https://ec.europa.eu/eurostat/databrowser/view/HLTH_EHIS_CD1E/default/table?lang=en&category=hlth.hlth_state.hlth_srcm / |
| | | |
| | **Percentage of self-perceived health - very good (16 years and over)** | |
| | Data - % of persons | 19.4 |
| | Time | 2021 |
| | Reference | https://ec.europa.eu/eurostat/databrowser/view/HLTH_SILC_02/default/table?lang=en&category=hlth.hlth_state.hlth_sph/ |
| | | |
| | **Percenatge of persons with current depressive symptoms** | |
| | Data - % of persons | 6.5 |
| | Time | 2019 |
| | Reference | https://ec.europa.eu/eurostat/databrowser/view/HLTH_EHIS_MH1E/default/table?lang=en&category=hlth.hlth_state.hlth_sph / |

| | | |
|---|---|---|
| **Condition-related factor** | **General health literacy** | |
| | Data - Inadequate health literacy, % of persons | NA |
| | Data - Problematic health literacy, % of persons | NA |
| | Data - Sufficient health literacy, % of persons | NA |
| | Data - Excellent health literacy, % of persons | NA |
| | Time | NA |

| | | |
|---|---|---|
| | Reference | NA |

| | | |
|---|---|---|
| **Healthcare system-relat** | **Percentage of patients receiving adherence interventions** | |
| | Data - % of persons | NA |
| | Time | NA |
| | Reference | NA |
| | | |
| | **Nationwide availability of e-prescription** | |
| | Data | Yes |
| | Time | 2023 |
| | Reference | https://www.kanta.fi/en/prescriptions |
| | | |
| | **Waiting time for prescriptions / medical appointments** | |
| | Data | From the assessment of patient's need of care, 56 % of patients got to see a physician within one week in primary care (non-urgent visits). 11 % got to see a physician in 8-14 days, 19 % in 15-30 days, 13 % in 31-90 days, and 1 % in more than 90 days. |
| | Time | October 2022 |
| | Reference | https://urn.fi/URN:NBN:fi-fe2022121672074 (only in Finnish) |
| | | |
| | **Number of practising physicians per 100,000 inhabitants** | |
| | Data - N of practisisng physicians per 100,000 inhabitants | 348.02 |
| | Time | 2018 |
| | Reference | https://ec.europa.eu/eurostat/databrowser/view/TPS00044/default/table?lang=en&category=hlth.hlth_care.hlth_res.hlth_staff%20%2F |
| | | |
| | **Proportion of health care expenditure on pharmaceuticals** | |
| | Data - % of health care expenditure | 12.2 |
| | Time | 2020 |
| | Reference | https://data.oecd.org/healthres/pharmaceutical-spending.htm |
| | | |
| | **Number of practising pharmacists per 100,000 inhabitants** | |
| | Data - N of practisisng pharmacists per 100,000 inhabitants | 103.2 |
| | Time | 2018 |
| | Reference | https://ec.europa.eu/eurostat/databrowser/view/HLTH_RS_PRS1__custom_4104351/default/table?lang=en |
| | | |

| | | |
|---|---|---|
| | **Total health care expenditure as percentage of GDP** | |
| | Data - % of GDP | 9.61 |
| | Time | 2020 |
| | Reference | https://ec.europa.eu/eurostat/databrowser/view/TPS00207/default/table?lang=en&category=hlth.hlth_care.hlth_sha11.hlth_sha11_sum |
| | | |
| | **Public pharmaceutical expenditure as percentage of total pharmaceutical expenditure** | |
| | Data - % of total pharmaceutical expenditure | 55.9 |
| | Time | 2011 |
| | Reference | https://gateway.euro.who.int/en/indicators/hfa_580-6790-public-pharmaceutical-expenditure-as-of-total-pharmaceutical-expenditure/visualizations/#id=19675&tab=table |
| | | |
| | **Self-reported consultations of a medical doctor*** | |
| | Data - No contact, % of population according to the number of consultations of a medical doctor in the past 4 weeks | 72.2 |
| | Data - 1 contact, % of population according to the number of consultations of a medical doctor in the past 4 weeks | 16.1 |
| | Data - 2 contacts, % of population according to the number of consultations of a medical doctor in the past 4 weeks | 6.7 |
| | Data - 3 or more contacts, % of population according to the number of consultations of a medical doctor in the past 4 weeks | 5.1 |
| | Time | 2019 |
| | Reference | https://ec.europa.eu/eurostat/databrowser/view/HLTH_EHIS_AM2U/default/table?lang=en&category=hlth.hlth_care.hlth_consult / |

*Medical doctors include generalist medical practitioners and specialist medical practitioners

FRANCE

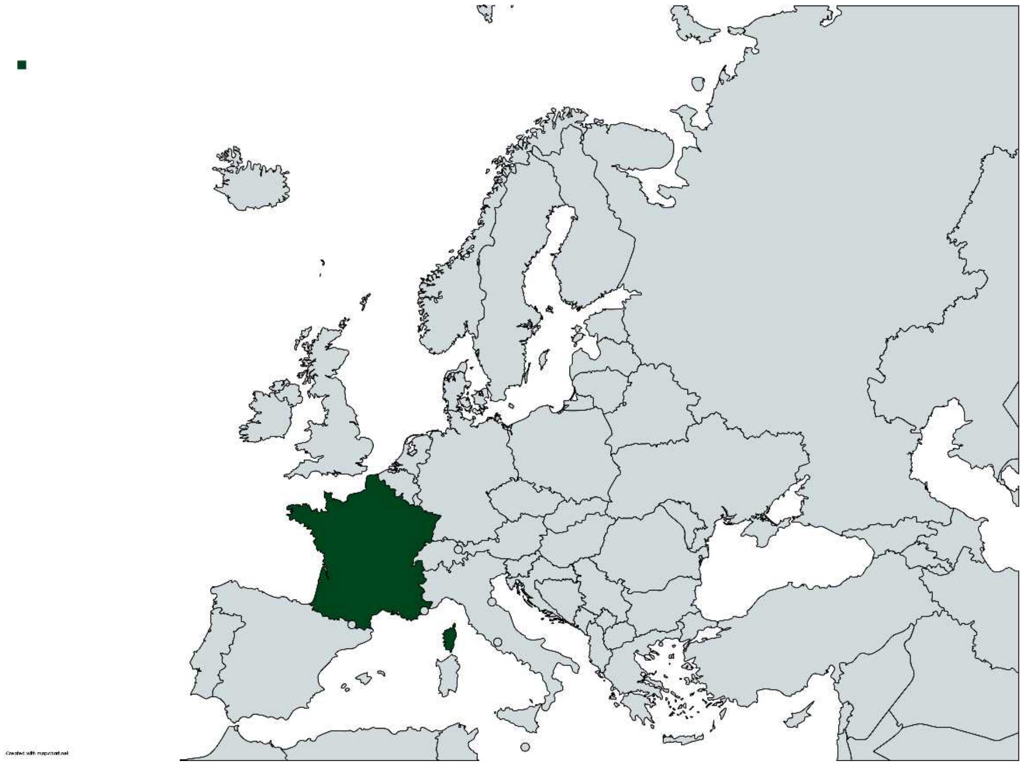

| Country | France |
|---|---|

Country-specific data

| Country characteristics | Method of payment | |
|---|---|---|
| | Data | The patient partially pays the cost of the medicines and the health insurer pays the rest to the pharmacist. |
| | Time | 2013 |
| | Reference | http://imi-protect.eu/documents/DUinventory_2013_COUNTRIESyear4_Dec2013.pdf |
| | | |
| | Medication adherence assessed and reported on the national level | |
| | Data | No |
| | Time | 2022 |
| | Reference | NA |
| | | |
| | Health care provider | |
| | Data | Public through Statutory Health Insurance System. Univrsal acces to health care services. |
| | Time | 2013 |
| | Reference | http://imi-protect.eu/documents/DUinventory_2013_COUNTRIESyear4_Dec2013.pdf |
| | | |
| | Model of healthcare system financing | |
| | Data | Mixed public and private financing. The Statutory Health Insurance is responsible of 73.8% of French healthcare expenditure; 5.2% is financed b ythe Sate; and 20.2% by private sources. Initially the SHI was financed by the meployer and the employee paroll taxes and other national taxes, currently, anyone can opt in to the system and pay a contribution to the SHI. In addition, 88% of population has a private for profit complementayr voluntary health insurance. |
| | Time | 2013 |
| | Reference | http://imi-protect.eu/documents/DUinventory_2013_COUNTRIESyear4_Dec2013.pdf |
| | | |
| | Proportion of population aged 65 years and over | |
| | Data - % of persons | 20.7 |
| | Time | 2021 |
| | Reference | https://ec.europa.eu/eurostat/databrowser/view/TPS00028/default/table?lang=en&category=demo.demo_ind / |
| | | |
| | Country population (projection) | |
| | Data - N of persons | 67197367 |

| | | |
|---|---|---|
| | Time | 2020 |
| | Reference | https://ec.europa.eu/eurostat/databrowser/view/CENS_HNMGA/default/table?lang=en&category=cens.cens_hn.cens_hnstr |

| | | |
|---|---|---|
| **Social/economic factors** | **Patient co-payment** | |
| | Data | The patient pays a flat fee of EUR0.50 per package plus a copayment of 85%, 70%, 35% and 0% depending of the therapeutic value of the medicine. |
| | Time | 2013 |
| | Reference | http://imi-protect.eu/documents/DUinventory_2013_COUNTRIESyear4_Dec2013.pdf |
| | | |
| | **Percentage of prescriptions dispensed at no cost to patients** | |
| | Data - % of prescriptions | NA |
| | Time | NA |
| | Reference | NA |
| | | |
| | **Population coverage** | |
| | Data | Statutory healthinsurance (SHI) provided by mutual associations which cover 99.9% of the French residents. People with low income and non-residents are covered by the state medical help. |
| | Time | 2013 |
| | Reference | http://imi-protect.eu/documents/DUinventory_2013_COUNTRIESyear4_Dec2013.pdf |
| | | |
| | **Availability of doctors' services for citizens at no payment** | |
| | Data | NA |
| | Time | NA |
| | Reference | NA |

| | | |
|---|---|---|
| **Therapy-related factors** | **Average number of medicines per patient** | |
| | Data - N of medicines per patient | NA |
| | Time | NA |
| | Reference | NA |
| | | |
| | **Proportion of 75 years and over who are taking more than 5 medications concurrently** | |
| | Data - % of persons | NA |
| | Time | NA |
| | Reference | NA |

| | | |
|---|---|---|
| | | |
| | **Percentage of self-reported use of prescribed medicines** | |
| | Data - % of persons | 49.4 |
| | Time | 2019 |
| | Reference | https://ec.europa.eu/eurostat/databrowser/view/HLTH_EHIS_MD1E__custom_3764895/default/table?lang=en / |

| | | |
|---|---|---|
| Patient-related factors | **Percentage of persons reporting a chronic disease** | |
| | Data - Asthma, % of persons | 7.8 |
| | Data - Chronic lower respiratory diseases, % of persons | 6.3 |
| | Data - High blood pressure, % of persons | 16.5 |
| | Data - Diabetes, % of persons | 7.3 |
| | Data - Chronic depression, % of persons | 7.7 |
| | Time | 2019 |
| | Reference | https://ec.europa.eu/eurostat/databrowser/view/HLTH_EHIS_CD1E/default/table?lang=en&category=hlth.hlth_state.hlth_srcm / |
| | | |
| | **Percentage of self-perceived health - very good (16 years and over)** | |
| | Data - % of persons | 27.6 |
| | Time | 2021 |
| | Reference | https://ec.europa.eu/eurostat/databrowser/view/HLTH_SILC_02/default/table?lang=en&category=hlth.hlth_state.hlth_sph/ |
| | | |
| | **Percenatge of persons with current depressive symptoms** | |
| | Data - % of persons | 10.8 |
| | Time | 2019 |
| | Reference | https://ec.europa.eu/eurostat/databrowser/view/HLTH_EHIS_MH1E/default/table?lang=en&category=hlth.hlth_state.hlth_sph / |

| | | |
|---|---|---|
| Condition-related factors | **General health literacy** | |
| | Data - Inadequate health literacy, % of persons | 14 |
| | Data - Problematic health literacy, % of persons | 30 |
| | Data - Sufficient health literacy, % of persons | 40 |
| | Data - Excellent health literacy, % of persons | 16 |
| | Time | 2021 |
| | Reference | https://m-pohl.net/Int_Report_methdology_results_recommendations |

| | | |
|---|---|---|
| Healthcare system-relat | **Percentage of patients receiving adherence interventions** | |
| | Data - % of persons | NA |
| | Time | NA |
| | Reference | NA |
| | | |
| | **Nationwide availability of e-prescription** | |
| | Data | NA |
| | Time | NA |
| | Reference | NA |
| | | |
| | **Waiting time for prescriptions / medical appointments** | |
| | Data | NA |
| | Time | NA |
| | Reference | NA |
| | | |
| | **Number of practising physicians per 100,000 inhabitants** | |
| | Data - N of practisisng physicians per 100,000 inhabitants | 318.07 |
| | Time | 2020 |
| | Reference | https://ec.europa.eu/eurostat/databrowser/view/TPS00044/default/table?lang=en&category=hlth.hlth_care.hlth_res.hlth_staff%20%2F |
| | | |
| | **Proportion of health care expenditure on pharmaceuticals** | |
| | Data - % of health care expenditure | 13.28 |
| | Time | 2020 |
| | Reference | https://data.oecd.org/healthres/pharmaceutical-spending.htm |
| | | |
| | **Number of practising pharmacists per 100,000 inhabitants** | |
| | Data - N of practisisng pharmacists per 100,000 inhabitants | 101.84 |
| | Time | 2020 |
| | Reference | https://ec.europa.eu/eurostat/databrowser/view/HLTH_RS_PRS1__custom_4104351/default/table?lang=en |
| | | |
| | **Total health care expenditure as percentage of GDP** | |
| | Data - % of GDP | 12.16 |
| | Time | 2020 |

| | | |
|---|---|---|
| | Reference | https://ec.europa.eu/eurostat/databrowser/view/TPS00207/default/table?lang=en&category=hlth.hlth_care.hlth_sha11.hlth_sha11_sum |
| | | |
| | **Public pharmaceutical expenditure as percentage of total pharmaceutical expenditure** | |
| | Data - % of total pharmaceutical expenditure | 68 |
| | Time | 2011 |
| | Reference | https://gateway.euro.who.int/en/indicators/hfa_580-6790-public-pharmaceutical-expenditure-as-of-total-pharmaceutical-expenditure/visualizations/#id=19675&tab=table |
| | | |
| | **Self-reported consultations of a medical doctor*** | |
| | Data - No contact, % of population according to the number of consultations of a medical doctor in the past 4 weeks | 58.9 |
| | Data - 1 contact, % of population according to the number of consultations of a medical doctor in the past 4 weeks | 26.5 |
| | Data - 2 contacts, % of population according to the number of consultations of a medical doctor in the past 4 weeks | 9.2 |
| | Data - 3 or more contacts, % of population according to the number of consultations of a medical doctor in the past 4 weeks | 5.4 |
| | Time | 2019 |
| | Reference | https://ec.europa.eu/eurostat/databrowser/view/HLTH_EHIS_AM2U/default/table?lang=en&category=hlth.hlth_care.hlth_consult / |

*Medical doctors include generalist medical practitioners and specialist medical practitioners

GERMANY

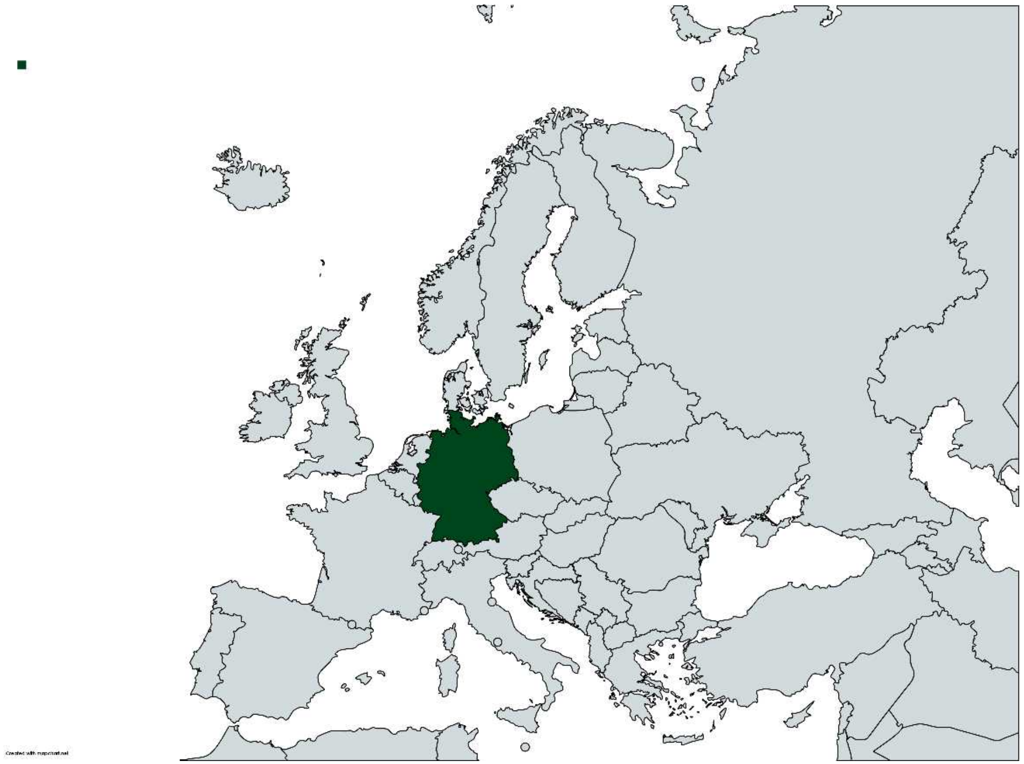

| Country | Germany |
|---|---|

Country-specific data

| Country characteristics | Method of payment | |
|---|---|---|
| | Data | The Statutory Health Insurance directly pays cost of drugs to the pharmacist. |
| | Time | 2013 |
| | Reference | http://imi-protect.eu/documents/DUinventory_2013_COUNTRIESyear4_Dec2013.pdf |
| | | |
| | Medication adherence assessed and reported on the national level | |
| | Data | No |
| | Time | 2022 |
| | Reference | NA |
| | | |
| | Health care provider | |
| | Data | Public Statutory Health Insurance (SHI) System, compulsory for all Geman citizens. Universal access to health care services. |
| | Time | 2013 |
| | Reference | http://imi-protect.eu/documents/DUinventory_2013_COUNTRIESyear4_Dec2013.pdf |
| | | |
| | Model of healthcare system financing | |
| | Data | Uniform wage-related contribution plus taxes. |
| | Time | 2013 |
| | Reference | http://imi-protect.eu/documents/DUinventory_2013_COUNTRIESyear4_Dec2013.pdf |
| | | |
| | Proportion of population aged 65 years and over | |
| | Data - % of persons | 22 |
| | Time | 2021 |
| | Reference | https://ec.europa.eu/eurostat/databrowser/view/TPS00028/default/table?lang=en&category=demo.demo_ind / |
| | | |
| | Country population (projection) | |
| | Data - N of persons | 83135181 |
| | Time | 2020 |
| | Reference | https://ec.europa.eu/eurostat/databrowser/view/CENS_HNMGA/default/table?lang=en&category=cens.cens_hn.cens_hnstr |

| | | |
|---|---|---|
| Social/economic factors | **Patient co-payment** | |
| | Data | The patient pays 10% of the price of medicines with a minimum of EUR5 and amaximum of EUR10 per prescription up to an annual upper limit based on patient's income. Exception: for those drugs with a price below 30% of the reference price. |
| | Time | 2013 |
| | Reference | http://imi-protect.eu/documents/DUinventory_2013_COUNTRIESyear4_Dec2013.pdf |
| | | |
| | **Percentage of prescriptions dispensed at no cost to patients** | |
| | Data - % of prescriptions | A quantification is not possible, but there are certain groups of the population that are exempt from co-payments, e.g. when low income, children, |
| | Time | 2023 |
| | Reference | https://www.bundesgesundheitsministerium.de/zuzahlung-und-erstattung-arzneimittel.html |
| | | |
| | **Population coverage** | |
| | Data | „Around 87% of the population is covered through SHI, while approximately 11% has substitutive PHI coverage. The other 2% (e.g. soldiers) are covered under special programmes. Around 61 000 people are uninsured." ([Blümel et al., p. xxv] |
| | Time | 2020 |
| | Reference | https://eurohealthobservatory.who.int/publications/i/germany-health-system-review-2020 |
| | | |
| | **Availability of doctors' services for citizens at no payment** | |
| | Data | NA |
| | Time | NA |
| | Reference | NA |
| | | |
| Therapy-related factors | **Average number of medicines per patient** | |
| | Data - N of medicines per patient | 4.1 |
| | Time | 2020 |
| | Reference | https://www.barmer.de/presse/infothek/studien-und-reporte/arzneimittelreporte |
| | | |
| | **Proportion of 75 years and over who are taking more than 5 medications concurrently** | |
| | Data - % of persons | approx 35% |
| | Time | 2021; 2016 |
| | Reference | https://bpspubs.onlinelibrary.wiley.com/doi/full/10.1111/bcp.14671; https://www.barmer.de/presse/infothek/studien-und-reporte/arzneimittelreporte |
| | | |

| | | |
|---|---|---|
| | **Percentage of self-reported use of prescribed medicines** | |
| | Data - % of persons | 54 |
| | Time | 2019 |
| | Reference | https://ec.europa.eu/eurostat/databrowser/view/HLTH_EHIS_MD1E__custom_3764895/default/table?lang=en / |

| | | |
|---|---|---|
| **Patient-related factors** | **Percentage of persons reporting a chronic disease** | |
| | Data - Asthma, % of persons | 8 |
| | Data - Chronic lower respiratory diseases, % of persons | 5.9 |
| | Data - High blood pressure, % of persons | 26.2 |
| | Data - Diabetes, % of persons | 8.7 |
| | Data - Chronic depression, % of persons | 11.6 |
| | Time | 2019 |
| | Reference | https://ec.europa.eu/eurostat/databrowser/view/HLTH_EHIS_CD1E/default/table?lang=en&category=hlth.hlth_state.hlth_srcm / |
| | | |
| | **Percentage of self-perceived health - very good (16 years and over)** | |
| | Data - % of persons | 19.7 |
| | Time | 2021 |
| | Reference | https://ec.europa.eu/eurostat/databrowser/view/HLTH_SILC_02/default/table?lang=en&category=hlth.hlth_state.hlth_sph/ |
| | | |
| | **Percenatge of persons with current depressive symptoms** | |
| | Data - % of persons | 9.4 |
| | Time | 2019 |
| | Reference | https://ec.europa.eu/eurostat/databrowser/view/HLTH_EHIS_MH1E/default/table?lang=en&category=hlth.hlth_state.hlth_sph / |

| | | |
|---|---|---|
| **Condition-related factor** | **General health literacy** | |
| | Data - Inadequate health literacy, % of persons | 24 |
| | Data - Problematic health literacy, % of persons | 48 |
| | Data - Sufficient health literacy, % of persons | 23 |
| | Data - Excellent health literacy, % of persons | 5 |
| | Time | 2021 |
| | Reference | https://m-pohl.net/Int_Report_methdology_results_recommendations |

| | | |
|---|---|---|
| **Healthcare system-relat** | **Percentage of patients receiving adherence interventions** | |

| | |
|---|---|
| Data - % of persons | NA |
| Time | NA |
| Reference | NA |
| | |
| **Nationwide availability of e-prescription** | |
| Data | Yes |
| Time | 2023 |
| Reference | https://www.bundesgesundheitsministerium.de/e-rezept.html |
| | |
| **Waiting time for prescriptions / medical appointments** | |
| Data | 49% of SHI insurees do not report waiting time, 12% up to three days, 37% more than three days. People with SHI wait longer than with private insurance |
| Time | 2020; 2008 |
| Reference | https://www.kbv.de/media/sp/Berichtband_Ergebnisse_KBV_Versichertenbefragung_2020.pdf; http://www.ncbi.nlm.nih.gov/pubmed/18184426 |
| | |
| **Number of practising physicians per 100,000 inhabitants** | |
| Data - N of practisisng physicians per 100,000 inhabitants | 453.22 |
| Time | 2021 |
| Reference | https://ec.europa.eu/eurostat/databrowser/view/TPS00044/default/table?lang=en&category=hlth.hlth_care.hlth_res.hlth_staff%20%2F |
| | |
| **Proportion of health care expenditure on pharmaceuticals** | |
| Data - % of health care expenditure | 13.658 |
| Time | 2020 |
| Reference | https://data.oecd.org/healthres/pharmaceutical-spending.htm |
| | |
| **Number of practising pharmacists per 100,000 inhabitants** | |
| Data - N of practisisng pharmacists per 100,000 inhabitants | 66.95 |
| Time | 2020 |
| Reference | https://ec.europa.eu/eurostat/databrowser/view/HLTH_RS_PRS1__custom_4104351/default/table?lang=en |
| | |
| **Total health care expenditure as percentage of GDP** | |
| Data - % of GDP | 12.82 |
| Time | 2020 |

| | | |
|---|---|---|
| | Reference | https://ec.europa.eu/eurostat/databrowser/view/TPS00207/default/table?lang=en&category=hlth.hlth_care.hlth_sha11.hlth_sha11_sum |
| | | |
| | **Public pharmaceutical expenditure as percentage of total pharmaceutical expenditure** | |
| | Data - % of total pharmaceutical expenditure | 75.6 |
| | Time | 2011 |
| | Reference | https://gateway.euro.who.int/en/indicators/hfa_580-6790-public-pharmaceutical-expenditure-as-of-total-pharmaceutical-expenditure/visualizations/#id=19675&tab=table |
| | | |
| | **Self-reported consultations of a medical doctor*** | |
| | Data - No contact, % of population according to the number of consultations of a medical doctor in the past 4 weeks | 54.7 |
| | Data - 1 contact, % of population according to the number of consultations of a medical doctor in the past 4 weeks | 23.3 |
| | Data - 2 contacts, % of population according to the number of consultations of a medical doctor in the past 4 weeks | 11.5 |
| | Data - 3 or more contacts, % of population according to the number of consultations of a medical doctor in the past 4 weeks | 10.4 |
| | Time | 2019 |
| | Reference | https://ec.europa.eu/eurostat/databrowser/view/HLTH_EHIS_AM2U/default/table?lang=en&category=hlth.hlth_care.hlth_consult / |

*Medical doctors include generalist medical practitioners and specialist medical practitioners

GREECE

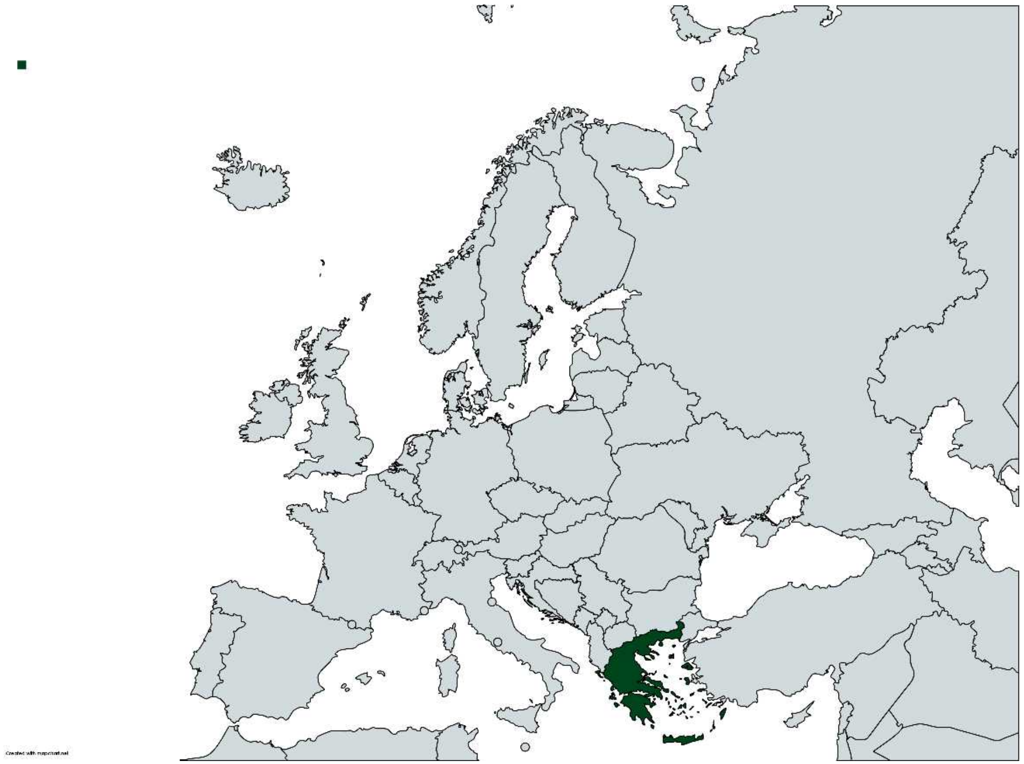

| Country | Greece |
|---|---|

Country-specific data

| Country characteristics | Method of payment | |
|---|---|---|
| | Data | All citizens have UHC and access to public health services, independently of coverage. As regards medication they pay 0-25% out of their pocket, depending on the disease eg 0% for cancer medication, 25% for diabetes (apart of insulin) |
| | Time | 2023 |
| | Reference | Expert opinion |
| | | |
| | Medication adherence assessed and reported on the national level | |
| | Data | No |
| | Time | 2022 |
| | Reference | NA |
| | | |
| | Health care provider | |
| | Data | Out of pocket payment for private health services 35.2% |
| | Time | 2021 |
| | Reference | https://health.ec.europa.eu/system/files/2022-01/2021_chp_gr_greek.pdf |
| | | |
| | Model of healthcare system financing | |
| | Data | NA |
| | Time | NA |
| | Reference | NA |
| | | |
| | Proportion of population aged 65 years and over | |
| | Data - % of persons | 22.5 |
| | Time | 2021 |
| | Reference | https://ec.europa.eu/eurostat/databrowser/view/TPS00028/default/table?lang=en&category=demo.demo_ind / |
| | | |
| | Country population (projection) | |
| | Data - N of persons | 10696535 |
| | Time | 2020 |
| | Reference | https://ec.europa.eu/eurostat/databrowser/view/CENS_HNMGA/default/table?lang=en&category=cens.cens_hn.cens_hnstr |

| | | |
|---|---|---|
| Social/economic factors | **Patient co-payment** | |
| | Data | NA |
| | Time | NA |
| | Reference | NA |
| | | |
| | **Percentage of prescriptions dispensed at no cost to patients** | |
| | Data - % of prescriptions | NA |
| | Time | NA |
| | Reference | NA |
| | | |
| | **Population coverage** | |
| | Data | NA |
| | Time | NA |
| | Reference | NA |
| | | |
| | **Availability of doctors' services for citizens at no payment** | |
| | Data | NA |
| | Time | NA |
| | Reference | NA |
| | | |
| Therapy-related factors | **Average number of medicines per patient** | |
| | Data - N of medicines per patient | NA |
| | Time | NA |
| | Reference | NA |
| | | |
| | **Proportion of 75 years and over who are taking more than 5 medications concurrently** | |
| | Data - % of persons | NA |
| | Time | NA |
| | Reference | NA |
| | | |
| | **Percentage of self-reported use of prescribed medicines** | |
| | Data - % of persons | 43.5 |

| | | |
|---|---|---|
| | Time | 2019 |
| | Reference | https://ec.europa.eu/eurostat/databrowser/view/HLTH_EHIS_MD1E__custom_3764895/default/table?lang=en / |

| | | |
|---|---|---|
| **Patient-related factors** | **Percentage of persons reporting a chronic disease** | |
| | Data - Asthma, % of persons | 3.3 |
| | Data - Chronic lower respiratory diseases, % of persons | 1.9 |
| | Data - High blood pressure, % of persons | 19.6 |
| | Data - Diabetes, % of persons | 8 |
| | Data - Chronic depression, % of persons | 3.8 |
| | Time | 2019 |
| | Reference | https://ec.europa.eu/eurostat/databrowser/view/HLTH_EHIS_CD1E/default/table?lang=en&category=hlth.hlth_state.hlth_srcm / |
| | | |
| | **Percentage of self-perceived health - very good (16 years and over)** | |
| | Data - % of persons | 46.9 |
| | Time | 2021 |
| | Reference | https://ec.europa.eu/eurostat/databrowser/view/HLTH_SILC_02/default/table?lang=en&category=hlth.hlth_state.hlth_sph/ |
| | | |
| | **Percenatge of persons with current depressive symptoms** | |
| | Data - % of persons | 2.7 |
| | Time | 2019 |
| | Reference | https://ec.europa.eu/eurostat/databrowser/view/HLTH_EHIS_MH1E/default/table?lang=en&category=hlth.hlth_state.hlth_sph / |

| | | |
|---|---|---|
| **Condition-related factor** | **General health literacy** | |
| | Data - Inadequate health literacy, % of persons | 13.9 |
| | Data - Problematic health literacy, % of persons | 30.9 |
| | Data - Sufficient health literacy, % of persons | 39.6 |
| | Data - Excellent health literacy, % of persons | 15.6 |
| | Time | 2011 |
| | Reference | https://academic.oup.com/eurpub/article/25/6/1053/2467145 |

| | | |
|---|---|---|
| **Healthcare system-relat** | **Percentage of patients receiving adherence interventions** | |
| | Data - % of persons | NA |
| | Time | NA |

| | |
|---|---|
| Reference | NA |
| | |
| **Nationwide availability of e-prescription** | |
| Data | NA |
| Time | NA |
| Reference | NA |
| | |
| **Waiting time for prescriptions / medical appointments** | |
| Data | NA |
| Time | NA |
| Reference | NA |
| | |
| **Number of practising physicians per 100,000 inhabitants** | |
| Data - N of practisisng physicians per 100,000 inhabitants | NA |
| Time | NA |
| Reference | NA |
| | |
| **Proportion of health care expenditure on pharmaceuticals** | |
| Data - % of health care expenditure | 30.198 |
| Time | 2020 |
| Reference | https://data.oecd.org/healthres/pharmaceutical-spending.htm |
| | |
| **Number of practising pharmacists per 100,000 inhabitants** | |
| Data - N of practisisng pharmacists per 100,000 inhabitants | NA |
| Time | NA |
| Reference | NA |
| | |
| **Total health care expenditure as percentage of GDP** | |
| Data - % of GDP | 9.51 |
| Time | 2020 |
| Reference | https://ec.europa.eu/eurostat/databrowser/view/TPS00207/default/table?lang=en&category=hlth.hlth_care.hlth_sha11.hlth_sha11_sum |
| | |
| **Public pharmaceutical expenditure as percentage of total pharmaceutical expenditure** | |

| | | |
|---|---|---|
| | Data - % of total pharmaceutical expenditure | 54.8 |
| | Time | 2020 |
| | Reference | https://gateway.euro.who.int/en/indicators/hfa_580-6790-public-pharmaceutical-expenditure-as-of-total-pharmaceutical-expenditure/visualizations/#id=19675&tab=table |
| | | |
| | **Self-reported consultations of a medical doctor*** | |
| | Data - No contact, % of population according to the number of consultations of a medical doctor in the past 4 weeks | 62.9 |
| | Data - 1 contact, % of population according to the number of consultations of a medical doctor in the past 4 weeks | 20.6 |
| | Data - 2 contacts, % of population according to the number of consultations of a medical doctor in the past 4 weeks | 9.7 |
| | Data - 3 or more contacts, % of population according to the number of consultations of a medical doctor in the past 4 weeks | 6.8 |
| | Time | 2019 |
| | Reference | https://ec.europa.eu/eurostat/databrowser/view/HLTH_EHIS_AM2U/default/table?lang=en&category=hlth.hlth_care.hlth_consult / |

*Medical doctors include generalist medical practitioners and specialist medical practitioners

HUNGARY

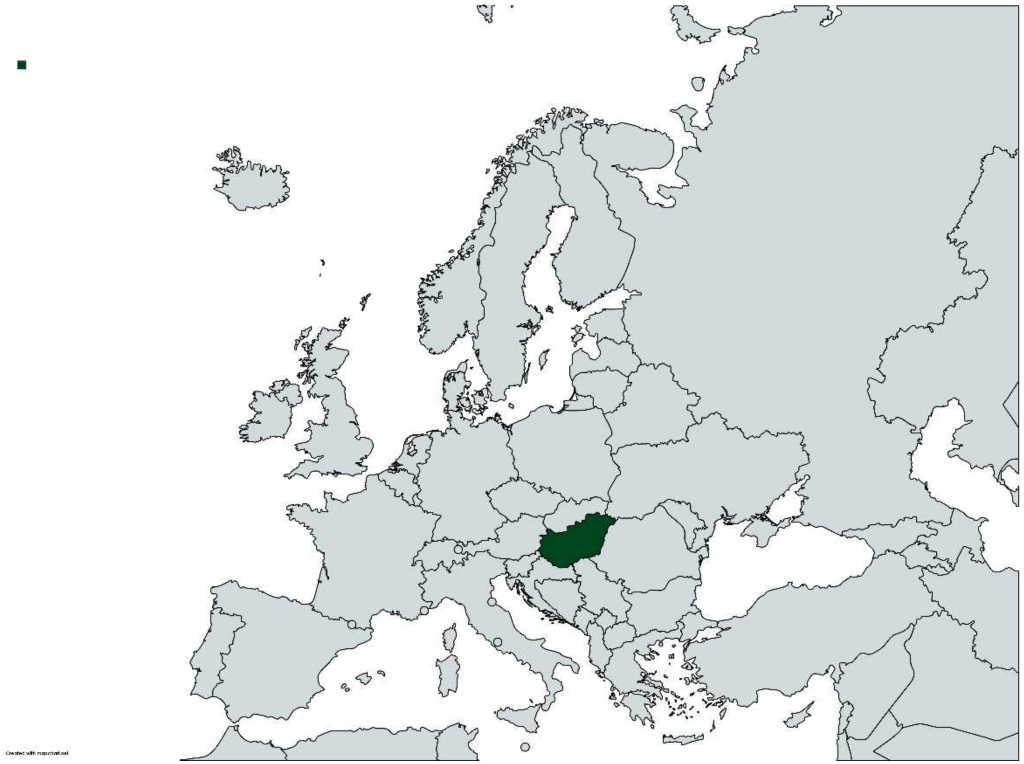

| Country | Hungary |
|---|---|

Country-specific data

| Country characteristics | Method of payment | |
|---|---|---|
| | Data | Cost of drugs paid by the National Health Insurance Fund Administration. |
| | Time | 2013 |
| | Reference | http://imi-protect.eu/documents/DUinventory_2013_COUNTRIESyear4_Dec2013.pdf |
| | | |
| | Medication adherence assessed and reported on the national level | |
| | Data | No |
| | Time | 2023 |
| | Reference | Expert opinion |
| | | |
| | Health care provider | |
| | Data | Public Statutory Health Isurance system. Universal access for all residents in Hungary, |
| | Time | 2013 |
| | Reference | http://imi-protect.eu/documents/DUinventory_2013_COUNTRIESyear4_Dec2013.pdf |
| | | |
| | Model of healthcare system financing | |
| | Data | Compulsory contributions to the SHI and genaral and local tax revenues. 23.7% is financed by out-of-pocket payments (including informal payments). |
| | Time | 2013 |
| | Reference | http://imi-protect.eu/documents/DUinventory_2013_COUNTRIESyear4_Dec2013.pdf |
| | | |
| | Proportion of population aged 65 years and over | |
| | Data - % of persons | 20.3 |
| | Time | 2021 |
| | Reference | https://ec.europa.eu/eurostat/databrowser/view/TPS00028/default/table?lang=en&category=demo.demo_ind / |
| | | |
| | Country population (projection) | |
| | Data - N of persons | 9771975 |
| | Time | 2020 |
| | Reference | https://ec.europa.eu/eurostat/databrowser/view/CENS_HNMGA/default/table?lang=en&category=cens.cens_hn.cens_hnstr |

| | | |
|---|---|---|
| Social/economic factors | **Patient co-payment** | |
| | Data | For fixed reimbursement type, copayment may be 20%, 45%, 75%. For some severe diseases indication realted reimbursement, copayment is 0% with a package fee of EUR1. For less severe diseases, the compayment is 10%, 30% or 50%. |
| | Time | 2013 |
| | Reference | http://imi-protect.eu/documents/DUinventory_2013_COUNTRIESyear4_Dec2013.pdf |
| | | |
| | **Percentage of prescriptions dispensed at no cost to patients** | |
| | Data - % of prescriptions | NA |
| | Time | NA |
| | Reference | NA |
| | | |
| | **Population coverage** | |
| | Data | 96% of population covered by the National Health Insurance Fund Administration. |
| | Time | 2013 |
| | Reference | http://imi-protect.eu/documents/DUinventory_2013_COUNTRIESyear4_Dec2013.pdf |
| | | |
| | **Availability of doctors' services for citizens at no payment** | |
| | Data | All services that are included in the basic health insurance pacakge are available at no payment for patients |
| | Time | 2023 |
| | Reference | www.neak.hu |
| | | |
| Therapy-related factors | **Average number of medicines per patient** | |
| | Data - N of medicines per patient | NA |
| | Time | NA |
| | Reference | NA |
| | | |
| | **Proportion of 75 years and over who are taking more than 5 medications concurrently** | |
| | Data - % of persons | NA |
| | Time | NA |
| | Reference | NA |
| | | |
| | **Percentage of self-reported use of prescribed medicines** | |
| | Data - % of persons | 50.8 |

| | | |
|---|---|---|
| | Time | 2019 |
| | Reference | https://ec.europa.eu/eurostat/databrowser/view/HLTH_EHIS_MD1E__custom_3764895/default/table?lang=en / |

| | | |
|---|---|---|
| Patient-related factors | **Percentage of persons reporting a chronic disease** | |
| | Data - Asthma, % of persons | 5 |
| | Data - Chronic lower respiratory diseases, % of persons | 4.2 |
| | Data - High blood pressure, % of persons | 31.5 |
| | Data - Diabetes, % of persons | 8.9 |
| | Data - Chronic depression, % of persons | 4 |
| | Time | 2019 |
| | Reference | https://ec.europa.eu/eurostat/databrowser/view/HLTH_EHIS_CD1E/default/table?lang=en&category=hlth.hlth_state.hlth_srcm / |
| | | |
| | **Percentage of self-perceived health - very good (16 years and over)** | |
| | Data - % of persons | 17.9 |
| | Time | 2021 |
| | Reference | https://ec.europa.eu/eurostat/databrowser/view/HLTH_SILC_02/default/table?lang=en&category=hlth.hlth_state.hlth_sph/ |
| | | |
| | **Percenatge of persons with current depressive symptoms** | |
| | Data - % of persons | 5.5 |
| | Time | 2019 |
| | Reference | https://ec.europa.eu/eurostat/databrowser/view/HLTH_EHIS_MH1E/default/table?lang=en&category=hlth.hlth_state.hlth_sph / |

| | | |
|---|---|---|
| Condition-related factor | **General health literacy** | |
| | Data - Inadequate health literacy, % of persons | 11 |
| | Data - Problematic health literacy, % of persons | 30 |
| | Data - Sufficient health literacy, % of persons | 50 |
| | Data - Excellent health literacy, % of persons | 9 |
| | Time | 2021 |
| | Reference | https://m-pohl.net/Int_Report_methdology_results_recommendations |

| | | |
|---|---|---|
| Healthcare system-relat | **Percentage of patients receiving adherence interventions** | |
| | Data - % of persons | NA |
| | Time | NA |

| | |
|---|---|
| Reference | NA |
| | |
| **Nationwide availability of e-prescription** | |
| Data | Yes |
| Time | 2019 |
| Reference | https://www.euro.who.int/__data/assets/pdf_file/0007/419461/Country-Health-Profile-2019-Hungary.pdf |
| | |
| **Waiting time for prescriptions / medical appointments** | |
| Data | NA |
| Time | NA |
| Reference | NA |
| | |
| **Number of practising physicians per 100,000 inhabitants** | |
| Data - N of practisisng physicians per 100,000 inhabitants | 328.35 |
| Time | 2021 |
| Reference | https://ec.europa.eu/eurostat/databrowser/view/TPS00044/default/table?lang=en&category=hlth.hlth_care.hlth_res.hlth_staff%20%2F |
| | |
| **Proportion of health care expenditure on pharmaceuticals** | |
| Data - % of health care expenditure | 25.616 |
| Time | 2020 |
| Reference | https://data.oecd.org/healthres/pharmaceutical-spending.htm |
| | |
| **Number of practising pharmacists per 100,000 inhabitants** | |
| Data - N of practisisng pharmacists per 100,000 inhabitants | 78.41 |
| Time | 2020 |
| Reference | https://ec.europa.eu/eurostat/databrowser/view/HLTH_RS_PRS1__custom_4104351/default/table?lang=en |
| | |
| **Total health care expenditure as percentage of GDP** | |
| Data - % of GDP | 7.25 |
| Time | 2020 |
| Reference | https://ec.europa.eu/eurostat/databrowser/view/TPS00207/default/table?lang=en&category=hlth.hlth_care.hlth_sha11.hlth_sha11_sum |
| | |

| | | |
|---|---|---|
| | **Public pharmaceutical expenditure as percentage of total pharmaceutical expenditure** | |
| | Data - % of total pharmaceutical expenditure | 49 |
| | Time | 2011 |
| | Reference | https://gateway.euro.who.int/en/indicators/hfa_580-6790-public-pharmaceutical-expenditure-as-of-total-pharmaceutical-expenditure/visualizations/#id=19675&tab=table |
| | | |
| | **Self-reported consultations of a medical doctor*** | |
| | Data - No contact, % of population according to the number of consultations of a medical doctor in the past 4 weeks | 53.6 |
| | Data - 1 contact, % of population according to the number of consultations of a medical doctor in the past 4 weeks | 23.5 |
| | Data - 2 contacts, % of population according to the number of consultations of a medical doctor in the past 4 weeks | 13.8 |
| | Data - 3 or more contacts, % of population according to the number of consultations of a medical doctor in the past 4 weeks | 9.1 |
| | Time | 2019 |
| | Reference | https://ec.europa.eu/eurostat/databrowser/view/HLTH_EHIS_AM2U/default/table?lang=en&category=hlth.hlth_care.hlth_consult / |

*Medical doctors include generalist medical practitioners and specialist medical practitioners

# ICELAND

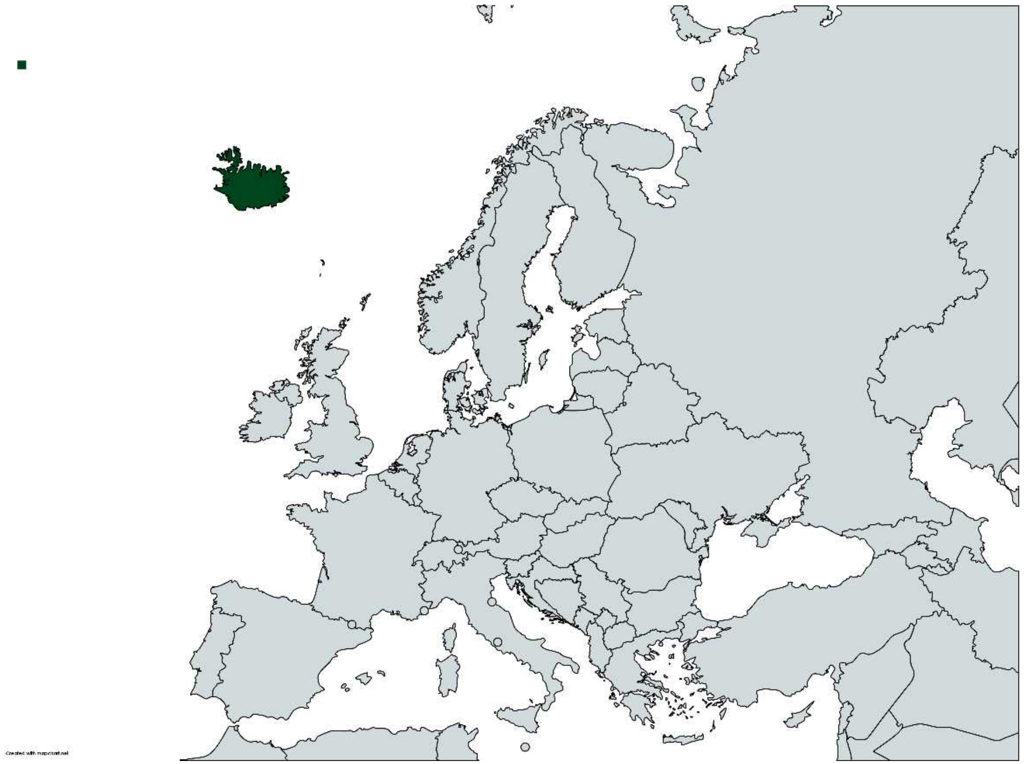

| Country | Iceland |
|---|---|

Country-specific data

| Country characteristics | Method of payment | |
|---|---|---|
| | Data | The patient pays part of the medicines and the remainder is reimbursed by the state outpatient health plan. |
| | Time | 2013 |
| | Reference | http://imi-protect.eu/documents/DUinventory_2013_COUNTRIESyear4_Dec2013.pdf |
| | | |
| | Medication adherence assessed and reported on the national level | |
| | Data | No |
| | Time | 2022 |
| | Reference | NA |
| | | |
| | Health care provider | |
| | Data | Public. It si covered by the Sate. Private health insurance hardly exists. |
| | Time | 2013 |
| | Reference | http://imi-protect.eu/documents/DUinventory_2013_COUNTRIESyear4_Dec2013.pdf |
| | | |
| | Model of healthcare system financing | |
| | Data | The healthcare system is largely paid by taxes (85%) and to some extent by service fees (15%). |
| | Time | 2013 |
| | Reference | http://imi-protect.eu/documents/DUinventory_2013_COUNTRIESyear4_Dec2013.pdf |
| | | |
| | Proportion of population aged 65 years and over | |
| | Data - % of persons | 14.7 |
| | Time | 2021 |
| | Reference | https://ec.europa.eu/eurostat/databrowser/view/TPS00028/default/table?lang=en&category=demo.demo_ind / |
| | | |
| | Country population (projection) | |
| | Data - N of persons | 365042 |
| | Time | 2020 |
| | Reference | https://ec.europa.eu/eurostat/databrowser/view/CENS_HNMGA/default/table?lang=en&category=cens.cens_hn.cens_hnstr |

| | | |
|---|---|---|
| Social/economic factors | **Patient co-payment** | |
| | Data | The level of copayment depends on the 12-month pharmaceutical expenditure for the individual, which starts with the first drug purchased. Step 1 ISK 20.000: 100% copayment. Step 2 ISK 31750: 15% copayment, Step 3: ISK 62000:7.5% copayment. Total ISK 62000. Above that amount the patients copayment is ISK0. For seniors 67 years and above . Step 1 ISK 11.000: 100% copayment. Step 2 ISK 17900: 15% copayment, Step 3: ISK 41000:7.5% copayment. Total ISK 41000. Above that amount the patients copayment is ISK0. |
| | Time | 2013 |
| | Reference | http://imi-protect.eu/documents/DUinventory_2013_COUNTRIESyear4_Dec2013.pdf |
| | | |
| | **Percentage of prescriptions dispensed at no cost to patients** | |
| | Data - % of prescriptions | NA |
| | Time | NA |
| | Reference | NA |
| | | |
| | **Population coverage** | |
| | Data | Universal. All residents for at least 6 months are entitled to health care. |
| | Time | 2013 |
| | Reference | http://imi-protect.eu/documents/DUinventory_2013_COUNTRIESyear4_Dec2013.pdf |
| | | |
| | **Availability of doctors' services for citizens at no payment** | |
| | Data | Children under 2 years old = no payment |
| | Time | 2023 |
| | Reference | https://island.is/greidsluthatttaka-vegna-heilbrigdisthjonustu |
| | | |
| Therapy-related factors | **Average number of medicines per patient** | |
| | Data - N of medicines per patient | NA |
| | Time | NA |
| | Reference | NA |
| | | |
| | **Proportion of 75 years and over who are taking more than 5 medications concurrently** | |
| | Data - % of persons | 55.9 |
| | Time | 2020 |
| | Reference | https://stats.oecd.org/Index.aspx?ThemeTreeId=24 |

| | | |
|---|---|---|
| | | |
| | **Percentage of self-reported use of prescribed medicines** | |
| | Data - % of persons | 59.4 |
| | Time | 2019 |
| | Reference | https://ec.europa.eu/eurostat/databrowser/view/HLTH_EHIS_MD1E__custom_3764895/default/table?lang=en / |

| | | |
|---|---|---|
| Patient-related factors | **Percentage of persons reporting a chronic disease** | |
| | Data - Asthma, % of persons | 10.2 |
| | Data - Chronic lower respiratory diseases, % of persons | 4.7 |
| | Data - High blood pressure, % of persons | 24.5 |
| | Data - Diabetes, % of persons | 5.6 |
| | Data - Chronic depression, % of persons | 15.6 |
| | Time | 2019 |
| | Reference | https://ec.europa.eu/eurostat/databrowser/view/HLTH_EHIS_CD1E/default/table?lang=en&category=hlth.hlth_state.hlth_srcm / |
| | | |
| | **Percentage of self-perceived health - very good (16 years and over)** | |
| | Data - % of persons | 38.1 |
| | Time | 2018 |
| | Reference | https://ec.europa.eu/eurostat/databrowser/view/HLTH_SILC_02/default/table?lang=en&category=hlth.hlth_state.hlth_sph/ |
| | | |
| | **Percenatge of persons with current depressive symptoms** | |
| | Data - % of persons | 7.7 |
| | Time | 2019 |
| | Reference | https://ec.europa.eu/eurostat/databrowser/view/HLTH_EHIS_MH1E/default/table?lang=en&category=hlth.hlth_state.hlth_sph / |

| | | |
|---|---|---|
| Condition-related factor | **General health literacy** | |
| | Data - Inadequate health literacy, % of persons | NA |
| | Data - Problematic health literacy, % of persons | NA |
| | Data - Sufficient health literacy, % of persons | NA |
| | Data - Excellent health literacy, % of persons | NA |
| | Time | NA |
| | Reference | NA |

| Healthcare system-relat | Percentage of patients receiving adherence interventions | |
|---|---|---|
| | Data - % of persons | NA |
| | Time | NA |
| | Reference | NA |
| | | |
| | **Nationwide availability of e-prescription** | |
| | Data | Yes |
| | Time | 2023 |
| | Reference | https://www.landlaeknir.is/gaedi-og-eftirlit/heilbrigdisthjonusta/rafraen-sjukraskra/adgangur-ad-lyfjagagnagrunni/ |
| | | |
| | **Waiting time for prescriptions / medical appointments** | |
| | Data | NA |
| | Time | NA |
| | Reference | NA |
| | | |
| | **Number of practising physicians per 100,000 inhabitants** | |
| | Data - N of practisisng physicians per 100,000 inhabitants | 389.39 |
| | Time | 2019 |
| | Reference | https://ec.europa.eu/eurostat/databrowser/view/TPS00044/default/table?lang=en&category=hlth.hlth_care.hlth_res.hlth_staff%20%2F |
| | | |
| | **Proportion of health care expenditure on pharmaceuticals** | |
| | Data - % of health care expenditure | 11.911 |
| | Time | 2021 |
| | Reference | https://data.oecd.org/healthres/pharmaceutical-spending.htm |
| | | |
| | **Number of practising pharmacists per 100,000 inhabitants** | |
| | Data - N of practisisng pharmacists per 100,000 inhabitants | 56.76 |
| | Time | 2020 |
| | Reference | https://ec.europa.eu/eurostat/databrowser/view/HLTH_RS_PRS1__custom_4104351/default/table?lang=en |
| | | |
| | **Total health care expenditure as percentage of GDP** | |
| | Data - % of GDP | 9.56 |
| | Time | 2020 |

| | | |
|---|---|---|
| | Reference | https://ec.europa.eu/eurostat/databrowser/view/TPS00207/default/table?lang=en&category=hlth.hlth_care.hlth_sha11.hlth_sha11_sum |
| | | |
| | **Public pharmaceutical expenditure as percentage of total pharmaceutical expenditure** | |
| | Data - % of total pharmaceutical expenditure | 42.1 |
| | Time | 2011 |
| | Reference | https://gateway.euro.who.int/en/indicators/hfa_580-6790-public-pharmaceutical-expenditure-as-of-total-pharmaceutical-expenditure/visualizations/#id=19675&tab=table |
| | | |
| | **Self-reported consultations of a medical doctor*** | |
| | Data - No contact, % of population according to the number of consultations of a medical doctor in the past 4 weeks | 69.2 |
| | Data - 1 contact, % of population according to the number of consultations of a medical doctor in the past 4 weeks | 18.9 |
| | Data - 2 contacts, % of population according to the number of consultations of a medical doctor in the past 4 weeks | 7.4 |
| | Data - 3 or more contacts, % of population according to the number of consultations of a medical doctor in the past 4 weeks | 4.5 |
| | Time | 2019 |
| | Reference | https://ec.europa.eu/eurostat/databrowser/view/HLTH_EHIS_AM2U/default/table?lang=en&category=hlth.hlth_care.hlth_consult / |

*Medical doctors include generalist medical practitioners and specialist medical practitioners

IRELAND

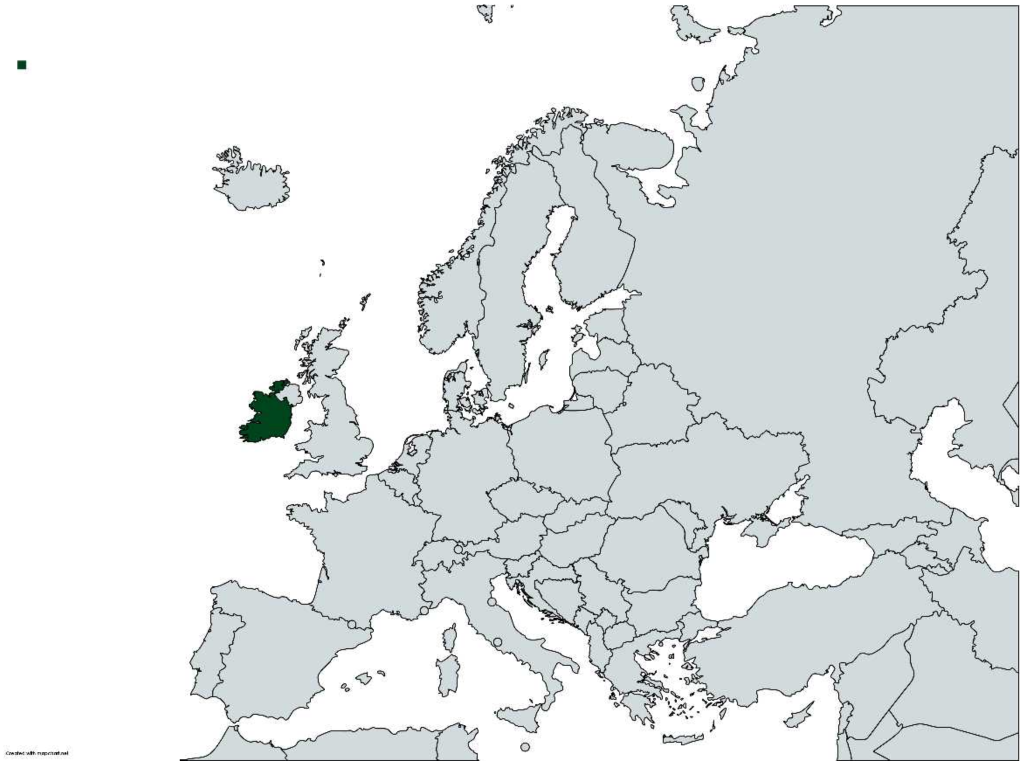

| Country | Ireland |
|---|---|

Country-specific data

| Country characteristics | Method of payment | |
|---|---|---|
| | Data | The Health Service Executive Primary Care Reimbursement pays totally or partially the cost of the drugs under the Drug Payment Scheme. |
| | Time | 2013 |
| | Reference | http://imi-protect.eu/documents/DUinventory_2013_COUNTRIESyear4_Dec2013.pdf |
| | | |
| | Medication adherence assessed and reported on the national level | |
| | Data | No |
| | Time | 2022 |
| | Reference | NA |
| | | |
| | Health care provider | |
| | Data | Public: managed by the Health and Safety Executive who are accountable to the Departement of Health. Private: private health insurance or co-payment. |
| | Time | 2013 |
| | Reference | http://imi-protect.eu/documents/DUinventory_2013_COUNTRIESyear4_Dec2013.pdf |
| | | |
| | Model of healthcare system financing | |
| | Data | Predominantly tax funded (78%). Although approximately 50% of the population had private health insurance and around two thirds may a co-payment towards public healthcare. Approximately one third has free access to public services. |
| | Time | 2013 |
| | Reference | http://imi-protect.eu/documents/DUinventory_2013_COUNTRIESyear4_Dec2013.pdf |
| | | |
| | Proportion of population aged 65 years and over | |
| | Data - % of persons | 14.8 |
| | Time | 2021 |
| | Reference | https://ec.europa.eu/eurostat/databrowser/view/TPS00028/default/table?lang=en&category=demo.demo_ind / |
| | | |
| | Country population (projection) | |
| | Data - N of persons | 4966879 |
| | Time | 2020 |

| | | |
|---|---|---|
| | Reference | https://ec.europa.eu/eurostat/databrowser/view/CENS_HNMGA/default/table?lang=en&category=cens.cens_hn.cens_hnstr |

| | | |
|---|---|---|
| **Social/economic factors** | **Patient co-payment** | |
| | Data | Ther are 4 schemes: (i) General Medical Srevices Scheme: the patient has to pay EUR2.50 per item prescription (up to a maximum charge of EUR25 per family/month). (ii) Drug Payment Scheme: the patient pays a maximum of EUR144/month for approved meicines. (iii) Long Term Illness Scheme: free of charge for specific conditions. (iv) Hi-Tech Scheme: free of charge. |
| | Time | 2013 |
| | Reference | http://imi-protect.eu/documents/DUinventory_2013_COUNTRIESyear4_Dec2013.pdf |
| | | |
| | **Percentage of prescriptions dispensed at no cost to patients** | |
| | Data - % of prescriptions | NA |
| | Time | NA |
| | Reference | NA |
| | | |
| | **Population coverage** | |
| | Data | All persons resident are entitled to receive health care through the public health care system. |
| | Time | 2013 |
| | Reference | http://imi-protect.eu/documents/DUinventory_2013_COUNTRIESyear4_Dec2013.pdf |
| | | |
| | **Availability of doctors' services for citizens at no payment** | |
| | Data | NA |
| | Time | NA |
| | Reference | NA |
| | | |
| **Therapy-related factors** | **Average number of medicines per patient** | |
| | Data - N of medicines per patient | NA |
| | Time | NA |
| | Reference | NA |
| | | |
| | **Proportion of 75 years and over who are taking more than 5 medications concurrently** | |
| | Data - % of persons | 61.5 |
| | Time | 2019 |
| | Reference | https://stats.oecd.org/Index.aspx?ThemeTreeId=25 |
| | | |

| | | |
|---|---|---|
| | **Percentage of self-reported use of prescribed medicines** | |
| | Data - % of persons | 44.7 |
| | Time | 2019 |
| | Reference | https://ec.europa.eu/eurostat/databrowser/view/HLTH_EHIS_MD1E__custom_3764895/default/table?lang=en / |

| | | |
|---|---|---|
| **Patient-related factors** | **Percentage of persons reporting a chronic disease** | |
| | Data - Asthma, % of persons | 7.3 |
| | Data - Chronic lower respiratory diseases, % of persons | 2.1 |
| | Data - High blood pressure, % of persons | 11.6 |
| | Data - Diabetes, % of persons | 3.5 |
| | Data - Chronic depression, % of persons | 3.9 |
| | Time | 2019 |
| | Reference | https://ec.europa.eu/eurostat/databrowser/view/HLTH_EHIS_CD1E/default/table?lang=en&category=hlth.hlth_state.hlth_srcm / |
| | | |
| | **Percentage of self-perceived health - very good (16 years and over)** | |
| | Data - % of persons | 45.6 |
| | Time | 2021 |
| | Reference | https://ec.europa.eu/eurostat/databrowser/view/HLTH_SILC_02/default/table?lang=en&category=hlth.hlth_state.hlth_sph/ |
| | | |
| | **Percenatge of persons with current depressive symptoms** | |
| | Data - % of persons | 4.9 |
| | Time | 2019 |
| | Reference | https://ec.europa.eu/eurostat/databrowser/view/HLTH_EHIS_MH1E/default/table?lang=en&category=hlth.hlth_state.hlth_sph / |

| | | |
|---|---|---|
| **Condition-related factor** | **General health literacy** | |
| | Data - Inadequate health literacy, % of persons | 6 |
| | Data - Problematic health literacy, % of persons | 37 |
| | Data - Sufficient health literacy, % of persons | 28 |
| | Data - Excellent health literacy, % of persons | 29 |
| | Time | 2021 |
| | Reference | https://m-pohl.net/Int_Report_methdology_results_recommendations |

| | | |
|---|---|---|
| **Healthcare system-relat** | **Percentage of patients receiving adherence interventions** | |

| | |
|---|---|
| Data - % of persons | NA |
| Time | NA |
| Reference | NA |
| | |
| **Nationwide availability of e-prescription** | |
| Data | NA |
| Time | NA |
| Reference | NA |
| | |
| **Waiting time for prescriptions / medical appointments** | |
| Data | NA |
| Time | NA |
| Reference | NA |
| | |
| **Number of practising physicians per 100,000 inhabitants** | |
| Data - N of practisisng physicians per 100,000 inhabitants | 404.56 |
| Time | 2021 |
| Reference | https://ec.europa.eu/eurostat/databrowser/view/TPS00044/default/table?lang=en&category=hlth.hlth_care.hlth_res.hlth_staff%20%2F |
| | |
| **Proportion of health care expenditure on pharmaceuticals** | |
| Data - % of health care expenditure | 11.514 |
| Time | 2020 |
| Reference | https://data.oecd.org/healthres/pharmaceutical-spending.htm |
| | |
| **Number of practising pharmacists per 100,000 inhabitants** | |
| Data - N of practisisng pharmacists per 100,000 inhabitants | 107.39 |
| Time | 2019 |
| Reference | https://ec.europa.eu/eurostat/databrowser/view/HLTH_RS_PRS1__custom_4104351/default/table?lang=en |
| | |
| **Total health care expenditure as percentage of GDP** | |
| Data - % of GDP | 7.1 |
| Time | 2020 |

| | | |
|---|---|---|
| | Reference | https://ec.europa.eu/eurostat/databrowser/view/TPS00207/default/table?lang=en&category=hlth.hlth_care.hlth_sha11.hlth_sha11_sum |
| | | |
| | **Public pharmaceutical expenditure as percentage of total pharmaceutical expenditure** | |
| | Data - % of total pharmaceutical expenditure | 78 |
| | Time | 2011 |
| | Reference | https://gateway.euro.who.int/en/indicators/hfa_580-6790-public-pharmaceutical-expenditure-as-of-total-pharmaceutical-expenditure/visualizations/#id=19675&tab=table |
| | | |
| | **Self-reported consultations of a medical doctor*** | |
| | Data - No contact, % of population according to the number of consultations of a medical doctor in the past 4 weeks | 69.4 |
| | Data - 1 contact, % of population according to the number of consultations of a medical doctor in the past 4 weeks | 21.6 |
| | Data - 2 contacts, % of population according to the number of consultations of a medical doctor in the past 4 weeks | 5.4 |
| | Data - 3 or more contacts, % of population according to the number of consultations of a medical doctor in the past 4 weeks | 3.6 |
| | Time | 2019 |
| | Reference | https://ec.europa.eu/eurostat/databrowser/view/HLTH_EHIS_AM2U/default/table?lang=en&category=hlth.hlth_care.hlth_consult / |

*Medical doctors include generalist medical practitioners and specialist medical practitioners

ISRAEL

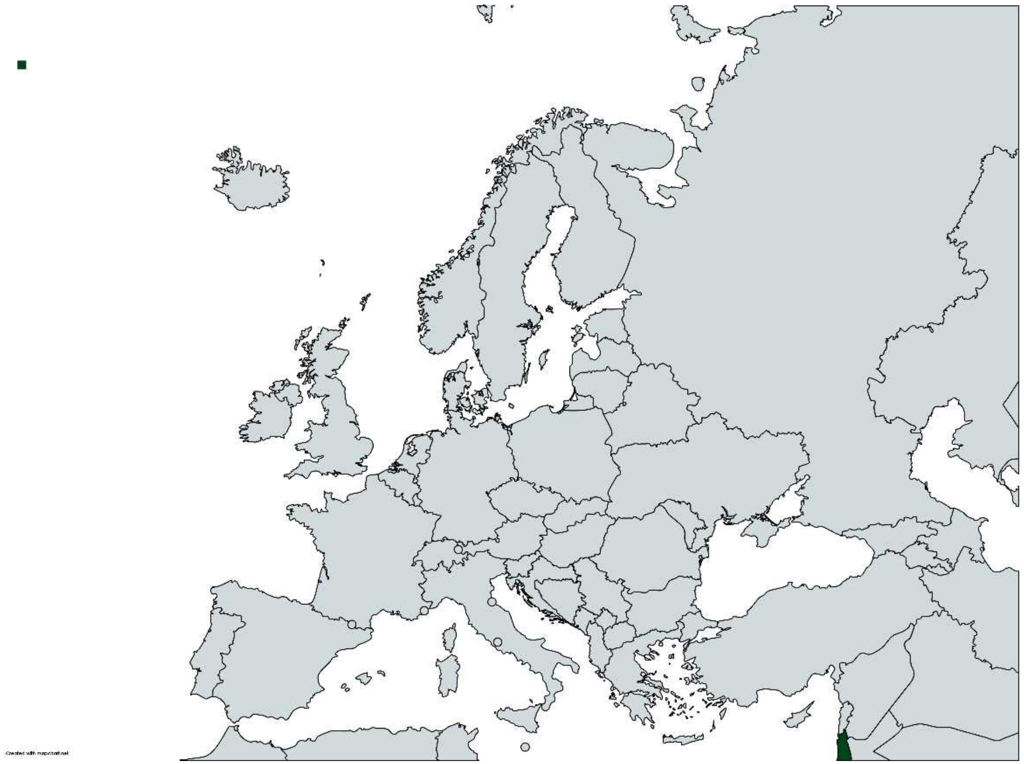

| Country | Israel |
|---|---|

Country-specific data

| Country characteristics | Method of payment | |
|---|---|---|
| | Data | NA |
| | Time | NA |
| | Reference | NA |
| | | |
| | Medication adherence assessed and reported on the national level | |
| | Data | No |
| | Time | 2022 |
| | Reference | NA |
| | | |
| | Health care provider | |
| | Data | NA |
| | Time | NA |
| | Reference | NA |
| | | |
| | Model of healthcare system financing | |
| | Data | NA |
| | Time | NA |
| | Reference | NA |
| | | |
| | Proportion of population aged 65 years and over | |
| | Data - % of persons | NA |
| | Time | NA |
| | Reference | NA |
| | | |
| | Country population (projection) | |
| | Data - N of persons | NA |
| | Time | NA |
| | Reference | NA |

| | | |
|---|---|---|
| Social/economic factors | **Patient co-payment** | |
| | Data | NA |
| | Time | NA |
| | Reference | NA |
| | | |
| | **Percentage of prescriptions dispensed at no cost to patients** | |
| | Data - % of prescriptions | NA |
| | Time | NA |
| | Reference | NA |
| | | |
| | **Population coverage** | |
| | Data | NA |
| | Time | NA |
| | Reference | NA |
| | | |
| | **Availability of doctors' services for citizens at no payment** | |
| | Data | NA |
| | Time | NA |
| | Reference | NA |
| | | |
| Therapy-related factors | **Average number of medicines per patient** | |
| | Data - N of medicines per patient | NA |
| | Time | NA |
| | Reference | NA |
| | | |
| | **Proportion of 75 years and over who are taking more than 5 medications concurrently** | |
| | Data - % of persons | NA |
| | Time | NA |
| | Reference | NA |
| | | |
| | **Percentage of self-reported use of prescribed medicines** | |
| | Data - % of persons | NA |

| | | |
|---|---|---|
| | Time | NA |
| | Reference | NA |

| | | |
|---|---|---|
| **Patient-related factors** | **Percentage of persons reporting a chronic disease** | |
| | Data - Asthma, % of persons | NA |
| | Data - Chronic lower respiratory diseases, % of persons | NA |
| | Data - High blood pressure, % of persons | NA |
| | Data - Diabetes, % of persons | NA |
| | Data - Chronic depression, % of persons | NA |
| | Time | NA |
| | Reference | NA |
| | | |
| | **Percentage of self-perceived health - very good (16 years and over)** | |
| | Data - % of persons | NA |
| | Time | NA |
| | Reference | NA |
| | | |
| | **Percenatge of persons with current depressive symptoms** | |
| | Data - % of persons | NA |
| | Time | NA |
| | Reference | NA |

| | | |
|---|---|---|
| **Condition-related factor** | **General health literacy** | |
| | Data - Inadequate health literacy, % of persons | 18 |
| | Data - Problematic health literacy, % of persons | 34 |
| | Data - Sufficient health literacy, % of persons | 33 |
| | Data - Excellent health literacy, % of persons | 15 |
| | Time | 2021 |
| | Reference | https://m-pohl.net/Int_Report_methdology_results_recommendations |

| | | |
|---|---|---|
| **Healthcare system-relat** | **Percentage of patients receiving adherence interventions** | |
| | Data - % of persons | NA |
| | Time | NA |

| | |
|---|---|
| Reference | NA |
| | |
| **Nationwide availability of e-prescription** | |
| Data | NA |
| Time | NA |
| Reference | NA |
| | |
| **Waiting time for prescriptions / medical appointments** | |
| Data | NA |
| Time | NA |
| Reference | NA |
| | |
| **Number of practising physicians per 100,000 inhabitants** | |
| Data - N of practisisng physicians per 100,000 inhabitants | NA |
| Time | NA |
| Reference | NA |
| | |
| **Proportion of health care expenditure on pharmaceuticals** | |
| Data - % of health care expenditure | 13.434 |
| Time | 2018 |
| Reference | https://data.oecd.org/healthres/pharmaceutical-spending.htm |
| | |
| **Number of practising pharmacists per 100,000 inhabitants** | |
| Data - N of practisisng pharmacists per 100,000 inhabitants | NA |
| Time | NA |
| Reference | NA |
| | |
| **Total health care expenditure as percentage of GDP** | |
| Data - % of GDP | NA |
| Time | NA |
| Reference | NA |
| | |
| **Public pharmaceutical expenditure as percentage of total pharmaceutical expenditure** | |

| | | |
|---|---|---|
| | Data - % of total pharmaceutical expenditure | 51 |
| | Time | 2019 |
| | Reference | https://gateway.euro.who.int/en/indicators/hfa_580-6790-public-pharmaceutical-expenditure-as-of-total-pharmaceutical-expenditure/visualizations/#id=19675&tab=table |
| | | |
| | **Self-reported consultations of a medical doctor*** | |
| | Data - No contact, % of population according to the number of consultations of a medical doctor in the past 4 weeks | NA |
| | Data - 1 contact, % of population according to the number of consultations of a medical doctor in the past 4 weeks | NA |
| | Data - 2 contacts, % of population according to the number of consultations of a medical doctor in the past 4 weeks | NA |
| | Data - 3 or more contacts, % of population according to the number of consultations of a medical doctor in the past 4 weeks | NA |
| | Time | NA |
| | Reference | NA |

*Medical doctors include generalist medical practitioners and specialist medical practitioners

ITALY

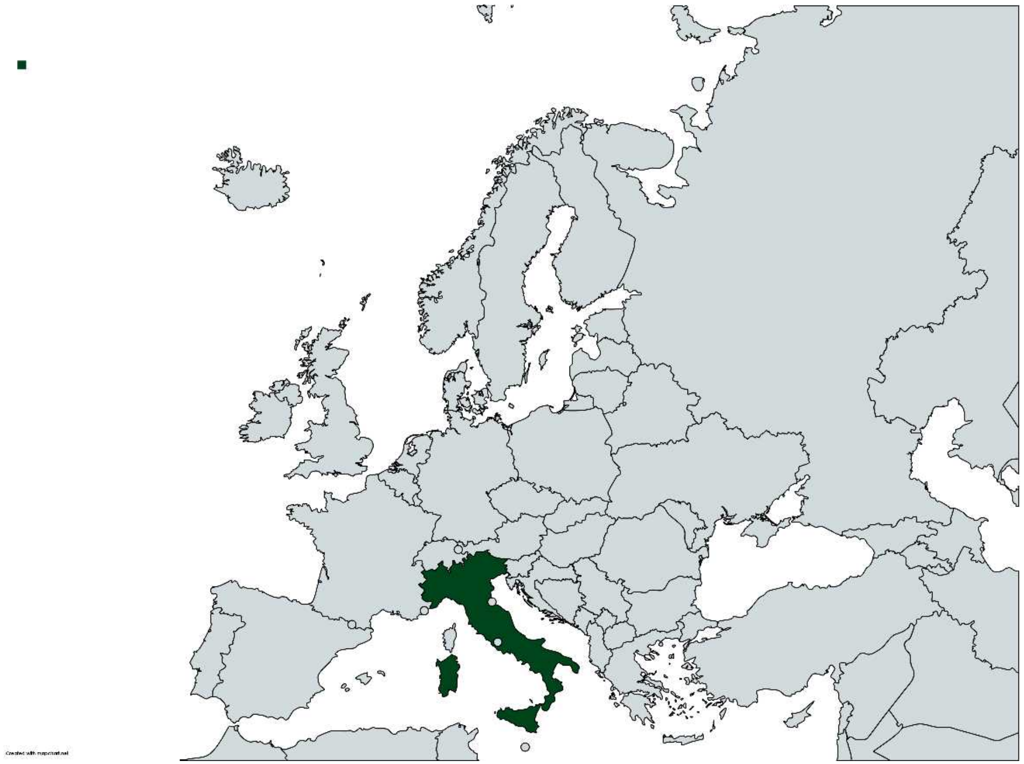

| Country | Italy |
|---|---|

Country-specific data

| Country characteristics | | |
|---|---|---|
| | **Method of payment** | |
| | Data | The Sistema Sanitario Nazionale pays cost of drugs. |
| | Time | 2013 |
| | Reference | http://imi-protect.eu/documents/DUinventory_2013_COUNTRIESyear4_Dec2013.pdf |
| | | |
| | **Medication adherence assessed and reported on the national level** | |
| | Data | Yes |
| | Time | 2023 |
| | Reference | https://www.aifa.gov.it/-/l-uso-dei-farmaci-in-italia-rapporto-osmed-2021 |
| | | |
| | **Health care provider** | |
| | Data | Public. All residnets have access to essential level of care defined by the government and provided by the regions. Regions are at liberty of providing other health services to their residents, but must finance these own-source revenues. |
| | Time | 2013 |
| | Reference | http://imi-protect.eu/documents/DUinventory_2013_COUNTRIESyear4_Dec2013.pdf |
| | | |
| | **Model of healthcare system financing** | |
| | Data | National and regional taxes 877.9%) and by patient compayments (19.7%). |
| | Time | 2013 |
| | Reference | http://imi-protect.eu/documents/DUinventory_2013_COUNTRIESyear4_Dec2013.pdf |
| | | |
| | **Proportion of population aged 65 years and over** | |
| | Data - % of persons | 23.5 |
| | Time | 2021 |
| | Reference | https://ec.europa.eu/eurostat/databrowser/view/TPS00028/default/table?lang=en&category=demo.demo_ind / |
| | | |
| | **Country population (projection)** | |
| | Data - N of persons | 60286529 |
| | Time | 2020 |
| | Reference | https://ec.europa.eu/eurostat/databrowser/view/CENS_HNMGA/default/table?lang=en&category=cens.cens_hn.cens_hnstr |

| | | |
|---|---|---|
| Social/economic factors | **Patient co-payment** | |
| | Data | Class A drugs are fully reimbursed and patient does not pay. Class C are not reimbursedin the outpatient sector expect for war veternas (100% copayment). Medicines in the inpatient sector are fully reimbursed. Regional variations in the reimbursement scheme. |
| | Time | 2013 |
| | Reference | http://imi-protect.eu/documents/DUinventory_2013_COUNTRIESyear4_Dec2013.pdf |
| | | |
| | **Percentage of prescriptions dispensed at no cost to patients** | |
| | Data - % of prescriptions | NA |
| | Time | NA |
| | Reference | NA |
| | | |
| | **Population coverage** | |
| | Data | 100%, around 15% of the population have private health insurance |
| | Time | 2013 |
| | Reference | http://imi-protect.eu/documents/DUinventory_2013_COUNTRIESyear4_Dec2013.pdf |
| | | |
| | **Availability of doctors' services for citizens at no payment** | |
| | Data | The National Health Service guarantees, through its own services and through contracted doctors and paediatricians, the outpatient and home management of acute and chronic diseases in accordance with best practice and in agreement with the patient, including interventions and actions for global health promotion and protection at no payment. |
| | Time | 2023 |
| | Reference | https://www.gazzettaufficiale.it/eli/id/2017/03/18/17A02015/sg |
| | | |
| Therapy-related factors | **Average number of medicines per patient** | |
| | Data - N of medicines per patient | NA |
| | Time | NA |
| | Reference | NA |
| | | |
| | **Proportion of 75 years and over who are taking more than 5 medications concurrently** | |
| | Data - % of persons | 44.5 |
| | Time | 2017 |
| | Reference | https://stats.oecd.org/Index.aspx?ThemeTreeId=27 |
| | | |

| | | |
|---|---|---|
| | **Percentage of self-reported use of prescribed medicines** | |
| | Data - % of persons | 37.7 |
| | Time | 2019 |
| | Reference | https://ec.europa.eu/eurostat/databrowser/view/HLTH_EHIS_MD1E__custom_3764895/default/table?lang=en / |

| | | |
|---|---|---|
| **Patient-related factors** | **Percentage of persons reporting a chronic disease** | |
| | Data - Asthma, % of persons | 4.6 |
| | Data - Chronic lower respiratory diseases, % of persons | 4.3 |
| | Data - High blood pressure, % of persons | 20.4 |
| | Data - Diabetes, % of persons | 6.5 |
| | Data - Chronic depression, % of persons | 5.3 |
| | Time | 2019 |
| | Reference | https://ec.europa.eu/eurostat/databrowser/view/HLTH_EHIS_CD1E/default/table?lang=en&category=hlth.hlth_state.hlth_srcm / |
| | | |
| | **Percentage of self-perceived health - very good (16 years and over)** | |
| | Data - % of persons | 18.7 |
| | Time | 2021 |
| | Reference | https://ec.europa.eu/eurostat/databrowser/view/HLTH_SILC_02/default/table?lang=en&category=hlth.hlth_state.hlth_sph/ |
| | | |
| | **Percenatge of persons with current depressive symptoms** | |
| | Data - % of persons | 4.2 |
| | Time | 2019 |
| | Reference | https://ec.europa.eu/eurostat/databrowser/view/HLTH_EHIS_MH1E/default/table?lang=en&category=hlth.hlth_state.hlth_sph / |

| | | |
|---|---|---|
| **Condition-related factor[illegible]** | **General health literacy** | |
| | Data - Inadequate health literacy, % of persons | 23 |
| | Data - Problematic health literacy, % of persons | 35 |
| | Data - Sufficient health literacy, % of persons | 34 |
| | Data - Excellent health literacy, % of persons | 9 |
| | Time | 2021 |
| | Reference | https://m-pohl.net/Int_Report_methdology_results_recommendations |

| | | |
|---|---|---|
| **Healthcare system-relat[illegible]** | **Percentage of patients receiving adherence interventions** | |

| | |
|---|---|
| Data - % of persons | NA |
| Time | NA |
| Reference | NA |
| | |
| **Nationwide availability of e-prescription** | |
| Data | Yes |
| Time | 2023 |
| Reference | https://www.gazzettaufficiale.it/eli/id/2011/11/12/11A14746/sg |
| | |
| **Waiting time for prescriptions / medical appointments** | |
| Data | The National Health System must guarantee a service in 72 hours if urgent, within 10 days if there is a "short" code, within 30 days for a visit and 60 for an examination if it is deferable, and again within 120 if they are scheduled |
| Time | 2021 |
| Reference | https://www.salute.gov.it/portale/listeAttesa/dettaglioPubblicazioniListeAttesa.jsp?lingua=italiano&id=2824 |
| | |
| **Number of practising physicians per 100,000 inhabitants** | |
| Data - N of practisisng physicians per 100,000 inhabitants | 412.51 |
| Time | 2021 |
| Reference | https://ec.europa.eu/eurostat/databrowser/view/TPS00044/default/table?lang=en&category=hlth.hlth_care.hlth_res.hlth_staff%20%2F |
| | |
| **Proportion of health care expenditure on pharmaceuticals** | |
| Data - % of health care expenditure | 17.538 |
| Time | 2021 |
| Reference | https://data.oecd.org/healthres/pharmaceutical-spending.htm |
| | |
| **Number of practising pharmacists per 100,000 inhabitants** | |
| Data - N of practisisng pharmacists per 100,000 inhabitants | 124.19 |
| Time | 2021 |
| Reference | https://ec.europa.eu/eurostat/databrowser/view/HLTH_RS_PRS1__custom_4104351/default/table?lang=en |
| | |
| **Total health care expenditure as percentage of GDP** | |
| Data - % of GDP | 9.63 |
| Time | 2020 |

| | | |
|---|---|---|
| | Reference | https://ec.europa.eu/eurostat/databrowser/view/TPS00207/default/table?lang=en&category=hlth.hlth_care.hlth_sha11.hlth_sha11_sum |
| | | |
| | **Public pharmaceutical expenditure as percentage of total pharmaceutical expenditure** | |
| | Data - % of total pharmaceutical expenditure | 61.9 |
| | Time | 2019 |
| | Reference | https://gateway.euro.who.int/en/indicators/hfa_580-6790-public-pharmaceutical-expenditure-as-of-total-pharmaceutical-expenditure/visualizations/#id=19675&tab=table |
| | | |
| | **Self-reported consultations of a medical doctor*** | |
| | Data - No contact, % of population according to the number of consultations of a medical doctor in the past 4 weeks | 63.8 |
| | Data - 1 contact, % of population according to the number of consultations of a medical doctor in the past 4 weeks | 18.5 |
| | Data - 2 contacts, % of population according to the number of consultations of a medical doctor in the past 4 weeks | 9.6 |
| | Data - 3 or more contacts, % of population according to the number of consultations of a medical doctor in the past 4 weeks | 8.2 |
| | Time | 2019 |
| | Reference | https://ec.europa.eu/eurostat/databrowser/view/HLTH_EHIS_AM2U/default/table?lang=en&category=hlth.hlth_care.hlth_consult / |

*Medical doctors include generalist medical practitioners and specialist medical practitioners

LATVIA

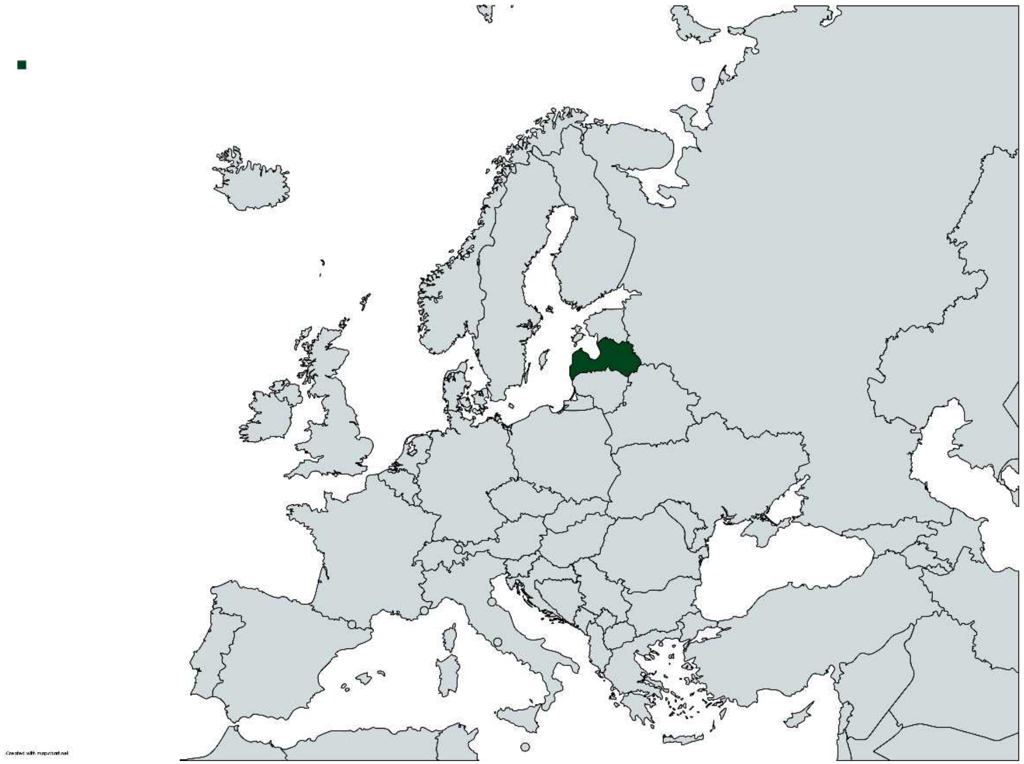

| Country | Latvia |
|---|---|

Country-specific data

| Country characteristics | Method of payment | |
|---|---|---|
| | Data | The National Health Service though their regional branches pays partially the cost of drugs. |
| | Time | 2013 |
| | Reference | http://imi-protect.eu/documents/DUinventory_2013_COUNTRIESyear4_Dec2013.pdf |
| | | |
| | Medication adherence assessed and reported on the national level | |
| | Data | No |
| | Time | 2022 |
| | Reference | NA |
| | | |
| | Health care provider | |
| | Data | Uniersal access to healthcare. The NHS is a state-run, state-budget funded organization under the control of the MoH, which allocates public health care funds and contracts health care providers. Providers contracting with the NHS may be public or private. Surce: Latvia Health System Review 2019 https://apps.who.int/iris/bitstream/handle/10665/331419/HiT-21-4-2019-eng.pdf?ua=1 |
| | Time | 2019 |
| | Reference | https://apps.who.int/iris/bitstream/handle/10665/331419/HiT-21-4-2019-eng.pdf?ua=1 |
| | | |
| | Model of healthcare system financing | |
| | Data | The Latvian National Health System is currently based on general tax financed statutory health care provision, with a purchaser–provider split and a mix of public and private providers. The central government raises resources for the statutory health care system, mainly through general taxation and a small part through social tax Surce: Latvia Health System Review 2019 https://apps.who.int/iris/bitstream/handle/10665/331419/HiT-21-4-2019-eng.pdf?ua=1 |
| | Time | 2019 |
| | Reference | https://apps.who.int/iris/bitstream/handle/10665/331419/HiT-21-4-2019-eng.pdf?ua=1 |
| | | |
| | Proportion of population aged 65 years and over | |
| | Data - % of persons | 20.8 |
| | Time | 2021 |
| | Reference | https://ec.europa.eu/eurostat/databrowser/view/TPS00028/default/table?lang=en&category=demo.demo_ind / |
| | | |

| | | |
|---|---|---|
| | **Country population (projection)** | |
| | Data - N of persons | 1907094 |
| | Time | 2020 |
| | Reference | https://ec.europa.eu/eurostat/databrowser/view/CENS_HNMGA/default/table?lang=en&category=cens.cens_hn.cens_hnstr |

| | | |
|---|---|---|
| Social/economic factors | **Patient co-payment** | |
| | Data | For most severe diagnoses pateints do not pay for the medicines. Otherwise, there are 4 different levels of co-payment: 0%, 25%, 50%, and 100%. In adition, generic substitution is compulsory: if a patient refuses, it is added 10%. |
| | Time | 2013 |
| | Reference | http://imi-protect.eu/documents/DUinventory_2013_COUNTRIESyear4_Dec2013.pdf |
| | | |
| | **Percentage of prescriptions dispensed at no cost to patients** | |
| | Data - % of prescriptions | NA |
| | Time | NA |
| | Reference | NA |
| | | |
| | **Population coverage** | |
| | Data | 1 |
| | Time | 2013 |
| | Reference | http://imi-protect.eu/documents/DUinventory_2013_COUNTRIESyear4_Dec2013.pdf |
| | | |
| | **Availability of doctors' services for citizens at no payment** | |
| | Data | NA |
| | Time | NA |
| | Reference | NA |

| | | |
|---|---|---|
| Therapy-related factors | **Average number of medicines per patient** | |
| | Data - N of medicines per patient | NA |
| | Time | NA |
| | Reference | NA |
| | | |
| | **Proportion of 75 years and over who are taking more than 5 medications concurrently** | |
| | Data - % of persons | NA |
| | Time | NA |

| | | |
|---|---|---|
| | Reference | NA |
| | | |
| | **Percentage of self-reported use of prescribed medicines** | |
| | Data - % of persons | 46.2 |
| | Time | 2019 |
| | Reference | https://ec.europa.eu/eurostat/databrowser/view/HLTH_EHIS_MD1E__custom_3764895/default/table?lang=en / |

| | | |
|---|---|---|
| **Patient-related factors** | **Percentage of persons reporting a chronic disease** | |
| | Data - Asthma, % of persons | 3.8 |
| | Data - Chronic lower respiratory diseases, % of persons | 3.6 |
| | Data - High blood pressure, % of persons | 31.7 |
| | Data - Diabetes, % of persons | 5.7 |
| | Data - Chronic depression, % of persons | 8.4 |
| | Time | 2019 |
| | Reference | https://ec.europa.eu/eurostat/databrowser/view/HLTH_EHIS_CD1E/default/table?lang=en&category=hlth.hlth_state.hlth_srcm / |
| | | |
| | **Percentage of self-perceived health - very good (16 years and over)** | |
| | Data - % of persons | 5.3 |
| | Time | 2021 |
| | Reference | https://ec.europa.eu/eurostat/databrowser/view/HLTH_SILC_02/default/table?lang=en&category=hlth.hlth_state.hlth_sph/ |
| | | |
| | **Percenatge of persons with current depressive symptoms** | |
| | Data - % of persons | 5.7 |
| | Time | 2019 |
| | Reference | https://ec.europa.eu/eurostat/databrowser/view/HLTH_EHIS_MH1E/default/table?lang=en&category=hlth.hlth_state.hlth_sph / |

| | | |
|---|---|---|
| **Condition-related factor[illegible]** | **General health literacy** | |
| | Data - Inadequate health literacy, % of persons | NA |
| | Data - Problematic health literacy, % of persons | NA |
| | Data - Sufficient health literacy, % of persons | NA |
| | Data - Excellent health literacy, % of persons | NA |
| | Time | NA |
| | Reference | NA |

| | | |
|---|---|---|
| Healthcare system-relat | **Percentage of patients receiving adherence interventions** | |
| | Data - % of persons | NA |
| | Time | NA |
| | Reference | NA |
| | | |
| | **Nationwide availability of e-prescription** | |
| | Data | Yes |
| | Time | 2020 |
| | Reference | Expert opinion |
| | | |
| | **Waiting time for prescriptions / medical appointments** | |
| | Data | NA |
| | Time | NA |
| | Reference | NA |
| | | |
| | **Number of practising physicians per 100,000 inhabitants** | |
| | Data - N of practisisng physicians per 100,000 inhabitants | 333.92 |
| | Time | 2020 |
| | Reference | https://ec.europa.eu/eurostat/databrowser/view/TPS00044/default/table?lang=en&category=hlth.hlth_care.hlth_res.hlth_staff%20%2F |
| | | |
| | **Proportion of health care expenditure on pharmaceuticals** | |
| | Data - % of health care expenditure | 23.159 |
| | Time | 2020 |
| | Reference | https://data.oecd.org/healthres/pharmaceutical-spending.htm |
| | | |
| | **Number of practising pharmacists per 100,000 inhabitants** | |
| | Data - N of practisisng pharmacists per 100,000 inhabitants | 87.21 |
| | Time | 2021 |
| | Reference | https://ec.europa.eu/eurostat/databrowser/view/HLTH_RS_PRS1__custom_4104351/default/table?lang=en |
| | | |
| | **Total health care expenditure as percentage of GDP** | |
| | Data - % of GDP | 7.45 |

| | | |
|---|---|---|
| | Time | 2020 |
| | Reference | https://ec.europa.eu/eurostat/databrowser/view/TPS00207/default/table?lang=en&category=hlth.hlth_care.hlth_sha11.hlth_sha11_sum |
| | | |
| | **Public pharmaceutical expenditure as percentage of total pharmaceutical expenditure** | |
| | Data - % of total pharmaceutical expenditure | 39.4 |
| | Time | 2019 |
| | Reference | https://gateway.euro.who.int/en/indicators/hfa_580-6790-public-pharmaceutical-expenditure-as-of-total-pharmaceutical-expenditure/visualizations/#id=19675&tab=table |
| | | |
| | **Self-reported consultations of a medical doctor*** | |
| | Data - No contact, % of population according to the number of consultations of a medical doctor in the past 4 weeks | 66.5 |
| | Data - 1 contact, % of population according to the number of consultations of a medical doctor in the past 4 weeks | 20.7 |
| | Data - 2 contacts, % of population according to the number of consultations of a medical doctor in the past 4 weeks | 9.3 |
| | Data - 3 or more contacts, % of population according to the number of consultations of a medical doctor in the past 4 weeks | 3.5 |
| | Time | 2019 |
| | Reference | https://ec.europa.eu/eurostat/databrowser/view/HLTH_EHIS_AM2U/default/table?lang=en&category=hlth.hlth_care.hlth_consult / |

*Medical doctors include generalist medical practitioners and specialist medical practitioners

LITHUANIA

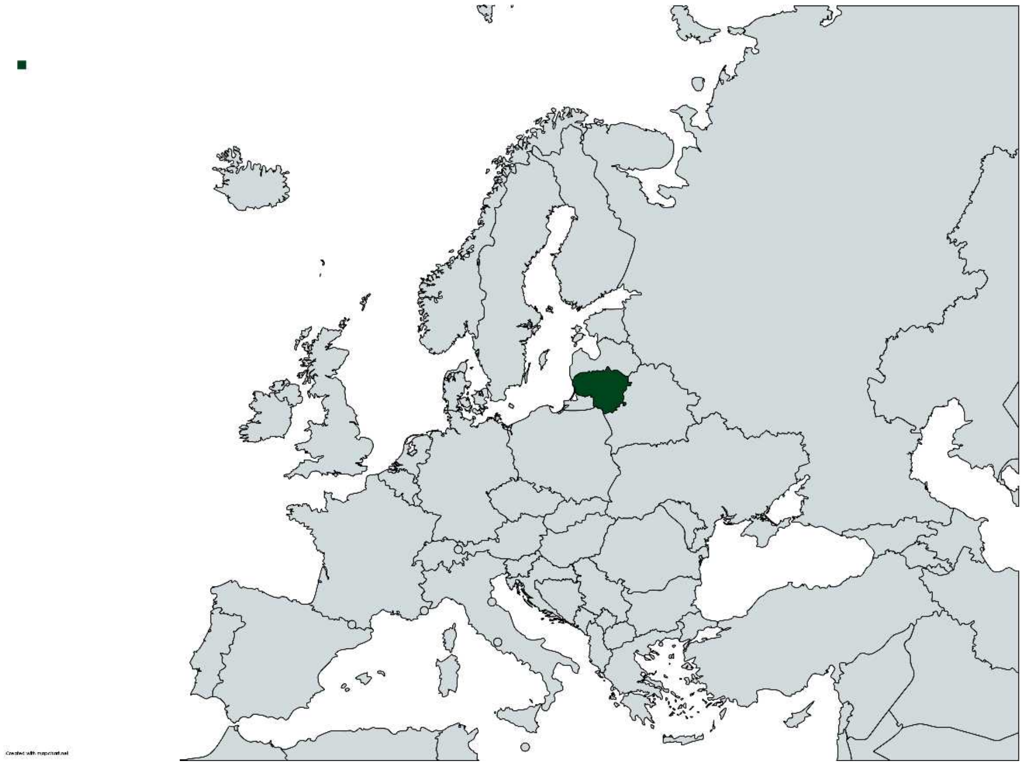

| Country | Lithuania |
|---|---|

Country-specific data

| Country characteristics | Method of payment | |
|---|---|---|
| | Data | National Health Insurance Fund under the Ministry of Health |
| | Time | 2023 |
| | Reference | https://ligoniukasa.lrv.lt/en/ |
| | | |
| | **Medication adherence assessed and reported on the national level** | |
| | Data | No |
| | Time | 2022 |
| | Reference | NA |
| | | |
| | **Health care provider** | |
| | Data | Public, funded by the national Health Insurance Fund (NHIF) through a national health insurance scheme and based on compulsory participation. |
| | Time | 2021 |
| | Reference | https://www.oecd.org/publications/lithuania-country-health-profile-2021-20b64b36-en.htm |
| | | |
| | **Model of healthcare system financing** | |
| | Data | 70% of health spending was publicly financed in 2020, with the remaining third coming from private sources – mainly out-of-pocket (OOP) payments. |
| | Time | 2022 |
| | Reference | https://read.oecd-ilibrary.org/social-issues-migration-health/health-at-a-glance-europe-2022_507433b0-en#page135 |
| | | |
| | **Proportion of population aged 65 years and over** | |
| | Data - % of persons | 19.9 |
| | Time | 2021 |
| | Reference | https://ec.europa.eu/eurostat/databrowser/view/TPS00028/default/table?lang=en&category=demo.demo_ind / |
| | | |
| | **Country population (projection)** | |
| | Data - N of persons | 2793592 |
| | Time | 2020 |

| | | |
|---|---|---|
| | Reference | https://ec.europa.eu/eurostat/databrowser/view/CENS_HNMGA/default/table?lang=en&category=cens.cens_hn.cens_hnstr |

| | | |
|---|---|---|
| Social/economic factors | **Patient co-payment** | |
| | Data | In July 2020, the government broadened the population groups entitled to 100 % reimbursement for listed medicines and medical products. As well as children under 18, people with severe disabilities and retired people on low incomes, this group now also includes everyone aged over 75 years. Other population groups (recipients of the retirement pension and people with less severe disabilities) remain entitled to 50 % reimbursement. Further exemptions from full user fees include 50 % or 100 % reimbursement for medicines for the treatment of specific conditions |
| | Time | 2021 |
| | Reference | https://www.oecd.org/publications/lithuania-country-health-profile-2021-20b64b36-en.htm |
| | | |
| | **Percentage of prescriptions dispensed at no cost to patients** | |
| | Data - % of prescriptions | NA |
| | Time | NA |
| | Reference | NA |
| | | |
| | **Population coverage** | |
| | Data | 1 |
| | Time | 2021 |
| | Reference | https://www.oecd.org/publications/lithuania-country-health-profile-2021-20b64b36-en.htm |
| | | |
| | **Availability of doctors' services for citizens at no payment** | |
| | Data | NA |
| | Time | NA |
| | Reference | NA |
| | | |
| Therapy-related factors | **Average number of medicines per patient** | |
| | Data - N of medicines per patient | NA |
| | Time | NA |
| | Reference | NA |
| | | |
| | **Proportion of 75 years and over who are taking more than 5 medications concurrently** | |
| | Data - % of persons | NA |
| | Time | NA |

| | | |
|---|---|---|
| | Reference | NA |
| | | |
| | **Percentage of self-reported use of prescribed medicines** | |
| | Data - % of persons | 43.3 |
| | Time | 2019 |
| | Reference | https://ec.europa.eu/eurostat/databrowser/view/HLTH_EHIS_MD1E__custom_3764895/default/table?lang=en / |

| | | |
|---|---|---|
| **Patient-related factors** | **Percentage of persons reporting a chronic disease** | |
| | Data - Asthma, % of persons | 2.8 |
| | Data - Chronic lower respiratory diseases, % of persons | 6.4 |
| | Data - High blood pressure, % of persons | 29.9 |
| | Data - Diabetes, % of persons | 5.3 |
| | Data - Chronic depression, % of persons | 7 |
| | Time | 2019 |
| | Reference | https://ec.europa.eu/eurostat/databrowser/view/HLTH_EHIS_CD1E/default/table?lang=en&category=hlth.hlth_state.hlth_srcm / |
| | | |
| | **Percentage of self-perceived health - very good (16 years and over)** | |
| | Data - % of persons | 9.1 |
| | Time | 2021 |
| | Reference | https://ec.europa.eu/eurostat/databrowser/view/HLTH_SILC_02/default/table?lang=en&category=hlth.hlth_state.hlth_sph/ |
| | | |
| | **Percenatge of persons with current depressive symptoms** | |
| | Data - % of persons | 6.2 |
| | Time | 2019 |
| | Reference | https://ec.europa.eu/eurostat/databrowser/view/HLTH_EHIS_MH1E/default/table?lang=en&category=hlth.hlth_state.hlth_sph / |

| | | |
|---|---|---|
| **Condition-related factor** | **General health literacy** | |
| | Data - Inadequate health literacy, % of persons | NA |
| | Data - Problematic health literacy, % of persons | NA |
| | Data - Sufficient health literacy, % of persons | NA |
| | Data - Excellent health literacy, % of persons | NA |
| | Time | NA |
| | Reference | NA |

| | | |
|---|---|---|
| Healthcare system-relat | **Percentage of patients receiving adherence interventions** | |
| | Data - % of persons | NA |
| | Time | NA |
| | Reference | NA |
| | | |
| | **Nationwide availability of e-prescription** | |
| | Data | Yes |
| | Time | 2021 |
| | Reference | https://www.oecd-ilibrary.org/sites/507433b0-en/1/3/2/6/5/index.html?itemId=/content/publication/507433b0-en&_csp_=69a1197833db420974a2940c01518500&itemIGO=oecd&itemContentType=book |
| | | |
| | **Waiting time for prescriptions / medical appointments** | |
| | Data | NA |
| | Time | NA |
| | Reference | NA |
| | | |
| | **Number of practising physicians per 100,000 inhabitants** | |
| | Data - N of practisisng physicians per 100,000 inhabitants | 448.28 |
| | Time | 2020 |
| | Reference | https://ec.europa.eu/eurostat/databrowser/view/TPS00044/default/table?lang=en&category=hlth.hlth_care.hlth_res.hlth_staff%20%2F |
| | | |
| | **Proportion of health care expenditure on pharmaceuticals** | |
| | Data - % of health care expenditure | 21.599 |
| | Time | 2020 |
| | Reference | https://data.oecd.org/healthres/pharmaceutical-spending.htm |
| | | |
| | **Number of practising pharmacists per 100,000 inhabitants** | |
| | Data - N of practisisng pharmacists per 100,000 inhabitants | 102.62 |
| | Time | 2020 |
| | Reference | https://ec.europa.eu/eurostat/databrowser/view/HLTH_RS_PRS1__custom_4104351/default/table?lang=en |
| | | |
| | **Total health care expenditure as percentage of GDP** | |

| | | |
|---|---|---|
| | Data - % of GDP | 7.54 |
| | Time | 2020 |
| | Reference | https://ec.europa.eu/eurostat/databrowser/view/TPS00207/default/table?lang=en&category=hlth.hlth_care.hlth_sha11.hlth_sha11_sum |
| | | |
| | **Public pharmaceutical expenditure as percentage of total pharmaceutical expenditure** | |
| | Data - % of total pharmaceutical expenditure | 45.2 |
| | Time | 2020 |
| | Reference | https://gateway.euro.who.int/en/indicators/hfa_580-6790-public-pharmaceutical-expenditure-as-of-total-pharmaceutical-expenditure/visualizations/#id=19675&tab=table |
| | | |
| | **Self-reported consultations of a medical doctor*** | |
| | Data - No contact, % of population according to the number of consultations of a medical doctor in the past 4 weeks | 62.6 |
| | Data - 1 contact, % of population according to the number of consultations of a medical doctor in the past 4 weeks | 21.3 |
| | Data - 2 contacts, % of population according to the number of consultations of a medical doctor in the past 4 weeks | 10.6 |
| | Data - 3 or more contacts, % of population according to the number of consultations of a medical doctor in the past 4 weeks | 5.5 |
| | Time | 2019 |
| | Reference | https://ec.europa.eu/eurostat/databrowser/view/HLTH_EHIS_AM2U/default/table?lang=en&category=hlth.hlth_care.hlth_consult / |

*Medical doctors include generalist medical practitioners and specialist medical practitioners

# LUXEMBOURG

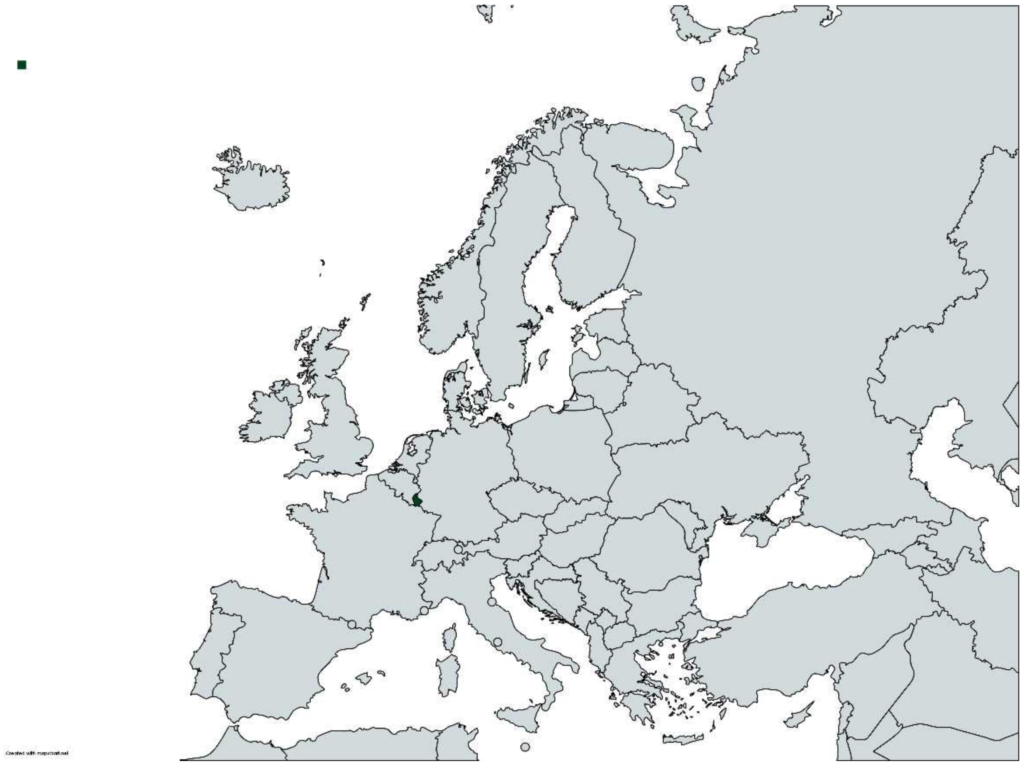

| Country | Luxembourg |
|---|---|

Country-specific data

| Country characteristics | Method of payment | |
|---|---|---|
| | Data | NA |
| | Time | NA |
| | Reference | NA |
| | | |
| | **Medication adherence assessed and reported on the national level** | |
| | Data | No |
| | Time | 2022 |
| | Reference | NA |
| | | |
| | **Health care provider** | |
| | Data | NA |
| | Time | NA |
| | Reference | NA |
| | | |
| | **Model of healthcare system financing** | |
| | Data | NA |
| | Time | NA |
| | Reference | NA |
| | | |
| | **Proportion of population aged 65 years and over** | |
| | Data - % of persons | 14.6 |
| | Time | 2021 |
| | Reference | https://ec.europa.eu/eurostat/databrowser/view/TPS00028/default/table?lang=en&category=demo.demo_ind / |
| | | |
| | **Country population (projection)** | |
| | Data - N of persons | 626031 |
| | Time | 2020 |
| | Reference | https://ec.europa.eu/eurostat/databrowser/view/CENS_HNMGA/default/table?lang=en&category=cens.cens_hn.cens_hnstr |

| | | |
|---|---|---|
| Social/economic factors | **Patient co-payment** | |
| | Data | NA |
| | Time | NA |
| | Reference | NA |
| | | |
| | **Percentage of prescriptions dispensed at no cost to patients** | |
| | Data - % of prescriptions | NA |
| | Time | NA |
| | Reference | NA |
| | | |
| | **Population coverage** | |
| | Data | NA |
| | Time | NA |
| | Reference | NA |
| | | |
| | **Availability of doctors' services for citizens at no payment** | |
| | Data | NA |
| | Time | NA |
| | Reference | NA |
| | | |
| Therapy-related factors | **Average number of medicines per patient** | |
| | Data - N of medicines per patient | NA |
| | Time | NA |
| | Reference | NA |
| | | |
| | **Proportion of 75 years and over who are taking more than 5 medications concurrently** | |
| | Data - % of persons | 87 |
| | Time | 2016 |
| | Reference | https://stats.oecd.org/Index.aspx?ThemeTreeId=30 |
| | | |
| | **Percentage of self-reported use of prescribed medicines** | |
| | Data - % of persons | 54.2 |

| | | |
|---|---|---|
| | Time | 2019 |
| | Reference | https://ec.europa.eu/eurostat/databrowser/view/HLTH_EHIS_MD1E__custom_3764895/default/table?lang=en / |

| | | |
|---|---|---|
| **Patient-related factors** | **Percentage of persons reporting a chronic disease** | |
| | Data - Asthma, % of persons | 6 |
| | Data - Chronic lower respiratory diseases, % of persons | 5 |
| | Data - High blood pressure, % of persons | 15.5 |
| | Data - Diabetes, % of persons | 4.6 |
| | Data - Chronic depression, % of persons | 10 |
| | Time | 2019 |
| | Reference | https://ec.europa.eu/eurostat/databrowser/view/HLTH_EHIS_CD1E/default/table?lang=en&category=hlth.hlth_state.hlth_srcm / |
| | | |
| | **Percentage of self-perceived health - very good (16 years and over)** | |
| | Data - % of persons | 23.7 |
| | Time | 2021 |
| | Reference | https://ec.europa.eu/eurostat/databrowser/view/HLTH_SILC_02/default/table?lang=en&category=hlth.hlth_state.hlth_sph/ |
| | | |
| | **Percenatge of persons with current depressive symptoms** | |
| | Data - % of persons | 8.8 |
| | Time | 2019 |
| | Reference | https://ec.europa.eu/eurostat/databrowser/view/HLTH_EHIS_MH1E/default/table?lang=en&category=hlth.hlth_state.hlth_sph / |

| | | |
|---|---|---|
| **Condition-related factor** | **General health literacy** | |
| | Data - Inadequate health literacy, % of persons | NA |
| | Data - Problematic health literacy, % of persons | NA |
| | Data - Sufficient health literacy, % of persons | NA |
| | Data - Excellent health literacy, % of persons | NA |
| | Time | NA |
| | Reference | NA |

| | | |
|---|---|---|
| **Healthcare system-relat** | **Percentage of patients receiving adherence interventions** | |
| | Data - % of persons | NA |
| | Time | NA |

| | |
|---|---|
| Reference | NA |
| | |
| **Nationwide availability of e-prescription** | |
| Data | NA |
| Time | NA |
| Reference | NA |
| | |
| **Waiting time for prescriptions / medical appointments** | |
| Data | NA |
| Time | NA |
| Reference | NA |
| | |
| **Number of practising physicians per 100,000 inhabitants** | |
| Data - N of practisisng physicians per 100,000 inhabitants | 298.49 |
| Time | 2017 |
| Reference | https://ec.europa.eu/eurostat/databrowser/view/TPS00044/default/table?lang=en&category=hlth.hlth_care.hlth_res.hlth_staff%20%2F |
| | |
| **Proportion of health care expenditure on pharmaceuticals** | |
| Data - % of health care expenditure | 10.485 |
| Time | 2020 |
| Reference | https://data.oecd.org/healthres/pharmaceutical-spending.htm |
| | |
| **Number of practising pharmacists per 100,000 inhabitants** | |
| Data - N of practisisng pharmacists per 100,000 inhabitants | 69.76 |
| Time | 2017 |
| Reference | https://ec.europa.eu/eurostat/databrowser/view/HLTH_RS_PRS1__custom_4104351/default/table?lang=en |
| | |
| **Total health care expenditure as percentage of GDP** | |
| Data - % of GDP | 5.77 |
| Time | 2020 |
| Reference | https://ec.europa.eu/eurostat/databrowser/view/TPS00207/default/table?lang=en&category=hlth.hlth_care.hlth_sha11.hlth_sha11_sum |
| | |

| | | |
|---|---|---|
| | **Public pharmaceutical expenditure as percentage of total pharmaceutical expenditure** | |
| | Data - % of total pharmaceutical expenditure | NA |
| | Time | NA |
| | Reference | NA |
| | | |
| | **Self-reported consultations of a medical doctor*** | |
| | Data - No contact, % of population according to the number of consultations of a medical doctor in the past 4 weeks | 50 |
| | Data - 1 contact, % of population according to the number of consultations of a medical doctor in the past 4 weeks | 23.5 |
| | Data - 2 contacts, % of population according to the number of consultations of a medical doctor in the past 4 weeks | 14.1 |
| | Data - 3 or more contacts, % of population according to the number of consultations of a medical doctor in the past 4 weeks | 12.4 |
| | Time | 2019 |
| | Reference | https://ec.europa.eu/eurostat/databrowser/view/HLTH_EHIS_AM2U/default/table?lang=en&category=hlth.hlth_care.hlth_consult / |

*Medical doctors include generalist medical practitioners and specialist medical practitioners

MALTA

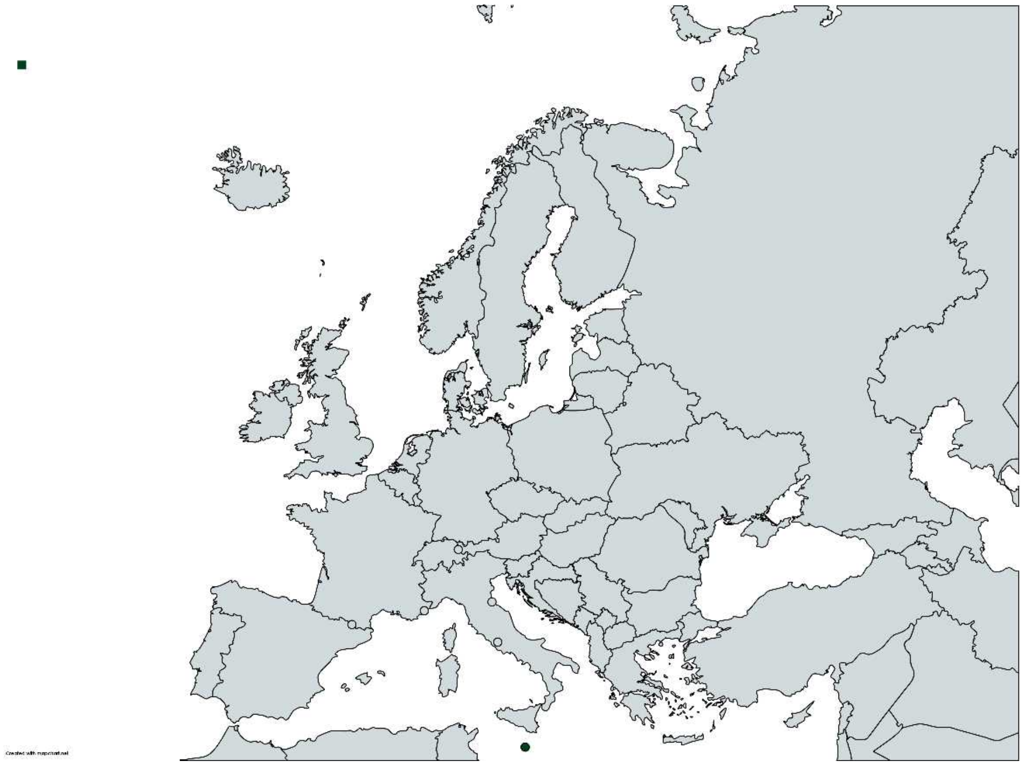

| Country | Malta |
|---|---|

Country-specific data

| Country characteristics | | |
|---|---|---|
| | **Method of payment** | |
| | Data | Certain individals are entitled to free medication as per relevant law |
| | Time | 2023 |
| | Reference | https://www.gov.mt/en/Life%20Events/Pages/Healthy%20Living/Healthcare-entitlement.aspx#:~:text=Maltese%20national%20patients%20are%20entitled,of%20this%20act%20%2D%20Act%20No. |
| | | |
| | **Medication adherence assessed and reported on the national level** | |
| | Data | No |
| | Time | 2022 |
| | Reference | NA |
| | | |
| | **Health care provider** | |
| | Data | Govenment of Malta Ministry of Health |
| | Time | 2023 |
| | Reference | Expert opinion |
| | | |
| | **Model of healthcare system financing** | |
| | Data | NA |
| | Time | NA |
| | Reference | NA |
| | | |
| | **Proportion of population aged 65 years and over** | |
| | Data - % of persons | 18.9 |
| | Time | 2021 |
| | Reference | https://ec.europa.eu/eurostat/databrowser/view/TPS00028/default/table?lang=en&category=demo.demo_ind / |
| | | |
| | **Country population (projection)** | |
| | Data - N of persons | 506951 |
| | Time | 2020 |
| | Reference | https://ec.europa.eu/eurostat/databrowser/view/CENS_HNMGA/default/table?lang=en&category=cens.cens_hn.cens_hnstr |

| | | |
|---|---|---|
| Social/economic factors | **Patient co-payment** | |
| | Data | There is no copayment system |
| | Time | 2023 |
| | Reference | Expert opinion |
| | | |
| | **Percentage of prescriptions dispensed at no cost to patients** | |
| | Data - % of prescriptions | NA |
| | Time | NA |
| | Reference | NA |
| | | |
| | **Population coverage** | |
| | Data | NA |
| | Time | NA |
| | Reference | NA |
| | | |
| | **Availability of doctors' services for citizens at no payment** | |
| | Data | All the population has access to free doctor's services |
| | Time | 2023 |
| | Reference | https://www.gov.mt/en/Life%20Events/Pages/Healthy%20Living/Healthcare-entitlement.aspx#:~:text=Maltese%20national%20patients%20are%20entitled,of%20this%20act%20%2D%20Act%20No. |
| | | |
| Therapy-related factors | **Average number of medicines per patient** | |
| | Data - N of medicines per patient | NA |
| | Time | NA |
| | Reference | NA |
| | | |
| | **Proportion of 75 years and over who are taking more than 5 medications concurrently** | |
| | Data - % of persons | NA |
| | Time | NA |
| | Reference | NA |
| | | |
| | **Percentage of self-reported use of prescribed medicines** | |
| | Data - % of persons | 42.5 |

| | | |
|---|---|---|
| | Time | 2019 |
| | Reference | https://ec.europa.eu/eurostat/databrowser/view/HLTH_EHIS_MD1E__custom_3764895/default/table?lang=en / |

| | | |
|---|---|---|
| **Patient-related factors** | **Percentage of persons reporting a chronic disease** | |
| | Data - Asthma, % of persons | 6 |
| | Data - Chronic lower respiratory diseases, % of persons | 0.6 |
| | Data - High blood pressure, % of persons | 18.1 |
| | Data - Diabetes, % of persons | 7.5 |
| | Data - Chronic depression, % of persons | 3.5 |
| | Time | 2019 |
| | Reference | https://ec.europa.eu/eurostat/databrowser/view/HLTH_EHIS_CD1E/default/table?lang=en&category=hlth.hlth_state.hlth_srcm / |
| | | |
| | **Percentage of self-perceived health - very good (16 years and over)** | |
| | Data - % of persons | 22.1 |
| | Time | 2021 |
| | Reference | https://ec.europa.eu/eurostat/databrowser/view/HLTH_SILC_02/default/table?lang=en&category=hlth.hlth_state.hlth_sph/ |
| | | |
| | **Percenatge of persons with current depressive symptoms** | |
| | Data - % of persons | 8.8 |
| | Time | 2019 |
| | Reference | https://ec.europa.eu/eurostat/databrowser/view/HLTH_EHIS_MH1E/default/table?lang=en&category=hlth.hlth_state.hlth_sph / |

| | | |
|---|---|---|
| **Condition-related factor** | **General health literacy** | |
| | Data - Inadequate health literacy, % of persons | NA |
| | Data - Problematic health literacy, % of persons | NA |
| | Data - Sufficient health literacy, % of persons | NA |
| | Data - Excellent health literacy, % of persons | NA |
| | Time | NA |
| | Reference | NA |

| | | |
|---|---|---|
| **Healthcare system-relat** | **Percentage of patients receiving adherence interventions** | |
| | Data - % of persons | NA |
| | Time | NA |

| | |
|---|---|
| Reference | NA |
| | |
| **Nationwide availability of e-prescription** | |
| Data | NA |
| Time | NA |
| Reference | NA |
| | |
| **Waiting time for prescriptions / medical appointments** | |
| Data | NA |
| Time | NA |
| Reference | NA |
| | |
| **Number of practising physicians per 100,000 inhabitants** | |
| Data - N of practisisng physicians per 100,000 inhabitants | 418.76 |
| Time | 2020 |
| Reference | https://ec.europa.eu/eurostat/databrowser/view/TPS00044/default/table?lang=en&category=hlth.hlth_care.hlth_res.hlth_staff%20%2F |
| | |
| **Proportion of health care expenditure on pharmaceuticals** | |
| Data - % of health care expenditure | 20.342 |
| Time | 2019 |
| Reference | https://data.oecd.org/healthres/pharmaceutical-spending.htm |
| | |
| **Number of practising pharmacists per 100,000 inhabitants** | |
| Data - N of practisisng pharmacists per 100,000 inhabitants | 135.83 |
| Time | 2020 |
| Reference | https://ec.europa.eu/eurostat/databrowser/view/HLTH_RS_PRS1__custom_4104351/default/table?lang=en |
| | |
| **Total health care expenditure as percentage of GDP** | |
| Data - % of GDP | NA |
| Time | NA |
| Reference | NA |
| | |
| **Public pharmaceutical expenditure as percentage of total pharmaceutical expenditure** | |

| | | |
|---|---|---|
| | Data - % of total pharmaceutical expenditure | 30.4 |
| | Time | 2017 |
| | Reference | https://gateway.euro.who.int/en/indicators/hfa_580-6790-public-pharmaceutical-expenditure-as-of-total-pharmaceutical-expenditure/visualizations/#id=19675&tab=table |
| | | |
| | **Self-reported consultations of a medical doctor*** | |
| | Data - No contact, % of population according to the number of consultations of a medical doctor in the past 4 weeks | 68.3 |
| | Data - 1 contact, % of population according to the number of consultations of a medical doctor in the past 4 weeks | 21.3 |
| | Data - 2 contacts, % of population according to the number of consultations of a medical doctor in the past 4 weeks | 6.5 |
| | Data - 3 or more contacts, % of population according to the number of consultations of a medical doctor in the past 4 weeks | 4 |
| | Time | 2019 |
| | Reference | https://ec.europa.eu/eurostat/databrowser/view/HLTH_EHIS_AM2U/default/table?lang=en&category=hlth.hlth_care.hlth_consult / |

*Medical doctors include generalist medical practitioners and specialist medical practitioners

MOLDOVA

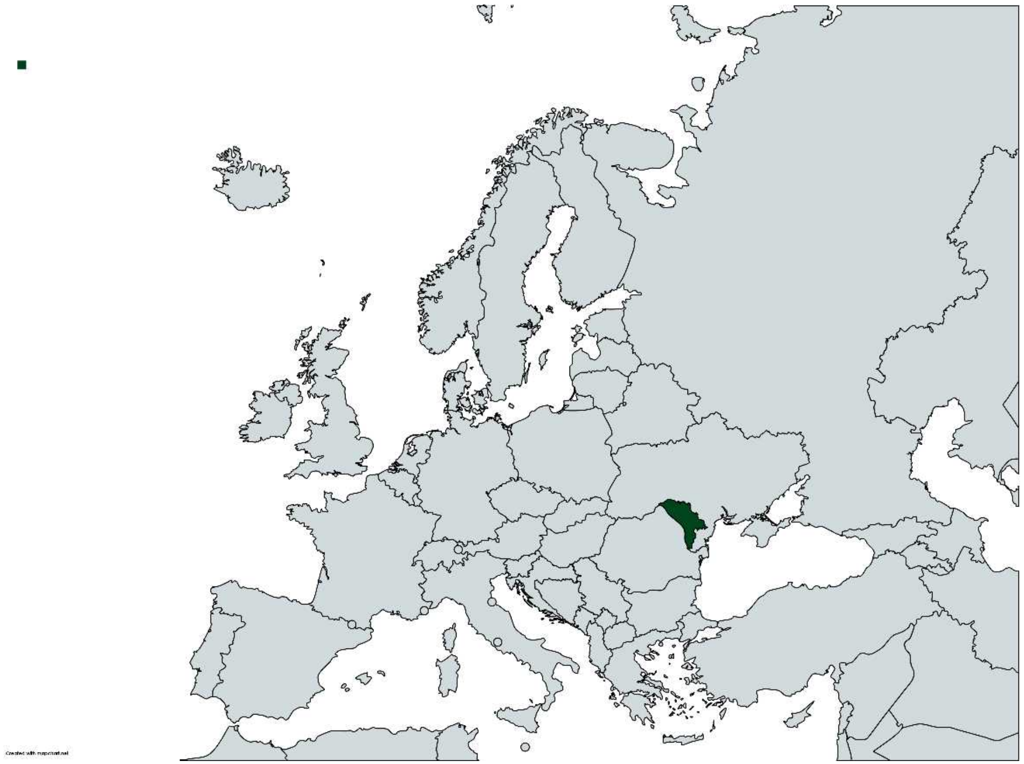

| Country | Moldova |
|---|---|

Country-specific data

| Country characteristics | Method of payment | |
|---|---|---|
| | Data | NA |
| | Time | NA |
| | Reference | NA |
| | | |
| | Medication adherence assessed and reported on the national level | |
| | Data | No |
| | Time | 2022 |
| | Reference | NA |
| | | |
| | Health care provider | |
| | Data | NA |
| | Time | NA |
| | Reference | NA |
| | | |
| | Model of healthcare system financing | |
| | Data | NA |
| | Time | NA |
| | Reference | NA |
| | | |
| | Proportion of population aged 65 years and over | |
| | Data - % of persons | 14.9 |
| | Time | 2021 |
| | Reference | https://ec.europa.eu/eurostat/databrowser/view/TPS00028/default/table?lang=en&category=demo.demo_ind / |
| | | |
| | Country population (projection) | |
| | Data - N of persons | NA |
| | Time | NA |
| | Reference | NA |

| | | |
|---|---|---|
| Social/economic factors | **Patient co-payment** | |
| | Data | NA |
| | Time | NA |
| | Reference | NA |
| | | |
| | **Percentage of prescriptions dispensed at no cost to patients** | |
| | Data - % of prescriptions | NA |
| | Time | NA |
| | Reference | NA |
| | | |
| | **Population coverage** | |
| | Data | NA |
| | Time | NA |
| | Reference | NA |
| | | |
| | **Availability of doctors' services for citizens at no payment** | |
| | Data | NA |
| | Time | NA |
| | Reference | NA |
| | | |
| Therapy-related factors | **Average number of medicines per patient** | |
| | Data - N of medicines per patient | NA |
| | Time | NA |
| | Reference | NA |
| | | |
| | **Proportion of 75 years and over who are taking more than 5 medications concurrently** | |
| | Data - % of persons | NA |
| | Time | NA |
| | Reference | NA |
| | | |
| | **Percentage of self-reported use of prescribed medicines** | |
| | Data - % of persons | NA |

| | | |
|---|---|---|
| | Time | NA |
| | Reference | NA |
| | | |
| **Patient-related factors** | **Percentage of persons reporting a chronic disease** | |
| | Data - Asthma, % of persons | NA |
| | Data - Chronic lower respiratory diseases, % of persons | NA |
| | Data - High blood pressure, % of persons | NA |
| | Data - Diabetes, % of persons | NA |
| | Data - Chronic depression, % of persons | NA |
| | Time | NA |
| | Reference | NA |
| | | |
| | **Percentage of self-perceived health - very good (16 years and over)** | |
| | Data - % of persons | NA |
| | Time | NA |
| | Reference | NA |
| | | |
| | **Percenatge of persons with current depressive symptoms** | |
| | Data - % of persons | NA |
| | Time | NA |
| | Reference | NA |
| | | |
| **Condition-related factor** | **General health literacy** | |
| | Data - Inadequate health literacy, % of persons | NA |
| | Data - Problematic health literacy, % of persons | NA |
| | Data - Sufficient health literacy, % of persons | NA |
| | Data - Excellent health literacy, % of persons | NA |
| | Time | NA |
| | Reference | NA |
| | | |
| **Healthcare system-relat** | **Percentage of patients receiving adherence interventions** | |
| | Data - % of persons | NA |
| | Time | NA |

| | |
|---|---|
| Reference | NA |
| | |
| **Nationwide availability of e-prescription** | |
| Data | NA |
| Time | NA |
| Reference | NA |
| | |
| **Waiting time for prescriptions / medical appointments** | |
| Data | NA |
| Time | NA |
| Reference | NA |
| | |
| **Number of practising physicians per 100,000 inhabitants** | |
| Data - N of practisisng physicians per 100,000 inhabitants | NA |
| Time | NA |
| Reference | NA |
| | |
| **Proportion of health care expenditure on pharmaceuticals** | |
| Data - % of health care expenditure | NA |
| Time | NA |
| Reference | NA |
| | |
| **Number of practising pharmacists per 100,000 inhabitants** | |
| Data - N of practisisng pharmacists per 100,000 inhabitants | NA |
| Time | NA |
| Reference | NA |
| | |
| **Total health care expenditure as percentage of GDP** | |
| Data - % of GDP | NA |
| Time | NA |
| Reference | NA |
| | |
| **Public pharmaceutical expenditure as percentage of total pharmaceutical expenditure** | |

| | | |
|---|---|---|
| | Data - % of total pharmaceutical expenditure | NA |
| | Time | NA |
| | Reference | NA |
| | | |
| | **Self-reported consultations of a medical doctor*** | |
| | Data - No contact, % of population according to the number of consultations of a medical doctor in the past 4 weeks | NA |
| | Data - 1 contact, % of population according to the number of consultations of a medical doctor in the past 4 weeks | NA |
| | Data - 2 contacts, % of population according to the number of consultations of a medical doctor in the past 4 weeks | NA |
| | Data - 3 or more contacts, % of population according to the number of consultations of a medical doctor in the past 4 weeks | NA |
| | Time | NA |
| | Reference | NA |

*Medical doctors include generalist medical practitioners and specialist medical practitioners

MONTENEGRO

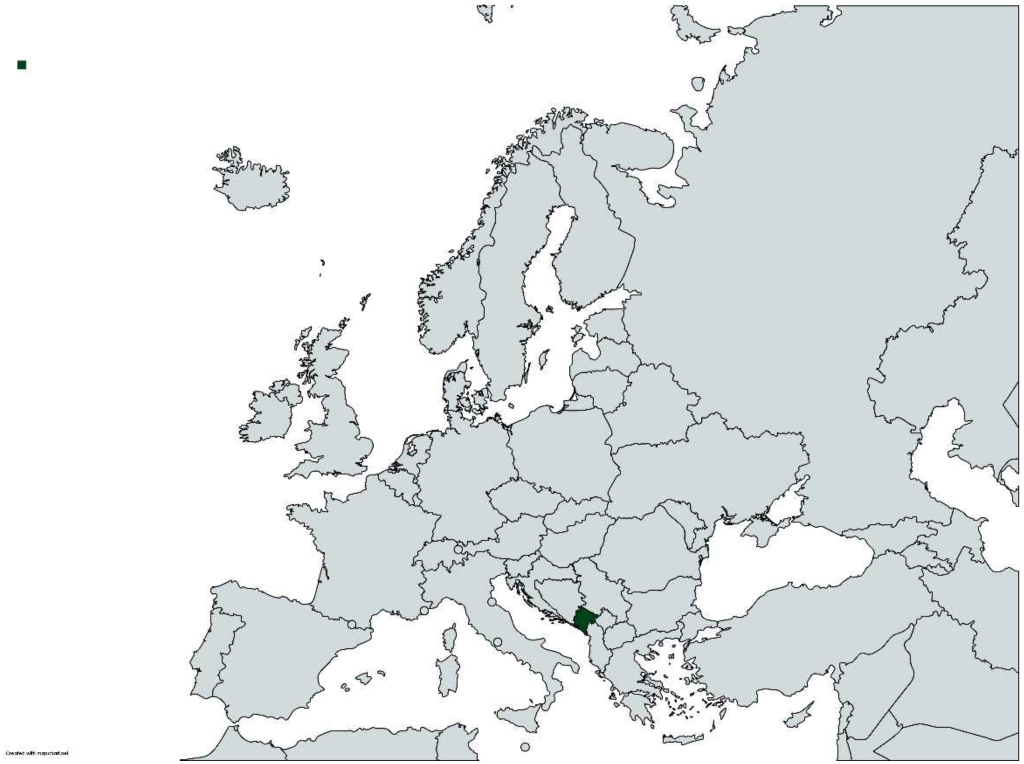

| Country | Montenegro |
|---|---|

Country-specific data

| Country characteristics | Method of payment | |
|---|---|---|
| | Data | NA |
| | Time | NA |
| | Reference | NA |
| | | |
| | Medication adherence assessed and reported on the national level | |
| | Data | No |
| | Time | 2022 |
| | Reference | NA |
| | | |
| | Health care provider | |
| | Data | NA |
| | Time | NA |
| | Reference | NA |
| | | |
| | Model of healthcare system financing | |
| | Data | NA |
| | Time | NA |
| | Reference | NA |
| | | |
| | Proportion of population aged 65 years and over | |
| | Data - % of persons | 15.9 |
| | Time | 2021 |
| | Reference | https://ec.europa.eu/eurostat/databrowser/view/TPS00028/default/table?lang=en&category=demo.demo_ind / |
| | | |
| | Country population (projection) | |
| | Data - N of persons | 619211 |
| | Time | 2021 |
| | Reference | Monstat |

| | | |
|---|---|---|
| Social/economic factors | **Patient co-payment** | |
| | Data | NA |
| | Time | NA |
| | Reference | NA |
| | | |
| | **Percentage of prescriptions dispensed at no cost to patients** | |
| | Data - % of prescriptions | NA |
| | Time | NA |
| | Reference | NA |
| | | |
| | **Population coverage** | |
| | Data | NA |
| | Time | NA |
| | Reference | NA |
| | | |
| | **Availability of doctors' services for citizens at no payment** | |
| | Data | NA |
| | Time | NA |
| | Reference | NA |
| | | |
| Therapy-related factors | **Average number of medicines per patient** | |
| | Data - N of medicines per patient | NA |
| | Time | NA |
| | Reference | NA |
| | | |
| | **Proportion of 75 years and over who are taking more than 5 medications concurrently** | |
| | Data - % of persons | NA |
| | Time | NA |
| | Reference | NA |
| | | |
| | **Percentage of self-reported use of prescribed medicines** | |
| | Data - % of persons | NA |

| | | |
|---|---|---|
| | Time | NA |
| | Reference | NA |

| Patient-related factors | Percentage of persons reporting a chronic disease | |
|---|---|---|
| | Data - Asthma, % of persons | NA |
| | Data - Chronic lower respiratory diseases, % of persons | NA |
| | Data - High blood pressure, % of persons | NA |
| | Data - Diabetes, % of persons | NA |
| | Data - Chronic depression, % of persons | NA |
| | Time | NA |
| | Reference | NA |
| | | |
| | Percentage of self-perceived health - very good (16 years and over) | |
| | Data - % of persons | 39.2 |
| | Time | 2020 |
| | Reference | https://ec.europa.eu/eurostat/databrowser/view/HLTH_SILC_02/default/table?lang=en&category=hlth.hlth_state.hlth_sph/ |
| | | |
| | Percenatge of persons with current depressive symptoms | |
| | Data - % of persons | NA |
| | Time | NA |
| | Reference | NA |

| Condition-related factor | General health literacy | |
|---|---|---|
| | Data - Inadequate health literacy, % of persons | NA |
| | Data - Problematic health literacy, % of persons | NA |
| | Data - Sufficient health literacy, % of persons | NA |
| | Data - Excellent health literacy, % of persons | NA |
| | Time | NA |
| | Reference | NA |

| Healthcare system-relat | Percentage of patients receiving adherence interventions | |
|---|---|---|
| | Data - % of persons | NA |
| | Time | NA |

| | |
|---|---|
| Reference | NA |
| | |
| **Nationwide availability of e-prescription** | |
| Data | Yes |
| Time | 2023 |
| Reference | Expert opinion |
| | |
| **Waiting time for prescriptions / medical appointments** | |
| Data | NA |
| Time | NA |
| Reference | NA |
| | |
| **Number of practising physicians per 100,000 inhabitants** | |
| Data - N of practisisng physicians per 100,000 inhabitants | NA |
| Time | NA |
| Reference | NA |
| | |
| **Proportion of health care expenditure on pharmaceuticals** | |
| Data - % of health care expenditure | NA |
| Time | NA |
| Reference | NA |
| | |
| **Number of practising pharmacists per 100,000 inhabitants** | |
| Data - N of practisisng pharmacists per 100,000 inhabitants | NA |
| Time | NA |
| Reference | NA |
| | |
| **Total health care expenditure as percentage of GDP** | |
| Data - % of GDP | NA |
| Time | NA |
| Reference | NA |
| | |
| **Public pharmaceutical expenditure as percentage of total pharmaceutical expenditure** | |

| | | |
|---|---|---|
| | Data - % of total pharmaceutical expenditure | NA |
| | Time | NA |
| | Reference | NA |
| | | |
| | **Self-reported consultations of a medical doctor*** | |
| | Data - No contact, % of population according to the number of consultations of a medical doctor in the past 4 weeks | NA |
| | Data - 1 contact, % of population according to the number of consultations of a medical doctor in the past 4 weeks | NA |
| | Data - 2 contacts, % of population according to the number of consultations of a medical doctor in the past 4 weeks | NA |
| | Data - 3 or more contacts, % of population according to the number of consultations of a medical doctor in the past 4 weeks | NA |
| | Time | NA |
| | Reference | NA |

*Medical doctors include generalist medical practitioners and specialist medical practitioners

# THE NETHERLANDS

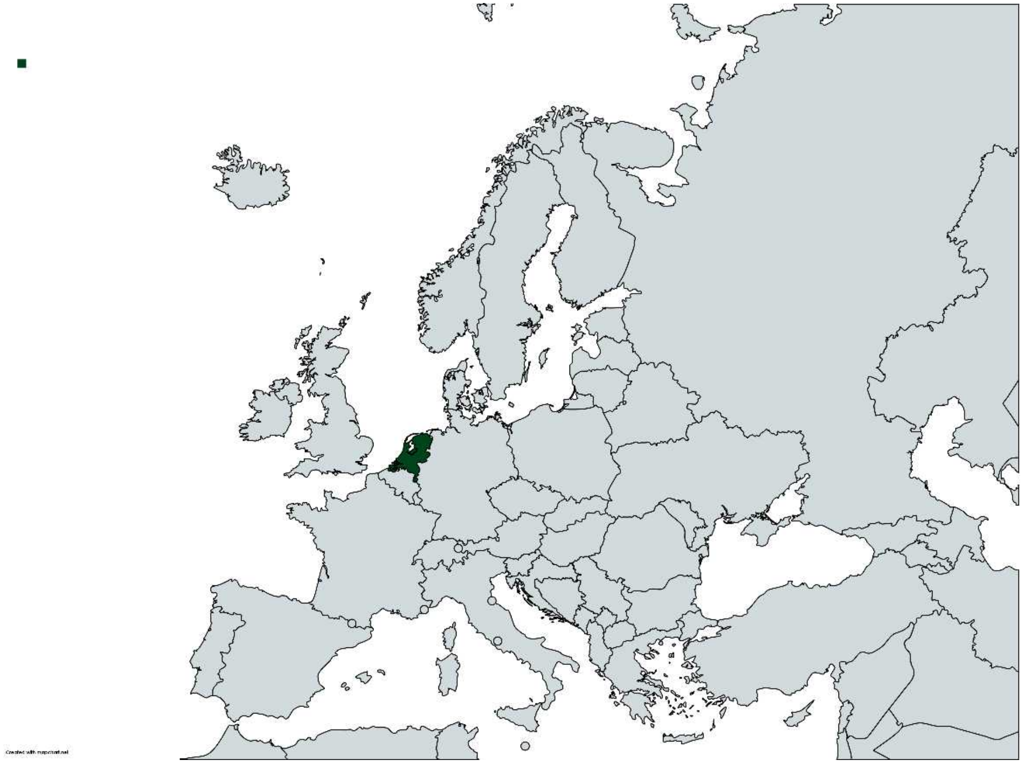

| Country | Netherlands |
|---|---|

Country-specific data

| Country characteristics | Method of payment | |
|---|---|---|
| | Data | Reimbursement of healthcare services is abateint's choice in-kind (the pateint pays the deductible to the health insurer and the health insurer directly reimburses the providers) or restitution (the patient pays the bill directly to the provider; the health insurer reimburses the patient) |
| | Time | 2013 |
| | Reference | http://imi-protect.eu/documents/DUinventory_2013_COUNTRIESyear4_Dec2013.pdf |
| | | |
| | **Medication adherence assessed and reported on the national level** | |
| | Data | No |
| | Time | 2022 |
| | Reference | NA |
| | | |
| | **Health care provider** | |
| | Data | Private. All residents have to purchase a basic benefit package which might be complemented by a voluntary supplementary health insurance. In addition to th estandard benefit package, all citizens are covered by the statutory Exceptional Medical Expenses Act scheme for a wide range of chronic and mental health illnesses, |
| | Time | 2013 |
| | Reference | http://imi-protect.eu/documents/DUinventory_2013_COUNTRIESyear4_Dec2013.pdf |
| | | |
| | **Model of healthcare system financing** | |
| | Data | Government funds (14%) and percentage compulsory contributions of taxable income (66%). Out-of-pocket payments 10% and 4%. Private voluntary health insurance. It is a free market controlled by the government. Of the 4 largest health insurers, only 1 has become a for-profit health insurance. |
| | Time | 2013 |
| | Reference | http://imi-protect.eu/documents/DUinventory_2013_COUNTRIESyear4_Dec2013.pdf |
| | | |
| | **Proportion of population aged 65 years and over** | |
| | Data - % of persons | 19.8 |
| | Time | 2021 |
| | Reference | https://ec.europa.eu/eurostat/databrowser/view/TPS00028/default/table?lang=en&category=demo.demo_ind / |
| | | |
| | **Country population (projection)** | |

| | | |
|---|---|---|
| | Data - N of persons | 17404793 |
| | Time | 2020 |
| | Reference | https://ec.europa.eu/eurostat/databrowser/view/CENS_HNMGA/default/table?lang=en&category=cens.cens_hn.cens_hnstr |

| | | |
|---|---|---|
| Social/economic factors | **Patient co-payment** | |
| | Data | If the price of a medicine is above the level of the reference price, the patient pays the difference between the reimbursement price and the pharamcy reatil price. Since 2009, benzodiazepines are not reimbursed anymore. |
| | Time | 2013 |
| | Reference | http://imi-protect.eu/documents/DUinventory_2013_COUNTRIESyear4_Dec2013.pdf |
| | | |
| | **Percentage of prescriptions dispensed at no cost to patients** | |
| | Data - % of prescriptions | NA |
| | Time | NA |
| | Reference | NA |
| | | |
| | **Population coverage** | |
| | Data | 0.97 |
| | Time | 2013 |
| | Reference | http://imi-protect.eu/documents/DUinventory_2013_COUNTRIESyear4_Dec2013.pdf |
| | | |
| | **Availability of doctors' services for citizens at no payment** | |
| | Data | NA |
| | Time | NA |
| | Reference | NA |

| | | |
|---|---|---|
| Therapy-related factors | **Average number of medicines per patient** | |
| | Data - N of medicines per patient | NA |
| | Time | NA |
| | Reference | NA |
| | | |
| | **Proportion of 75 years and over who are taking more than 5 medications concurrently** | |
| | Data - % of persons | 50.7 |
| | Time | 2020 |
| | Reference | https://stats.oecd.org/Index.aspx?ThemeTreeId=34 |

| | | |
|---|---|---|
| | | |
| | **Percentage of self-reported use of prescribed medicines** | |
| | Data - % of persons | 45.6 |
| | Time | 2019 |
| | Reference | https://ec.europa.eu/eurostat/databrowser/view/HLTH_EHIS_MD1E__custom_3764895/default/table?lang=en / |

| | | |
|---|---|---|
| Patient-related factors | **Percentage of persons reporting a chronic disease** | |
| | Data - Asthma, % of persons | 6.4 |
| | Data - Chronic lower respiratory diseases, % of persons | 5 |
| | Data - High blood pressure, % of persons | 16.1 |
| | Data - Diabetes, % of persons | 5.8 |
| | Data - Chronic depression, % of persons | 8.3 |
| | Time | 2019 |
| | Reference | https://ec.europa.eu/eurostat/databrowser/view/HLTH_EHIS_CD1E/default/table?lang=en&category=hlth.hlth_state.hlth_srcm / |
| | | |
| | **Percentage of self-perceived health - very good (16 years and over)** | |
| | Data - % of persons | 17.7 |
| | Time | 2021 |
| | Reference | https://ec.europa.eu/eurostat/databrowser/view/HLTH_SILC_02/default/table?lang=en&category=hlth.hlth_state.hlth_sph/ |
| | | |
| | **Percenatge of persons with current depressive symptoms** | |
| | Data - % of persons | 8.3 |
| | Time | 2019 |
| | Reference | https://ec.europa.eu/eurostat/databrowser/view/HLTH_EHIS_MH1E/default/table?lang=en&category=hlth.hlth_state.hlth_sph / |

| | | |
|---|---|---|
| Condition-related factor | **General health literacy** | |
| | Data - Inadequate health literacy, % of persons | 1.8 |
| | Data - Problematic health literacy, % of persons | 26.9 |
| | Data - Sufficient health literacy, % of persons | 46.3 |
| | Data - Excellent health literacy, % of persons | 25.1 |
| | Time | 2011 |
| | Reference | https://academic.oup.com/eurpub/article/25/6/1053/2467145 |

| | | |
|---|---|---|
| Healthcare system-relat | Percentage of patients receiving adherence interventions | |
| | Data - % of persons | NA |
| | Time | NA |
| | Reference | NA |
| | | |
| | Nationwide availability of e-prescription | |
| | Data | NA |
| | Time | NA |
| | Reference | NA |
| | | |
| | Waiting time for prescriptions / medical appointments | |
| | Data | NA |
| | Time | NA |
| | Reference | NA |
| | | |
| | Number of practising physicians per 100,000 inhabitants | |
| | Data - N of practisisng physicians per 100,000 inhabitants | 383.4 |
| | Time | 2020 |
| | Reference | https://ec.europa.eu/eurostat/databrowser/view/TPS00044/default/table?lang=en&category=hlth.hlth_care.hlth_res.hlth_staff%20%2F |
| | | |
| | Proportion of health care expenditure on pharmaceuticals | |
| | Data - % of health care expenditure | 6.877 |
| | Time | 2020 |
| | Reference | https://data.oecd.org/healthres/pharmaceutical-spending.htm |
| | | |
| | Number of practising pharmacists per 100,000 inhabitants | |
| | Data - N of practisisng pharmacists per 100,000 inhabitants | 21.64 |
| | Time | 2020 |
| | Reference | https://ec.europa.eu/eurostat/databrowser/view/HLTH_RS_PRS1__custom_4104351/default/table?lang=en |
| | | |
| | Total health care expenditure as percentage of GDP | |
| | Data - % of GDP | 11.14 |
| | Time | 2020 |

| | | |
|---|---|---|
| | Reference | https://ec.europa.eu/eurostat/databrowser/view/TPS00207/default/table?lang=en&category=hlth.hlth_care.hlth_sha11.hlth_sha11_sum |
| | | |
| | **Public pharmaceutical expenditure as percentage of total pharmaceutical expenditure** | |
| | Data - % of total pharmaceutical expenditure | 68.4 |
| | Time | 2019 |
| | Reference | https://gateway.euro.who.int/en/indicators/hfa_580-6790-public-pharmaceutical-expenditure-as-of-total-pharmaceutical-expenditure/visualizations/#id=19675&tab=table |
| | | |
| | **Self-reported consultations of a medical doctor*** | |
| | Data - No contact, % of population according to the number of consultations of a medical doctor in the past 4 weeks | 64.1 |
| | Data - 1 contact, % of population according to the number of consultations of a medical doctor in the past 4 weeks | 19.3 |
| | Data - 2 contacts, % of population according to the number of consultations of a medical doctor in the past 4 weeks | 8.6 |
| | Data - 3 or more contacts, % of population according to the number of consultations of a medical doctor in the past 4 weeks | 8.1 |
| | Time | 2019 |
| | Reference | https://ec.europa.eu/eurostat/databrowser/view/HLTH_EHIS_AM2U/default/table?lang=en&category=hlth.hlth_care.hlth_consult / |

*Medical doctors include generalist medical practitioners and specialist medical practitioners

NORTH MACEDONIA

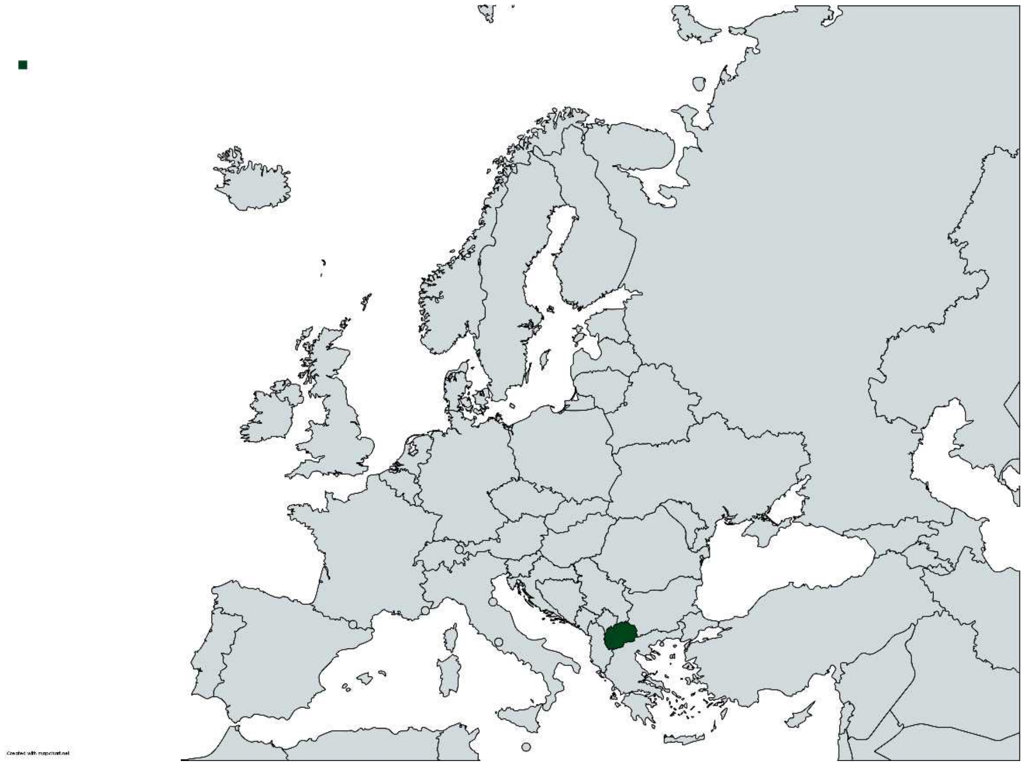

| Country | North Macedonia |
|---|---|

**Country-specific data**

| Country characteristics | Method of payment | |
|---|---|---|
| | Data | NA |
| | Time | NA |
| | Reference | NA |
| | | |
| | **Medication adherence assessed and reported on the national level** | |
| | Data | No |
| | Time | 2022 |
| | Reference | NA |
| | | |
| | **Health care provider** | |
| | Data | NA |
| | Time | NA |
| | Reference | NA |
| | | |
| | **Model of healthcare system financing** | |
| | Data | single-payer Health Insurance Fund (HIF) that acts as the main purchaser of publicly funded health services |
| | Time | 2022 |
| | Reference | https://www.google.com/url?sa=t&rct=j&q=&esrc=s&source=web&cd=&cad=rja&uact=8&ved=2ahUKEwjFkM761O_8AhWos4sKHRoVCI8QFnoECA8QAQ&url=https%3A%2F%2Fapps.who.int%2Firis%2Frest%2Fbitstreams%2F1463899%2Fretrieve&usg=AOvVaw0RYiFiWKl0YF0pGpEEDgFx |
| | | |
| | **Proportion of population aged 65 years and over** | |
| | Data - % of persons | 14.8 |
| | Time | 2021 |
| | Reference | https://ec.europa.eu/eurostat/databrowser/view/TPS00028/default/table?lang=en&category=demo.demo_ind / |
| | | |
| | **Country population (projection)** | |
| | Data - N of persons | 1836713 |
| | Time | 2021 |

| | | |
|---|---|---|
| | Reference | https://popis2021.stat.gov.mk/ |
| | | |
| Social/economic factors | **Patient co-payment** | |
| | Data | User charges (co-payments) up to a maximum of 20% of the price (50% for medical products). Overall, co-payments are capped at EUR 98 per service and there is an annual income-related cap on co-payments and exemptions for some people in vulnerable situations. However, these protection mechanisms do not apply to co-payments for outpatient medicines and medical products and there are no exemptions from co-payments for outpatient medicines and medical products for low-income households. |
| | Time | 2022 |
| | Reference | https://www.google.com/url?sa=t&rct=j&q=&esrc=s&source=web&cd=&cad=rja&uact=8&ved=2ahUKEwjFkM761O_8AhWos4sKHRoVCI8QFnoECA8QAQ&url=https%3A%2F%2Fapps.who.int%2Firis%2Frest%2Fbitstreams%2F1463899%2Fretrieve&usg=AOvVaw0RYiFiWKl0YF0pGpEEDgFx |
| | | |
| | **Percentage of prescriptions dispensed at no cost to patients** | |
| | Data - % of prescriptions | NA |
| | Time | NA |
| | Reference | NA |
| | | |
| | **Population coverage** | |
| | Data | North Macedonia's health system provides a relatively comprehensive basic benefit package, with about 90% of the population being covered under the social health insurance scheme. Most primary care services are free of charge but certain health services, in particular outpatient specialist visits, prescribed outpatient medicines and inpatient care, require user charges. |
| | Time | 2021 |
| | Reference | Ministry of Health (2021). Health Strategy 2021– 2030 Republic of North Macedonia. Skopje, Ministry of Health. |
| | | |
| | **Availability of doctors' services for citizens at no payment** | |
| | Data | Most primary care services are free of charge but certain health services, in particular outpatient specialist visits, prescribed outpatient medicines and inpatient care, require user charges. |
| | Time | 2021 |
| | Reference | Ministry of Health (2021). Health Strategy 2021– 2030 Republic of North Macedonia. Skopje, Ministry of Health. |
| | | |
| Therapy-related factors | **Average number of medicines per patient** | |
| | Data - N of medicines per patient | NA |
| | Time | NA |
| | Reference | NA |
| | | |
| | **Proportion of 75 years and over who are taking more than 5 medications concurrently** | |

| | | |
|---|---|---|
| | Data - % of persons | NA |
| | Time | NA |
| | Reference | NA |
| | | |
| | **Percentage of self-reported use of prescribed medicines** | |
| | Data - % of persons | NA |
| | Time | NA |
| | Reference | NA |

| | | |
|---|---|---|
| **Patient-related factors** | **Percentage of persons reporting a chronic disease** | |
| | Data - Asthma, % of persons | NA |
| | Data - Chronic lower respiratory diseases, % of persons | NA |
| | Data - High blood pressure, % of persons | NA |
| | Data - Diabetes, % of persons | NA |
| | Data - Chronic depression, % of persons | NA |
| | Time | NA |
| | Reference | NA |
| | | |
| | **Percentage of self-perceived health - very good (16 years and over)** | |
| | Data - % of persons | 29.6 |
| | Time | 2020 |
| | Reference | https://ec.europa.eu/eurostat/databrowser/view/HLTH_SILC_02/default/table?lang=en&category=hlth.hlth_state.hlth_sph/ |
| | | |
| | **Percenatge of persons with current depressive symptoms** | |
| | Data - % of persons | NA |
| | Time | NA |
| | Reference | NA |

| | | |
|---|---|---|
| **Condition-related factor** | **General health literacy** | |
| | Data - Inadequate health literacy, % of persons | NA |
| | Data - Problematic health literacy, % of persons | NA |
| | Data - Sufficient health literacy, % of persons | NA |
| | Data - Excellent health literacy, % of persons | NA |

| | | |
|---|---|---|
| | Time | NA |
| | Reference | NA |

| | | |
|---|---|---|
| Healthcare system-relat | **Percentage of patients receiving adherence interventions** | |
| | Data - % of persons | NA |
| | Time | NA |
| | Reference | NA |
| | | |
| | **Nationwide availability of e-prescription** | |
| | Data | Yes (partial) |
| | Time | 2022 |
| | Reference | https://www.google.com/url?sa=t&rct=j&q=&esrc=s&source=web&cd=&cad=rja&uact=8&ved=2ahUKEwjFkM761O_8AhWos4sKHRoVCl8QFnoECA8QAQ&url=https%3A%2F%2Fapps.who.int%2Firis%2Frest%2Fbitstreams%2F1463899%2Fretrieve&usg=AOvVaw0RYiFiWKl0YF0pGpEEDgFx |
| | | |
| | **Waiting time for prescriptions / medical appointments** | |
| | Data | NA |
| | Time | NA |
| | Reference | NA |
| | | |
| | **Number of practising physicians per 100,000 inhabitants** | |
| | Data - N of practisisng physicians per 100,000 inhabitants | 312 |
| | Time | 2019 |
| | Reference | https://www.google.com/url?sa=t&rct=j&q=&esrc=s&source=web&cd=&cad=rja&uact=8&ved=2ahUKEwjFkM761O_8AhWos4sKHRoVCl8QFnoECA8QAQ&url=https%3A%2F%2Fapps.who.int%2Firis%2Frest%2Fbitstreams%2F1463899%2Fretrieve&usg=AOvVaw0RYiFiWKl0YF0pGpEEDgFx |
| | | |
| | **Proportion of health care expenditure on pharmaceuticals** | |
| | Data - % of health care expenditure | NA |
| | Time | NA |
| | Reference | NA |
| | | |
| | **Number of practising pharmacists per 100,000 inhabitants** | |
| | Data - N of practisisng pharmacists per 100,000 inhabitants | 51 |
| | Time | 2019 |

| | |
|---|---|
| Reference | http://zdravstvo.gov.mk/wp-content/uploads/2021/12/19.11.-SZ-posledna-Konechna-Natsrt-Strategija-MKD.pdf |
| | |
| **Total health care expenditure as percentage of GDP** | |
| Data - % of GDP | 4.3 |
| Time | 2019 |
| Reference | https://www.google.com/url?sa=t&rct=j&q=&esrc=s&source=web&cd=&cad=rja&uact=8&ved=2ahUKEwjFkM761O_8AhWos4sKHRoVCI8QFnoECA8QAQ&url=https%3A%2F%2Fapps.who.int%2Firis%2Frest%2Fbitstreams%2F1463899%2Fretrieve&usg=AOvVaw0RYiFiWKl0YF0pGpEEDgFx |
| | |
| **Public pharmaceutical expenditure as percentage of total pharmaceutical expenditure** | |
| Data - % of total pharmaceutical expenditure | NA |
| Time | NA |
| Reference | NA |
| | |
| **Self-reported consultations of a medical doctor*** | |
| Data - No contact, % of population according to the number of consultations of a medical doctor in the past 4 weeks | NA |
| Data - 1 contact, % of population according to the number of consultations of a medical doctor in the past 4 weeks | NA |
| Data - 2 contacts, % of population according to the number of consultations of a medical doctor in the past 4 weeks | NA |
| Data - 3 or more contacts, % of population according to the number of consultations of a medical doctor in the past 4 weeks | NA |
| Time | NA |
| Reference | NA |

*Medical doctors include generalist medical practitioners and specialist medical practitioners

NORWAY

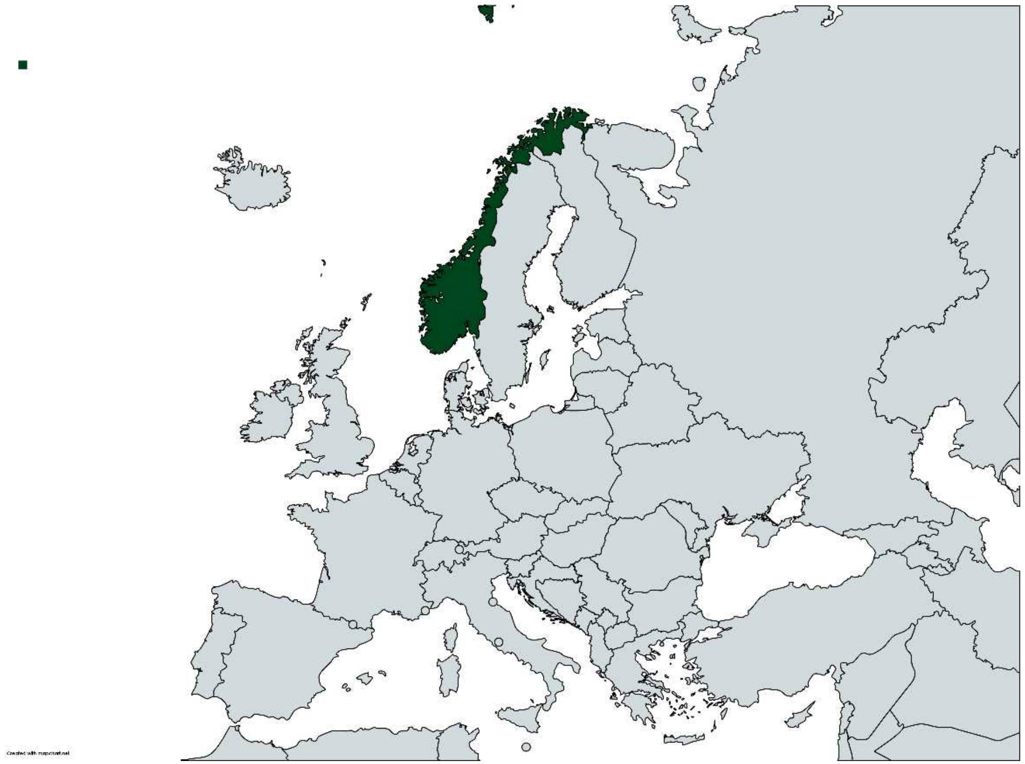

| Country | Norway |
|---|---|

**Country-specific data**

| Country characteristics | Method of payment | |
|---|---|---|
| | Data | The National Insurance Scheme (NIS) pays fully or partially the costs of drugs. |
| | Time | 2013 |
| | Reference | http://imi-protect.eu/documents/DUinventory_2013_COUNTRIESyear4_Dec2013.pdf |
| | | |
| | Medication adherence assessed and reported on the national level | |
| | Data | No |
| | Time | 2022 |
| | Reference | NA |
| | | |
| | Health care provider | |
| | Data | Public. Universal access to health care. |
| | Time | 2013 |
| | Reference | http://imi-protect.eu/documents/DUinventory_2013_COUNTRIESyear4_Dec2013.pdf |
| | | |
| | Model of healthcare system financing | |
| | Data | Predominantly tax based. Patient copayment of treatment by a general practitioner or for specialist treatment as outpatient, visit psychologist/psychiatrist, prescription of certain drugs and travel expenses. Private health care: Voluntary Health Insurance: barely plays any role.<br>A small number of private health care centres are opening up in urban areas, services only available to members. |
| | Time | 2013 |
| | Reference | http://imi-protect.eu/documents/DUinventory_2013_COUNTRIESyear4_Dec2013.pdf |
| | | |
| | Proportion of population aged 65 years and over | |
| | Data - % of persons | 17.9 |
| | Time | 2021 |
| | Reference | https://ec.europa.eu/eurostat/databrowser/view/TPS00028/default/table?lang=en&category=demo.demo_ind / |
| | | |
| | Country population (projection) | |

| | | |
|---|---|---|
| | Data - N of persons | 5475240 |
| | Time | 2023 |
| | Reference | Statistisk sentralbyrå (ssb.no) |

| | | |
|---|---|---|
| Social/economic factors | **Patient co-payment** | |
| | Data | Patient copayment is always 38% of the medicines price, up to a ceiling above which the NIS reimburses 90% of further expenditures. This ceiling includes all healthcare out-of-pocket expenditure. If patient refuses generic substitution, there is additional copayment. |
| | Time | 2013 |
| | Reference | http://imi-protect.eu/documents/DUinventory_2013_COUNTRIESyear4_Dec2013.pdf |
| | | |
| | **Percentage of prescriptions dispensed at no cost to patients** | |
| | Data - % of prescriptions | NA |
| | Time | NA |
| | Reference | NA |
| | | |
| | **Population coverage** | |
| | Data | All inhabitants covered by the National Insurance System. |
| | Time | 2013 |
| | Reference | http://imi-protect.eu/documents/DUinventory_2013_COUNTRIESyear4_Dec2013.pdf |
| | | |
| | **Availability of doctors' services for citizens at no payment** | |
| | Data | NA |
| | Time | NA |
| | Reference | NA |

| | | |
|---|---|---|
| Therapy-related factors | **Average number of medicines per patient** | |
| | Data - N of medicines per patient | NA |
| | Time | NA |
| | Reference | NA |
| | | |
| | **Proportion of 75 years and over who are taking more than 5 medications concurrently** | |
| | Data - % of persons | NA |
| | Time | NA |

| | | |
|---|---|---|
| | Reference | NA |
| | | |
| | **Percentage of self-reported use of prescribed medicines** | |
| | Data - % of persons | 71% of the population received at least one prescription drug in 2021 (78% of women, 65% of men) |
| | Time | 2021 |
| | Reference | https://www.fhi.no/contentassets/1b4b603c4ecf410588d584d5062cc9b8/legemiddelforbruket-i-norge-20172021.pdf |

| | | |
|---|---|---|
| Patient-related factors | **Percentage of persons reporting a chronic disease** | |
| | Data - Asthma, % of persons | 7.9 |
| | Data - Chronic lower respiratory diseases, % of persons | 2.5 |
| | Data - High blood pressure, % of persons | 15.1 |
| | Data - Diabetes, % of persons | 4.5 |
| | Data - Chronic depression, % of persons | 8.4 |
| | Time | 2019 |
| | Reference | https://ec.europa.eu/eurostat/databrowser/view/HLTH_EHIS_CD1E/default/table?lang=en&category=hlth.hlth_state.hlth_srcm / |
| | | |
| | **Percentage of self-perceived health - very good (16 years and over)** | |
| | Data - % of persons | 26.6 |
| | Time | 2020 |
| | Reference | https://ec.europa.eu/eurostat/databrowser/view/HLTH_SILC_02/default/table?lang=en&category=hlth.hlth_state.hlth_sph/ |
| | | |
| | **Percenatge of persons with current depressive symptoms** | |
| | Data - % of persons | 6.3 |
| | Time | 2019 |
| | Reference | https://ec.europa.eu/eurostat/databrowser/view/HLTH_EHIS_MH1E/default/table?lang=en&category=hlth.hlth_state.hlth_sph / |

| | | |
|---|---|---|
| Condition-related factor | **General health literacy** | |
| | Data - Inadequate health literacy, % of persons | 8 |
| | Data - Problematic health literacy, % of persons | 38 |
| | Data - Sufficient health literacy, % of persons | 35 |
| | Data - Excellent health literacy, % of persons | 20 |
| | Time | 2021 |
| | Reference | https://m-pohl.net/Int_Report_methdology_results_recommendations |

| | | |
|---|---|---|
| Healthcare system-relat | **Percentage of patients receiving adherence interventions** | |
| | Data - % of persons | NA |
| | Time | NA |
| | Reference | NA |
| | | |
| | **Nationwide availability of e-prescription** | |
| | Data | Yes |
| | Time | 2023 |
| | Reference | https://www.helsenorge.no/en/medicines/e-resept-og-mine-resepter/what-is-e-prescription/ |
| | | |
| | **Waiting time for prescriptions / medical appointments** | |
| | Data | Prescriptions - 0-1 day. Medical appointments: Acute - the same day. Chronic - 1 -5 days |
| | Time | 2023 |
| | Reference | https://lovdata.no/dokument/SF/forskrift/2012-08-29-842 |
| | | |
| | **Number of practising physicians per 100,000 inhabitants** | |
| | Data - N of practisisng physicians per 100,000 inhabitants | 517.96 |
| | Time | 2021 |
| | Reference | https://ec.europa.eu/eurostat/databrowser/view/TPS00044/default/table?lang=en&category=hlth.hlth_care.hlth_res.hlth_staff%20%2F |
| | | |
| | **Proportion of health care expenditure on pharmaceuticals** | |
| | Data - % of health care expenditure | 7.194 |
| | Time | 2020 |
| | Reference | https://data.oecd.org/healthres/pharmaceutical-spending.htm |
| | | |
| | **Number of practising pharmacists per 100,000 inhabitants** | |
| | Data - N of practisisng pharmacists per 100,000 inhabitants | 91.61 |
| | Time | 2021 |
| | Reference | https://ec.europa.eu/eurostat/databrowser/view/HLTH_RS_PRS1__custom_4104351/default/table?lang=en |
| | | |
| | **Total health care expenditure as percentage of GDP** | |
| | Data - % of GDP | 10.54 |

| | |
|---|---|
| Time | 2019 |
| Reference | https://ec.europa.eu/eurostat/databrowser/view/TPS00207/default/table?lang=en&category=hlth.hlth_care.hlth_sha11.hlth_sha11_sum |
| | |
| **Public pharmaceutical expenditure as percentage of total pharmaceutical expenditure** | |
| Data - % of total pharmaceutical expenditure | 54.3 |
| Time | 2013 |
| Reference | https://gateway.euro.who.int/en/indicators/hfa_580-6790-public-pharmaceutical-expenditure-as-of-total-pharmaceutical-expenditure/visualizations/#id=19675&tab=table |
| | |
| **Self-reported consultations of a medical doctor*** | |
| Data - No contact, % of population according to the number of consultations of a medical doctor in the past 4 weeks | 72.3 |
| Data - 1 contact, % of population according to the number of consultations of a medical doctor in the past 4 weeks | 19.5 |
| Data - 2 contacts, % of population according to the number of consultations of a medical doctor in the past 4 weeks | 5.3 |
| Data - 3 or more contacts, % of population according to the number of consultations of a medical doctor in the past 4 weeks | 2.9 |
| Time | 2019 |
| Reference | https://ec.europa.eu/eurostat/databrowser/view/HLTH_EHIS_AM2U/default/table?lang=en&category=hlth.hlth_care.hlth_consult / |

*Medical doctors include generalist medical practitioners and specialist medical practitioners

POLAND

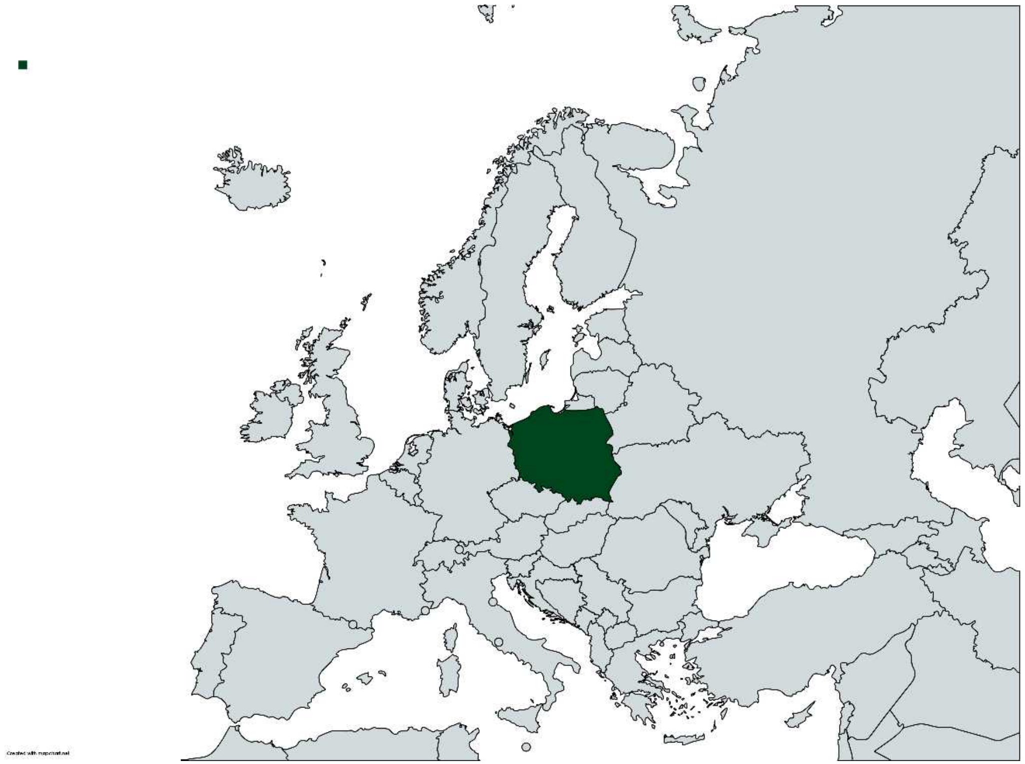

| Country | Poland |
|---|---|

Country-specific data

| Country characteristics | Method of payment | |
|---|---|---|
| | Data | The National Health Insurance Fund (NFZ) partially pays the medicines direct to the pharmacist. |
| | Time | 2013 |
| | Reference | http://imi-protect.eu/documents/DUinventory_2013_COUNTRIESyear4_Dec2013.pdf |
| | | |
| | **Medication adherence assessed and reported on the national level** | |
| | Data | No |
| | Time | 2022 |
| | Reference | NA |
| | | |
| | **Health care provider** | |
| | Data | Public social insurance system |
| | Time | 2013 |
| | Reference | http://imi-protect.eu/documents/DUinventory_2013_COUNTRIESyear4_Dec2013.pdf |
| | | |
| | **Model of healthcare system financing** | |
| | Data | Model of financing is universal public healthcare insurance. Compulsory health insurance contributions at a percentage rate of the employee income. Money is collected from those professionally active, and goes directly to the National Health Fund (and NOT to the state budget). State budget pays for several special health programs only. In public health system, patients do not pay any money. However, they pay out-of-pocket payments, ranging from the lump sum of PLN 3.20, to 100% of the drug costs, for many drugs. Market share of private health insurance is small: currently (as for January, 2022) 3.7 million Polish citizens are insured on private, and they are spending on this at app. 1 billion PLN a year. For comparison, total private spending on healthcare were estimated in 2020 at 44 billion PLN, and the total budget of National Health Fund for 2023 is 144 billion PLN |
| | Time | 2022 |
| | Reference | [https://www.prawo.pl/zdrowie/rynek-prywatnych-ubezpieczen-zdrowotnych,513006.html, https://www.nfz.gov.pl/bip/finanse-nfz/ |
| | | |
| | **Proportion of population aged 65 years and over** | |
| | Data - % of persons | 18.7 |
| | Time | 2021 |

| | | |
|---|---|---|
| | Reference | https://ec.europa.eu/eurostat/databrowser/view/TPS00028/default/table?lang=en&category=demo.demo_ind / |
| | | |
| | **Country population (projection)** | |
| | Data - N of persons | 37908000 |
| | Time | 2021 |
| | Reference | https://stat.gov.pl/obszary-tematyczne/ludnosc/ludnosc/ludnosc-stan-i-struktura-ludnosci-oraz-ruch-naturalny-w-przekroju-terytorialnym-w-2022-r-stan-w-dniu-30-czerwca-2022,6,33.html |

| | | |
|---|---|---|
| Social/economic factors | **Patient co-payment** | |
| | Data | In those who are beneficiary of universal national health service, patient co-payment may be 0% for several basic drugs which are free of charge (e.g. antipsychotics, anticancer drugs, etc); a flat fee of PLN 3.20 per package prescribed for drugs on the basic drug list, whereas in the other cases 30% or 50% co-payment depending on the class of drugs, independent for how long the treatment is prescribed, nor how wealthy is a person. Less effective drugs (according to public health technology assessment authority) are subject of 100% patient payment. Special reduction of drug prices down to 0% of co-payment for war veterans, and those aged 75+ (covers selected drugs only). |
| | Time | 2023 |
| | Reference | Expert opinion |
| | | |
| | **Percentage of prescriptions dispensed at no cost to patients** | |
| | Data - % of prescriptions | NA |
| | Time | NA |
| | Reference | NA |
| | | |
| | **Population coverage** | |
| | Data | 0.98 |
| | Time | 2013 |
| | Reference | http://imi-protect.eu/documents/DUinventory_2013_COUNTRIESyear4_Dec2013.pdf |
| | | |
| | **Availability of doctors' services for citizens at no payment** | |
| | Data | Services provided within universal national health service are free of charge, this inclueds doctor's services also. |
| | Time | 2023 |
| | Reference | Expert opinion |

| | | |
|---|---|---|
| Therapy-related factors | **Average number of medicines per patient** | |

| | | |
|---|---|---|
| | Data - N of medicines per patient | According to the NHF data, in 2018 a total of 23.3 million Polish citizens filled in their prescriptions for reimbursed drugs, and 19.1 million were dispensed medicines with ATC codes included in this study. The given number, on average, included 3.7 (+/−3.2) active substances, 4.8 (+/− 4.5) different drug EAN codes and 19.7 (+/−24.2) drug packages per patient per year |
| | Time | 2018 |
| | Reference | Kardas P, Urbański F, Lichwierowicz A, Chudzyńska E, Kardas G, Czech M. Prevalence and Age Structure of Polypharmacy in Poland: Results of the Analysis of the National Real-World Database of 38 Million Citizens. Front Pharmacol. 2021 Apr 15;12:655364 |
| | | |
| | **Proportion of 75 years and over who are taking more than 5 medications concurrently** | |
| | Data - % of persons | Thosa aged 65-79: 37.9-38.8%; Those aged 80+: 55.0-56.0% |
| | Time | 2018, 2019 |
| | Reference | Kardas P, Urbański F, Lichwierowicz A, Chudzyńska E, Kardas G, Czech M. Prevalence and Age Structure of Polypharmacy in Poland: Results of the Analysis of the National Real-World Database of 38 Million Citizens. Front Pharmacol. 2021 Apr 15;12:655364 |
| | | |
| | **Percentage of self-reported use of prescribed medicines** | |
| | Data - % of persons | 49.2 |
| | Time | 2019 |
| | Reference | https://ec.europa.eu/eurostat/databrowser/view/HLTH_EHIS_MD1E__custom_3764895/default/table?lang=en / |

| | | |
|---|---|---|
| Patient-related factors | **Percentage of persons reporting a chronic disease** | |
| | Data - Asthma, % of persons | 4.1 |
| | Data - Chronic lower respiratory diseases, % of persons | 2.7 |
| | Data - High blood pressure, % of persons | 26.5 |
| | Data - Diabetes, % of persons | 8.1 |
| | Data - Chronic depression, % of persons | 4.2 |
| | Time | 2019 |
| | Reference | https://ec.europa.eu/eurostat/databrowser/view/HLTH_EHIS_CD1E/default/table?lang=en&category=hlth.hlth_state.hlth_srcm / |
| | | |
| | **Percentage of self-perceived health - very good (16 years and over)** | |
| | Data - % of persons | 15.7 |
| | Time | 2021 |
| | Reference | https://ec.europa.eu/eurostat/databrowser/view/HLTH_SILC_02/default/table?lang=en&category=hlth.hlth_state.hlth_sph/ |
| | | |
| | **Percenatge of persons with current depressive symptoms** | |
| | Data - % of persons | 5.2 |

| | | |
|---|---|---|
| | Time | 2019 |
| | Reference | https://ec.europa.eu/eurostat/databrowser/view/HLTH_EHIS_MH1E/default/table?lang=en&category=hlth.hlth_state.hlth_sph / |

| | | |
|---|---|---|
| **Condition-related factor** | **General health literacy** | |
| | Data - Inadequate health literacy, % of persons | 10.2 |
| | Data - Problematic health literacy, % of persons | 34.4 |
| | Data - Sufficient health literacy, % of persons | 35.9 |
| | Data - Excellent health literacy, % of persons | 19.5 |
| | Time | 2011 |
| | Reference | https://academic.oup.com/eurpub/article/25/6/1053/2467145 |

| | | |
|---|---|---|
| **Healthcare system-relat** | **Percentage of patients receiving adherence interventions** | |
| | Data - % of persons | NA |
| | Time | NA |
| | Reference | NA |
| | | |
| | **Nationwide availability of e-prescription** | |
| | Data | Yes |
| | Time | 2023 |
| | Reference | https://www.cez.gov.pl/pl/node/156 |
| | | |
| | **Waiting time for prescriptions / medical appointments** | |
| | Data | Primary care: same day in acute cases, by appointment in the other cases - usually, within a week. Specialist consultaions: from days to 2 months, usually. |
| | Time | 2023 |
| | Reference | https://terminyleczenia.nfz.gov.pl/# |
| | | |
| | **Number of practising physicians per 100,000 inhabitants** | |
| | Data - N of practisisng physicians per 100,000 inhabitants | 237.75 |
| | Time | 2017 |
| | Reference | https://ec.europa.eu/eurostat/databrowser/view/TPS00044/default/table?lang=en&category=hlth.hlth_care.hlth_res.hlth_staff%20%2F |
| | | |
| | **Proportion of health care expenditure on pharmaceuticals** | |

| | | |
|---|---|---|
| | Data - % of health care expenditure | 11 |
| | Time | 2023 |
| | Reference | https://www.nfz.gov.pl/bip/finanse-nfz/ |
| | | |
| | **Number of practising pharmacists per 100,000 inhabitants** | |
| | Data - N of practisisng pharmacists per 100,000 inhabitants | 77.24 |
| | Time | 2017 |
| | Reference | https://ec.europa.eu/eurostat/databrowser/view/HLTH_RS_PRS1__custom_4104351/default/table?lang=en |
| | | |
| | **Total health care expenditure as percentage of GDP** | |
| | Data - % of GDP | 6.22% (public), 6.6% (public + private) |
| | Time | 2021 |
| | Reference | https://stat.gov.pl/obszary-tematyczne/zdrowie/zdrowie/wydatki-na-ochrone-zdrowia-w-latach-2019-2021,27,2.html |
| | | |
| | **Public pharmaceutical expenditure as percentage of total pharmaceutical expenditure** | |
| | Data - % of total pharmaceutical expenditure | 39.4 |
| | Time | 2011 |
| | Reference | https://gateway.euro.who.int/en/indicators/hfa_580-6790-public-pharmaceutical-expenditure-as-of-total-pharmaceutical-expenditure/visualizations/#id=19675&tab=table |
| | | |
| | **Self-reported consultations of a medical doctor*** | |
| | Data - No contact, % of population according to the number of consultations of a medical doctor in the past 4 weeks | 38 |
| | Data - 1 contact, % of population according to the number of consultations of a medical doctor in the past 4 weeks | 17 |
| | Data - 2 contacts, % of population according to the number of consultations of a medical doctor in the past 4 weeks | 26.7 |
| | Data - 3 or more contacts, % of population according to the number of consultations of a medical doctor in the past 4 weeks | 18.3 |
| | Time | 2019 |
| | Reference | https://ec.europa.eu/eurostat/databrowser/view/HLTH_EHIS_AM2U/default/table?lang=en&category=hlth.hlth_care.hlth_consult / |

*Medical doctors include generalist medical practitioners and specialist medical practitioners

PORTUGAL

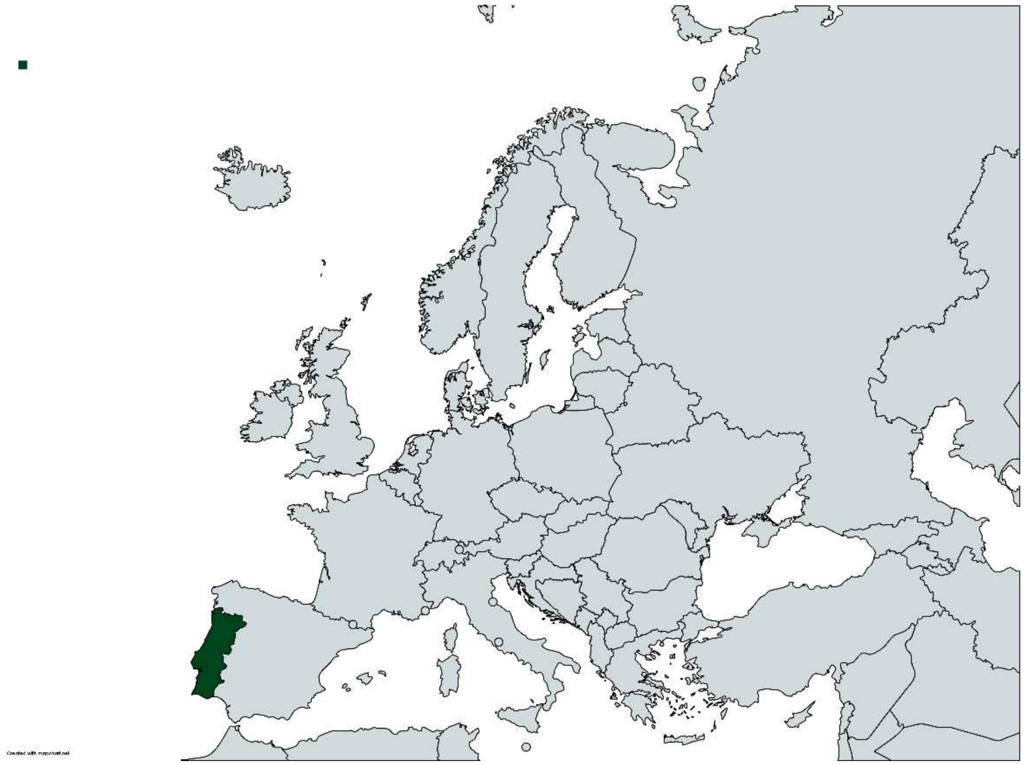

| Country | Portugal |
|---|---|

Country-specific data

| Country characteristics | | |
|---|---|---|
| | **Method of payment** | |
| | Data | The health system in Portugal is constituted by a publicly funded National Health Service (NHS - Bevedrigde type), and by a myriad of other sub-systems (mostly Bismark type supported by employers and workers contributions). People can also have a voluntary health insurance to facilitate access to private providers. |
| | Time | 2017 |
| | Reference | https://www.euro.who.int/__data/assets/pdf_file/0007/337471/HiT-Portugal.pdf |
| | | |
| | **Medication adherence assessed and reported on the national level** | |
| | Data | No |
| | Time | 2022 |
| | Reference | NA |
| | | |
| | **Health care provider** | |
| | Data | Three coexisting systems: universal national health system (SNS, Serviço Nacional Saúde). Special public and private insurance schemes known as health subsystems; and private voluntary health insurance (VHI). Universal access to health services. The third provider is the Social Sector composed by NGO's and Charities. It often contracts with the NHS to provide complementary services in convalescent and continuing care |
| | Time | 2021 |
| | Reference | https://www.pordata.pt/db/portugal/ambiente+de+consulta/tabela |
| | | |
| | **Model of healthcare system financing** | |
| | Data | The NHS is covered by general taxation. Health subsystems – which for some professions are compulsory -, are financed with employee/employer contributions. Out-of-pocket payments represent about 23.6% of the healthcare expenditure in Portugal. |
| | Time | 2021 |
| | Reference | https://www.pordata.pt/db/portugal/ambiente+de+consulta/tabela |
| | | |
| | **Proportion of population aged 65 years and over** | |
| | Data - % of persons | 23.4 |
| | Time | 2022 |
| | Reference | https://tabulador.ine.pt/indicador/?id=0011609 |
| | | |

| | | |
|---|---|---|
| | **Country population (projection)** | |
| | Data - N of persons | 10343066 |
| | Time | 2022 |
| | Reference | https://tabulador.ine.pt/indicador/?id=0011609 |
| Social/economic factors | **Patient co-payment** | |
| | Data | There are no flat fee charges for prescriptions in Portugal. General scheme: medicines categories (copayment rate): A (10%), B (31%); C (67%); D (95%). Specific scheme: pensioners can get an extra percentage reduction of 5% for A drugs and 15% for B, C, and D drugs. Compound drugs 50% copayment. |
| | Time | 2013 |
| | Reference | http://imi-protect.eu/documents/DUinventory_2013_COUNTRIESyear4_Dec2013.pdf |
| | | |
| | **Percentage of prescriptions dispensed at no cost to patients** | |
| | Data - % of prescriptions | NA |
| | Time | NA |
| | Reference | NA |
| | | |
| | **Population coverage** | |
| | Data | The SNS is universal. Private VHI covers about 26% of the population, and it mainly has a supplementary role. The majority of the population cannot choose between 3 health systems. There are 26% of people that can have double coverage, and some of these even triple coverage. These are a minority (only public service employees or other employees with a bismark type sub-system can access this triple coverage). |
| | Time | 2017 |
| | Reference | https://www.euro.who.int/__data/assets/pdf_file/0007/337471/HiT-Portugal.pdf |
| | | |
| | **Availability of doctors' services for citizens at no payment** | |
| | Data | Since Jan 2021 there are no charges for primary health care consultations. While user charges exist for consultations, emergency visits, home visits, diagnostic tests and therapeutic procedures, around 60% of the population is exempted from paying them. |
| | Time | 2017 |
| | Reference | https://www.euro.who.int/__data/assets/pdf_file/0007/337471/HiT-Portugal.pdf |
| | | |
| Therapy-related factors | **Average number of medicines per patient** | |
| | Data - N of medicines per patient | NA |
| | Time | NA |
| | Reference | NA |

| | | |
|---|---|---|
| | | |
| | **Proportion of 75 years and over who are taking more than 5 medications concurrently** | |
| | Data - % of persons | 73.9 |
| | Time | 2020 |
| | Reference | https://stats.oecd.org/Index.aspx?ThemeTreeId=38 |
| | | |
| | **Percentage of self-reported use of prescribed medicines** | |
| | Data - % of persons | 55.6 |
| | Time | 2019 |
| | Reference | https://ec.europa.eu/eurostat/databrowser/view/HLTH_EHIS_MD1E__custom_3764895/default/table?lang=en / |

| | | |
|---|---|---|
| Patient-related factors | **Percentage of persons reporting a chronic disease** | |
| | Data - Asthma, % of persons | 6.1 |
| | Data - Chronic lower respiratory diseases, % of persons | 5.5 |
| | Data - High blood pressure, % of persons | 26.6 |
| | Data - Diabetes, % of persons | 10 |
| | Data - Chronic depression, % of persons | 12.2 |
| | Time | 2019 |
| | Reference | https://ec.europa.eu/eurostat/databrowser/view/HLTH_EHIS_CD1E/default/table?lang=en&category=hlth.hlth_state.hlth_srcm / |
| | | |
| | **Percentage of self-perceived health - very good (16 years and over)** | |
| | Data - % of persons | 12.7 |
| | Time | 2021 |
| | Reference | https://ec.europa.eu/eurostat/databrowser/view/HLTH_SILC_02/default/table?lang=en&category=hlth.hlth_state.hlth_sph/ |
| | | |
| | **Percenatge of persons with current depressive symptoms** | |
| | Data - % of persons | 8.5 |
| | Time | 2019 |
| | Reference | https://ec.europa.eu/eurostat/databrowser/view/HLTH_EHIS_MH1E/default/table?lang=en&category=hlth.hlth_state.hlth_sph / |

| | | |
|---|---|---|
| Condition-related factor | **General health literacy** | |
| | Data - Inadequate health literacy, % of persons | 8 |
| | Data - Problematic health literacy, % of persons | 22 |

| | | |
|---|---|---|
| | Data - Sufficient health literacy, % of persons | 65 |
| | Data - Excellent health literacy, % of persons | 5 |
| | Time | 2021 |
| | Reference | https://m-pohl.net/Int_Report_methdology_results_recommendations |

| | | |
|---|---|---|
| Healthcare system-relat | **Percentage of patients receiving adherence interventions** | |
| | Data - % of persons | NA |
| | Time | NA |
| | Reference | NA |
| | | |
| | **Nationwide availability of e-prescription** | |
| | Data | Yes |
| | Time | 2022 |
| | Reference | https://www.infarmed.pt/documents/15786/1229727/Estat%C3%ADstica+do+Medicamento+2019/b2e448a8-dc71-c2e8-a93a-f0cbef7ad6eb?version=1.0 |
| | | |
| | **Waiting time for prescriptions / medical appointments** | |
| | Data | NA |
| | Time | NA |
| | Reference | NA |
| | | |
| | **Number of practising physicians per 100,000 inhabitants** | |
| | Data - N of practisisng physicians per 100,000 inhabitants | 532 |
| | Time | 2019 |
| | Reference | https://www.who.int/europe/publications/i/item/9789289058339 |
| | | |
| | **Proportion of health care expenditure on pharmaceuticals** | |
| | Data - % of health care expenditure | 15.647 |
| | Time | 2020 |
| | Reference | https://data.oecd.org/healthres/pharmaceutical-spending.htm |
| | | |
| | **Number of practising pharmacists per 100,000 inhabitants** | |
| | Data - N of practisisng pharmacists per 100,000 inhabitants | 95.32 |
| | Time | 2020 |

| | | |
|---|---|---|
| | Reference | https://ec.europa.eu/eurostat/databrowser/view/HLTH_RS_PRS1__custom_4104351/default/table?lang=en |
| | | |
| | **Total health care expenditure as percentage of GDP** | |
| | Data - % of GDP | 10.55 |
| | Time | 2020 |
| | Reference | https://ec.europa.eu/eurostat/databrowser/view/TPS00207/default/table?lang=en&category=hlth.hlth_care.hlth_sha11.hlth_sha11_sum |
| | | |
| | **Public pharmaceutical expenditure as percentage of total pharmaceutical expenditure** | |
| | Data - % of total pharmaceutical expenditure | 48.3 |
| | Time | 2019 |
| | Reference | https://www.infarmed.pt/documents/15786/1229727/Estat%C3%ADstica+do+Medicamento+2019/b2e448a8-dc71-c2e8-a93a-f0cbef7ad6eb?version=1.0 |
| | | |
| | **Self-reported consultations of a medical doctor*** | |
| | Data - No contact, % of population according to the number of consultations of a medical doctor in the past 4 weeks | 59.6 |
| | Data - 1 contact, % of population according to the number of consultations of a medical doctor in the past 4 weeks | 24.4 |
| | Data - 2 contacts, % of population according to the number of consultations of a medical doctor in the past 4 weeks | 9.6 |
| | Data - 3 or more contacts, % of population according to the number of consultations of a medical doctor in the past 4 weeks | 6.4 |
| | Time | 2019 |
| | Reference | https://ec.europa.eu/eurostat/databrowser/view/HLTH_EHIS_AM2U/default/table?lang=en&category=hlth.hlth_care.hlth_consult / |

*Medical doctors include generalist medical practitioners and specialist medical practitioners

ROMANIA

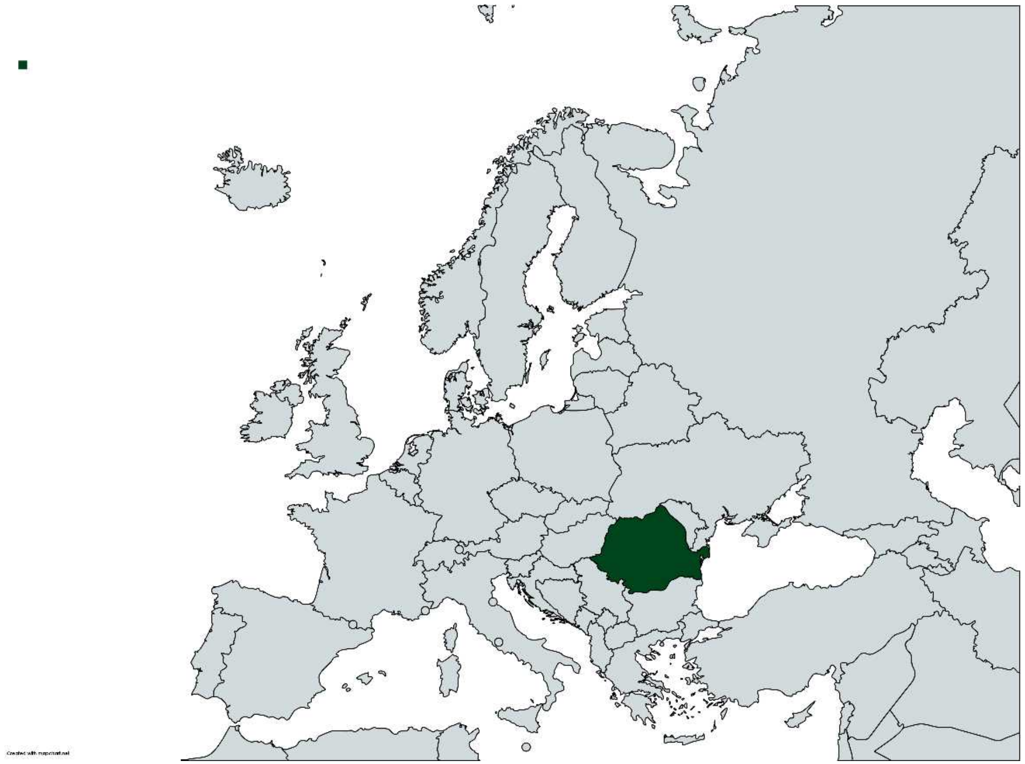

| Country | Romania |
|---|---|

Country-specific data

| Country characteristics | Method of payment | |
|---|---|---|
| | Data | National Agency for Medicines and Medical Devices (NAMMD), when performing the health technology assessment, decide if a medicine can be reimbursed unconditionally or conditionally in Romania. The price of the medicine, one of the elements analysed by the NAMMD, can be reimbursed at a level of 20%, 50%, 90% or 100%, depending on the reimbursement list where the medicine is included. The reimbursement prices are computed and approved by the National Health Insurance House (NHIH) for each medicine included in the national list of reimbursed medicines, depending on the reimbursement level applicable to the relevant product. The reimbursement price can be equal to or lower than the maximum price approved by the Ministry of Health. The medicines can be subject to certain clawback or payback taxes, which are calculated and due on a quarterly basis depending on the values borne by the Romanian public health insurance system. However, these taxes are only payable on medicines sold in Romania at the (maximum) price levels approved by the Ministry of Health for Romania. Certain medical devices are reimbursed in the national health insurance system, at a value established by the Ministry of Health and/or the NHIH. |
| | Time | 2018 |
| | Reference | https://uk.practicallaw.thomsonreuters.com/2-531-0685?transitionType=Default&contextData=(sc.Default)&firstPage=true#co_anchor_a754121 |
| | | |
| | Medication adherence assessed and reported on the national level | |
| | Data | No |
| | Time | 2022 |
| | Reference | Expert opinion |
| | | |
| | Health care provider | |
| | Data | Public and private healthcare providers operate in parallel. |
| | Time | 2018 |
| | Reference | https://uk.practicallaw.thomsonreuters.com/2-531-0685?transitionType=Default&contextData=(sc.Default)&firstPage=true#co_anchor_a754121 |
| | | |
| | Model of healthcare system financing | |
| | Data | The Romanian health system is mainly public financed (80.45%) through the following sources: Social Health Insurance (65%), State and Local Authorities Budget (15.45%), while the private sources (out of pocket and voluntary health insurance) add an additional 19.55% to the public funds. For the additional funding, out-of-pocket payments represent the main driver, and voluntary health insurance is marginal (0.1%). The largest expenditure of the public budget is for inpatient care (40%), followed by drugs (25%). |
| | Time | 2018 |

| | | |
|---|---|---|
| | Reference | Radu CP, Pana BC, Pele DT, Costea RV. Evolution of Public Health Expenditure Financed by the Romanian Social Health Insurance Scheme From 1999 to 2019. Front Public Health. 2021 Dec 1;9:795869. doi: 10.3389/fpubh.2021.795869. Erratum in: Front Public Health. 2022 Feb 15;10:857426. PMID: 34926399; PMCID: PMC8673551. |
| | | |
| | **Proportion of population aged 65 years and over** | |
| | Data - % of persons | 19.3 |
| | Time | 2021 |
| | Reference | https://ec.europa.eu/eurostat/databrowser/view/TPS00028/default/table?lang=en&category=demo.demo_ind / |
| | | |
| | **Country population (projection)** | |
| | Data - N of persons | 19281118 |
| | Time | 2020 |
| | Reference | https://ec.europa.eu/eurostat/databrowser/view/CENS_HNMGA/default/table?lang=en&category=cens.cens_hn.cens_hnstr |

| | | |
|---|---|---|
| Social/economic factors | **Patient co-payment** | |
| | Data | NHIH and the other public health insurance houses in its subordination cover the reimbursed value, by providing the reimbursement amounts to the pharmacies releasing the reimbursed medicinal products and/or medical devices, based on special supply agreements. The role of private payers in relation to the reimbursement of medicines and/or medical devices is not expressly regulated by the legislation currently in force in the healthcare field. |
| | Time | 2018 |
| | Reference | https://uk.practicallaw.thomsonreuters.com/2-531-0685?transitionType=Default&contextData=(sc.Default)&firstPage=true#co_anchor_a754121 |
| | | |
| | **Percentage of prescriptions dispensed at no cost to patients** | |
| | Data - % of prescriptions | NA |
| | Time | NA |
| | Reference | NA |
| | | |
| | **Population coverage** | |
| | Data | Patients insured in the national health insurance system can receive medicinal products and/or certain medical devices from pharmacies which have concluded reimbursement agreements with health insurance houses, either free of charge or by making a co-payment. |
| | Time | 2018 |
| | Reference | https://uk.practicallaw.thomsonreuters.com/2-531-0685?transitionType=Default&contextData=(sc.Default)&firstPage=true#co_anchor_a754121 |
| | | |
| | **Availability of doctors' services for citizens at no payment** | |

| | | |
|---|---|---|
| | Data | NA |
| | Time | NA |
| | Reference | NA |
| | | |
| Therapy-related factors | **Average number of medicines per patient** | |
| | Data - N of medicines per patient | NA |
| | Time | NA |
| | Reference | NA |
| | | |
| | **Proportion of 75 years and over who are taking more than 5 medications concurrently** | |
| | Data - % of persons | NA |
| | Time | NA |
| | Reference | NA |
| | | |
| | **Percentage of self-reported use of prescribed medicines** | |
| | Data - % of persons | 23.1 |
| | Time | 2019 |
| | Reference | https://ec.europa.eu/eurostat/databrowser/view/HLTH_EHIS_MD1E__custom_3764895/default/table?lang=en / |
| | | |
| Patient-related factors | **Percentage of persons reporting a chronic disease** | |
| | Data - Asthma, % of persons | 1.5 |
| | Data - Chronic lower respiratory diseases, % of persons | 1.2 |
| | Data - High blood pressure, % of persons | 15.7 |
| | Data - Diabetes, % of persons | 5 |
| | Data - Chronic depression, % of persons | 1 |
| | Time | 2019 |
| | Reference | https://ec.europa.eu/eurostat/databrowser/view/HLTH_EHIS_CD1E/default/table?lang=en&category=hlth.hlth_state.hlth_srcm / |
| | | |
| | **Percentage of self-perceived health - very good (16 years and over)** | |
| | Data - % of persons | 31.8 |
| | Time | 2021 |
| | Reference | https://ec.europa.eu/eurostat/databrowser/view/HLTH_SILC_02/default/table?lang=en&category=hlth.hlth_state.hlth_sph/ |
| | | |

| | | |
|---|---|---|
| | **Percenatge of persons with current depressive symptoms** | |
| | Data - % of persons | 4.3 |
| | Time | 2019 |
| | Reference | https://ec.europa.eu/eurostat/databrowser/view/HLTH_EHIS_MH1E/default/table?lang=en&category=hlth.hlth_state.hlth_sph / |

| | | |
|---|---|---|
| **Condition-related factors** | **General health literacy** | |
| | Data - Inadequate health literacy, % of persons | 7.5 |
| | Data - Problematic health literacy, % of persons | 33.2 |
| | Data - Sufficient health literacy, % of persons | 59.2 |
| | Data - Excellent health literacy, % of persons | NA |
| | Time | 2022 |
| | Reference | Coman MA, Forray AI, Van den Broucke S, Chereches RM. Measuring Health Literacy in Romania: Validation of the HLS-EU-Q16 Survey Questionnaire. Int J Public Health. 2022 Feb 4;67:1604272. doi: 10.3389/ijph.2022.1604272. PMID: 35185446; PMCID: PMC8855985. |

| | | |
|---|---|---|
| **Healthcare system-relat** | **Percentage of patients receiving adherence interventions** | |
| | Data - % of persons | NA |
| | Time | NA |
| | Reference | NA |
| | | |
| | **Nationwide availability of e-prescription** | |
| | Data | NA |
| | Time | NA |
| | Reference | NA |
| | | |
| | **Waiting time for prescriptions / medical appointments** | |
| | Data | NA |
| | Time | NA |
| | Reference | NA |
| | | |
| | **Number of practising physicians per 100,000 inhabitants** | |
| | Data - N of practisisng physicians per 100,000 inhabitants | 333 |
| | Time | 2020 |

| | |
|---|---|
| Reference | https://ec.europa.eu/eurostat/databrowser/view/TPS00044/default/table?lang=en&category=hlth.hlth_care.hlth_res.hlth_staff%20%2F |
| | |
| **Proportion of health care expenditure on pharmaceuticals** | |
| Data - % of health care expenditure | 24.792 |
| Time | 2020 |
| Reference | https://data.oecd.org/healthres/pharmaceutical-spending.htm |
| | |
| **Number of practising pharmacists per 100,000 inhabitants** | |
| Data - N of practisisng pharmacists per 100,000 inhabitants | 100.64 |
| Time | 2020 |
| Reference | https://ec.europa.eu/eurostat/databrowser/view/HLTH_RS_PRS1__custom_4104351/default/table?lang=en |
| | |
| **Total health care expenditure as percentage of GDP** | |
| Data - % of GDP | 6.27 |
| Time | 2020 |
| Reference | https://ec.europa.eu/eurostat/databrowser/view/TPS00207/default/table?lang=en&category=hlth.hlth_care.hlth_sha11.hlth_sha11_sum |
| | |
| **Public pharmaceutical expenditure as percentage of total pharmaceutical expenditure** | |
| Data - % of total pharmaceutical expenditure | NA |
| Time | NA |
| Reference | NA |
| | |
| **Self-reported consultations of a medical doctor*** | |
| Data - No contact, % of population according to the number of consultations of a medical doctor in the past 4 weeks | 77.1 |
| Data - 1 contact, % of population according to the number of consultations of a medical doctor in the past 4 weeks | 16.5 |
| Data - 2 contacts, % of population according to the number of consultations of a medical doctor in the past 4 weeks | 5.2 |
| Data - 3 or more contacts, % of population according to the number of consultations of a medical doctor in the past 4 weeks | 1.1 |

| | | |
|---|---|---|
| | Time | 2019 |
| | Reference | https://ec.europa.eu/eurostat/databrowser/view/HLTH_EHIS_AM2U/default/table?lang=en&category=hlth.hlth_care.hlth_consult / |

*Medical doctors include generalist medical practitioners and specialist medical practitioners

SERBIA

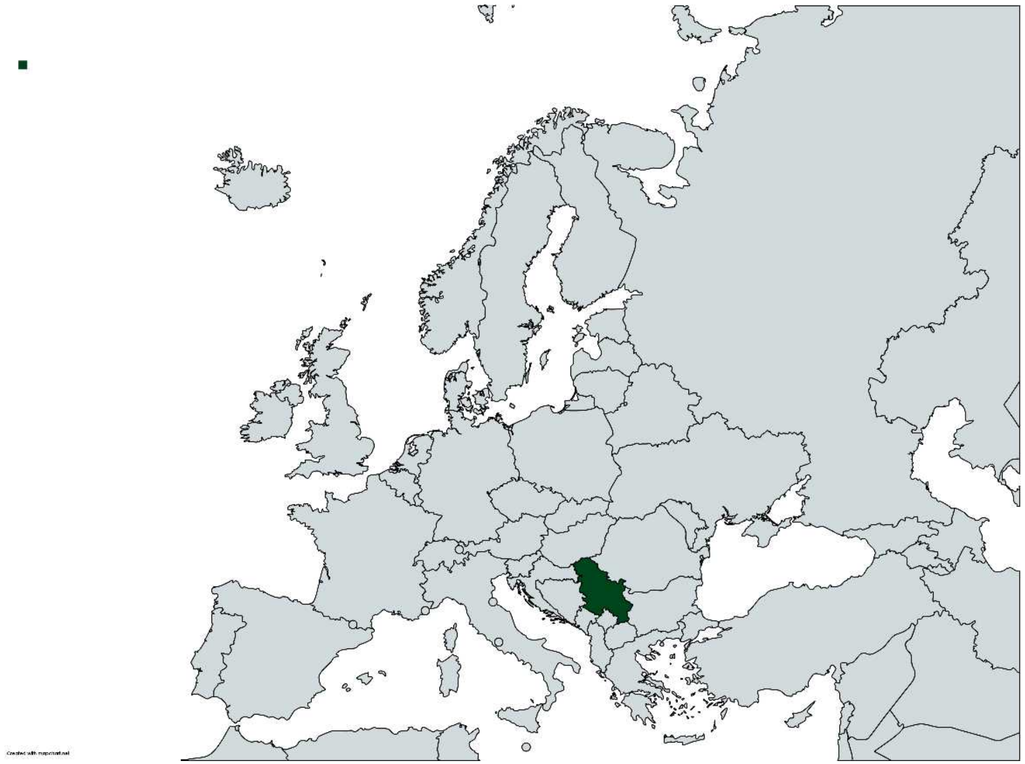

| Country | Serbia |
|---|---|

Country-specific data

| Country characteristics | Method of payment | |
|---|---|---|
| | Data | NA |
| | Time | NA |
| | Reference | NA |
| | | |
| | **Medication adherence assessed and reported on the national level** | |
| | Data | No |
| | Time | 2022 |
| | Reference | NA |
| | | |
| | **Health care provider** | |
| | Data | NA |
| | Time | NA |
| | Reference | NA |
| | | |
| | **Model of healthcare system financing** | |
| | Data | The Serbian Healthcare System is based on the Bismarck Model with certain modifications.Revenue flows to the health system through compulsory health insurance contributions, general taxation, OOP spending, VHI premiums and international donor-based funding initiatives. |
| | Time | 2019 |
| | Reference | file:///C:/Users/HP/Downloads/HiT-21-3-2019-eng.pdf |
| | | |
| | **Proportion of population aged 65 years and over** | |
| | Data - % of persons | 20.6 |
| | Time | 2022 |
| | Reference | https://knoema.com/atlas/Serbia/Population-aged-65-years-and-above |
| | | |
| | **Country population (projection)** | |
| | Data - N of persons | 6 690 887 |
| | Time | 2022 |

| | | |
|---|---|---|
| | Reference | https://popis2022.stat.gov.rs/ |

| | | |
|---|---|---|
| **Social/economic factors** | **Patient co-payment** | |
| | Data | A List: 50, 00 RSD (0.4 €)/pack. A1 List: 10%-95% of retail price |
| | Time | 2018 |
| | Reference | https://ppri.goeg.at/sites/ppri.goeg.at/files/inline-files/Serbia.pdf |
| | | |
| | **Percentage of prescriptions dispensed at no cost to patients** | |
| | Data - % of prescriptions | 14.75% |
| | Time | 2017 |
| | Reference | Expert opinion |
| | | |
| | **Population coverage** | |
| | Data | 0.98 |
| | Time | 2019 |
| | Reference | Expert opinion |
| | | |
| | **Availability of doctors' services for citizens at no payment** | |
| | Data | NA |
| | Time | NA |
| | Reference | NA |
| | | |
| **Therapy-related factors** | **Average number of medicines per patient** | |
| | Data - N of medicines per patient | NA |
| | Time | NA |
| | Reference | NA |
| | | |
| | **Proportion of 75 years and over who are taking more than 5 medications concurrently** | |
| | Data - % of persons | NA |
| | Time | NA |
| | Reference | NA |
| | | |
| | **Percentage of self-reported use of prescribed medicines** | |

| | | |
|---|---|---|
| | Data - % of persons | 45.3 |
| | Time | 2019 |
| | Reference | https://ec.europa.eu/eurostat/databrowser/view/HLTH_EHIS_MD1E__custom_3764895/default/table?lang=en / |

| | | |
|---|---|---|
| Patient-related factors | **Percentage of persons reporting a chronic disease** | |
| | Data - Asthma, % of persons | 3.6 |
| | Data - Chronic lower respiratory diseases, % of persons | 3.5 |
| | Data - High blood pressure, % of persons | 29.6 |
| | Data - Diabetes, % of persons | 7.8 |
| | Data - Chronic depression, % of persons | 4.3 |
| | Time | 2019 |
| | Reference | https://ec.europa.eu/eurostat/databrowser/view/HLTH_EHIS_CD1E/default/table?lang=en&category=hlth.hlth_state.hlth_srcm / |
| | | |
| | **Percentage of self-perceived health - very good (16 years and over)** | |
| | Data - % of persons | 22.7 |
| | Time | 2020 |
| | Reference | https://ec.europa.eu/eurostat/databrowser/view/HLTH_SILC_02/default/table?lang=en&category=hlth.hlth_state.hlth_sph/ |
| | | |
| | **Percenatge of persons with current depressive symptoms** | |
| | Data - % of persons | 2.3 |
| | Time | 2019 |
| | Reference | https://ec.europa.eu/eurostat/databrowser/view/HLTH_EHIS_MH1E/default/table?lang=en&category=hlth.hlth_state.hlth_sph / |

| | | |
|---|---|---|
| Condition-related factor | **General health literacy** | |
| | Data - Inadequate health literacy, % of persons | NA |
| | Data - Problematic health literacy, % of persons | NA |
| | Data - Sufficient health literacy, % of persons | NA |
| | Data - Excellent health literacy, % of persons | NA |
| | Time | NA |
| | Reference | NA |

| | | |
|---|---|---|
| Healthcare system-relat | **Percentage of patients receiving adherence interventions** | |
| | Data - % of persons | NA |

| | |
|---|---|
| Time | NA |
| Reference | NA |
| | |
| **Nationwide availability of e-prescription** | |
| Data | NA |
| Time | NA |
| Reference | NA |
| | |
| **Waiting time for prescriptions / medical appointments** | |
| Data | NA |
| Time | NA |
| Reference | NA |
| | |
| **Number of practising physicians per 100,000 inhabitants** | |
| Data - N of practisisng physicians per 100,000 inhabitants | 301.29 |
| Time | 2021 |
| Reference | https://ec.europa.eu/eurostat/databrowser/view/TPS00044/default/table?lang=en&category=hlth.hlth_care.hlth_res.hlth_staff%20%2F |
| | |
| **Proportion of health care expenditure on pharmaceuticals** | |
| Data - % of health care expenditure | 32.59 |
| Time | 2017 |
| Reference | Expert opinion |
| | |
| **Number of practising pharmacists per 100,000 inhabitants** | |
| Data - N of practisisng pharmacists per 100,000 inhabitants | 15.69 |
| Time | 2021 |
| Reference | https://ec.europa.eu/eurostat/databrowser/view/HLTH_RS_PRS1__custom_4104351/default/table?lang=en |
| | |
| **Total health care expenditure as percentage of GDP** | |
| Data - % of GDP | NA |
| Time | NA |
| Reference | NA |
| | |

| | | |
|---|---|---|
| | **Public pharmaceutical expenditure as percentage of total pharmaceutical expenditure** | |
| | Data - % of total pharmaceutical expenditure | 34.3 |
| | Time | 2018 |
| | Reference | https://gateway.euro.who.int/en/indicators/hfa_580-6790-public-pharmaceutical-expenditure-as-of-total-pharmaceutical-expenditure/visualizations/#id=19675&tab=table |
| | | |
| | **Self-reported consultations of a medical doctor*** | |
| | Data - No contact, % of population according to the number of consultations of a medical doctor in the past 4 weeks | 55.6 |
| | Data - 1 contact, % of population according to the number of consultations of a medical doctor in the past 4 weeks | 23.8 |
| | Data - 2 contacts, % of population according to the number of consultations of a medical doctor in the past 4 weeks | 9.5 |
| | Data - 3 or more contacts, % of population according to the number of consultations of a medical doctor in the past 4 weeks | 11 |
| | Time | 2019 |
| | Reference | https://ec.europa.eu/eurostat/databrowser/view/HLTH_EHIS_AM2U/default/table?lang=en&category=hlth.hlth_care.hlth_consult / |

*Medical doctors include generalist medical practitioners and specialist medical practitioners

SLOVAKIA

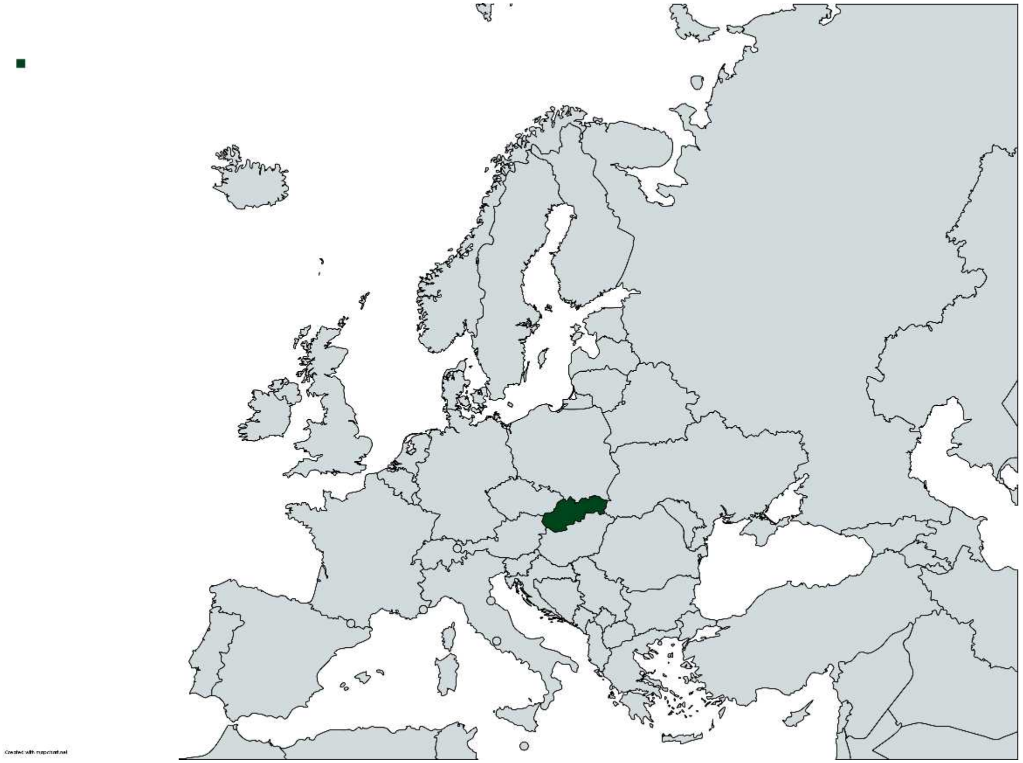

| Country | Slovakia |
|---|---|

Country-specific data

| Country characteristics | Method of payment | |
|---|---|---|
| | Data | NA |
| | Time | NA |
| | Reference | NA |
| | | |
| | Medication adherence assessed and reported on the national level | |
| | Data | No |
| | Time | 2022 |
| | Reference | NA |
| | | |
| | Health care provider | |
| | Data | NA |
| | Time | NA |
| | Reference | NA |
| | | |
| | Model of healthcare system financing | |
| | Data | NA |
| | Time | NA |
| | Reference | NA |
| | | |
| | Proportion of population aged 65 years and over | |
| | Data - % of persons | 17.1 |
| | Time | 2021 |
| | Reference | https://ec.europa.eu/eurostat/databrowser/view/TPS00028/default/table?lang=en&category=demo.demo_ind / |
| | | |
| | Country population (projection) | |
| | Data - N of persons | 5457679 |
| | Time | 2020 |
| | Reference | https://ec.europa.eu/eurostat/databrowser/view/CENS_HNMGA/default/table?lang=en&category=cens.cens_hn.cens_hnstr |

| | | |
|---|---|---|
| Social/economic factors | **Patient co-payment** | |
| | Data | NA |
| | Time | NA |
| | Reference | NA |
| | | |
| | **Percentage of prescriptions dispensed at no cost to patients** | |
| | Data - % of prescriptions | NA |
| | Time | NA |
| | Reference | NA |
| | | |
| | **Population coverage** | |
| | Data | NA |
| | Time | NA |
| | Reference | NA |
| | | |
| | **Availability of doctors' services for citizens at no payment** | |
| | Data | NA |
| | Time | NA |
| | Reference | NA |
| | | |
| Therapy-related factors | **Average number of medicines per patient** | |
| | Data - N of medicines per patient | NA |
| | Time | NA |
| | Reference | NA |
| | | |
| | **Proportion of 75 years and over who are taking more than 5 medications concurrently** | |
| | Data - % of persons | NA |
| | Time | NA |
| | Reference | NA |
| | | |
| | **Percentage of self-reported use of prescribed medicines** | |
| | Data - % of persons | 45.9 |

| | | |
|---|---|---|
| | Time | 2019 |
| | Reference | https://ec.europa.eu/eurostat/databrowser/view/HLTH_EHIS_MD1E__custom_3764895/default/table?lang=en / |
| **Patient-related factors** | **Percentage of persons reporting a chronic disease** | |
| | Data - Asthma, % of persons | 4 |
| | Data - Chronic lower respiratory diseases, % of persons | 2.9 |
| | Data - High blood pressure, % of persons | 28.4 |
| | Data - Diabetes, % of persons | 7.6 |
| | Data - Chronic depression, % of persons | 4.3 |
| | Time | 2019 |
| | Reference | https://ec.europa.eu/eurostat/databrowser/view/HLTH_EHIS_CD1E/default/table?lang=en&category=hlth.hlth_state.hlth_srcm / |
| | | |
| | **Percentage of self-perceived health - very good (16 years and over)** | |
| | Data - % of persons | 20.3 |
| | Time | 2020 |
| | Reference | https://ec.europa.eu/eurostat/databrowser/view/HLTH_SILC_02/default/table?lang=en&category=hlth.hlth_state.hlth_sph/ |
| | | |
| | **Percenatge of persons with current depressive symptoms** | |
| | Data - % of persons | 3.2 |
| | Time | 2019 |
| | Reference | https://ec.europa.eu/eurostat/databrowser/view/HLTH_EHIS_MH1E/default/table?lang=en&category=hlth.hlth_state.hlth_sph / |
| **Condition-related factor** | **General health literacy** | |
| | Data - Inadequate health literacy, % of persons | 23 |
| | Data - Problematic health literacy, % of persons | 36 |
| | Data - Sufficient health literacy, % of persons | 31 |
| | Data - Excellent health literacy, % of persons | 10 |
| | Time | 2021 |
| | Reference | https://m-pohl.net/Int_Report_methdology_results_recommendations |
| **Healthcare system-relat** | **Percentage of patients receiving adherence interventions** | |
| | Data - % of persons | NA |
| | Time | NA |

| | |
|---|---|
| Reference | NA |
| | |
| **Nationwide availability of e-prescription** | |
| Data | NA |
| Time | NA |
| Reference | NA |
| | |
| **Waiting time for prescriptions / medical appointments** | |
| Data | NA |
| Time | NA |
| Reference | NA |
| | |
| **Number of practising physicians per 100,000 inhabitants** | |
| Data - N of practisisng physicians per 100,000 inhabitants | NA |
| Time | NA |
| Reference | NA |
| | |
| **Proportion of health care expenditure on pharmaceuticals** | |
| Data - % of health care expenditure | 23.626 |
| Time | 2020 |
| Reference | https://data.oecd.org/healthres/pharmaceutical-spending.htm |
| | |
| **Number of practising pharmacists per 100,000 inhabitants** | |
| Data - N of practisisng pharmacists per 100,000 inhabitants | NA |
| Time | NA |
| Reference | NA |
| | |
| **Total health care expenditure as percentage of GDP** | |
| Data - % of GDP | 7.23 |
| Time | 2020 |
| Reference | https://ec.europa.eu/eurostat/databrowser/view/TPS00207/default/table?lang=en&category=hlth.hlth_care.hlth_sha11.hlth_sha11_sum |
| | |
| **Public pharmaceutical expenditure as percentage of total pharmaceutical expenditure** | |

| | | |
|---|---|---|
| | Data - % of total pharmaceutical expenditure | 69.4 |
| | Time | 2011 |
| | Reference | https://gateway.euro.who.int/en/indicators/hfa_580-6790-public-pharmaceutical-expenditure-as-of-total-pharmaceutical-expenditure/visualizations/#id=19675&tab=table |
| | | |
| | **Self-reported consultations of a medical doctor*** | |
| | Data - No contact, % of population according to the number of consultations of a medical doctor in the past 4 weeks | 54.5 |
| | Data - 1 contact, % of population according to the number of consultations of a medical doctor in the past 4 weeks | 19 |
| | Data - 2 contacts, % of population according to the number of consultations of a medical doctor in the past 4 weeks | 14.1 |
| | Data - 3 or more contacts, % of population according to the number of consultations of a medical doctor in the past 4 weeks | 12.4 |
| | Time | 2019 |
| | Reference | https://ec.europa.eu/eurostat/databrowser/view/HLTH_EHIS_AM2U/default/table?lang=en&category=hlth.hlth_care.hlth_consult / |

*Medical doctors include generalist medical practitioners and specialist medical practitioners

SLOVENIA

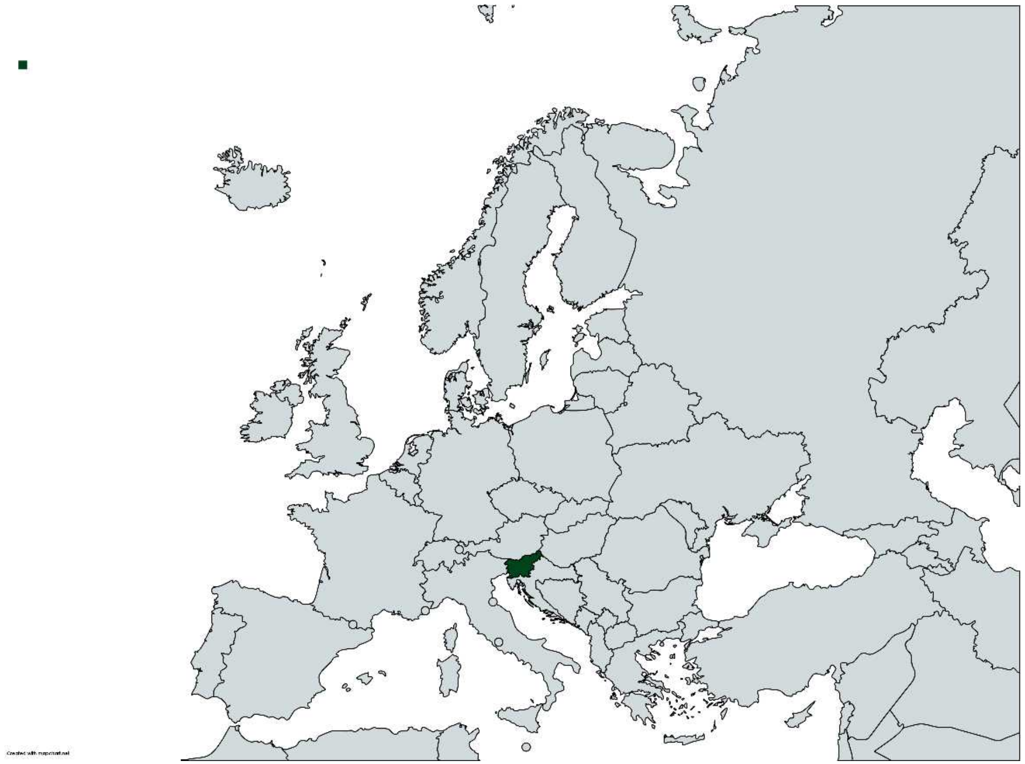

| Country | Slovenia |
|---|---|

**Country-specific data**

| Country characteristics | Method of payment | |
|---|---|---|
| | Data | HIIS is the payer and reimburses the medicines based on the approved reimbursment scheme which has three lists. The scheme has two major sources - compulsory health insurance managed by HIIS and voluntary healht insurance managed by three insurnace companies. Depending on the list the medicines are reimbursed in full (100% compulsory), partly (70% from compulsory or 10% compulsory). If the patient does not have the voulntary insurance they pay the difference from pocket. The patients pay full price for medicines, which do not get on any of the lists. And there are also co-payments for payments about a set price for certain groups of medicines - generic medicine exchange and therapeutics price exchange |
| | Time | 2017-2018 |
| | Reference | Mardetko et al. Development and performance of the external reference pricing system in Slovenia from 2007 to 2012. Health policy and technology. 2017, vol. 4, iss. 3, str. 348-357, iDOI: 10.1016/j.hlpt.2017.06.005.<br>Mardetko N. Introduction of therapeutic reference pricing in Slovenia and its economic consequences. The European journal of health economics. 2018, vol. 19, iss. 4, str. 571-584; DOI: 10.1007/s10198-017-0903-9.<br>Mardetko N. Influence of generic reference pricing on medicine cost in Slovenia : a retrospective study. Croatian medical journal online. 2018, vol. 59, no. 2, str. 79-89 DOI: 10.3325/cmj.2018.59.79. |
| | | |
| | Medication adherence assessed and reported on the national level | |
| | Data | No |
| | Time | 2022 |
| | Reference | NA |
| | | |
| | Health care provider | |
| | Data | HIIS is not a healthcare provider, it's the healthcare payer. Not sure what you mean by the category healthcare provider. In Slovenia we have three levels of healthcare providers (primary, secondary, tertiary).Mostly providers are public, meaning primary level institutions are established by municipalities (e.g. primary care centres, community pharmacies) or municipalities issue approval to provide public health service to concessionaries (privately owned institutions, providing public health service). Secondary and tertiary level care is established by the state. Public healthcare services are funded through HIIS, depending on the reimbursment scheme. |
| | Time | 20.1.2023 |
| | Reference | https://www.gov.si/novice/2023-01-20-zdravstvo-bomo-pripravili-na-vse-izzive/ |
| | | |
| | Model of healthcare system financing | |
| | Data | HIIS (64%); voluntary health insurance (13,5%); state (9%); other (e.g. out of pocket) (13,5%) |
| | Time | 20.1.2023 |
| | Reference | https://www.gov.si/novice/2023-01-20-zdravstvo-bomo-pripravili-na-vse-izzive/ |

| | | |
|---|---|---|
| | | |
| | **Proportion of population aged 65 years and over** | |
| | Data - % of persons | 21.3 |
| | Time | 2022 |
| | Reference | https://www.stat.si/StatWeb/en/Field/Index/17/104 |
| | | |
| | **Country population (projection)** | |
| | Data - N of persons | 2110547 |
| | Time | 2022 |
| | Reference | https://www.stat.si/StatWeb/en/Field/Index/17/104 |

| | | |
|---|---|---|
| Social/economic factors | **Patient co-payment** | |
| | Data | Copayments are dependant on which medicine list the medicine is listed. There are two positive lists. A P100, where medicine is 100% reimbursment from compulsory health insurance (HIIS); P70% where HIIS covers 70% of the price and 30% is from voluntary health insurance or as a copayment. Then there is intermediate list where the % is usually 10% from compulsory and 90% from voluntary/out of pocket. And then there are medicines which can be prescribed. but are not reimbursed so 100% of the price is out of pocket. We have systems of generic and therapeutic reference pricing in this case if the medicine price is higher than the reference price, the patient would co-pay. However, there is always a possibility for the patient to receive the medicine within the reference price (without copayement).H16 |
| | Time | 2018 |
| | Reference | Mardetko et al. Development and performance of the external reference pricing system in Slovenia from 2007 to 2012. Health policy and technology. 2017, vol. 4, iss. 3, str. 348-357, iDOI: 10.1016/j.hlpt.2017.06.005.<br>Mardetko N. Introduction of therapeutic reference pricing in Slovenia and its economic consequences. The European journal of health economics. 2018, vol. 19, iss. 4, str. 571-584; DOI: 10.1007/s10198-017-0903-9.<br>Mardetko N. Influence of generic reference pricing on medicine cost in Slovenia : a retrospective study. Croatian medical journal online. 2018, vol. 59, no. 2, str. 79-89 DOI: 10.3325/cmj.2018.59.79. |
| | | |
| | **Percentage of prescriptions dispensed at no cost to patients** | |
| | Data - % of prescriptions | 51.9% of medicines from positive lists; 66% of all prescribed medicines costs were covered by compulsary health insurance |
| | Time | 2022 |
| | Reference | HIIS Annual report 2022 (https://www.zzzs.si/?id=126&detail=538C71578F0FD063C125896C0047B208) |
| | | |
| | **Population coverage** | |
| | Data | 0.99 |
| | Time | 2013 |
| | Reference | http://imi-protect.eu/documents/DUinventory_2013_COUNTRIESyear4_Dec2013.pdf |
| | | |

| | | |
|---|---|---|
| | **Availability of doctors' services for citizens at no payment** | |
| | Data | All patients can choose a designated general practitioner or a peadiatrician for children. Women can choose designated gynecologist. This is covered by the compulsory health insurance. The GP can then refer you to the specialists. In geenral, if you have both insurances (compulsory and voluntary) the majority of the healthcare services are free of charge. This is depenedant on the list of services, that both insurance cover. |
| | Time | 2023 |
| | Reference | Ministry of Health report 2023 https://www.gov.si/novice/2023-01-20-zdravstvo-bomo-pripravili-na-vse-izzive/ |
| | | |
| **Therapy-related factors** | **Average number of medicines per patient** | |
| | Data - N of medicines per patient | 8.9 |
| | Time | 2022 |
| | Reference | HIIS Annual report 2022 (https://www.zzzs.si/?id=126&detail=538C71578F0FD063C125896C0047B208) |
| | | |
| | **Proportion of 75 years and over who are taking more than 5 medications concurrently** | |
| | Data - % of persons | 53.1 |
| | Time | 2019 |
| | Reference | https://stats.oecd.org/Index.aspx?ThemeTreeId=42 |
| | | |
| | **Percentage of self-reported use of prescribed medicines** | |
| | Data - % of persons | 47.8 |
| | Time | 2019 |
| | Reference | https://ec.europa.eu/eurostat/databrowser/view/HLTH_EHIS_MD1E__custom_3764895/default/table?lang=en / |
| | | |
| **Patient-related factors** | **Percentage of persons reporting a chronic disease** | |
| | Data - Asthma, % of persons | 4.8 |
| | Data - Chronic lower respiratory diseases, % of persons | 3.6 |
| | Data - High blood pressure, % of persons | 25.4 |
| | Data - Diabetes, % of persons | 7.8 |
| | Data - Chronic depression, % of persons | 7.9 |
| | Time | 2019 |
| | Reference | https://ec.europa.eu/eurostat/databrowser/view/HLTH_EHIS_CD1E/default/table?lang=en&category=hlth.hlth_state.hlth_srcm / |
| | | |
| | **Percentage of self-perceived health - very good (16 years and over)** | |
| | Data - % of persons | 22 |

| | | |
|---|---|---|
| | Time | 2022 |
| | Reference | https://www.stat.si/StatWeb/en/Field/Index/10/117 |
| | | |
| | **Percenatge of persons with current depressive symptoms** | |
| | Data - % of persons | 7.5 |
| | Time | 2019 |
| | Reference | https://ec.europa.eu/eurostat/databrowser/view/HLTH_EHIS_MH1E/default/table?lang=en&category=hlth.hlth_state.hlth_sph / |

| | | |
|---|---|---|
| **Condition-related factor** | **General health literacy** | |
| | Data - Inadequate health literacy, % of persons | 7 |
| | Data - Problematic health literacy, % of persons | 18 |
| | Data - Sufficient health literacy, % of persons | 53 |
| | Data - Excellent health literacy, % of persons | 23 |
| | Time | 2021 |
| | Reference | https://m-pohl.net/Int_Report_methdology_results_recommendations |

| | | |
|---|---|---|
| **Healthcare system-relat** | **Percentage of patients receiving adherence interventions** | |
| | Data - % of persons | There are two pharmacist services: MUR in community pharmacies and Clinical medication review in primary and secondary care, which address adhrence. However there are no publicaly available data on how many interventison are perfomed per year. |
| | Time | 2023 |
| | Reference | Expert opinion |
| | | |
| | **Nationwide availability of e-prescription** | |
| | Data | Yes |
| | Time | 2022 |
| | Reference | HIIS Annual report 2022 (https://www.zzzs.si/?id=126&detail=538C71578F0FD063C125896C0047B208); Ministry of Health report 2023 https://www.gov.si/novice/2023-01-20-zdravstvo-bomo-pripravili-na-vse-izzive/ |
| | | |
| | **Waiting time for prescriptions / medical appointments** | |
| | Data | NA |
| | Time | NA |
| | Reference | NA |
| | | |
| | **Number of practising physicians per 100,000 inhabitants** | |

| | |
|---|---|
| Data - N of practisisng physicians per 100,000 inhabitants | 330.29 |
| Time | 2020 |
| Reference | https://ec.europa.eu/eurostat/databrowser/view/TPS00044/default/table?lang=en&category=hlth.hlth_care.hlth_res.hlth_staff%20%2F |
| | |
| **Proportion of health care expenditure on pharmaceuticals** | |
| Data - % of health care expenditure | 17.143 |
| Time | 2020 |
| Reference | https://data.oecd.org/healthres/pharmaceutical-spending.htm |
| | |
| **Number of practising pharmacists per 100,000 inhabitants** | |
| Data - N of practisisng pharmacists per 100,000 inhabitants | 74.06 |
| Time | 2020 |
| Reference | https://ec.europa.eu/eurostat/databrowser/view/HLTH_RS_PRS1__custom_4104351/default/table?lang=en |
| | |
| **Total health care expenditure as percentage of GDP** | |
| Data - % of GDP | 9.45 |
| Time | 2020 |
| Reference | https://ec.europa.eu/eurostat/databrowser/view/TPS00207/default/table?lang=en&category=hlth.hlth_care.hlth_sha11.hlth_sha11_sum |
| | |
| **Public pharmaceutical expenditure as percentage of total pharmaceutical expenditure** | |
| Data - % of total pharmaceutical expenditure | 51.1 |
| Time | 2019 |
| Reference | https://gateway.euro.who.int/en/indicators/hfa_580-6790-public-pharmaceutical-expenditure-as-of-total-pharmaceutical-expenditure/visualizations/#id=19675&tab=table |
| | |
| **Self-reported consultations of a medical doctor*** | |
| Data - No contact, % of population according to the number of consultations of a medical doctor in the past 4 weeks | 62.6 |
| Data - 1 contact, % of population according to the number of consultations of a medical doctor in the past 4 weeks | 18.1 |
| Data - 2 contacts, % of population according to the number of consultations of a medical doctor in the past 4 weeks | 10.4 |

| | | |
|---|---|---|
| | Data - 3 or more contacts, % of population according to the number of consultations of a medical doctor in the past 4 weeks | 8.8 |
| | Time | 2019 |
| | Reference | https://ec.europa.eu/eurostat/databrowser/view/HLTH_EHIS_AM2U/default/table?lang=en&category=hlth.hlth_care.hlth_consult / |

*Medical doctors include generalist medical practitioners and specialist medical practitioners

SPAIN

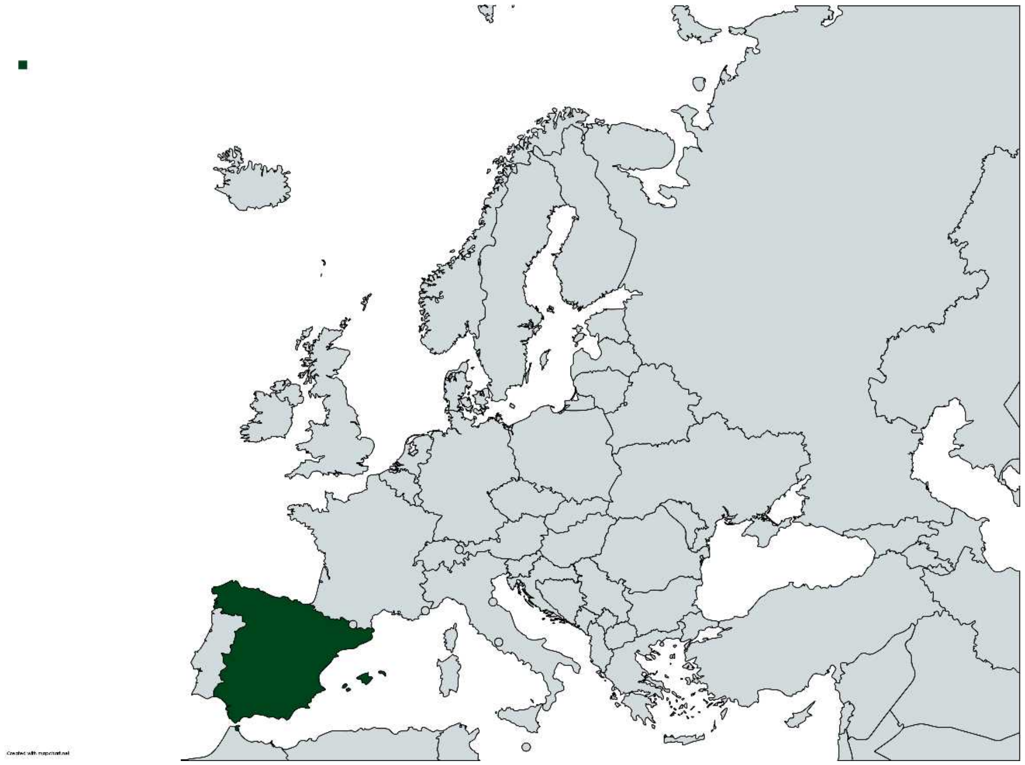

| Country | Spain |
|---|---|

**Country-specific data**

| Country characteristics | Method of payment | |
|---|---|---|
| | Data | The National Health System (SNS) partially pays reimbursed medicines. Patients pay the rest. |
| | Time | 2013 |
| | Reference | http://imi-protect.eu/documents/DUinventory_2013_COUNTRIESyear4_Dec2013.pdf |
| | | |
| | **Medication adherence assessed and reported on the national level** | |
| | Data | No |
| | Time | 2022 |
| | Reference | NA |
| | | |
| | **Health care provider** | |
| | Data | Public health sector. Universal access to health services. |
| | Time | 2013 |
| | Reference | http://imi-protect.eu/documents/DUinventory_2013_COUNTRIESyear4_Dec2013.pdf |
| | | |
| | **Model of healthcare system financing** | |
| | Data | Highly decentralised model with the allocation of block grants – obtained through taxation-, from the central government to the autonomous communities, except for Navarre and the Basque Country with high autonomy taxation. Taxation represents 94.1% of the funding of the social security system. |
| | Time | 2013 |
| | Reference | http://imi-protect.eu/documents/DUinventory_2013_COUNTRIESyear4_Dec2013.pdf |
| | | |
| | **Proportion of population aged 65 years and over** | |
| | Data - % of persons | 19.8 |
| | Time | 2021 |
| | Reference | https://ec.europa.eu/eurostat/databrowser/view/TPS00028/default/table?lang=en&category=demo.demo_ind / |
| | | |
| | **Country population (projection)** | |
| | Data - N of persons | 47321434 |
| | Time | 2020 |

| | | |
|---|---|---|
| | Reference | https://ec.europa.eu/eurostat/databrowser/view/CENS_HNMGA/default/table?lang=en&category=cens.cens_hn.cens_hnstr |
| | | |
| Social/economic factors | **Patient co-payment** | |
| | Data | Retired people pay 10% of the medicines price with a monthly maximum depending on annual income :> €100,000, copayment is 60%; < €18,000 (max per month €8), between >€18,000-<€100,000 (max per month €18), >€100,000 (max per month €60). Employees and beneficiaries copayment rate based on their annual income: < €18,000 40% of the medicines price; >€18,000-<€100,000 50%; >€100,000 (60%). Exemptions for people with toxic syndrome and other disabilities, on social cash aid, retired with non-contributory pensions, unemployed not receiving any social aid, work derived diseases or injuries. For specific treatments copayment is 10% up to a maximum of €4.13/package dispensed. Some food products no copayment after a medical application and approval. There are regional variations in flat-fees per prescription. Reimbursable scheme applied in 2012. |
| | Time | 2013 |
| | Reference | http://imi-protect.eu/documents/DUinventory_2013_COUNTRIESyear4_Dec2013.pdf |
| | | |
| | **Percentage of prescriptions dispensed at no cost to patients** | |
| | Data - % of prescriptions | NA |
| | Time | NA |
| | Reference | NA |
| | | |
| | **Population coverage** | |
| | Data | 99.5% including low-income inhabitants. Civil servants can opt out of the public financed system. 88% of this population and their beneficiaries are covered for non-for-profit private sector. 13% of the Spanish population are covered by private-for-profit voluntary health insurance, with an important regional variation. |
| | Time | 2013 |
| | Reference | http://imi-protect.eu/documents/DUinventory_2013_COUNTRIESyear4_Dec2013.pdf |
| | | |
| | **Availability of doctors' services for citizens at no payment** | |
| | Data | NA |
| | Time | NA |
| | Reference | NA |
| | | |
| Therapy-related factors | **Average number of medicines per patient** | |
| | Data - N of medicines per patient | NA |
| | Time | NA |
| | Reference | NA |

| | | |
|---|---|---|
| | | |
| | **Proportion of 75 years and over who are taking more than 5 medications concurrently** | |
| | Data - % of persons | NA |
| | Time | NA |
| | Reference | NA |
| | | |
| | **Percentage of self-reported use of prescribed medicines** | |
| | Data - % of persons | 50.9 |
| | Time | 2019 |
| | Reference | https://ec.europa.eu/eurostat/databrowser/view/HLTH_EHIS_MD1E__custom_3764895/default/table?lang=en / |

| | | |
|---|---|---|
| Patient-related factors | **Percentage of persons reporting a chronic disease** | |
| | Data - Asthma, % of persons | 4.1 |
| | Data - Chronic lower respiratory diseases, % of persons | 2.7 |
| | Data - High blood pressure, % of persons | 19.3 |
| | Data - Diabetes, % of persons | 7.5 |
| | Data - Chronic depression, % of persons | 5.7 |
| | Time | 2019 |
| | Reference | https://ec.europa.eu/eurostat/databrowser/view/HLTH_EHIS_CD1E/default/table?lang=en&category=hlth.hlth_state.hlth_srcm / |
| | | |
| | **Percentage of self-perceived health - very good (16 years and over)** | |
| | Data - % of persons | 17.8 |
| | Time | 2021 |
| | Reference | https://ec.europa.eu/eurostat/databrowser/view/HLTH_SILC_02/default/table?lang=en&category=hlth.hlth_state.hlth_sph/ |
| | | |
| | **Percenatge of persons with current depressive symptoms** | |
| | Data - % of persons | 4.8 |
| | Time | 2019 |
| | Reference | https://ec.europa.eu/eurostat/databrowser/view/HLTH_EHIS_MH1E/default/table?lang=en&category=hlth.hlth_state.hlth_sph / |

| | | |
|---|---|---|
| Condition-related factor: | **General health literacy** | |
| | Data - Inadequate health literacy, % of persons | 7.5 |
| | Data - Problematic health literacy, % of persons | 50.8 |

| | | |
|---|---|---|
| | Data - Sufficient health literacy, % of persons | 32.6 |
| | Data - Excellent health literacy, % of persons | 9.1 |
| | Time | 2011 |
| | Reference | https://academic.oup.com/eurpub/article/25/6/1053/2467145 |

| | | |
|---|---|---|
| **Healthcare system-relat** | **Percentage of patients receiving adherence interventions** | |
| | Data - % of persons | NA |
| | Time | NA |
| | Reference | NA |
| | | |
| | **Nationwide availability of e-prescription** | |
| | Data | NA |
| | Time | NA |
| | Reference | NA |
| | | |
| | **Waiting time for prescriptions / medical appointments** | |
| | Data | NA |
| | Time | NA |
| | Reference | NA |
| | | |
| | **Number of practising physicians per 100,000 inhabitants** | |
| | Data - N of practisisng physicians per 100,000 inhabitants | 457.67 |
| | Time | 2020 |
| | Reference | https://ec.europa.eu/eurostat/databrowser/view/TPS00044/default/table?lang=en&category=hlth.hlth_care.hlth_res.hlth_staff%20%2F |
| | | |
| | **Proportion of health care expenditure on pharmaceuticals** | |
| | Data - % of health care expenditure | 15.069 |
| | Time | 2020 |
| | Reference | https://data.oecd.org/healthres/pharmaceutical-spending.htm |
| | | |
| | **Number of practising pharmacists per 100,000 inhabitants** | |
| | Data - N of practisisng pharmacists per 100,000 inhabitants | 131.61 |
| | Time | 2020 |

| | | |
|---|---|---|
| | Reference | https://ec.europa.eu/eurostat/databrowser/view/HLTH_RS_PRS1__custom_4104351/default/table?lang=en |
| | | |
| | **Total health care expenditure as percentage of GDP** | |
| | Data - % of GDP | 10.71 |
| | Time | 2020 |
| | Reference | https://ec.europa.eu/eurostat/databrowser/view/TPS00207/default/table?lang=en&category=hlth.hlth_care.hlth_sha11.hlth_sha11_sum |
| | | |
| | **Public pharmaceutical expenditure as percentage of total pharmaceutical expenditure** | |
| | Data - % of total pharmaceutical expenditure | 71 |
| | Time | 2011 |
| | Reference | https://gateway.euro.who.int/en/indicators/hfa_580-6790-public-pharmaceutical-expenditure-as-of-total-pharmaceutical-expenditure/visualizations/#id=19675&tab=table |
| | | |
| | **Self-reported consultations of a medical doctor*** | |
| | Data - No contact, % of population according to the number of consultations of a medical doctor in the past 4 weeks | 74.9 |
| | Data - 1 contact, % of population according to the number of consultations of a medical doctor in the past 4 weeks | 16.4 |
| | Data - 2 contacts, % of population according to the number of consultations of a medical doctor in the past 4 weeks | 5.6 |
| | Data - 3 or more contacts, % of population according to the number of consultations of a medical doctor in the past 4 weeks | 3.1 |
| | Time | 2019 |
| | Reference | https://ec.europa.eu/eurostat/databrowser/view/HLTH_EHIS_AM2U/default/table?lang=en&category=hlth.hlth_care.hlth_consult / |

*Medical doctors include generalist medical practitioners and specialist medical practitioners

SWEDEN

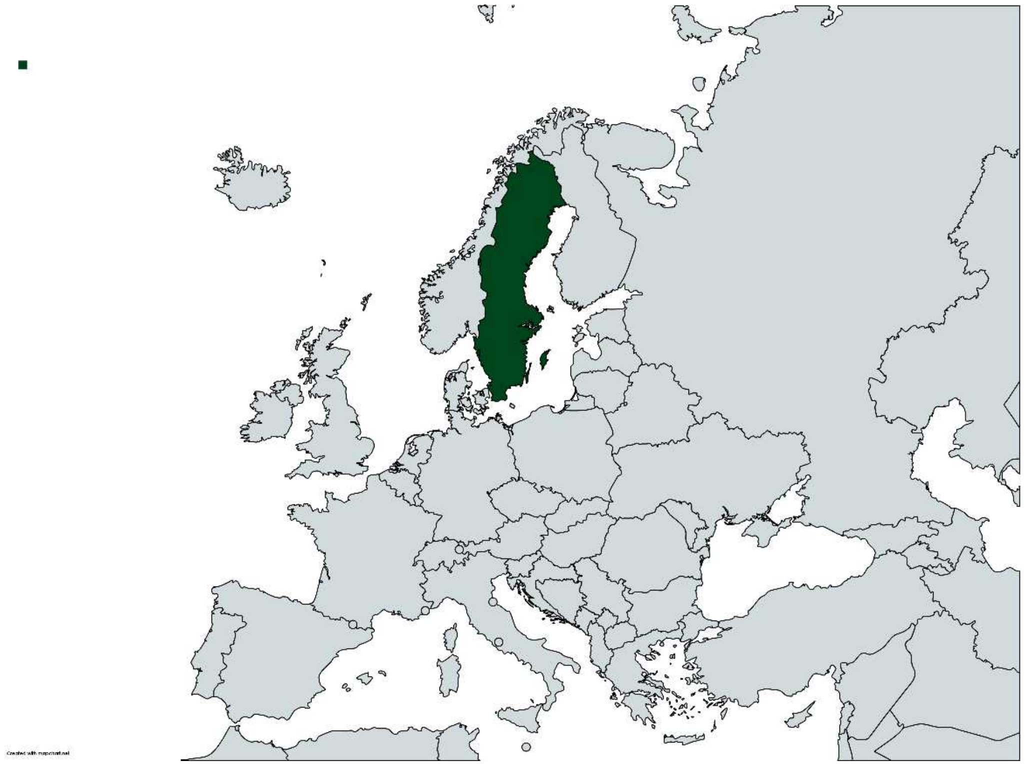

| Country | Sweden |
|---|---|

Country-specific data

| Country characteristics | Method of payment | |
|---|---|---|
| | Data | The county councils partially pay the medicines direct to the pharmacists. |
| | Time | 2013 |
| | Reference | http://imi-protect.eu/documents/DUinventory_2013_COUNTRIESyear4_Dec2013.pdf |
| | | |
| | Medication adherence assessed and reported on the national level | |
| | Data | No |
| | Time | 2022 |
| | Reference | NA |
| | | |
| | Health care provider | |
| | Data | Public: Universal access to health care. |
| | Time | 2013 |
| | Reference | http://imi-protect.eu/documents/DUinventory_2013_COUNTRIESyear4_Dec2013.pdf |
| | | |
| | Model of healthcare system financing | |
| | Data | Mainly proportional income taxes (80%), supplemented by Government grants and patient copayment (17%). |
| | Time | 2013 |
| | Reference | http://imi-protect.eu/documents/DUinventory_2013_COUNTRIESyear4_Dec2013.pdf |
| | | |
| | Proportion of population aged 65 years and over | |
| | Data - % of persons | 20.1 |
| | Time | 2021 |
| | Reference | https://ec.europa.eu/eurostat/databrowser/view/TPS00028/default/table?lang=en&category=demo.demo_ind / |
| | | |
| | Country population (projection) | |
| | Data - N of persons | 10322613 |
| | Time | 2020 |
| | Reference | https://ec.europa.eu/eurostat/databrowser/view/CENS_HNMGA/default/table?lang=en&category=cens.cens_hn.cens_hnstr |

| | | |
|---|---|---|
| Social/economic factors | **Patient co-payment** | |
| | Data | Patients pay the full cost for prescribed and reimbursed medicines up to a ceiling. Thereafter the copayment is progressively reduced according to his/her pharmaceutical expenditure. 50%, 25%, and 10%. The patient will pay for prescribed drugs a maximum of EUR244 annually. |
| | Time | 2013 |
| | Reference | http://imi-protect.eu/documents/DUinventory_2013_COUNTRIESyear4_Dec2013.pdf |
| | | |
| | **Percentage of prescriptions dispensed at no cost to patients** | |
| | Data - % of prescriptions | NA |
| | Time | NA |
| | Reference | NA |
| | | |
| | **Population coverage** | |
| | Data | All residents in Sweden regardless of their nationality. Around 2.3% of Swedes have a private medical insurance. |
| | Time | 2013 |
| | Reference | http://imi-protect.eu/documents/DUinventory_2013_COUNTRIESyear4_Dec2013.pdf |
| | | |
| | **Availability of doctors' services for citizens at no payment** | |
| | Data | NA |
| | Time | NA |
| | Reference | NA |
| | | |
| Therapy-related factors | **Average number of medicines per patient** | |
| | Data - N of medicines per patient | NA |
| | Time | NA |
| | Reference | NA |
| | | |
| | **Proportion of 75 years and over who are taking more than 5 medications concurrently** | |
| | Data - % of persons | 44.3 |
| | Time | 2019 |
| | Reference | https://stats.oecd.org/Index.aspx?ThemeTreeId=44 |
| | | |
| | **Percentage of self-reported use of prescribed medicines** | |

| | | |
|---|---|---|
| | Data - % of persons | 48.5 |
| | Time | 2019 |
| | Reference | https://ec.europa.eu/eurostat/databrowser/view/HLTH_EHIS_MD1E__custom_3764895/default/table?lang=en / |

| | | |
|---|---|---|
| **Patient-related factors** | **Percentage of persons reporting a chronic disease** | |
| | Data - Asthma, % of persons | 7.5 |
| | Data - Chronic lower respiratory diseases, % of persons | 1.6 |
| | Data - High blood pressure, % of persons | 18.2 |
| | Data - Diabetes, % of persons | 6.3 |
| | Data - Chronic depression, % of persons | 11.7 |
| | Time | 2019 |
| | Reference | https://ec.europa.eu/eurostat/databrowser/view/HLTH_EHIS_CD1E/default/table?lang=en&category=hlth.hlth_state.hlth_srcm / |
| | | |
| | **Percentage of self-perceived health - very good (16 years and over)** | |
| | Data - % of persons | 24.4 |
| | Time | 2021 |
| | Reference | https://ec.europa.eu/eurostat/databrowser/view/HLTH_SILC_02/default/table?lang=en&category=hlth.hlth_state.hlth_sph/ |
| | | |
| | **Percenatge of persons with current depressive symptoms** | |
| | Data - % of persons | 10.5 |
| | Time | 2019 |
| | Reference | https://ec.europa.eu/eurostat/databrowser/view/HLTH_EHIS_MH1E/default/table?lang=en&category=hlth.hlth_state.hlth_sph / |

| | | |
|---|---|---|
| **Condition-related factor** | **General health literacy** | |
| | Data - Inadequate health literacy, % of persons | NA |
| | Data - Problematic health literacy, % of persons | NA |
| | Data - Sufficient health literacy, % of persons | NA |
| | Data - Excellent health literacy, % of persons | NA |
| | Time | NA |
| | Reference | NA |

| | | |
|---|---|---|
| **Healthcare system-relat** | **Percentage of patients receiving adherence interventions** | |
| | Data - % of persons | NA |

| | |
|---|---|
| Time | NA |
| Reference | NA |
| | |
| **Nationwide availability of e-prescription** | |
| Data | NA |
| Time | NA |
| Reference | NA |
| | |
| **Waiting time for prescriptions / medical appointments** | |
| Data | NA |
| Time | NA |
| Reference | NA |
| | |
| **Number of practising physicians per 100,000 inhabitants** | |
| Data - N of practisisng physicians per 100,000 inhabitants | 428.51 |
| Time | 2019 |
| Reference | https://ec.europa.eu/eurostat/databrowser/view/TPS00044/default/table?lang=en&category=hlth.hlth_care.hlth_res.hlth_staff%20%2F |
| | |
| **Proportion of health care expenditure on pharmaceuticals** | |
| Data - % of health care expenditure | 9.754 |
| Time | 2020 |
| Reference | https://data.oecd.org/healthres/pharmaceutical-spending.htm |
| | |
| **Number of practising pharmacists per 100,000 inhabitants** | |
| Data - N of practisisng pharmacists per 100,000 inhabitants | 79.38 |
| Time | 2019 |
| Reference | https://ec.europa.eu/eurostat/databrowser/view/HLTH_RS_PRS1__custom_4104351/default/table?lang=en |
| | |
| **Total health care expenditure as percentage of GDP** | |
| Data - % of GDP | 11.38 |
| Time | 2020 |
| Reference | https://ec.europa.eu/eurostat/databrowser/view/TPS00207/default/table?lang=en&category=hlth.hlth_care.hlth_sha11.hlth_sha11_sum |

| | | |
|---|---|---|
| | | |
| | **Public pharmaceutical expenditure as percentage of total pharmaceutical expenditure** | |
| | Data - % of total pharmaceutical expenditure | 58.3 |
| | Time | 2011 |
| | Reference | https://gateway.euro.who.int/en/indicators/hfa_580-6790-public-pharmaceutical-expenditure-as-of-total-pharmaceutical-expenditure/visualizations/#id=19675&tab=table |
| | | |
| | **Self-reported consultations of a medical doctor*** | |
| | Data - No contact, % of population according to the number of consultations of a medical doctor in the past 4 weeks | 66.9 |
| | Data - 1 contact, % of population according to the number of consultations of a medical doctor in the past 4 weeks | 12.9 |
| | Data - 2 contacts, % of population according to the number of consultations of a medical doctor in the past 4 weeks | 9.4 |
| | Data - 3 or more contacts, % of population according to the number of consultations of a medical doctor in the past 4 weeks | 10.7 |
| | Time | 2019 |
| | Reference | https://ec.europa.eu/eurostat/databrowser/view/HLTH_EHIS_AM2U/default/table?lang=en&category=hlth.hlth_care.hlth_consult / |

*Medical doctors include generalist medical practitioners and specialist medical practitioners

SWITZERLAND

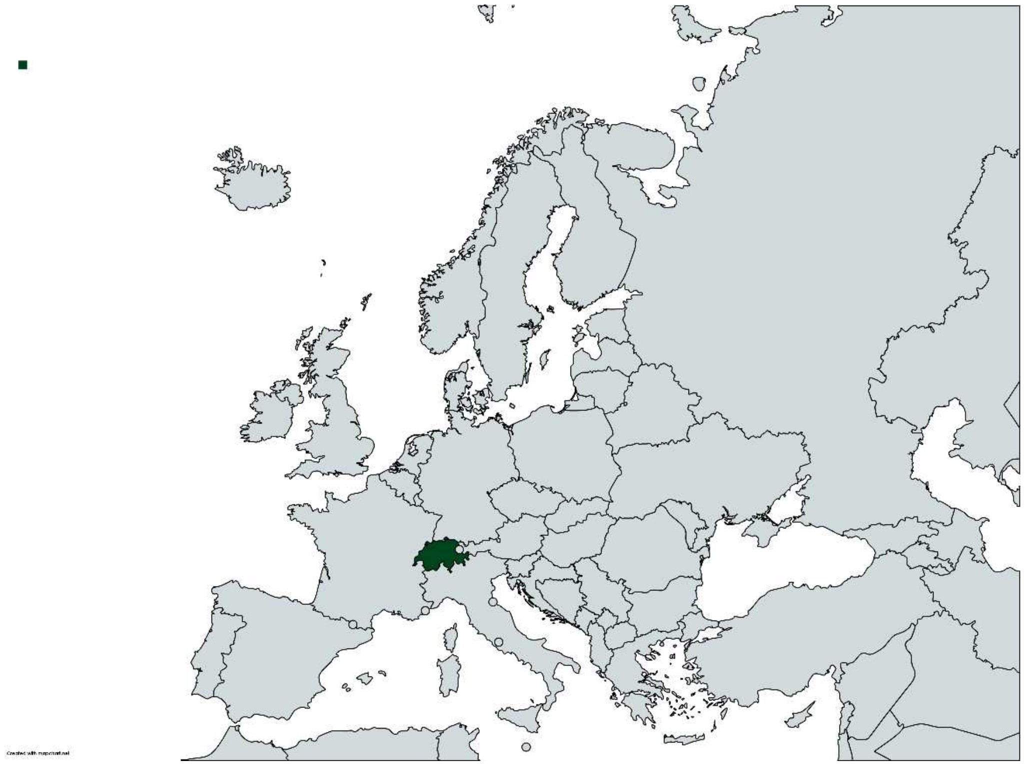

| Country | Switzerland |
|---|---|

Country-specific data

| Country characteristics | Method of payment | |
|---|---|---|
| | Data | Mandatory health insurance plan for all Swiss citizens. A monthly premium independent of salary, a yearly 300-Euro deductible + 10% of the costs exceeding deductible, up to a maximum of CHF 700/year |
| | Time | 2006 |
| | Reference | Guignard and Bugnon. Ann Pharmacother 2006;40:512-7 |
| | | |
| | Medication adherence assessed and reported on the national level | |
| | Data | No |
| | Time | 2022 |
| | Reference | NA |
| | | |
| | Health care provider | |
| | Data | NA |
| | Time | NA |
| | Reference | NA |
| | | |
| | Model of healthcare system financing | |
| | Data | Mandatory health insurance plan for all residents in Switzerland. Each resident pays a monthly premium independent of salary, + a yearly 300-Euro deductible (or more, depending on contract) + 10% of the costs exceeding deductible, up to a maximum of CHF 700/year per adult, 350 CHF/year per child |
| | Time | 2006 |
| | Reference | Guignard and Bugnon. Ann Pharmacother 2006;40:512-7 |
| | | |
| | Proportion of population aged 65 years and over | |
| | Data - % of persons | 19 |
| | Time | 2021 |
| | Reference | https://www.bfs.admin.ch/bfs/fr/home/statistiques/population/effectif-evolution/population.html |
| | | |
| | Country population (projection) | |
| | Data - N of persons | 8789726 |

| | | |
|---|---|---|
| | Time | 2022 |
| | Reference | https://www.bfs.admin.ch/bfs/fr/home/statistiques/population/effectif-evolution.gnpdetail.2022-0455.html |

| | | |
|---|---|---|
| **Social/economic factors** | **Patient co-payment** | |
| | Data | Each resident pays a yearly 300-Euro deductible (or more, depending on contract) + 10% of the costs exceeding deductible, up to a maximum of CHF 700/year per adult, 350 CHF/year per child |
| | Time | 2006 |
| | Reference | Guignard and Bugnon. Ann Pharmacother 2006;40:512-7 |
| | | |
| | **Percentage of prescriptions dispensed at no cost to patients** | |
| | Data - % of prescriptions | NA |
| | Time | NA |
| | Reference | NA |
| | | |
| | **Population coverage** | |
| | Data | 1 |
| | Time | 2023 |
| | Reference | Expert opinion |
| | | |
| | **Availability of doctors' services for citizens at no payment** | |
| | Data | NA |
| | Time | NA |
| | Reference | NA |
| | | |
| **Therapy-related factors** | **Average number of medicines per patient** | |
| | Data - N of medicines per patient | NA |
| | Time | NA |
| | Reference | NA |
| | | |
| | **Proportion of 75 years and over who are taking more than 5 medications concurrently** | |
| | Data - % of persons | 28,6% of patients between 75-84 years old and 38,3% of patients 85 years old and older |
| | Time | 2015 |
| | Reference | https://serval.unil.ch/resource/serval:BIB_A61083A8B4D6.P001/REF |
| | | |

| | | |
|---|---|---|
| | **Percentage of self-reported use of prescribed medicines** | |
| | Data - % of persons | NA |
| | Time | NA |
| | Reference | NA |
| **Patient-related factors** | **Percentage of persons reporting a chronic disease** | |
| | Data - Asthma, % of persons | 9 |
| | Data - Chronic lower respiratory diseases, % of persons | 7.1 |
| | Data - High blood pressure, % of persons | 16.4 |
| | Data - Diabetes, % of persons | 10.2 |
| | Data - Chronic depression, % of persons | 9 |
| | Time | 2019 |
| | Reference | https://ec.europa.eu/eurostat/databrowser/view/HLTH_EHIS_CD1E/default/table?lang=en&category=hlth.hlth_state.hlth_srcm / |
| | | |
| | **Percentage of self-perceived health - very good (16 years and over)** | |
| | Data - % of persons | 84.7% good to very good (not possible to distinguish very good from good) |
| | Time | 2017 |
| | Reference | https://www.bfs.admin.ch/bfs/en/home.assetdetail.8206064.html |
| | | |
| | **Percenatge of persons with current depressive symptoms** | |
| | Data - % of persons | 9 |
| | Time | 2017 |
| | Reference | https://www.bfs.admin.ch/bfs/fr/home/statistiques/sante/etat-sante/psychique.html |
| **Condition-related factors** | **General health literacy** | |
| | Data - Inadequate health literacy, % of persons | 11 |
| | Data - Problematic health literacy, % of persons | 38 |
| | Data - Sufficient health literacy, % of persons | 39 |
| | Data - Excellent health literacy, % of persons | 12 |
| | Time | 2021 |
| | Reference | https://www.bag.admin.ch/dam/bag/fr/dokumente/nat-gesundheitspolitik/gesundheitskompetenz/schlussbericht-health-literacy-survey-careum.pdf.download.pdf/HLS19-21-CH_Schlussbericht_Careum%20Gesundheitskompetenz_Health%20Literacy%20Survey_20210914.pdf |

| | | |
|---|---|---|
| Healthcare system-relat | **Percentage of patients receiving adherence interventions** | |
| | Data - % of persons | NA |
| | Time | NA |
| | Reference | NA |
| | | |
| | **Nationwide availability of e-prescription** | |
| | Data | NA |
| | Time | NA |
| | Reference | NA |
| | | |
| | **Waiting time for prescriptions / medical appointments** | |
| | Data | NA |
| | Time | NA |
| | Reference | NA |
| | | |
| | **Number of practising physicians per 100,000 inhabitants** | |
| | Data - N of practisisng physicians per 100,000 inhabitants | 445.51 |
| | Time | 2021 |
| | Reference | https://ec.europa.eu/eurostat/databrowser/view/TPS00044/default/table?lang=en&category=hlth.hlth_care.hlth_res.hlth_staff%20%2F |
| | | |
| | **Proportion of health care expenditure on pharmaceuticals** | |
| | Data - % of health care expenditure | 12.003 |
| | Time | 2020 |
| | Reference | https://data.oecd.org/healthres/pharmaceutical-spending.htm |
| | | |
| | **Number of practising pharmacists per 100,000 inhabitants** | |
| | Data - N of practisisng pharmacists per 100,000 inhabitants | 21 |
| | Time | 2021 |
| | Reference | https://www.pharmasuisse.org/data/docs/fr/45138/Faits-et-chiffres-pharmaSuisse-2021.pdf?v=1.0 |
| | | |
| | **Total health care expenditure as percentage of GDP** | |
| | Data - % of GDP | 11.8 |
| | Time | 2020 |

| | | |
|---|---|---|
| | Reference | https://ec.europa.eu/eurostat/databrowser/view/TPS00207/default/table?lang=en&category=hlth.hlth_care.hlth_sha11.hlth_sha11_sum |
| | | |
| | **Public pharmaceutical expenditure as percentage of total pharmaceutical expenditure** | |
| | Data - % of total pharmaceutical expenditure | 68.9 |
| | Time | 2011 |
| | Reference | https://gateway.euro.who.int/en/indicators/hfa_580-6790-public-pharmaceutical-expenditure-as-of-total-pharmaceutical-expenditure/visualizations/#id=19675&tab=table |
| | | |
| | **Self-reported consultations of a medical doctor*** | |
| | Data - No contact, % of population according to the number of consultations of a medical doctor in the past 4 weeks | NA |
| | Data - 1 contact, % of population according to the number of consultations of a medical doctor in the past 4 weeks | NA |
| | Data - 2 contacts, % of population according to the number of consultations of a medical doctor in the past 4 weeks | NA |
| | Data - 3 or more contacts, % of population according to the number of consultations of a medical doctor in the past 4 weeks | NA |
| | Time | NA |
| | Reference | NA |

*Medical doctors include generalist medical practitioners and specialist medical practitioners

TURKEY

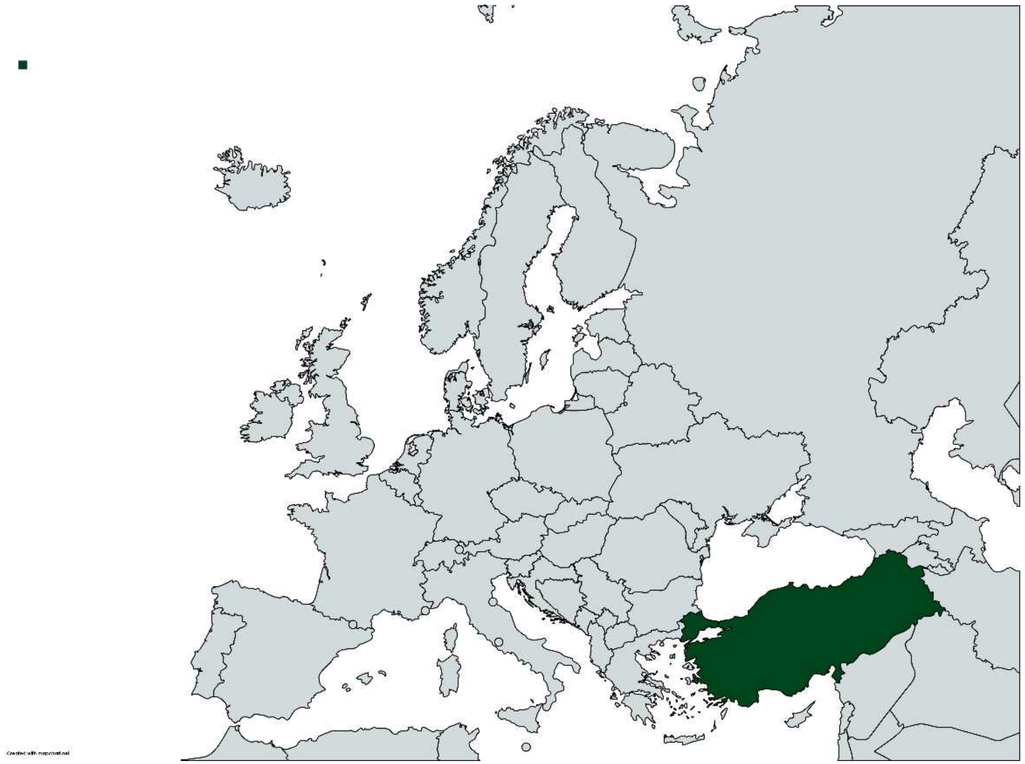

| Country | Turkey |
|---|---|

Country-specific data

| Country characteristics | Method of payment | |
|---|---|---|
| | Data | Health expenditures are reimbursed by the Social Security Institution of Turkey within the scope of universal health insurance. The contribution made by individuals for reimbursement of medication vary between 2% and 20%. |
| | Time | 2022 |
| | Reference | https://www.mevzuat.gov.tr/File/GeneratePdf?mevzuatNo=17229&mevzuatTur=Teblig&mevzuatTertip=5 |
| | | |
| | Medication adherence assessed and reported on the national level | |
| | Data | No |
| | Time | 2022 |
| | Reference | NA |
| | | |
| | Health care provider | |
| | Data | NA |
| | Time | NA |
| | Reference | NA |
| | | |
| | Model of healthcare system financing | |
| | Data | The Turkish healthcare system is financed by taxes/public (79%), insurance premiums (4%), and out-of-pocket payments (17%). |
| | Time | 2020 |
| | Reference | https://www.saglik.gov.tr/TR,89801/saglik-istatistikleri-yilligi-2020-yayinlanmistir.html |
| | | |
| | Proportion of population aged 65 years and over | |
| | Data - % of persons | 9.5 |
| | Time | 2021 |
| | Reference | https://ec.europa.eu/eurostat/databrowser/view/TPS00028/default/table?lang=en&category=demo.demo_ind / |
| | | |
| | Country population (projection) | |
| | Data - N of persons | 83614362 |
| | Time | 2020 |

| | | |
|---|---|---|
| | Reference | https://www.saglik.gov.tr/TR,89801/saglik-istatistikleri-yilligi-2020-yayinlanmistir.html |
| Social/economic factors | **Patient co-payment** | |
| | Data | Patient co-payment for medicine is vary between 2% and 20%.<br>These are exempted from co-payment;<br>A) In-patient treatment and medications<br>b) Medications for chronic diseases, |
| | Time | 2022 |
| | Reference | https://www.mevzuat.gov.tr/File/GeneratePdf?mevzuatNo=17229&mevzuatTur=Teblig&mevzuatTertip=5 |
| | | |
| | **Percentage of prescriptions dispensed at no cost to patients** | |
| | Data - % of prescriptions | NA |
| | Time | NA |
| | Reference | NA |
| | | |
| | **Population coverage** | |
| | Data | 0.91 |
| | Time | 2020 |
| | Reference | https://www.saglik.gov.tr/TR,89801/saglik-istatistikleri-yilligi-2020-yayinlanmistir.html |
| | | |
| | **Availability of doctors' services for citizens at no payment** | |
| | Data | Medical doctor and dentist outpatient treatments at the State primary healthcare service providers and family doctors. |
| | Time | 2020 |
| | Reference | https://www.saglik.gov.tr/TR,89801/saglik-istatistikleri-yilligi-2020-yayinlanmistir.html |
| | | |
| Therapy-related factors | **Average number of medicines per patient** | |
| | Data - N of medicines per patient | NA |
| | Time | NA |
| | Reference | NA |
| | | |
| | **Proportion of 75 years and over who are taking more than 5 medications concurrently** | |
| | Data - % of persons | 9.9 |
| | Time | 2019 |

| | | |
|---|---|---|
| | Reference | https://stats.oecd.org/Index.aspx?ThemeTreeId=46 |
| | | |
| | **Percentage of self-reported use of prescribed medicines** | |
| | Data - % of persons | NA |
| | Time | NA |
| | Reference | NA |

| | | |
|---|---|---|
| **Patient-related factors** | **Percentage of persons reporting a chronic disease** | |
| | Data - Asthma, % of persons | 0.3-7.6 |
| | Data - Chronic lower respiratory diseases, % of persons | 45048 |
| | Data - High blood pressure, % of persons | 30.3 |
| | Data - Diabetes, % of persons | 14.5 |
| | Data - Chronic depression, % of persons | 9.3 |
| | Time | NA |
| | Reference | https://hsgm.saglik.gov.tr/depo/birimler/kronik-hastaliklar-engelli-db/hastaliklar/kronik_havayolu/raporlar/KRONIK_ARASTIRMA_TEMEL_BULGULAR.pdf, https://sbu.saglik.gov.tr/Ekutuphane/Yayin/507, https://journals.lww.com/jhypertension/Fulltext/2016/06000/Changes_in_hypertension_prevalence,_awareness,.27.aspx |
| | | |
| | **Percentage of self-perceived health - very good (16 years and over)** | |
| | Data - % of persons | 6.5 |
| | Time | 2020 |
| | Reference | https://ec.europa.eu/eurostat/databrowser/view/HLTH_SILC_02/default/table?lang=en&category=hlth.hlth_state.hlth_sph/ |
| | | |
| | **Percenatge of persons with current depressive symptoms** | |
| | Data - % of persons | 6.6 |
| | Time | 2019 |
| | Reference | https://ec.europa.eu/eurostat/databrowser/view/HLTH_EHIS_MH1E/default/table?lang=en&category=hlth.hlth_state.hlth_sph / |

| | | |
|---|---|---|
| **Condition-related factor** | **General health literacy** | |
| | Data - Inadequate health literacy, % of persons | NA |
| | Data - Problematic health literacy, % of persons | NA |
| | Data - Sufficient health literacy, % of persons | NA |
| | Data - Excellent health literacy, % of persons | NA |

| | | |
|---|---|---|
| | Time | NA |
| | Reference | NA |

| | | |
|---|---|---|
| **Healthcare system-relat** | **Percentage of patients receiving adherence interventions** | |
| | Data - % of persons | NA |
| | Time | NA |
| | Reference | NA |
| | | |
| | **Nationwide availability of e-prescription** | |
| | Data | Yes |
| | Time | 2022 |
| | Reference | Expert opinion |
| | | |
| | **Waiting time for prescriptions / medical appointments** | |
| | Data | NA |
| | Time | NA |
| | Reference | NA |
| | | |
| | **Number of practising physicians per 100,000 inhabitants** | |
| | Data - N of practisisng physicians per 100,000 inhabitants | 205 |
| | Time | 2019 |
| | Reference | https://www.saglik.gov.tr/TR,89801/saglik-istatistikleri-yilligi-2020-yayinlanmistir.html |
| | | |
| | **Proportion of health care expenditure on pharmaceuticals** | |
| | Data - % of health care expenditure | NA |
| | Time | NA |
| | Reference | NA |
| | | |
| | **Number of practising pharmacists per 100,000 inhabitants** | |
| | Data - N of practisisng pharmacists per 100,000 inhabitants | https://www.pharmasuisse.org/data/docs/fr/45138/Faits-et-chiffres-pharmaSuisse-2021.pdf?v=1.0 |
| | Time | 2019 |
| | Reference | https://www.saglik.gov.tr/TR,89801/saglik-istatistikleri-yilligi-2020-yayinlanmistir.html |
| | | |

| | | |
|---|---|---|
| | **Total health care expenditure as percentage of GDP** | |
| | Data - % of GDP | 4.9 |
| | Time | 2021 |
| | Reference | https://data.tuik.gov.tr/Bulten/Index?p=Saglik-Harcamalari-Istatistikleri-2021-45728 |
| | | |
| | **Public pharmaceutical expenditure as percentage of total pharmaceutical expenditure** | |
| | Data - % of total pharmaceutical expenditure | 34.55 |
| | Time | 2021 |
| | Reference | https://www.sgk.gov.tr/Istatistik/Yillik/fcd5e59b-6af9-4d90-a451-ee7500eb1cb4/ |
| | | |
| | **Self-reported consultations of a medical doctor*** | |
| | Data - No contact, % of population according to the number of consultations of a medical doctor in the past 4 weeks | 47.9 |
| | Data - 1 contact, % of population according to the number of consultations of a medical doctor in the past 4 weeks | 21.4 |
| | Data - 2 contacts, % of population according to the number of consultations of a medical doctor in the past 4 weeks | 12.1 |
| | Data - 3 or more contacts, % of population according to the number of consultations of a medical doctor in the past 4 weeks | 18.6 |
| | Time | 2019 |
| | Reference | https://ec.europa.eu/eurostat/databrowser/view/HLTH_EHIS_AM2U/default/table?lang=en&category=hlth.hlth_care.hlth_consult / |

*Medical doctors include generalist medical practitioners and specialist medical practitioners

UKRAINE

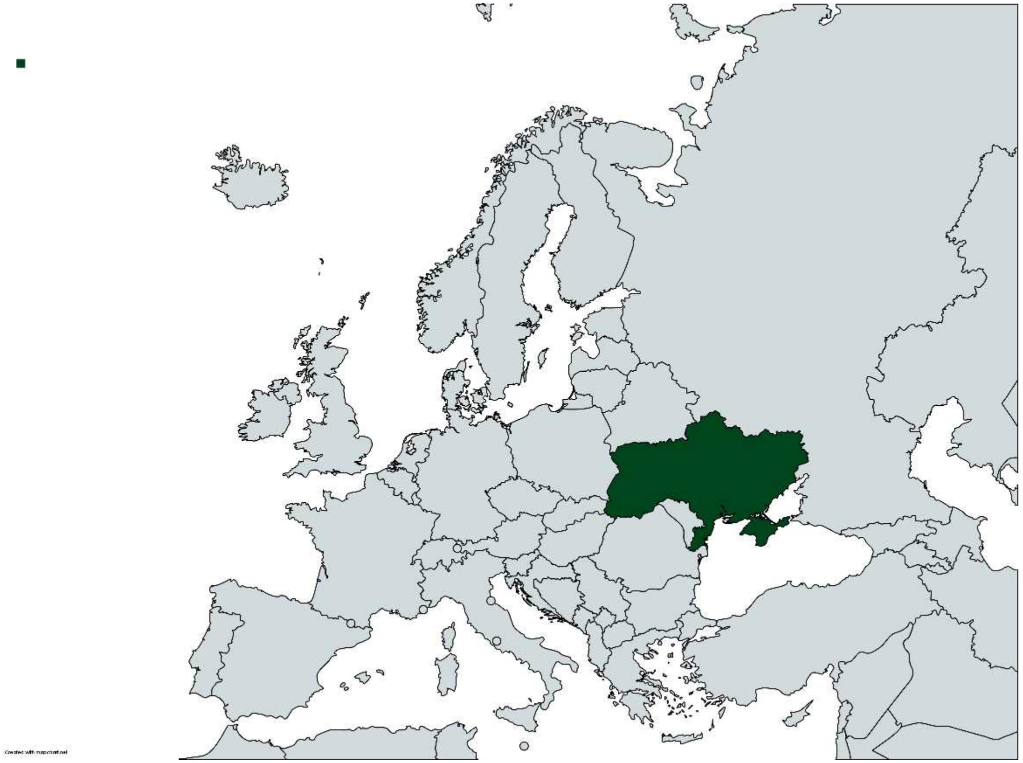

| Country | Ukraine |
|---|---|

Country-specific data

| Country characteristics | Method of payment | |
|---|---|---|
| | Data | The National Health Service of Ukraine (NHSU) is the central executive body that implements the state policy regarding state financial guarantees for the healthcare servicing of the population. Activities of the NHSU are directed and coordinated by the Cabinet of Ministers of Ukraine through the Minister of Health. |
| | Time | 2023 |
| | Reference | https://zakon.rada.gov.ua/laws/show/en/2168-19?lang=en#Text |
| | | |
| | Medication adherence assessed and reported on the national level | |
| | Data | No |
| | Time | 2022 |
| | Reference | Expert opinion |
| | | |
| | Health care provider | |
| | Data | providers of medical service – healthcare institutions of all forms of ownership and individuals – entrepreneurs who have obtained a license to conduct business in medical practice and who have entered into a contract for medical service to the population with the chief administrators of budgetary funds; |
| | Time | 2023 |
| | Reference | https://zakon.rada.gov.ua/laws/show/en/2168-19?lang=en#Text |
| | | |
| | Model of healthcare system financing | |
| | Data | The healthcare financing model includes the introduction of a state-guaranteed package of medical care, the creation of a single national customer of medical services, the autonomy of medical care providers, new payment methods, a new service quality management system, and the introduction of electronic healthcare. |
| | Time | 2022 |
| | Reference | https://www.kmu.gov.ua/npas/249626689 |
| | | |
| | Proportion of population aged 65 years and over | |
| | Data - % of persons | 18.8 |
| | Time | 2022 |
| | Reference | https://knoema.com/atlas/Ukraine/Population-aged-65-years-and-above |
| | | |
| | Country population (projection) | |

| | | |
|---|---|---|
| | Data - N of persons | 40997698 |
| | Time | 2022 |
| | Reference | http://db.ukrcensus.gov.ua/PXWEB2007/ukr/publ_new1/2022/roz_nas22.pdf |

| | | |
|---|---|---|
| **Social/economic factors** | **Patient co-payment** | |
| | Data | The reimbursement system covers 462 medicines for cardiovascular diseases, type II diabetes, insulins, mental and behavioral disorders, epilepsy; bronchial asthma, diabetes insipidus, Parkinson's disease, and chronic obstructive pulmonary disease, 143 from them for free and the rest with co-payment. |
| | Time | 2022 |
| | Reference | https://moz.gov.ua/article/ministry-mandates/nakaz-moz-ukraini-vid-27102022--1931-pro-zatverdzhennja-reestru-likarskih-zasobiv-jaki-pidljagajut-reimbursacii-za-programoju-derzhavnih-garantij-medichnogo-obslugovuvannja-naselennja-stanom-na-20-zhovtnja-2022-roku?preview=1 |
| | | |
| | **Percentage of prescriptions dispensed at no cost to patients** | |
| | Data - % of prescriptions | Total 10,379,445 reimbursed prescriptions in 2022. Their cost was 2,454,108,551 UAH. |
| | Time | the end of 2022 |
| | Reference | https://edata.e-health.gov.ua/e-data/dashboard/reimb-manufacturer-details |
| | | |
| | **Population coverage** | |
| | Data | 83% of population made an agreement with a family doctor. |
| | Time | 2022 |
| | Reference | https://edata.e-health.gov.ua/e-data/dashboard/pmd-coverage-indicators |
| | | |
| | **Availability of doctors' services for citizens at no payment** | |

| | | |
|---|---|---|
| | Data | 37 medical services. 1. Emergency medical care; 2. Primary medical care; 3. Management of pregnancy in outpatient settings; 4. Diagnosis, treatment, and support of persons with immunodeficiency virus; 5. Diagnosis and treatment of adults and children with tuberculosis; 6. Treatment of persons with mental and behavioral disorders due to the use of opioids with the help of substitute maintenance therapy drugs; 7. Treatment and support of patients with hematological and oncohematological diseases in inpatient and outpatient settings; 8. Medical care for acute myocardial infarction; 9. Medical care for acute cerebral stroke; 10. Mobile palliative medical care for adults and children; 11. Prevention, diagnosis, monitoring, and treatment of adults and children in outpatient settings; 12. Psychiatric care for adults and children provided by mobile multidisciplinary teams; 13. Radiological treatment and support of patients with oncological diseases in inpatient and outpatient settings; 14. Inpatient care for adults and children without surgical operations; 15. Inpatient palliative medical care for adults and children; 16. Dental care for adults and children; 17. Support and treatment of adults and children with tuberculosis at the primary level of medical care; 18. Chemotherapeutic treatment and support of patients with oncological diseases in inpatient and outpatient settings; 19. Surgical operations for adults and children in one-day hospital conditions; 20. Surgical operations for adults and children in hospital conditions; 21. Bronchoscopy; 22. Hysteroscopy; 23. Esophagogastroduodenoscopy; 24. Colonoscopy; 25. Treatment of patients by the hemodialysis method in outpatient settings; 26. Treatment of patients by peritoneal dialysis method in outpatient settings; 27. Mammography; 28. Medical assistance during childbirth; 29. Medical care for newborns in complicated neonatal cases; 30. Medical rehabilitation of babies born prematurely and/or sick; 31. Cystoscopy; 32. Neonatal screening; 33. Support and treatment of adults and children with mental disorders at the primary level of medical care; 34. Ensuring the personnel potential of the health care system by organizing the provision of medical care with the involvement of intern doctors; 35. Readiness of the health care institution to provide medical assistance in emergencies; 36. Cross-sectional study; 37. Psychiatric care for adults and children in inpatient settings. |
| | Time | 2023 |
| | Reference | https://contracting.nszu.gov.ua/vimogi-pmg-paket |
| | | |
| **Therapy-related factors** | **Average number of medicines per patient** | |
| | Data - N of medicines per patient | NA |
| | Time | NA |
| | Reference | NA |
| | | |
| | **Proportion of 75 years and over who are taking more than 5 medications concurrently** | |
| | Data - % of persons | NA |
| | Time | NA |
| | Reference | NA |
| | | |
| | **Percentage of self-reported use of prescribed medicines** | |
| | Data - % of persons | NA |
| | Time | NA |
| | Reference | NA |
| | | |
| **Patient-related factors** | **Percentage of persons reporting a chronic disease** | |

| | | |
|---|---|---|
| | Data - Asthma, % of persons | NA |
| | Data - Chronic lower respiratory diseases, % of persons | NA |
| | Data - High blood pressure, % of persons | NA |
| | Data - Diabetes, % of persons | NA |
| | Data - Chronic depression, % of persons | NA |
| | Time | NA |
| | Reference | NA |
| | | |
| | **Percentage of self-perceived health - very good (16 years and over)** | |
| | Data - % of persons | NA |
| | Time | NA |
| | Reference | NA |
| | | |
| | **Percenatge of persons with current depressive symptoms** | |
| | Data - % of persons | NA |
| | Time | NA |
| | Reference | NA |

| | | |
|---|---|---|
| **Condition-related factor** | **General health literacy** | |
| | Data - Inadequate health literacy, % of persons | NA |
| | Data - Problematic health literacy, % of persons | NA |
| | Data - Sufficient health literacy, % of persons | NA |
| | Data - Excellent health literacy, % of persons | NA |
| | Time | NA |
| | Reference | NA |

| | | |
|---|---|---|
| **Healthcare system-relat** | **Percentage of patients receiving adherence interventions** | |
| | Data - % of persons | NA |
| | Time | NA |
| | Reference | NA |
| | | |
| | **Nationwide availability of e-prescription** | |

| | |
|---|---|
| Data | Currently, e-prescription is available for 462 medications for cardiovascular diseases, type II diabetes, insulins, mental and behavioral disorders, epilepsy; bronchial asthma, diabetes insipidus, Parkinson's disease, and chronic obstructive pulmonary disease), antibacterial medicines (paid by the patients themself) and narcotics (paid by the patients themself) |
| Time | 2023 |
| Reference | https://edata.e-health.gov.ua/e-data/dashboard/reimb-w-prescriptions |
| | |
| **Waiting time for prescriptions / medical appointments** | |
| Data | NA |
| Time | NA |
| Reference | NA |
| | |
| **Number of practising physicians per 100,000 inhabitants** | |
| Data - N of practisisng physicians per 100,000 inhabitants | 65.5 (primary medical care) |
| Time | 2022 |
| Reference | https://edata.e-health.gov.ua/e-data/dashboard/pmd-coverage-indicators |
| | |
| **Proportion of health care expenditure on pharmaceuticals** | |
| Data - % of health care expenditure | NA |
| Time | NA |
| Reference | NA |
| | |
| **Number of practising pharmacists per 100,000 inhabitants** | |
| Data - N of practisisng pharmacists per 100,000 inhabitants | NA |
| Time | NA |
| Reference | NA |
| | |
| **Total health care expenditure as percentage of GDP** | |
| Data - % of GDP | 4.8 |
| Time | 2022 |
| Reference | https://www.kmu.gov.ua/news/minfin-za-6-misiatsiv-vydatky-derzhavnoho-biudzhetu-na-okhoronu-zdorovia-zdiisneno-v-obsiazi-822-mlrd-hryven |
| | |
| **Public pharmaceutical expenditure as percentage of total pharmaceutical expenditure** | |
| Data - % of total pharmaceutical expenditure | NA |

| | | |
|---|---|---|
| | Time | NA |
| | Reference | NA |
| | | |
| | **Self-reported consultations of a medical doctor*** | |
| | Data - No contact, % of population according to the number of consultations of a medical doctor in the past 4 weeks | NA |
| | Data - 1 contact, % of population according to the number of consultations of a medical doctor in the past 4 weeks | NA |
| | Data - 2 contacts, % of population according to the number of consultations of a medical doctor in the past 4 weeks | NA |
| | Data - 3 or more contacts, % of population according to the number of consultations of a medical doctor in the past 4 weeks | NA |
| | Time | NA |
| | Reference | NA |

*Medical doctors include generalist medical practitioners and specialist medical practitioners

UNITED KINGDOM

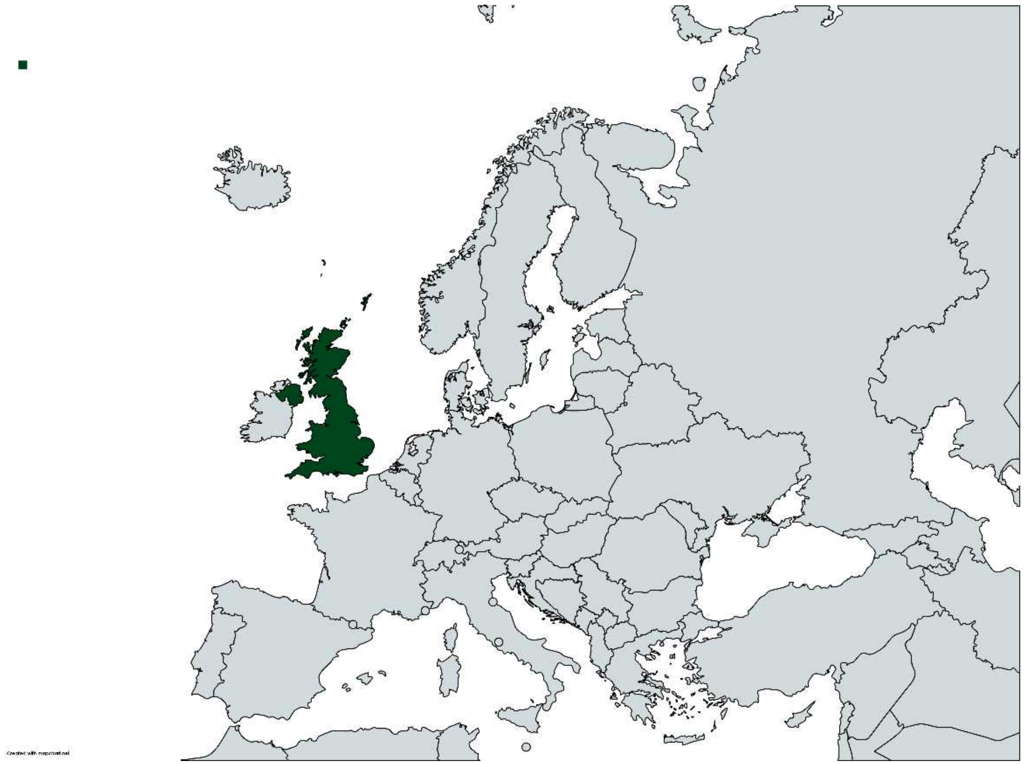

| Country | United Kingdom |
|---|---|

Country-specific data

| Country characteristics | Method of payment | |
|---|---|---|
| | Data | The National Health System pays the cost of medicines. |
| | Time | 2013 |
| | Reference | http://imi-protect.eu/documents/DUinventory_2013_COUNTRIESyear4_Dec2013.pdf |
| | | |
| | **Medication adherence assessed and reported on the national level** | |
| | Data | No |
| | Time | 2022 |
| | Reference | NA |
| | | |
| | **Health care provider** | |
| | Data | Public. Universal access to health care. About 11% of the population is covered by a private health insurance. |
| | Time | 2013 |
| | Reference | http://imi-protect.eu/documents/DUinventory_2013_COUNTRIESyear4_Dec2013.pdf |
| | | |
| | **Model of healthcare system financing** | |
| | Data | Predominantly the government through general taxation, compulsory contributions to the NHS by employers, employees, self-employed and some local taxation. The first 2 sources of financing represent 94.6% of the NHS financing. The rest is financed through out of pocket payments and private health insurance. |
| | Time | 2013 |
| | Reference | http://imi-protect.eu/documents/DUinventory_2013_COUNTRIESyear4_Dec2013.pdf |
| | | |
| | **Proportion of population aged 65 years and over** | |
| | Data - % of persons | 18.4 |
| | Time | 2019 |
| | Reference | https://ec.europa.eu/eurostat/databrowser/view/TPS00028/default/table?lang=en&category=demo.demo_ind / |
| | | |
| | **Country population (projection)** | |
| | Data - N of persons | NA |
| | Time | NA |

| | | |
|---|---|---|
| | Reference | NA |
| Social/economic factors | **Patient co-payment** | |
| | Data | The patient pays a flat fee per prescription. There is the possibility to buy a prescription pre-payment certificate for 3 or 12 months to get a discount on the fee per prescription. Medicines free of charge are exempted to the flatfee per prescription: prescribed contraceptives, medication personally administered by a general practitioner, medication supplied at a hospital or Primary Care Trust for sexually transmitted infections or Tuberculosis. People aged >60 years, <16 years, or between 16-18 years and in full-time education. Conditions that grant a maternity exemption certificate or a medical exemption certificate. People with a valid war pensions exemption certificate and the prescription is for the accepted disability. People entitled to an NHS tax credit exemption certificate, or a valid HC2 certificate or if they receive income support. |
| | Time | 2013 |
| | Reference | http://imi-protect.eu/documents/DUinventory_2013_COUNTRIESyear4_Dec2013.pdf |
| | | |
| | **Percentage of prescriptions dispensed at no cost to patients** | |
| | Data - % of prescriptions | NA |
| | Time | NA |
| | Reference | NA |
| | | |
| | **Population coverage** | |
| | Data | All residents in the United Kingdom (UK) are eligible for the National Health System (NHS). |
| | Time | 2013 |
| | Reference | http://imi-protect.eu/documents/DUinventory_2013_COUNTRIESyear4_Dec2013.pdf |
| | | |
| | **Availability of doctors' services for citizens at no payment** | |
| | Data | NA |
| | Time | NA |
| | Reference | NA |
| | | |
| Therapy-related factors | **Average number of medicines per patient** | |
| | Data - N of medicines per patient | NA |
| | Time | NA |
| | Reference | NA |
| | | |
| | **Proportion of 75 years and over who are taking more than 5 medications concurrently** | |

| | | |
|---|---|---|
| | Data - % of persons | NA |
| | Time | NA |
| | Reference | NA |
| | | |
| | **Percentage of self-reported use of prescribed medicines** | |
| | Data - % of persons | 53 |
| | Time | 2014 |
| | Reference | https://ec.europa.eu/eurostat/databrowser/view/HLTH_EHIS_MD1E__custom_3764895/default/table?lang=en / |

| | | |
|---|---|---|
| **Patient-related factors** | **Percentage of persons reporting a chronic disease** | |
| | Data - Asthma, % of persons | NA |
| | Data - Chronic lower respiratory diseases, % of persons | NA |
| | Data - High blood pressure, % of persons | NA |
| | Data - Diabetes, % of persons | NA |
| | Data - Chronic depression, % of persons | NA |
| | Time | NA |
| | Reference | NA |
| | | |
| | **Percentage of self-perceived health - very good (16 years and over)** | |
| | Data - % of persons | 31.8 |
| | Time | 2018 |
| | Reference | https://ec.europa.eu/eurostat/databrowser/view/HLTH_SILC_02/default/table?lang=en&category=hlth.hlth_state.hlth_sph/ |
| | | |
| | **Percenatge of persons with current depressive symptoms** | |
| | Data - % of persons | NA |
| | Time | NA |
| | Reference | NA |

| | | |
|---|---|---|
| **Condition-related factor[illegible]** | **General health literacy** | |
| | Data - Inadequate health literacy, % of persons | NA |
| | Data - Problematic health literacy, % of persons | NA |
| | Data - Sufficient health literacy, % of persons | NA |
| | Data - Excellent health literacy, % of persons | NA |

| | | |
|---|---|---|
| | Time | NA |
| | Reference | NA |

| | | |
|---|---|---|
| Healthcare system-relat | **Percentage of patients receiving adherence interventions** | |
| | Data - % of persons | NA |
| | Time | NA |
| | Reference | NA |
| | | |
| | **Nationwide availability of e-prescription** | |
| | Data | NA |
| | Time | NA |
| | Reference | NA |
| | | |
| | **Waiting time for prescriptions / medical appointments** | |
| | Data | NA |
| | Time | NA |
| | Reference | NA |
| | | |
| | **Number of practising physicians per 100,000 inhabitants** | |
| | Data - N of practisisng physicians per 100,000 inhabitants | 284.05 |
| | Time | 2018 |
| | Reference | https://ec.europa.eu/eurostat/databrowser/view/TPS00044/default/table?lang=en&category=hlth.hlth_care.hlth_res.hlth_staff%20%2F |
| | | |
| | **Proportion of health care expenditure on pharmaceuticals** | |
| | Data - % of health care expenditure | 11.759 |
| | Time | 2020 |
| | Reference | https://data.oecd.org/healthres/pharmaceutical-spending.htm |
| | | |
| | **Number of practising pharmacists per 100,000 inhabitants** | |
| | Data - N of practisisng pharmacists per 100,000 inhabitants | 89.83 |
| | Time | 2018 |
| | Reference | https://ec.europa.eu/eurostat/databrowser/view/HLTH_RS_PRS1__custom_4104351/default/table?lang=en |
| | | |

| | | |
|---|---|---|
| | **Total health care expenditure as percentage of GDP** | |
| | Data - % of GDP | NA |
| | Time | NA |
| | Reference | NA |
| | | |
| | **Public pharmaceutical expenditure as percentage of total pharmaceutical expenditure** | |
| | Data - % of total pharmaceutical expenditure | 61.2 |
| | Time | 2019 |
| | Reference | https://gateway.euro.who.int/en/indicators/hfa_580-6790-public-pharmaceutical-expenditure-as-of-total-pharmaceutical-expenditure/visualizations/#id=19675&tab=table |
| | | |
| | **Self-reported consultations of a medical doctor*** | |
| | Data - No contact, % of population according to the number of consultations of a medical doctor in the past 4 weeks | NA |
| | Data - 1 contact, % of population according to the number of consultations of a medical doctor in the past 4 weeks | NA |
| | Data - 2 contacts, % of population according to the number of consultations of a medical doctor in the past 4 weeks | NA |
| | Data - 3 or more contacts, % of population according to the number of consultations of a medical doctor in the past 4 weeks | NA |
| | Time | NA |
| | Reference | NA |

*Medical doctors include generalist medical practitioners and specialist medical practitioners
